# Supplementary material for: Generation of DNA oligomers with similar chemical kinetics via in-silico optimization
Source: Commun Chem. 2023 Oct 18;6:226. doi: 10.1038/s42004-023-01026-w (PMC10584830; doi:10.1038/s42004-023-01026-w)
Supplement: Supplementary file 2 — Supplementary Information [file 42004_2023_1026_MOESM2_ESM.pdf]

# Generation of DNA Oligomers with Similar Chemical Kinetics via In-Silico Optimization

## Supplementary Material

**Contents**

Supplementary Note 1: Analysis of Existing Experimental Data........................................................... 1

Supplementary Note 2: Generation of New Oligomers (*SeqEvo Program*)........................................ 15

Supplementary Note 3: Calculation of Fitness Scores (*DevPro Program*) .......................................... 21

Supplementary Note 4: Characterization of New Oligomers .............................................................. 25

Supplementary Note 5: New Oligomers for Existing Designs............................................................ 109

Supplementary Note 6: Completion Time Calculations..................................................................... 124

# Supplementary Note 1: Analysis of Existing Experimental Data

**Table S1**. Select properties of the five datasets. Properties include: the total number of samples (n), the experimental temperature (T), the mean of the natural log of the rates (mean ln k), and kinetic dispersion – the Inter Quartile Range of the Natural Log of the rates (IQRNL).

| DataSet | n | T | Reaction | Units of k | Buffer | Mean Ln k | IQRNL | Median ΔN | Median ΔO |
|---|---|---|---|---|---|---|---|---|---|
| **H22F** | 47 | 22C | Duplex-Formation | $M^{-1}s^{-1}$ | 1x saline-sodium-citrate (150mM NaCl, 15mM sodium citrate) | 13.1 | 2.56 | 63100 | 5000 |
| **O25C** | 51 | 25C | Catalysis | $M^{-2}s^{-1}$ | 1 x TE (10mM Tris-HCl, 1 mM ethylenediaminetetraacetic acid, 12.5 mM $MgCl_2$) | 28.8 | 3.25 | 1061598 | 74840 |
| **O25L** | 51 | 25C | Leak | $M^{-1}s^{-1}$ | 1 x TE (10mM Tris-HCl, 1 mM ethylenediaminetetraacetic acid, 12.5 mM $MgCl_2$) | 1.14 | 1.09 | 1061598 | 74840 |
| **Z37F** | 98 | 37C | Duplex-Formation | $M^{-1}s^{-1}$ | 5 x Phosphate-Buffered-Saline | 14.0 | 1.86 | 2851380 | 113510 |
| **Z55F** | 95 | 55C | Duplex-Formation | $M^{-1}s^{-1}$ | 5 x Phosphate-Buffered-Saline | 15.4 | 1.29 | 2808020 | 118940 |

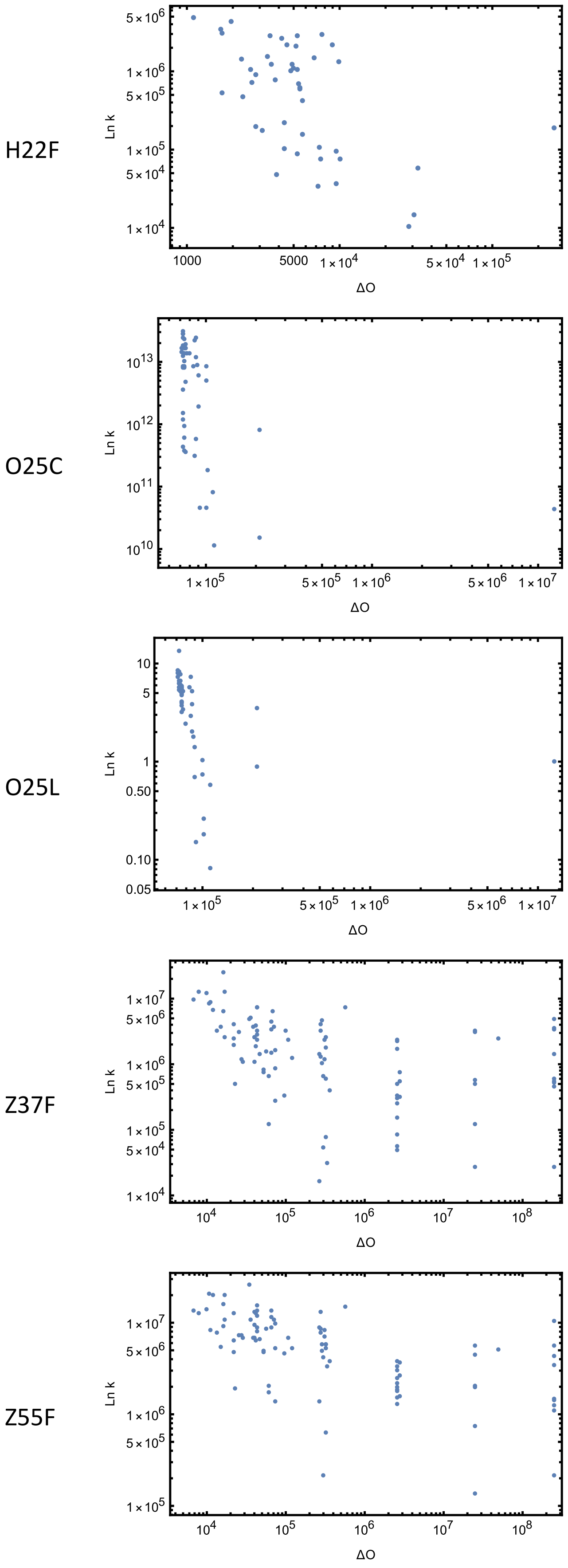


**Figure S1.** Correlation plots of the rate constants in each dataset as a function of ΔO.

Dataset: H22F

Selection Criterion: Smallest Delta-O

| Number of Samples (n) | | 47 (All) | 16 | 8 | 4 | 3 | 2 |
|---|---|---|---|---|---|---|---|

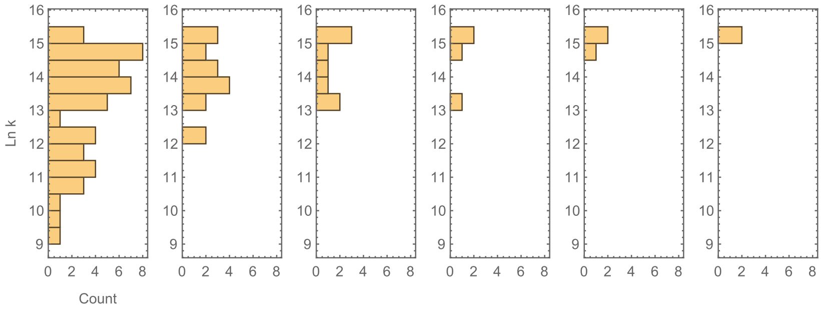


| | | | | | | | |
|---|---|---|---|---|---|---|---|
| Histogram | | | | | | | |
| p-value (KS vs All) | | 1. | 0.0370959 | 0.0427299 | 0.016741 | 0.00049337 | 0.00555042 |
| p-value (WSR vs All) | | 0.899417 | 0.0318798 | 0.0423153 | 0.201243 | 0.181449 | 0.371093 |
| p-value (AD vs All) | | 1. | 0.00837368 | 0.00254122 | 0.00185893 | 0.000161651 | 0.000309616 |
| Mean ln k | | 13.0824 | 13.9424 | 14.3673 | 14.6417 | 15.1331 | 15.2262 |
| Median ln k | | 13.5008 | 13.9352 | 14.56 | 15.0076 | 15.0683 | 15.2262 |
| Exact IQRNL: | | 2.56249 | 1.56195 | 1.65538 | 1.16908 | 0.32791 | 0.315853 |
| IQRNL Estimate: | $25^{th}$ percentile | 2.27017 | 1.17509 | 1.10293 | 0.315853 | 0.0910206 | 0. |
| | $50^{th}$ percentile | 2.55559 | 1.44611 | 1.44614 | 1.01115 | 0.23689 | 0.315853 |
| | $75^{th}$ percentile | 2.78166 | 1.72695 | 1.81252 | 1.29666 | 0.32791 | 0.315853 |
| IQRNL Estimate of n Random: | $25^{th}$ percentile | 2.19637 | 1.84063 | 1.43664 | 0.935709 | 0.371491 | 0. |
| | $50^{th}$ percentile | 2.51585 | 2.34456 | 2.07101 | 1.66014 | 1.44016 | 0. |
| | $75^{th}$ percentile | 2.74199 | 2.77869 | 2.70051 | 2.16905 | 1.9953 | 1.51698 |
| Largest Intra (bp) | | 5. | 2. | 2. | 2. | 2. | 2. |
| Largest Inter (bp) | | 7. | 5. | 5. | 4. | 4. | 4. |
| Mean ΔO | | 11 810.2 | 2563.75 | 1917.5 | 1540. | 1486.67 | 1380. |
| Median ΔO | | 5000. | 2630. | 1830. | 1680. | 1660. | 1380. |
| Mean ΔN | | 983 659. | 71 698.8 | 67 597.5 | 40 560. | 48 573.3 | 65 200. |
| Median ΔN | | 63 100. | 51 560. | 28 730. | 40 800. | 65 080. | 65 200. |

**Figure S2.** Statistical analysis of the rate constants for the most ΔO-fit samples in the H22F dataset. Statistical analyses were performed independently for each sample size (n). A Kolmogorov-Smirnov (KS), Wilcoxon-Singed-Rank (WRS), and Anderson-Darling (AD) test were performed comparing the distribution of the most-fit samples to the distribution of the other samples. For the WSR test, the median of 100 tests involving random pairings is reported. The "Exact IQRNL" value represents the IQRNL calculated directly from the rate-constant distribution without estimation. The "IQRNL Estimate" values represent the $25^{th}$,$50^{th}$, and $75^{th}$ percentiles of 1,000 random resamplings of the distribution. The "IQRNL Estimate of n Random" values represent the median $25^{th}$,$50^{th}$, and $75^{th}$ percentiles of 1,000 random resamplings of 10,000 randomly selected sub-populations of n samples. The largest-intra and largest-inter rows report the size of the largest unnecessary duplexes in base pairs. Values in this table may differ slightly from the values reported in table S1-01 due to the random sampling involved. The values in table S1-01 are the values reported and used in the manuscript text.

Dataset: O25C
Selection Criterion: Smallest Delta-O

| Number of Samples (n) | | 51 (All) | 16 | 8 | 4 | 3 | 2 |
|---|---|---|---|---|---|---|---|
| Histogram | | 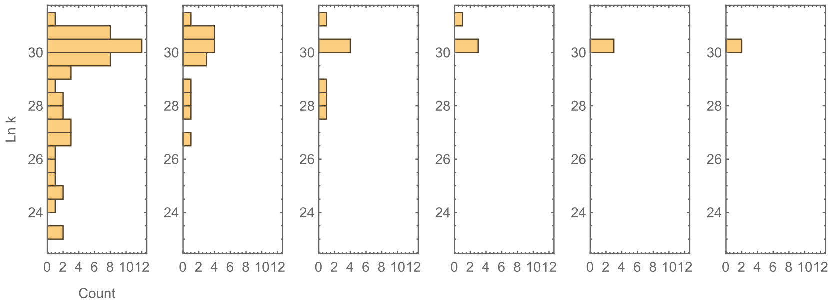 | | | | | |
| p-value (KS vs All) | | 1. | 0.124409 | 0.299739 | 0.0148459 | 0.0537815 | 0.164706 |
| p-value (WSR vs All) | | 0.891185 | 0.0524917 | 0.505958 | 0.201243 | 0.422678 | 0.371093 |
| p-value (AD vs All) | | 1. | 0.02118 | 0.256133 | 0.0130823 | 0.0701069 | 0.1223 |
| Mean ln k | | 28.7737 | 29.7893 | 29.6343 | 30.5345 | 30.3513 | 30.3812 |
| Median ln k | | 29.7781 | 30.228 | 30.228 | 30.3812 | 30.2982 | 30.3812 |
| Exact IQRNL: | | 3.24702 | 1.36756 | 1.91022 | 0.479441 | 0.129715 | 0.165985 |
| IQRNL Estimate: | $25^{th}$ percentile | 2.68637 | 0.927589 | 1.1858 | 0.165985 | 0.0052265 | 0. |
| | $50^{th}$ percentile | 3.15291 | 1.31885 | 1.82374 | 0.396449 | 0.124489 | 0. |
| | $75^{th}$ percentile | 3.49156 | 2.02179 | 2.30318 | 0.482926 | 0.129715 | 0.165985 |
| IQRNL Estimate of n Random: | $25^{th}$ percentile | 2.67761 | 2.11337 | 1.63079 | 0.955424 | 0.363638 | 0. |
| | $50^{th}$ percentile | 3.09436 | 2.88785 | 2.61656 | 2.04259 | 2.0039 | 0.0118345 |
| | $75^{th}$ percentile | 3.43874 | 3.5293 | 3.55605 | 2.72319 | 2.5385 | 1.72263 |
| Largest Intra (bp) | | 7. | 4. | 4. | 4. | 4. | 4. |
| Largest Inter (bp) | | 7. | 5. | 5. | 5. | 5. | 5. |
| Mean ΔO | | 328 169. | 72 752.5 | 72 321.3 | 71 955. | 71 826.7 | 71 820. |
| Median ΔO | | 74 840. | 72 970. | 72 380. | 71 830. | 71 820. | 71 820. |
| Mean ΔN | | $1.82784\times10^6$ | $1.02728\times10^6$ | $1.01318\times10^6$ | $1.01302\times10^6$ | $1.01071\times10^6$ | $1.00735\times10^6$ |
| Median ΔN | | $1.0616\times10^6$ | $1.02184\times10^6$ | $1.01485\times10^6$ | $1.01299\times10^6$ | $1.00854\times10^6$ | $1.00735\times10^6$ |

**Figure S3.** Statistical analysis of the rate constants for the most ΔO-fit samples in the O25C dataset. Statistical analyses were performed independently for each sample size (n). A Kolmogorov-Smirnov (KS), Wilcoxon-Singed-Rank (WRS), and Anderson-Darling (AD) test were performed comparing the distribution of the most-fit samples to the distribution of the other samples. For the WSR test, the median of 100 tests involving random pairings is reported. The "Exact IQRNL" value represents the IQRNL calculated directly from the rate-constant distribution without estimation. The "IQRNL Estimate" values represent the $25^{th}$,$50^{th}$, and $75^{th}$ percentiles of 1,000 random resamplings of the distribution. The "IQRNL Estimate of n Random" values represent the median $25^{th}$,$50^{th}$, and $75^{th}$ percentiles of 1,000 random resamplings of 10,000 randomly selected sub-populations of n samples. The largest-intra and largest-inter rows report the size of the largest unnecessary duplexes in base pairs. Values in this table may differ slightly from the values reported in table S1-01 due to the random sampling involved. The values in table S1-01 are the values reported and used in the manuscript text.

Dataset: O25L
Selection Criterion: Smallest Delta-O

| Number of Samples (n) | 51 (All) | 16 | 8 | 4 | 3 | 2 |
|---|---|---|---|---|---|---|
| Histogram | 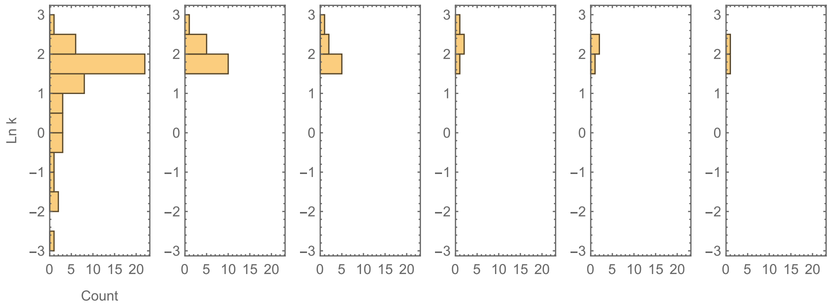 | | | | | |
| p-value (KS vs All) | 1. | $1.00285 \times 10^{-6}$ | 0.000412028 | 0.000560224 | 0.00537815 | 0.0439216 |
| p-value (WSR vs All) | 0.899303 | 0.000706809 | 0.0208626 | 0.100348 | 0.181449 | 0.371093 |
| p-value (AD vs All) | 1. | 0.0000189415 | 0.000188752 | 0.000122915 | 0.00163107 | 0.00725999 |
| Mean ln k | 1.13621 | 1.95186 | 2.00988 | 2.20114 | 2.06507 | 2.06177 |
| Median ln k | 1.64287 | 1.90213 | 1.94743 | 2.10628 | 2.07166 | 2.06177 |
| Exact IQRNL: | 1.08735 | 0.295133 | 0.267396 | 0.347953 | 0.118676 | 0.158234 |
| IQRNL Estimate: 25th percentile | 0.716283 | 0.241106 | 0.188279 | 0.113731 | 0.051921 | 0. |
| IQRNL Estimate: 50th percentile | 1.05344 | 0.278266 | 0.264293 | 0.313339 | 0.0667546 | 0. |
| IQRNL Estimate: 75th percentile | 1.39403 | 0.3148 | 0.418994 | 0.357843 | 0.118676 | 0.158234 |
| IQRNL Estimate of n Random: 25th percentile | 0.743298 | 0.663048 | 0.544981 | 0.390331 | 0.146833 | 0. |
| IQRNL Estimate of n Random: 50th percentile | 1.06179 | 1.05983 | 1.06108 | 1.00205 | 0.790645 | 0. |
| IQRNL Estimate of n Random: 75th percentile | 1.41491 | 1.55488 | 1.74347 | 1.23885 | 1.06118 | 0.657267 |
| Largest Intra (bp) | 7. | 4. | 4. | 4. | 4. | 4. |
| Largest Inter (bp) | 7. | 5. | 5. | 5. | 5. | 5. |
| Mean $\Delta$O | 328169. | 72752.5 | 72321.3 | 71955. | 71826.7 | 71820. |
| Median $\Delta$O | 74840. | 72970. | 72380. | 71830. | 71820. | 71820. |
| Mean $\Delta$N | $1.82784 \times 10^{6}$ | $1.02728 \times 10^{6}$ | $1.01318 \times 10^{6}$ | $1.01302 \times 10^{6}$ | $1.01071 \times 10^{6}$ | $1.00735 \times 10^{6}$ |
| Median $\Delta$N | $1.0616 \times 10^{6}$ | $1.02184 \times 10^{6}$ | $1.01485 \times 10^{6}$ | $1.01299 \times 10^{6}$ | $1.00854 \times 10^{6}$ | $1.00735 \times 10^{6}$ |

**Figure S4.** Statistical analysis of the rate constants for the most ΔO-fit samples in the O25L dataset. Statistical analyses were performed independently for each sample size (n). A Kolmogorov-Smirnov (KS), Wilcoxon-Singed-Rank (WRS), and Anderson-Darling (AD) test were performed comparing the distribution of the most-fit samples to the distribution of the other samples. For the WSR test, the median of 100 tests involving random pairings is reported. The “Exact IQRNL” value represents the IQRNL calculated directly from the rate-constant distribution without estimation. The “IQRNL Estimate” values represent the $25^{th}$,$50^{th}$, and $75^{th}$ percentiles of 1,000 random resamplings of the distribution. The “IQRNL Estimate of n Random” values represent the median $25^{th}$,$50^{th}$, and $75^{th}$ percentiles of 1,000 random resamplings of 10,000 randomly selected sub-populations of n samples. The largest-intra and largest-inter rows report the size of the largest unnecessary duplexes in base pairs. Values in this table may differ slightly from the values reported in table S1-01 due to the random sampling involved. The values in table S1-01 are the values reported and used in the manuscript text.

Dataset: Z37F
Selection Criterion: Smallest Delta-O

| | Number of Samples (n) | 98 (All) | 16 | 8 | 4 | 3 | 2 |
|---|---|---|---|---|---|---|---|
| | Histogram | 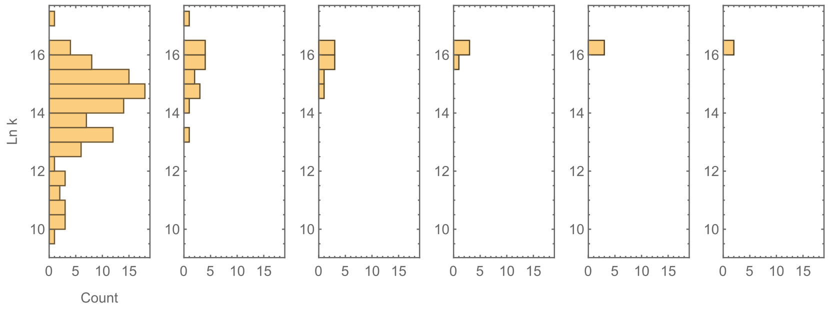 | | | | | |
| | p-value (KS vs All) | 1. | 0.000250954 | 0.0000911934 | 0.0000193783 | 0.000131496 | 0.00420787 |
| | p-value (WSR vs All) | 0.887699 | 0.0029466 | 0.0142662 | 0.100348 | 0.181449 | 0.371093 |
| | p-value (AD vs All) | 1. | 0.0000193208 | 0.0000397618 | 0.0000808994 | 0.000121932 | 0.000413395 |
| | Mean ln k | 14.0481 | 15.4929 | 15.8149 | 16.1772 | 16.2519 | 16.222 |
| | Median ln k | 14.3786 | 15.6869 | 15.9659 | 16.1908 | 16.3116 | 16.222 |
| | Exact IQRNL: | 1.85981 | 1.30606 | 0.76601 | 0.331174 | 0.227968 | 0.303957 |
| IQRNL Estimate: | $25^{th}$ percentile | 1.77075 | 1.02954 | 0.426815 | 0.151979 | 0.0467949 | 0. |
| | $50^{th}$ percentile | 1.85982 | 1.20958 | 0.645228 | 0.237608 | 0.181173 | 0.303957 |
| | $75^{th}$ percentile | 1.97506 | 1.39533 | 0.912619 | 0.299977 | 0.227968 | 0.303957 |
| IQRNL Estimate of n Random: | $25^{th}$ percentile | 1.75268 | 1.42543 | 1.19348 | 0.838772 | 0.363808 | 0. |
| | $50^{th}$ percentile | 1.85981 | 1.79884 | 1.72147 | 1.47369 | 1.23971 | 0.000791321 |
| | $75^{th}$ percentile | 1.99311 | 2.21722 | 2.35428 | 1.92138 | 1.69748 | 1.35889 |
| | Largest Intra (bp) | 8. | 3. | 3. | 3. | 3. | 3. |
| | Largest Inter (bp) | 10. | 8. | 7. | 7. | 7. | 7. |
| | Mean ΔO | $2.52568 \times 10^7$ | 15075. | 10792.5 | 8805. | 8140. | 7250. |
| | Median ΔO | 113510. | 15420. | 10890. | 8850. | 7780. | 7250. |
| | Mean ΔN | $3.30944 \times 10^8$ | $2.16394 \times 10^7$ | $7.52592 \times 10^6$ | $6.4681 \times 10^6$ | $8.4842 \times 10^6$ | $1.26605 \times 10^7$ |
| | Median ΔN | $2.85138 \times 10^6$ | $2.87373 \times 10^6$ | $1.7456 \times 10^6$ | 473610. | 527420. | $1.26605 \times 10^7$ |

**Figure S5.** Statistical analysis of the rate constants for the most ΔO-fit samples in the Z37F dataset. Statistical analyses were performed independently for each sample size (n). A Kolmogorov-Smirnov (KS), Wilcoxon-Singed-Rank (WRS), and Anderson-Darling (AD) test were performed comparing the distribution of the most-fit samples to the distribution of the other samples. For the WSR test, the median of 100 tests involving random pairings is reported. The "Exact IQRNL" value represents the IQRNL calculated directly from the rate-constant distribution without estimation. The "IQRNL Estimate" values represent the $25^{th}$,$50^{th}$, and $75^{th}$ percentiles of 1,000 random resamplings of the distribution. The "IQRNL Estimate of n Random" values represent the median $25^{th}$,$50^{th}$, and $75^{th}$ percentiles of 1,000 random resamplings of 10,000 randomly selected sub-populations of n samples. The largest-intra and largest-inter rows report the size of the largest unnecessary duplexes in base pairs. Values in this table may differ slightly from the values reported in table S1-01 due to the random sampling involved. The values in table S1-01 are the values reported and used in the manuscript text.

Dataset: Z55F
Selection Criterion: Smallest Delta-O

| Number of Samples (n) | 95 (All) | 16 | 8 | 4 | 3 | 2 |
|---|---|---|---|---|---|---|

Histogram

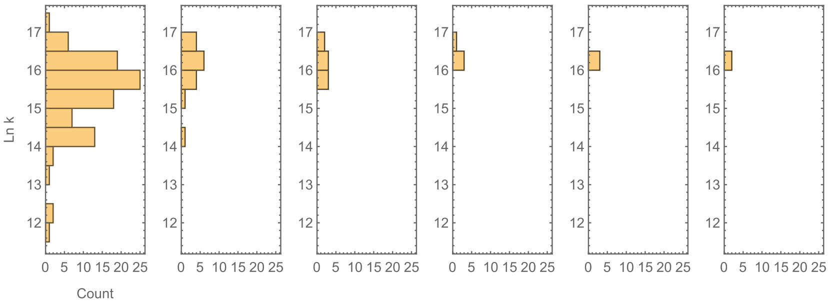


| | | 95 (All) | 16 | 8 | 4 | 3 | 2 |
|---|---|---|---|---|---|---|---|
| p-value (KS vs All) | | 1. | 0.00313738 | 0.0103885 | 0.000857535 | 0.00657443 | 0.0470325 |
| p-value (WSR vs All) | | 0.869703 | 0.0113129 | 0.0587074 | 0.100348 | 0.181449 | 0.371093 |
| p-value (AD vs All) | | 1. | 0.000153599 | 0.000795475 | 0.000366732 | 0.00300428 | 0.0184396 |
| Mean ln k | | 15.379 | 16.1079 | 16.2752 | 16.5163 | 16.4108 | 16.394 |
| Median ln k | | 15.5626 | 16.2681 | 16.394 | 16.4388 | 16.4331 | 16.394 |
| Exact IQRNL: | | 1.29129 | 0.754281 | 0.724609 | 0.244577 | 0.0672625 | 0.0782464 |
| IQRNL Estimate: | $25^{th}$ percentile | 1.0992 | 0.615186 | 0.487689 | 0.0839649 | 0.00857771 | 0. |
| | $50^{th}$ percentile | 1.31654 | 0.76405 | 0.672084 | 0.238859 | 0.0586848 | 0.0391232 |
| | $75^{th}$ percentile | 1.49034 | 0.929406 | 0.769288 | 0.2837 | 0.0672625 | 0.0782464 |
| IQRNL Estimate of n Random: | $25^{th}$ percentile | 1.10269 | 0.906589 | 0.752857 | 0.525651 | 0.223594 | 0. |
| | $50^{th}$ percentile | 1.30867 | 1.18897 | 1.10115 | 0.915275 | 0.783122 | 0. |
| | $75^{th}$ percentile | 1.46747 | 1.47647 | 1.50072 | 1.1969 | 1.0579 | 0.850306 |
| Largest Intra (bp) | | 8. | 3. | 3. | 3. | 3. | 3. |
| Largest Inter (bp) | | 8. | 8. | 7. | 7. | 7. | 7. |
| Mean ΔO | | $2.6026\times10^{7}$ | 15075. | 10792.5 | 8805. | 8140. | 7250. |
| Median ΔO | | 118940. | 15420. | 10890. | 8850. | 7780. | 7250. |
| Mean ΔN | | $7.87067\times10^{7}$ | $2.16394\times10^{7}$ | $7.52592\times10^{6}$ | $6.4681\times10^{6}$ | $8.4842\times10^{6}$ | $1.26605\times10^{7}$ |
| Median ΔN | | $2.80802\times10^{6}$ | $2.87373\times10^{6}$ | $1.7456\times10^{6}$ | 473610. | 527420. | $1.26605\times10^{7}$ |

**Figure S6.** Statistical analysis of the rate constants for the most ΔO-fit samples in the Z55F dataset. Statistical analyses were performed independently for each sample size (n). A Kolmogorov-Smirnov (KS), Wilcoxon-Singed-Rank (WRS), and Anderson-Darling (AD) test were performed comparing the distribution of the most-fit samples to the distribution of the other samples. For the WSR test, the median of 100 tests involving random pairings is reported. The "Exact IQRNL" value represents the IQRNL calculated directly from the rate-constant distribution without estimation. The "IQRNL Estimate" values represent the $25^{th}$,$50^{th}$, and $75^{th}$ percentiles of 1,000 random resamplings of the distribution. The "IQRNL Estimate of n Random" values represent the median $25^{th}$,$50^{th}$, and $75^{th}$ percentiles of 1,000 random resamplings of 10,000 randomly selected sub-populations of n samples. The largest-intra and largest-inter rows report the size of the largest unnecessary duplexes in base pairs. Values in this table may differ slightly from the values reported in table S1-01 due to the random sampling involved. The values in table S1-01 are the values reported and used in the manuscript text.

**Table S2**. **Kinetic dispersions estimated for the 3 most-fit samples in each dataset.** Reported values include the number of samples in the population (n) and kinetic dispersion (IQRNL). The three values reported for IQRNL represent the 25th, 50th, and 75th percentiles of the estimate.

| **Dataset** | **Units of k** | **Population** | **n** | **Estimated IQRNL (25th, 50th, 75th percentiles)** | | | **Median ΔN** | **Median ΔO** |
|---|---|---|---|---|---|---|---|---|
| H22F | $M^{-1}s^{-1}$ | Random | 3 | 0.89 | 1.5 | 2.0 | $6.3 \times 10^4$ | $5.0 \times 10^3$ |
| | | N-Fit | 3 | 0.09 | 0.95 | 1.1 | $1.3 \times 10^4$ | $2.6 \times 10^3$ |
| | | O-Fit | 3 | 0.091 | 0.24 | 0.33 | $6.5 \times 10^4$ | $1.7 \times 10^3$ |
| O25C | $M^{-2}s^{-1}$ | Random | 3 | 0.85 | 2.1 | 3.1 | $1.1 \times 10^6$ | $7.8 \times 10^4$ |
| | | N-Fit | 3 | 1.2 | 1.7 | 2.9 | $1.0 \times 10^6$ | $7.4 \times 10^4$ |
| | | O-Fit | 3 | 0.0052 | 0.12 | 0.13 | $1.0 \times 10^6$ | $7.1 \times 10^4$ |
| O25L | $M^{-1}s^{-1}$ | Random | 3 | 0.33 | 0.80 | 1.5 | $1.1 \times 10^6$ | $7.8 \times 10^4$ |
| | | N-Fit | 3 | 0.17 | 0.39 | 0.56 | $1.0 \times 10^6$ | $7.4 \times 10^4$ |
| | | O-Fit | 3 | 0.052 | 0.067 | 0.12 | $1.0 \times 10^6$ | $7.1 \times 10^4$ |
| Z37F | $M^{-1}s^{-1}$ | Random | 3 | 0.75 | 1.3 | 1.9 | $2.9 \times 10^6$ | $1.1 \times 10^5$ |
| | | N-Fit | 3 | 0.025 | 1.0 | 1.1 | $1.3 x10^5$ | $1.7 \times 10^4$ |
| | | O-Fit | 3 | 0.047 | 0.18 | 0.23 | $5.3 \times 10^5$ | $7.8 \times 10^3$ |
| Z55F | $M^{-1}s^{-1}$ | Random | 3 | 0.48 | 0.80 | 1.2 | $2.8 \times 10^6$ | $1.2 \times 10^5$ |
| | | N-Fit | 3 | 0.27 | 0.49 | 0.76 | $1.3 x10^5$ | $1.7 \times 10^4$ |
| | | O-Fit | 3 | 0.0086 | 0.059 | 0.067 | $5.3 \times 10^5$ | $7.8 \times 10^3$ |

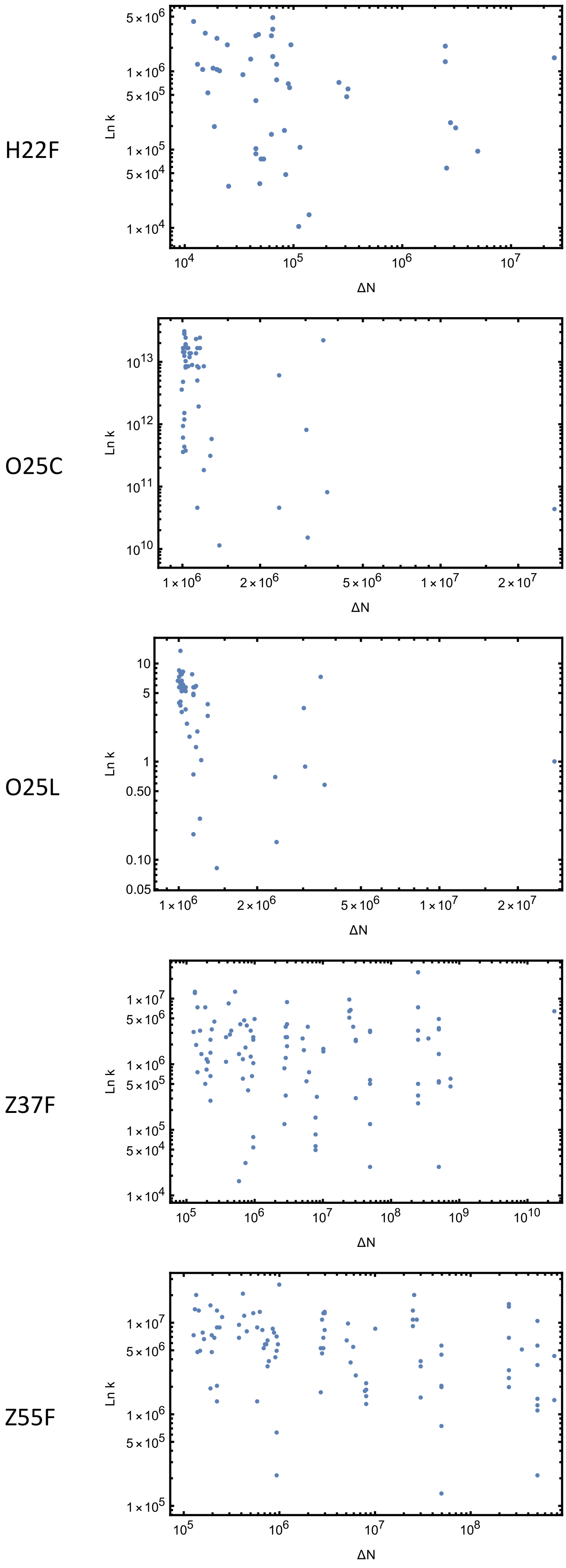


**Figure S7.** Correlation plots of the rate constants in each dataset as a function of ΔN.

Dataset: H22F
Selection Criterion: Smallest Delta-N

| Number of Samples (n) | 47 (All) | 16 | 8 | 4 | 3 | 2 |
|---|---|---|---|---|---|---|
| Histogram | 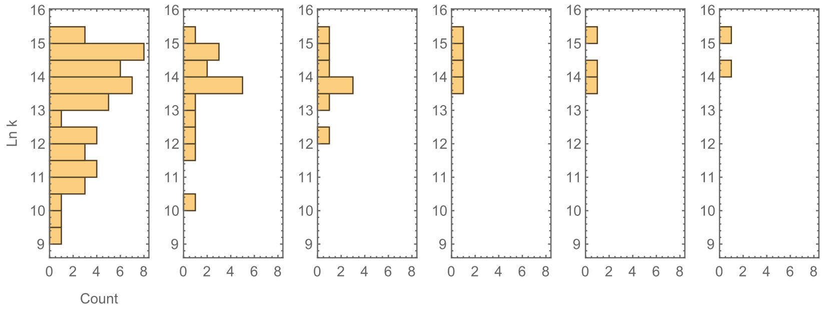 | | | | | |
| p-value (KS vs All) | 1. | 0.150183 | 0.199946 | 0.0712808 | 0.160592 | 0.251619 |
| p-value (WSR vs All) | 0.912264 | 0.244648 | 0.293622 | 0.201243 | 0.422678 | 0.371093 |
| p-value (AD vs All) | 1. | 0.205087 | 0.118408 | 0.0261269 | 0.113646 | 0.0598221 |
| Mean ln k | 13.0824 | 13.5808 | 13.9014 | 14.5229 | 14.3815 | 14.6401 |
| Median ln k | 13.5008 | 13.869 | 13.8831 | 14.4765 | 14.0061 | 14.6401 |
| Exact IQRNL: | 2.56249 | 1.32141 | 0.960703 | 1.1753 | 1.05737 | 1.26799 |
| IQRNL Estimate: 25th percentile | 2.23891 | 0.920931 | 0.706443 | 0.612221 | 0.106373 | 0. |
| IQRNL Estimate: 50th percentile | 2.57574 | 1.24003 | 1.07787 | 0.704912 | 0.950996 | 0. |
| IQRNL Estimate: 75th percentile | 2.82051 | 1.71822 | 1.45144 | 1.10439 | 1.05737 | 1.26799 |
| IQRNL Estimate of n Random: 25th percentile | 2.20098 | 1.82753 | 1.43917 | 0.946975 | 0.370243 | 0. |
| IQRNL Estimate of n Random: 50th percentile | 2.51625 | 2.34015 | 2.0896 | 1.66677 | 1.4375 | 0.0303629 |
| IQRNL Estimate of n Random: 75th percentile | 2.74338 | 2.7771 | 2.70109 | 2.19278 | 1.99445 | 1.54766 |
| Largest Intra (bp) | 5. | 3. | 3. | 2. | 2. | 2. |
| Largest Inter (bp) | 7. | 4. | 3. | 3. | 3. | 3. |
| Mean ΔO | 11 810.2 | 4065. | 3085. | 2465. | 2720. | 2770. |
| Median ΔO | 5000. | 3890. | 2720. | 2290. | 2620. | 2770. |
| Mean ΔN | 983 659. | 23 997.5 | 16 015. | 13 745. | 13 220. | 12 490. |
| Median ΔN | 63 100. | 19 770. | 15 920. | 13 840. | 13 000. | 12 490. |

**Figure S8.** Statistical analysis of the rate constants for the most ΔN-fit samples in the H22F dataset. Statistical analyses were performed independently for each sample size (n). A Kolmogorov-Smirnov (KS), Wilcoxon-Singed-Rank (WRS), and Anderson-Darling (AD) test were performed comparing the distribution of the most-fit samples to the distribution of the other samples. For the WSR test, the median of 100 tests involving random pairings is reported. The "Exact IQRNL" value represents the IQRNL calculated directly from the rate-constant distribution without estimation. The "IQRNL Estimate" values represent the $25^{th}$,$50^{th}$, and $75^{th}$ percentiles of 1,000 random resamplings of the distribution. The "IQRNL Estimate of n Random" values represent the median $25^{th}$,$50^{th}$, and $75^{th}$ percentiles of 1,000 random resamplings of 10,000 randomly selected sub-populations of n samples. The largest-intra and largest-inter rows report the size of the largest unnecessary duplexes in base pairs. Values in this table may differ slightly from the values reported in table S1-01 due to the random sampling involved. The values in table S1-01 are the values reported and used in the manuscript text.

Dataset: O25C
Selection Criterion: Smallest Delta-N

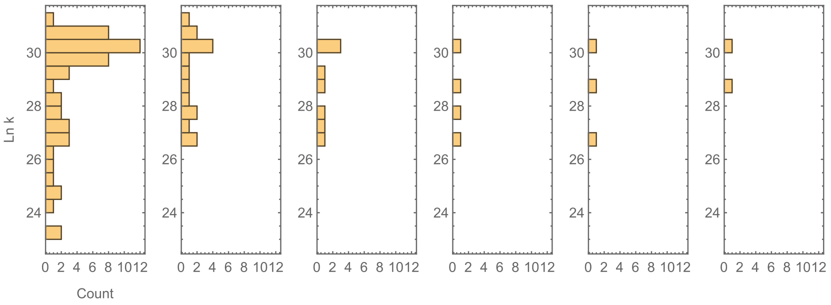


| Number of Samples (n) | | 51 (All) | 16 | 8 | 4 | 3 | 2 |
|---|---|---|---|---|---|---|---|
| Histogram | | | | | | | |
| p-value (KS vs All) | | 1. | 0.511221 | 0.589674 | 0.456323 | 0.894502 | 0.905098 |
| p-value (WSR vs All) | | 0.885635 | 0.518046 | 0.624065 | 0.583882 | 0.789268 | 1. |
| p-value (AD vs All) | | 1. | 0.279465 | 0.484824 | 0.567498 | 0.904607 | 0.811722 |
| Mean ln k | | 28.7737 | 29.1357 | 28.7938 | 28.3861 | 28.6599 | 29.6783 |
| Median ln k | | 29.7781 | 29.5007 | 29.0491 | 28.2286 | 28.8923 | 29.6783 |
| Exact IQRNL: | | 3.24702 | 2.68387 | 2.88068 | 2.58425 | 2.8808 | 1.57192 |
| IQRNL Estimate: | $25^{th}$ percentile | 2.7264 | 2.35492 | 1.88372 | 1.32744 | 1.17894 | 0. |
| | $50^{th}$ percentile | 3.16152 | 2.70502 | 2.33449 | 1.92054 | 1.70187 | 1.57192 |
| | $75^{th}$ percentile | 3.48572 | 3.07228 | 2.89935 | 2.58425 | 2.8808 | 1.57192 |
| IQRNL Estimate of n Random: | $25^{th}$ percentile | 2.67348 | 2.11583 | 1.64435 | 0.967789 | 0.36286 | 0. |
| | $50^{th}$ percentile | 3.09739 | 2.89935 | 2.63436 | 2.06912 | 1.99269 | 0. |
| | $75^{th}$ percentile | 3.44007 | 3.52581 | 3.57729 | 2.74844 | 2.51942 | 1.72613 |
| Largest Intra (bp) | | 7. | 4. | 4. | 4. | 4. | 4. |
| Largest Inter (bp) | | 7. | 5. | 5. | 5. | 5. | 5. |
| Mean ΔO | | 328169. | 73275. | 73632.5 | 73525. | 73300. | 72170. |
| Median ΔO | | 74840. | 72990. | 73560. | 73360. | 72520. | 72170. |
| Mean ΔN | | $1.82784 \times 10^6$ | $1.01412 \times 10^6$ | $1.00734 \times 10^6$ | $1.00467 \times 10^6$ | $1.00354 \times 10^6$ | $1.00166 \times 10^6$ |
| Median ΔN | | $1.0616 \times 10^6$ | $1.01317 \times 10^6$ | $1.00831 \times 10^6$ | $1.00673 \times 10^6$ | $1.00616 \times 10^6$ | $1.00166 \times 10^6$ |

**Figure S9.** Statistical analysis of the rate constants for the most ΔN-fit samples in the O25C dataset. Statistical analyses were performed independently for each sample size (n). A Kolmogorov-Smirnov (KS), Wilcoxon-Singed-Rank (WRS), and Anderson-Darling (AD) test were performed comparing the distribution of the most-fit samples to the distribution of the other samples. For the WSR test, the median of 100 tests involving random pairings is reported. The “Exact IQRNL” value represents the IQRNL calculated directly from the rate-constant distribution without estimation. The “IQRNL Estimate” values represent the $25^{th}$,$50^{th}$, and $75^{th}$ percentiles of 1,000 random resamplings of the distribution. The “IQRNL Estimate of n Random” values represent the median $25^{th}$,$50^{th}$, and $75^{th}$ percentiles of 1,000 random resamplings of 10,000 randomly selected sub-populations of n samples. The largest-intra and largest-inter rows report the size of the largest unnecessary duplexes in base pairs. Values in this table may differ slightly from the values reported in table S1-01 due to the random sampling involved. The values in table S1-01 are the values reported and used in the manuscript text.

Dataset: O25L
Selection Criterion: Smallest Delta-N

| Number of Samples (n) | | 51 (All) | 16 | 8 | 4 | 3 | 2 |
|---|---|---|---|---|---|---|---|
| Histogram | | 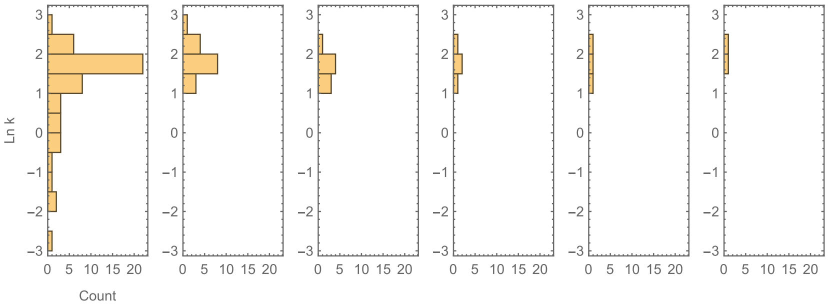 | | | | | |
| p-value (KS vs All) | | 1. | 0.002527 | 0.137046 | 0.416727 | 0.384586 | 0.0705882 |
| p-value (WSR vs All) | | 0.884488 | 0.00410684 | 0.233953 | 0.583882 | 0.422678 | 0.371093 |
| p-value (AD vs All) | | 1. | 0.000193265 | 0.141065 | 0.186394 | 0.126293 | 0.0128354 |
| Mean ln k | | 1.13621 | 1.85065 | 1.71802 | 1.79769 | 1.81554 | 2.02655 |
| Median ln k | | 1.64287 | 1.83888 | 1.79036 | 1.82817 | 1.91221 | 2.02655 |
| Exact IQRNL: | | 1.08735 | 0.348163 | 0.541775 | 0.457717 | 0.560528 | 0.228684 |
| IQRNL Estimate: | $25^{th}$ percentile | 0.766635 | 0.298235 | 0.366462 | 0.259344 | 0.171513 | 0. |
| | $50^{th}$ percentile | 1.08735 | 0.345143 | 0.469881 | 0.373686 | 0.389016 | 0.228684 |
| | $75^{th}$ percentile | 1.43382 | 0.471713 | 0.553912 | 0.457717 | 0.560528 | 0.228684 |
| IQRNL Estimate of n Random: | $25^{th}$ percentile | 0.736032 | 0.653103 | 0.550156 | 0.38981 | 0.151705 | 0. |
| | $50^{th}$ percentile | 1.05938 | 1.05194 | 1.05963 | 1.01213 | 0.853829 | 0. |
| | $75^{th}$ percentile | 1.40508 | 1.53894 | 1.74288 | 1.25099 | 1.1208 | 0.67207 |
| Largest Intra (bp) | | 7. | 4. | 4. | 4. | 4. | 4. |
| Largest Inter (bp) | | 7. | 5. | 5. | 5. | 5. | 5. |
| Mean △O | | 328 169. | 73 275. | 73 632.5 | 73 525. | 73 300. | 72 170. |
| Median △O | | 74 840. | 72 990. | 73 560. | 73 360. | 72 520. | 72 170. |
| Mean △N | | $1.82784 \times 10^6$ | $1.01412 \times 10^6$ | $1.00734 \times 10^6$ | $1.00467 \times 10^6$ | $1.00354 \times 10^6$ | $1.00166 \times 10^6$ |
| Median △N | | $1.0616 \times 10^6$ | $1.01317 \times 10^6$ | $1.00831 \times 10^6$ | $1.00673 \times 10^6$ | $1.00616 \times 10^6$ | $1.00166 \times 10^6$ |

**Figure S10.** Statistical analysis of the rate constants for the most ΔN-fit samples in the O25L dataset. Statistical analyses were performed independently for each sample size (n). A Kolmogorov-Smirnov (KS), Wilcoxon-Singed-Rank (WRS), and Anderson-Darling (AD) test were performed comparing the distribution of the most-fit samples to the distribution of the other samples. For the WSR test, the median of 100 tests involving random pairings is reported. The “Exact IQRNL” value represents the IQRNL calculated directly from the rate-constant distribution without estimation. The “IQRNL Estimate” values represent the $25^{th}$,$50^{th}$, and $75^{th}$ percentiles of 1,000 random resamplings of the distribution. The “IQRNL Estimate of n Random” values represent the median $25^{th}$,$50^{th}$, and $75^{th}$ percentiles of 1,000 random resamplings of 10,000 randomly selected sub-populations of n samples. The largest-intra and largest-inter rows report the size of the largest unnecessary duplexes in base pairs. Values in this table may differ slightly from the values reported in table S1-01 due to the random sampling involved. The values in table S1-01 are the values reported and used in the manuscript text.

Dataset: Z37F
Selection Criterion: Smallest Delta-N

| Number of Samples (n) | | 98 (All) | 16 | 8 | 4 | 3 | 2 |
|---|---|---|---|---|---|---|---|
| Histogram | | 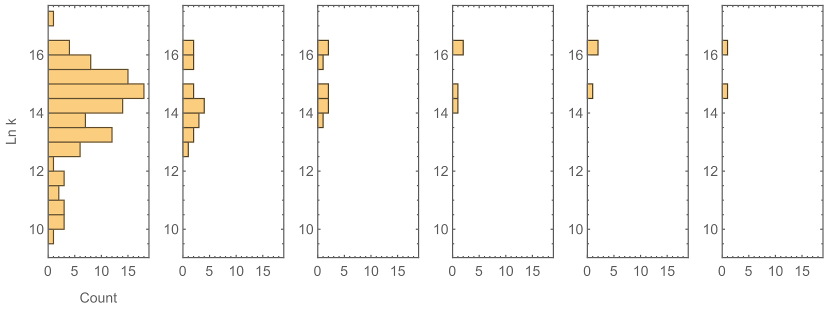 | | | | | |
| p-value (KS vs All) | | 1. | 0.559294 | 0.246396 | 0.14224 | 0.0786871 | 0.236061 |
| p-value (WSR vs All) | | 0.890083 | 0.408201 | 0.141482 | 0.201243 | 0.181449 | 0.371093 |
| p-value (AD vs All) | | 1. | 0.337305 | 0.0424171 | 0.0174769 | 0.00348038 | 0.0642988 |
| Mean ln k | | 14.0481 | 14.4508 | 15.0751 | 15.517 | 15.8606 | 15.6182 |
| Median ln k | | 14.3786 | 14.2152 | 14.9535 | 15.6182 | 16.3116 | 15.6182 |
| Exact IQRNL: | | 1.85981 | 1.81576 | 1.7314 | 1.62298 | 1.0653 | 1.38667 |
| IQRNL Estimate: | $25^{th}$ percentile | 1.76162 | 1.26613 | 1.12588 | 0.727059 | 0.025291 | 0. |
| | $50^{th}$ percentile | 1.85869 | 1.68331 | 1.51195 | 0.946506 | 1.04001 | 1.38667 |
| | $75^{th}$ percentile | 1.9863 | 2.1037 | 1.84243 | 1.60612 | 1.0653 | 1.38667 |
| IQRNL Estimate of n Random: | $25^{th}$ percentile | 1.7521 | 1.42031 | 1.18635 | 0.831597 | 0.361205 | 0. |
| | $50^{th}$ percentile | 1.85981 | 1.80302 | 1.71559 | 1.46014 | 1.24919 | 0.0119458 |
| | $75^{th}$ percentile | 1.98746 | 2.22367 | 2.36047 | 1.89471 | 1.70733 | 1.34297 |
| Largest Intra (bp) | | 8. | 4. | 4. | 3. | 3. | 3. |
| Largest Inter (bp) | | 10. | 4. | 4. | 4. | 4. | 4. |
| Mean ΔO | | $2.52568 \times 10^7$ | 38 441.3 | 28 567.5 | 18 675. | 17 513.3 | 17 720. |
| Median ΔO | | 113 510. | 41 530. | 23 840. | 19 630. | 17 100. | 17 720. |
| Mean ΔN | | $3.30944 \times 10^8$ | 174 391. | 142 838. | 133 070. | 130 640. | 128 690. |
| Median ΔN | | $2.85138 \times 10^6$ | 175 800. | 142 350. | 133 080. | 131 620. | 128 690. |

**Figure S11.** Statistical analysis of the rate constants for the most ΔN-fit samples in the Z37F dataset. Statistical analyses were performed independently for each sample size (n). A Kolmogorov-Smirnov (KS), Wilcoxon-Singed-Rank (WRS), and Anderson-Darling (AD) test were performed comparing the distribution of the most-fit samples to the distribution of the other samples. For the WSR test, the median of 100 tests involving random pairings is reported. The "Exact IQRNL" value represents the IQRNL calculated directly from the rate-constant distribution without estimation. The "IQRNL Estimate" values represent the $25^{th}$,$50^{th}$, and $75^{th}$ percentiles of 1,000 random resamplings of the distribution. The "IQRNL Estimate of n Random" values represent the median $25^{th}$,$50^{th}$, and $75^{th}$ percentiles of 1,000 random resamplings of 10,000 randomly selected sub-populations of n samples. The largest-intra and largest-inter rows report the size of the largest unnecessary duplexes in base pairs. Values in this table may differ slightly from the values reported in table S1-01 due to the random sampling involved. The values in table S1-01 are the values reported and used in the manuscript text.

Dataset: Z55F
Selection Criterion: Smallest Delta-N

| | | | | | | | |
|---|---|---|---|---|---|---|---|
| | Number of Samples (n) | 95 (All) | 16 | 8 | 4 | 3 | 2 |
| | Histogram | 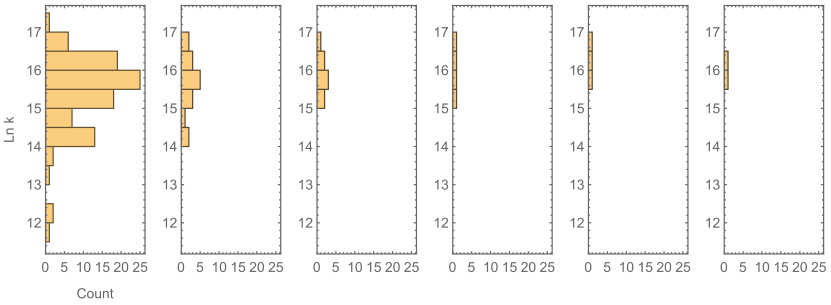 | | | | | |
| | p-value (KS vs All) | 1. | 0.370631 | 0.105391 | 0.357149 | 0.120652 | 0.29832 |
| | p-value (WSR vs All) | 0.884898 | 0.289131 | 0.208692 | 0.201243 | 0.181449 | 0.371093 |
| | p-value (AD vs All) | 1. | 0.242231 | 0.0732145 | 0.0899777 | 0.0167897 | 0.153024 |
| | Mean ln k | 15.379 | 15.6847 | 15.9853 | 16.11 | 16.3481 | 16.1185 |
| | Median ln k | 15.5626 | 15.7666 | 15.8272 | 16.1185 | 16.4445 | 16.1185 |
| | Exact IQRNL: | 1.29129 | 1.0298 | 0.861274 | 1.03162 | 0.760926 | 0.652 |
| IQRNL Estimate: | $25^{th}$ percentile | 1.11613 | 0.75113 | 0.577612 | 0.524339 | 0.271926 | 0. |
| | $50^{th}$ percentile | 1.31921 | 1.01986 | 0.722282 | 0.722678 | 0.489 | 0. |
| | $75^{th}$ percentile | 1.48079 | 1.25866 | 0.88548 | 1.01457 | 0.760926 | 0.652 |
| IQRNL Estimate of n Random: | $25^{th}$ percentile | 1.10167 | 0.904448 | 0.768734 | 0.52401 | 0.223984 | 0. |
| | $50^{th}$ percentile | 1.30512 | 1.19357 | 1.11879 | 0.918802 | 0.785417 | 0.0202495 |
| | $75^{th}$ percentile | 1.46718 | 1.48563 | 1.5121 | 1.1939 | 1.07096 | 0.852633 |
| | Largest Intra (bp) | 8. | 4. | 4. | 3. | 3. | 3. |
| | Largest Inter (bp) | 8. | 4. | 4. | 4. | 4. | 4. |
| | Mean ΔO | $2.6026 \times 10^7$ | 38 441.3 | 28 567.5 | 18 675. | 17 513.3 | 17 720. |
| | Median ΔO | 118 940. | 41 530. | 23 840. | 19 630. | 17 100. | 17 720. |
| | Mean ΔN | $7.87067 \times 10^7$ | 174 391. | 142 838. | 133 070. | 130 640. | 128 690. |
| | Median ΔN | $2.80802 \times 10^6$ | 175 800. | 142 350. | 133 080. | 131 620. | 128 690. |

**Figure S12.** Statistical analysis of the rate constants for the most ΔN-fit samples in the Z55F dataset. Statistical analyses were performed independently for each sample size (n). A Kolmogorov-Smirnov (KS), Wilcoxon-Singed-Rank (WRS), and Anderson-Darling (AD) test were performed comparing the distribution of the most-fit samples to the distribution of the other samples. For the WSR test, the median of 100 tests involving random pairings is reported. The "Exact IQRNL" value represents the IQRNL calculated directly from the rate-constant distribution without estimation. The "IQRNL Estimate" values represent the $25^{th}$,$50^{th}$, and $75^{th}$ percentiles of 1,000 random resamplings of the distribution. The "IQRNL Estimate of n Random" values represent the median $25^{th}$,$50^{th}$, and $75^{th}$ percentiles of 1,000 random resamplings of 10,000 randomly selected sub-populations of n samples. The largest-intra and largest-inter rows report the size of the largest unnecessary duplexes in base pairs. Values in this table may differ slightly from the values reported in table S1-01 due to the random sampling involved. The values in table S1-01 are the values reported and used in the manuscript text.

## Supplementary Note 2: Generation of New Oligomers (*SeqEvo Program*)

Oligomers were generated using the custom written "Sequence Evolver" (abbreviated SeqEvo) computer program.

This program starts by reading four input files. The first file (called the fixed domains file) contains a list of binding domains who's base-sequences will not be modified during optimization. Each of these domains has a name and a base-sequence. The second file (called the variable domains file) contains a list of binding domains who's base-sequences can be modified during optimization. The format of this file is the same as the fixed domains file. The third file (called the oligomers file) contains a list of oligomers. Each oligomer has a name followed by one or more binding domains. Oligomers can also contain binding domain complements, which are specified by "c." and then a domain name. The fourth file is a list of runtime parameters for the program. This includes the following parameters: 1) The value for x in the $W_x$ calculations 2) the thresholds for consecutive bases 3) NL, 4) CPL, 5) NMPC, 6) GPC, and 7) NDPM.

Based on these input files, the program conducts an evolution-inspired search for fit oligomers by executing a series of staged mutation-selection cycles. Mutation events include: 1) a random shuffling of a variable domain's sequence, 2) a relocation of a sub-sequence within a variable domain, and 3) the swapping of two bases within a variable domain. Selection events are based on the $W_x$ fitness of the oligomers. The algorithm conducts a pre-determined number of mutation-selection events, and then returns the fittest set of oligomers encountered. New oligomers produced by SeqEvo are subject to several constraints. First, the base-sequence in each "fixed" binding domain is unchanged. Second, the base-sequence in each "variable" binding domain may vary, but the total number of Adenine (A), Cytosine (C), Guanine (G), and Thymine (T) in the domain is conserved. Third, no oligonucleotides in the set may contain stretches of consecutive A's, C's, G's or T's longer than thresholds declared at runtime. For oligomers generated in this manuscript, thresholds of 6, 3, 3, and 6 were used for the bases A, C, G, T, respectively;

### Heuristic algorithm

The evolution-inspired heuristic algorithm is as follows and is depicted visually in Figure S2-01 below.

***Algorithm Start.*** An initial network and network design are provided to the algorithm. Networks termed lineage mothers are created which include a clone of the original network and *Number-of-Lineages* **(NL)** minus one additional clones which have been altered by *shuffle* mutations (below). Loop one (below) is iterated *Cycles-Per-Lineage* **(CPL)** times on each of the lineage mothers. After each iteration of loop one, if the returned network is more fit than the lineage mother, it replaces that lineage mother. After the last iteration of loop one, the fittest lineage mother is declared the victor and returned to the user.

***Loop One*****.** This loop is provided an initial network termed the lineage-mother. Networks termed cycle-mothers are created, including a clone of the lineage mother and *New-Mothers-Per-Cycle*

**(NMPC)** additional clones, which have been altered by *cut-and-paste* mutations (below). Loop two (below) is iterated *Generations-Per-Cycle* **(GPC)** times on each cycle-mother. After each iteration of loop two, if the returned network is more fit than the cycle mother, it replaces that cycle mother. After the final iteration of loop two, the current cycle-mother is returned.

***Loop Two.*** This loop is performed on an initial network termed the cycle-mother. Networks termed cycle-daughters are created, including a clone of the selected design and *New-Daughters-Per-Mother* **(NDPM)** additional clones which have been altered by *swap* mutations (below). The fittest cycle-daughter is returned.

***Shuffle Mutation**.* The sequence of each variable domain is shuffled using a pseudo-random number generator. This leads to a new design where all variable domains have been changed.

***Cut-and-paste Mutation.*** A variable domain is selected. Two bases are selected at random from the domain. The sequence of bases between and including these bases is removed from the domain. A position within or at the ends of the remaining sequence is selected at random. The removed sequence is reintroduced to the domain at the chosen position.

***Swap Mutation.*** A variable domain is selected. From this domain, two bases are chosen at random. The identity of these bases is exchanged.

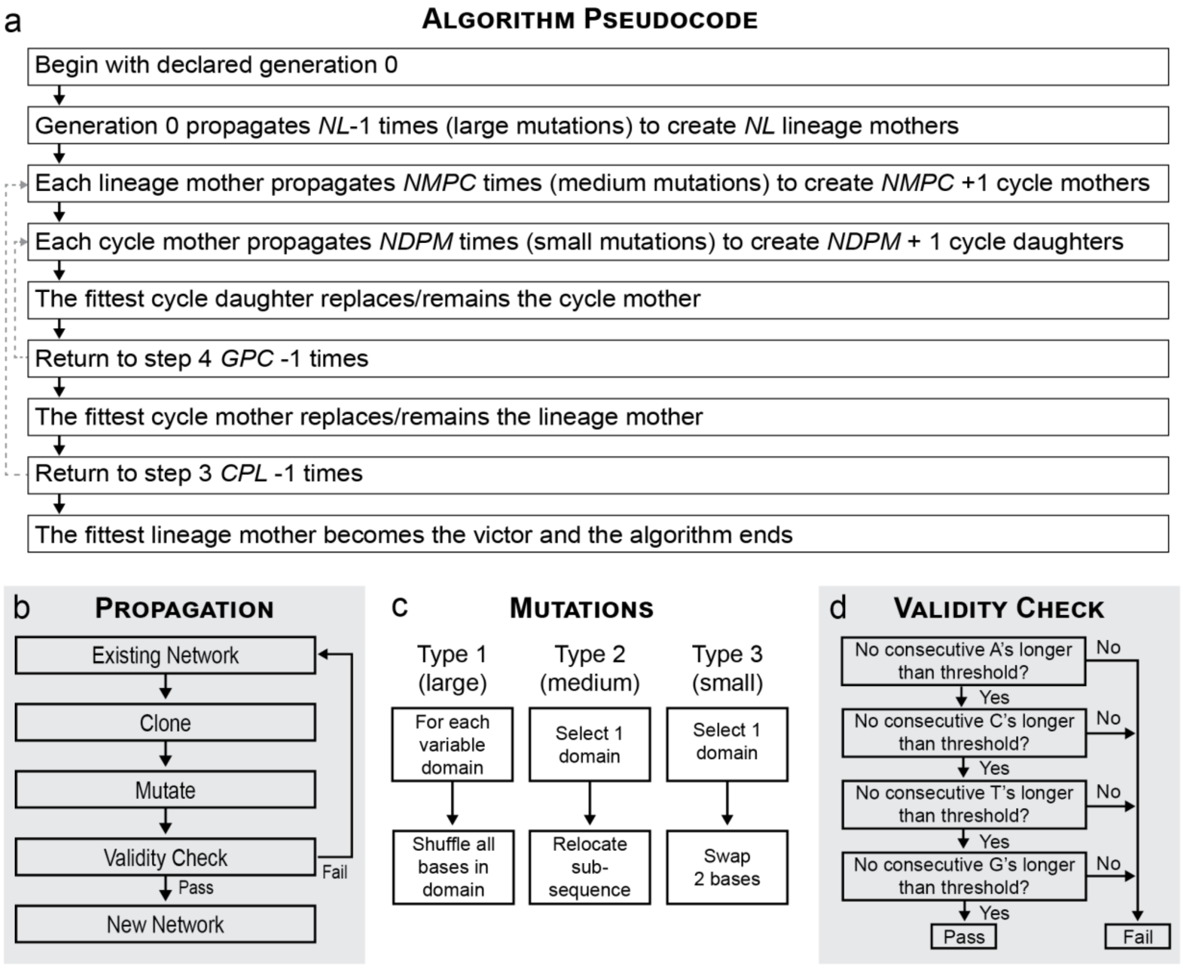


**Figure S13**. Architecture of the sequence generation algorithm. **(a)** A visual depiction of the process followed by the algorithm. Key parameters, which control the frequency and duration of branching events, are denoted in italics. **(b)** During algorithm execution, networks propagate via a clone-then-mutate process, which utilizes one of three types of mutation **(c)** and a validity check **(d)**. The architecture is designed to modularly interface with fitness functions necessary in the fifth, seventh and ninth steps in (a).

**Algorithm Tuning and Optimization**

It was observed that algorithm efficiency varies substantially depending on the value of key algorithm parameters. Consequently, a search for optimal algorithm parameters was conducted. SeqEvo was challenged to identify a pair of oligomers which form a single 34 base-pair duplex with no unnecessary inter-oligomer duplexes larger than 2 base-pairs (referred to below as the “design problem”). Using a trial-and-error approach, initial algorithm parameters were identified which solved the design problem (Parameter set #0 in Table S2-01). The efficiency of a given set of algorithm parameters was defined to be the inverse of the total number of oligo-sets scored prior to identifying a valid solution to the design problem. Since SeqEvo performance is limited by the time necessary to score each network, this quantity is a reasonable representation of the total computation required by the program.

*Global Search.* Building on the knowledge that parameter set #0 could reliably solve the design problem after considering ~256,000 networks, the search for efficient parameter sets was narrowed to parameters-sets which considered up to ~2,560,000 oligo-sets. Starting with the minimal parameters of CPL=1, GPC=1, NDPM=1, NL=8, and NMPC=1, parameter-sets representing equal investment of 2,560,000 networks worth of resources into 1, 2, 3, 4 or all 5 parameters were generated (Parameter Sets #1-31 in Table S2-01). The resulting 31 parameter sets are expected to sample parameter space in a course-grained fashion. The efficiency of each parameter set was determined in 81 independent trials. Of the 31 parameter sets, 24 reliably identified a solution to the problem (> 80% success). A statistical summary of the observed efficiencies is reported in Figure S2-02b, where data from the 7 ineffective parameters is omitted (*). For effective parameter sets, median efficiencies were observed to vary more than 2 orders of magnitude. The most efficient parameter set was observed to be #5 (CPL = 80000, GPC = 1, NDPM = 1, NL = 8, NMPC = 1), which has been marked in orange in Figure S2-02. A typical instance of this algorithm considered ~14,000 networks before arriving at a solution, whereas the next most efficient parameter set (#27 in Table S2-01) typically required ~43,000 considerations.

*Local Search.* The area surrounding parameter set #5 was further explored to confirm a local maximum in efficiency. Parameter set #5 resides on the boundary of parameter space, with a CPL value of 80,000 and all other parameters at their minimal value. Each of the five key parameters was systematically increased while holding all the other parameters fixed. The ranges over which the parameters were varied are specified in Figure S2-02b. The efficiency of these additional 21 algorithms were determined using a similar 81 trials-per-algorithm approach. A statistically significant decrease in efficiency was observed immediately for the NL, NDPM, and GPC parameters. Efficiency appears to be relatively stable for NMPC values up to 32, above which a decrease in efficiency was resolvable. No variation in efficiency was observed for the CPL parameter, which was anticipated since CPL controls algorithm duration, but has minimal effect on the structure of the evolutionary search. These results suggest that the region of high efficiency encompasses parameter sets with the following parameter values; NL of 8, NDPM of 1, GPC of 1, NMPC between (and including) 1 to 32, and no observed limitation on CPL. It is suggested that parameters of NL = 8, NDPM =1, GPC =1,

and NMPC = 2 be used as default values, and that CPL be tuned depending on the algorithm runtime/device quality desired.

**Table S3.** 31 parameter sets used for during the “Global” sampling of algorithm efficiencies. Initially, a parameter set capable of solving the design problem was identified in a trial-and-error fashion (parameter-set 0). Based on this result, 31 sets of parameters were identified which span the finite region of parameter space inhabited by parameter sets consuming approximately 10x the resources of parameter set 0 (parameter-sets 1-31).

| | Parameter Values | | | | |
|---|---|---|---|---|---|
| **Parameter-Set** | **CPL** | **GPC** | **NDPM** | **NL** | **NMPC** |
| **0** | 1000 | 1 | 1 | 64 | 1 |
| **1** | 1 | 1 | 1 | 8 | 160000 |
| **2** | 1 | 1 | 1 | 512000 | 1 |
| **3** | 1 | 1 | 160000 | 8 | 1 |
| **4** | 1 | 160000 | 1 | 8 | 1 |
| **5** | 80000 | 1 | 1 | 8 | 1 |
| **6** | 1 | 1 | 1 | 1131 | 1131 |
| **7** | 1 | 1 | 565 | 8 | 565 |
| **8** | 1 | 565 | 1 | 8 | 565 |
| **9** | 400 | 1 | 1 | 8 | 400 |
| **10** | 1 | 1 | 1131 | 1131 | 1 |
| **11** | 1 | 1131 | 1 | 1131 | 1 |
| **12** | 800 | 1 | 1 | 800 | 1 |
| **13** | 1 | 400 | 400 | 8 | 1 |
| **14** | 400 | 1 | 400 | 8 | 1 |
| **15** | 400 | 400 | 1 | 8 | 1 |
| **16** | 1 | 1 | 137 | 137 | 137 |
| **17** | 1 | 137 | 1 | 137 | 137 |
| **18** | 109 | 1 | 1 | 109 | 109 |
| **19** | 1 | 69 | 69 | 8 | 69 |
| **20** | 68 | 1 | 68 | 8 | 68 |
| **21** | 68 | 68 | 1 | 8 | 68 |
| **22** | 1 | 109 | 109 | 109 | 1 |
| **23** | 109 | 1 | 109 | 109 | 1 |
| **24** | 109 | 109 | 1 | 109 | 1 |
| **25** | 55 | 55 | 55 | 8 | 1 |
| **26** | 1 | 40 | 40 | 40 | 40 |
| **27** | 40 | 1 | 40 | 40 | 40 |
| **28** | 40 | 40 | 1 | 40 | 40 |
| **29** | 24 | 24 | 24 | 8 | 24 |
| **30** | 34 | 34 | 34 | 34 | 1 |
| **31** | 19 | 19 | 19 | 19 | 19 |

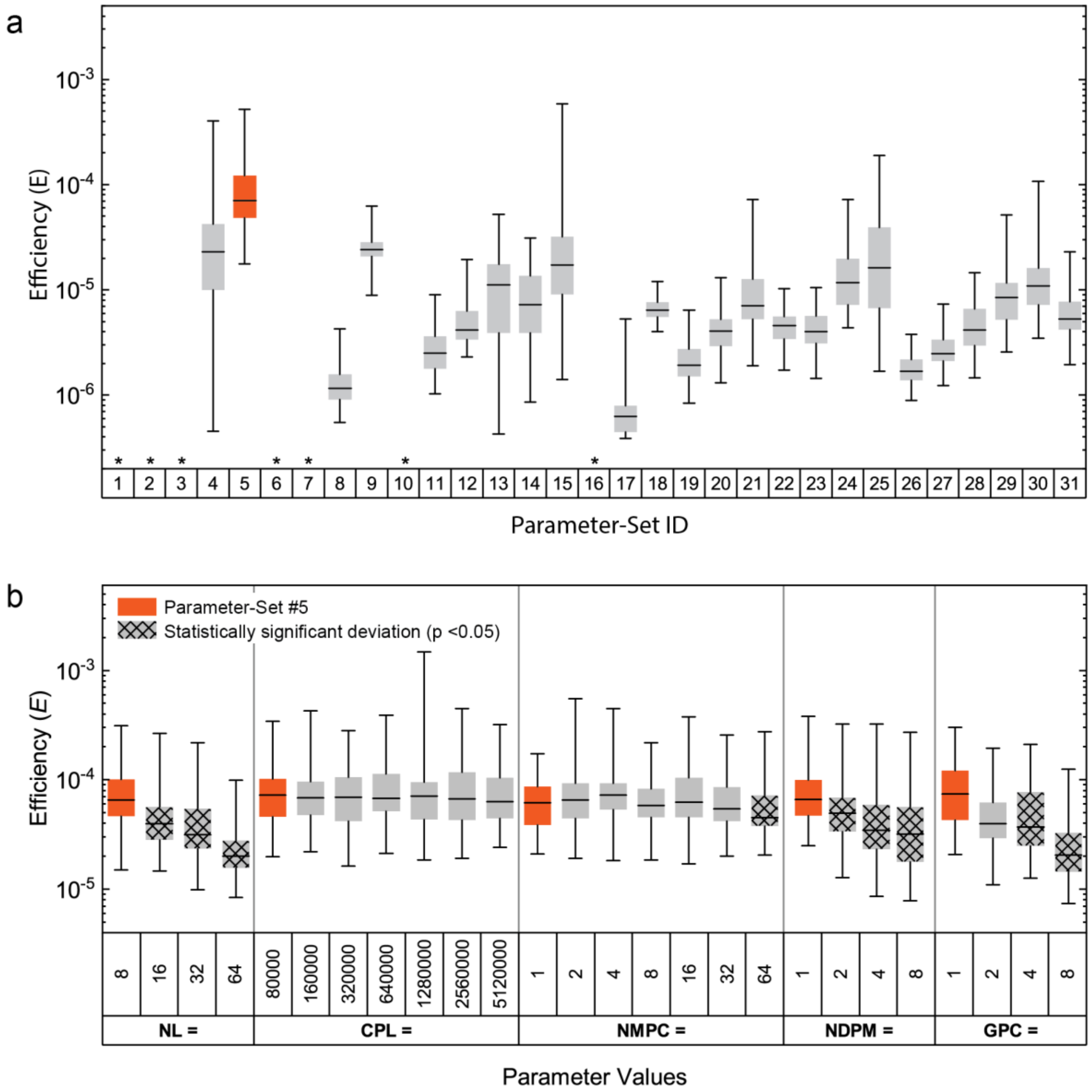


**Figure S14**. Efficiencies of the algorithm with varying parameters. Efficiency is a metric where higher efficiencies are more favorable. **(a)** Global sampling of efficiencies. Efficiencies of 31 parameter sets sampling a cross section of parameter space were determined using 81 independent design-trials. The efficiencies observed are summarized by a median line, a box spanning the 25$^{th}$-75$^{th}$ percentiles, and bars connecting the minimum and maximum values. Several parameter sets did not reliably solve the design problem given the finite resources and were termed inefficient, their results have been replaced with an asterisk (*). The region surrounding the most efficient parameter set observed (#5, colored orange) was further studied. **(b)** Localized sampling of efficiencies proximal to parameter set #5. Starting with the parameter values of set 5, each parameter was individually and systematically increased until a decrease in efficiency could be resolved. Statistical significance was evaluated using a 2-sided Kolmogorov-Smirnov test with $p < 0.05$.

## Supplementary Note 3: Calculation of Fitness Scores (*DevPro Program*)

The “Device Profiler” (abbreviated DevPro) computer program was created for calculating the fitness of a given network. This program was implemented in the java programing language and calculates unnecessary states using an exhaustive linear search which considers all possible base pairings. Pseudo code describing the linear search is provided below. Example calculations of N and O for a simple network consisting of two oligomers are illustrated in figures S3-01 and S3-02. By default, DevPro outputs values for ΔN, ΔO, ΔW. A number of additional properties can be calculated by the program if requested in the parameters file. The DevPro program uses the same 4 input files as the SeqEvo program (i.e., a fixed domains file, a variable domains file, an oligomers file, and a parameters file).

**Figure S15.** Algorithm pseudo-code for identifying inter-oligo unnecessary duplexes.

- For each oligomer-combination (e.g. Oligomer-1 and Oligomer-2)
    - For each possible alignment of the oligomers: (e.g. first base of oligomer-1 aligned with first base of oligomer 2).
        - L = 0
        - For each possible base-pair in this alignment: (e.g. first base of oligomer 1 and first base of oligomer 2)
            - If the bases are complementary:
                - L = L + 1
            - If the bases are not complementary and L > 0:
                - Record an inter-oligomer duplex of length L.
                - L = 0
        - If L > 0:
            - Record a inter-oligomer duplex of length L.

**Figure S16.** Algorithm pseudo-code for identifying intra-oligo unnecessary duplexes.

- For each oligomer (e.g. oligomer 1)
  - For each possible alignment of the bases: (e.g. the first base of oligomer 1 aligned with the last base of oligomer 1)
    - L = 0
    - For each possible base-pair in the alignment: (e.g. the first base of oligomer 1 and the last base of oligomer 1)
      - If the bases are complementary:
        - L = L + 1
      - If the bases are not complementary and L > 0:
        - Record an intra-oligomer duplex of length L.
        - L = 0
    - If L > 0:
      - Record an intra-oligomer duplex of length L

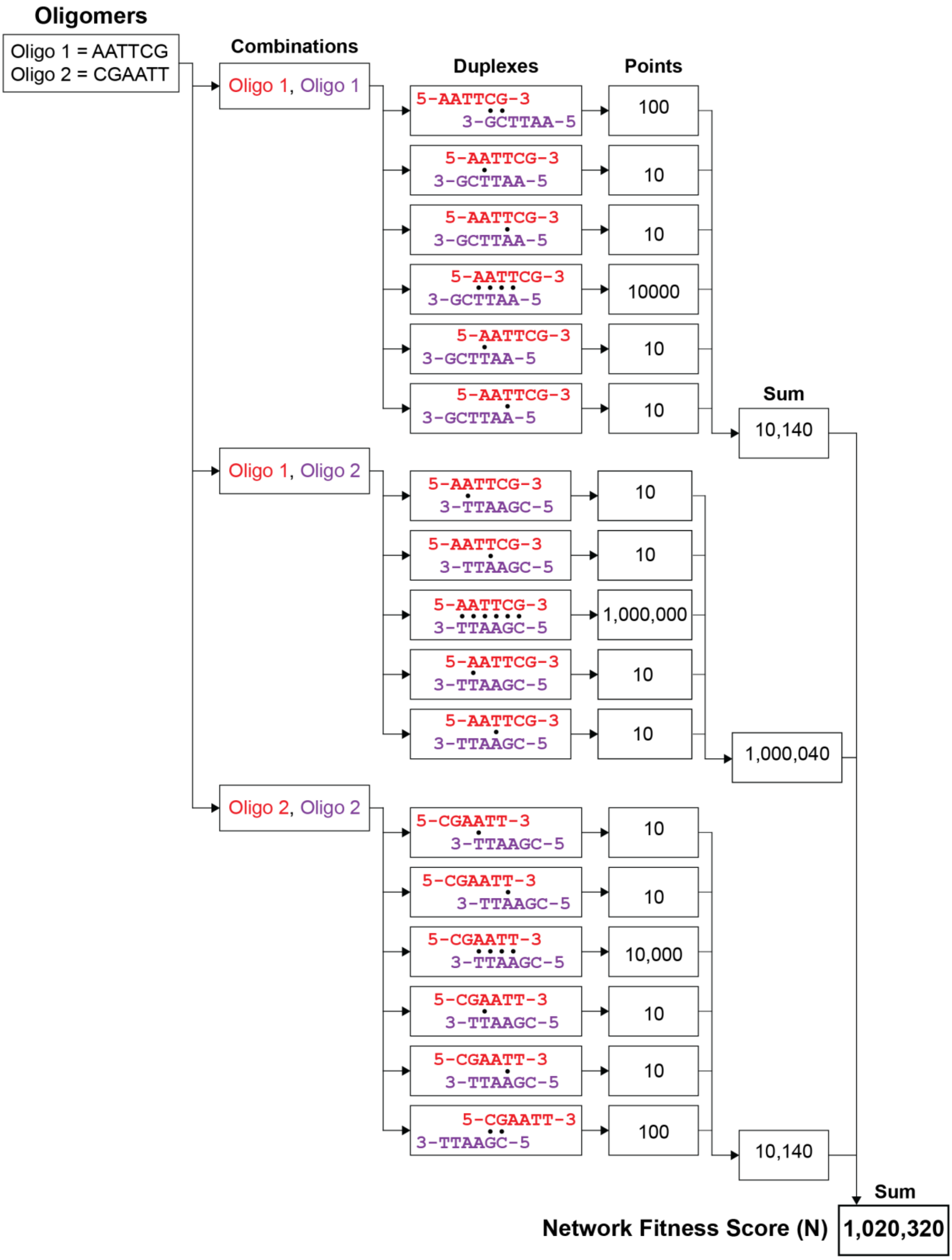


**Figure S17.** Example calculation of Network Fitness Score (N) for a network consisting of two oligomers.

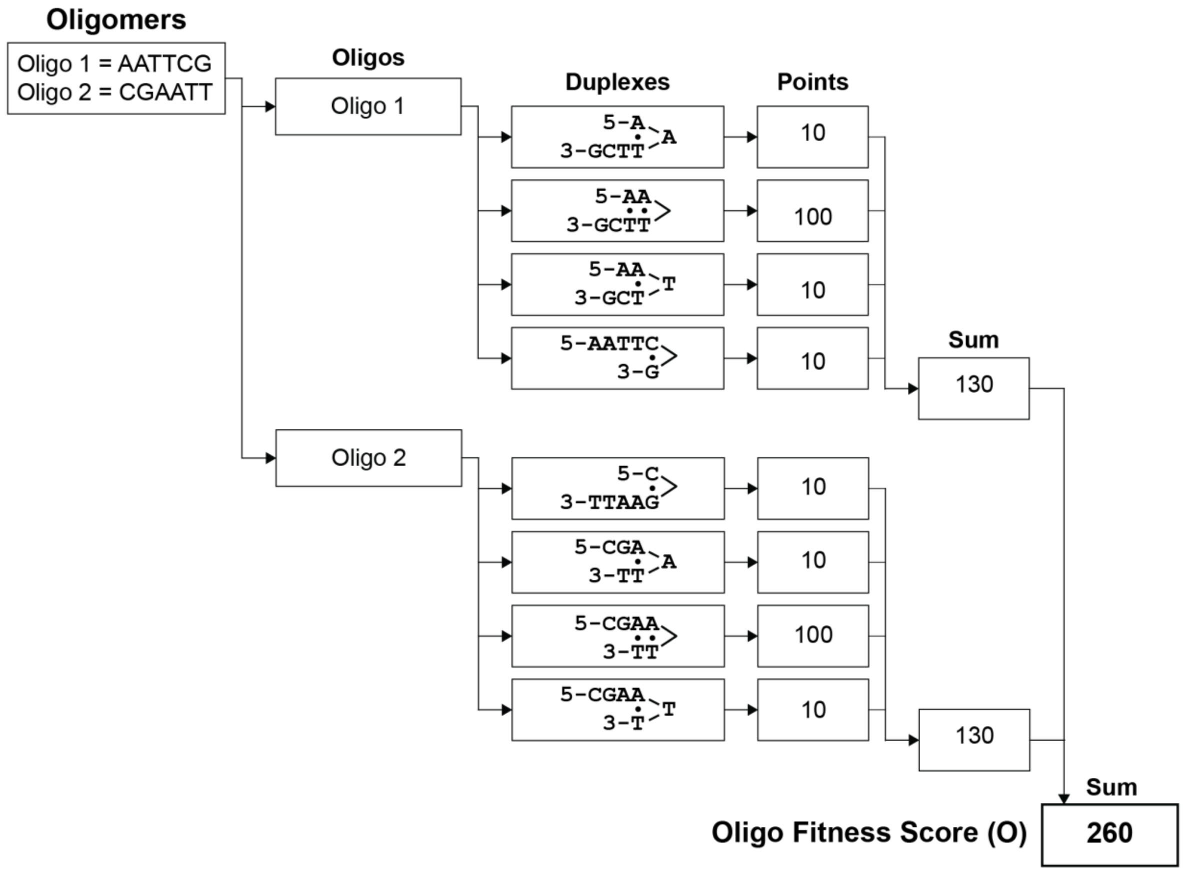


**Figure S18.** Example calculation of Oligomer Fitness Score (O) for a network consisting of two oligomers.

## Supplementary Note 4: Characterization of New Oligomers

**Experimental process**

Networks were characterized using the following procedure. HPLC purified oligomers were purchased from Integrated DNA Technologies. $S_1$ and $S_3$ were purchased with Cy3 and Cy5 dye modifications at their 5' ends, respectively. $S_2$ was purchased with a "Black Hole Quencher 1" modification at its 3' end. Oligomers were resuspended in 1x TE buffer (pH 8.0) at approximately 100 micromolar (µM) concentration. The concentration of each solution of oligomers was calculated using absorption measurements at 260nm (Thermo Nanodrop One spectrophotometer) and the absorbance coefficients were reported by Integrated DNA Technologies. Diluted working stocks containing approximately 1 µM $S_1$, 1 µM $S_2$, or 1 µM of both $S_2$ & $S_3$ were created by drawing from the 100 µM samples (the buffer for these samples was 1x TE buffer containing 1 M NaCl). These samples were allowed to incubate for at least 24 hours, and then sample concentration was again calculated using absorbance measurements. For the duplex formation reaction, aliquots from the 1 µM $S_1$, 1 µM $S_2$, and 1x TE (1 M NaCl) samples were incubated at the experimental temperature and then combined to create a 4 milliliter (mL) sample of 10 nM $S_1$, 10 nM $S_2$ and 1x TE (1M NaCl). For the displacement reaction, samples were incubated at the experimental temperature and then aliquots from the 1 µM $S_1$, 1 µM $S_2 + S_3$, and 1x TE (1 M NaCl) samples were combined to create a 4 mL sample of 10 nM $S_1$, 10 nM $S_2$, 10 nM $S_3$, and 1x TE (1M NaCl). A control sample was also created containing 4 mL of 10 nM $S_1$ in 1x TE (1M NaCl). Following the addition of the last aliquot, the 4 mL samples were mixed using a pipette, and immediately transferred to one of two Cary Eclipse spectrophotometers where fluorescence was monitored. Sample temperature was controlled using a temperature-controlled sample holder and this is also where reactants were incubated to reach experimental temperature. The concentration of unquenched Cy3 dye, which was proportional to the concentration of unreacted $S_1$, was monitored using excitation and emission wavelengths of 548 nanometers and 574 nanometers, respectively.

**Table S4.** New networks generated for experimental characterization. Strands $S_1$,$S_2$, and $S_3$ in the text are labeled $O_1$, $O_2$ and $O_3$. /5Cy3/ indicates the location of a 5' Cy3 modification. /3BHQ_1/ indicates the location 3' black hole quencher. /5Cy5/ indicates the location of a 5' Cy5 modification.

| **Name** | **Base Sequence (5'-3')** |
|---|---|
| Network W-Fit-1 | |
| W-Fit-1_O1 | /5Cy3/TCC AAT CGC CCG TCG TAG GTG TGT CAG TAA TAA AGC AGT TCT CTC CAT G |
| W-Fit-1_O2 | CAT GGA GAG AAC TGC TTT ATT ACT GAC ACA CCT ACG ACG GGC GAT TGG A/3BHQ_1/ |
| W-Fit-1_O3 | /5Cy5/TCC AAT CGC CCG TCG TAG GTG TGT CAG TAA TAA AGC AGT TC |
| | |
| Network W-Fit-2 | |
| W-Fit -2_O1 | /5Cy3/TAG TGT ATC CAA AGC CCG TAA GTC GCA GGT TCG TGT CAA TCT CTC CAT G |
| W-Fit-2_O2 | CAT GGA GAG ATT GAC ACG AAC CTG CGA CTT ACG GGC TTT GGA TAC ACT A/3BHQ_1/ |
| W-Fit-2_O3 | /5Cy5/TAG TGT ATC CAA AGC CCG TAA GTC GCA GGT TCG TGT CAA TC |
| | |
| Network W-Fit-3 | |
| W-Fit-3_O1 | /5Cy3/TCG TAG TGT GTC AGC AAA GTC CAA TAG GTT CGC CCG TAA TCT CTC CAT G |
| W-Fit-3_O2 | CAT GGA GAG ATT ACG GGC GAA CCT ATT GGA CTT TGC TGA CAC ACT ACG A/3BHQ_1/ |
| W-Fit-3_O3 | /5Cy5/TCG TAG TGT GTC AGC AAA GTC CAA TAG GTT CGC CCG TAA TC |
| | |
| Network N-Fit-1 | |
| N-Fit-1_O1 | /5Cy3/TTA TCG TCA CAG TTC GGT TCC AAA GGG CAA TCA GCG TAG TCT CTC CAT G |
| N-Fit-1_O2 | CAT GGA GAG ACT ACG CTG ATT GCC CTT TGG AAC CGA ACT GTG ACG ATA A/3BHQ_1/ |
| N-Fit-1_O3 | /5Cy5/TTA TCG TCA CAG TTC GGT TCC AAA GGG CAA TCA GCG TAG TC |
| | |
| Network N-Fit-2 | |
| N-Fit-2_O1 | /5Cy3/TCG GCG TAA GCA ATA GGT TTC ACA ATC CCA GGT AGT CGT TCT CTC CAT G |
| N-Fit-2_O2 | CAT GGA GAG AAC GAC TAC CTG GGA TTG TGA AAC CTA TTG CTT ACG CCG A/3BHQ_1/ |
| N-Fit-2_O3 | /5Cy5/TCG GCG TAA GCA ATA GGT TTC ACA ATC CCA GGT AGT CGT TC |
| | |
| Network N-Fit-3 | |
| N-Fit-3_O1 | /5Cy3/TGT AAA TCC CGT GCT AAA GTA TCG TCG CCA AGG TTC AGG TCT CTC CAT G |
| N-Fit-3_O2 | CAT GGA GAG ACC TGA ACC TTG GCG ACG ATA CTT TAG CAC GGG ATT TAC A/3BHQ_1/ |
| N-Fit-3_O3 | /5Cy5/TGT AAA TCC CGT GCT AAA GTA TCG TCG CCA AGG TTC AGG TC |
| | |
| Network O-Fit-1 | |
| O-Fit-1_O1 | /5Cy3/TAA AAG TGT GTA AAA AAG TCC CGT GTC CGT GTG TCC GTC CCT CTC CAT G |
| O-Fit -1_O2 | CAT GGA GAG GGA CGG ACA CAC GGA CAC GGG ACT TTT TTA CAC ACT TTT A/3BHQ_1/ |
| O-Fit -1_O3 | /5Cy5/TAA AAG TGT GTA AAA AAG TCC CGT GTC CGT GTG TCC GTC CC |
| | |
| Network O-Fit-2 | |
| O-Fit -2_O1 | /5Cy3/TCG TGT GTG TGT CCC GTA AAA GTA AAA AAG TCC CGT GTC CCT CTC CAT G |
| O-Fit -2_O2 | CAT GGA GAG GGA CAC GGG ACT TTT TTA CTT TTA CGG GAC ACA CAC ACG A/3BHQ_1/ |
| O-Fit -2_O3 | /5Cy5/TCG TGT GTG TGT CCC GTA AAA GTA AAA AAG TCC CGT GTC CC |

| **Name** | **Base Sequence (5’-3’)** |
|---|---|
| Network O-Fit-3 | |
| O-Fit -3_O1 | /5Cy3/TGT GTA AAA GTG TCC CGT GTC GTA AAA AAG TCC CGT GTC CCT CTC CAT G |
| O-Fit -3_O2 | CAT GGA GAG GGA CAC GGG ACT TTT TTA CGA CAC GGG ACA CTT TTA CAC A/3BHQ_1/ |
| O-Fit -3_O3 | /5Cy5/TGT GTA AAA GTG TCC CGT GTC GTA AAA AAG TCC CGT GTC CC |
| | |
| Network RND-1 | |
| RND-1_O1 | /5Cy3/GTG TCA ACA CCT CGC TAG AGA TGG TGC GCT AAA TTA CGC TTC TCC ATG |
| RND-1_O2 | CAT GGA GAA GCG TAA TTT AGC GCA CCA TCT CTA GCG AGG TGT TGA CAC /3BHQ_1/ |
| RND-1_O3 | /5Cy5/GTG TCA ACA CCT CGC TAG AGA TGG TGC GCT AAA TTA CGC T |
| | |
| Network RND-2 | |
| RND-2_O1 | /5Cy3/GAT TAG TCA TTA AGG GAT CGA CAC CAC GGG CTT CTT CCG ATC TCC ATG |
| RND-2_O2 | CAT GGA GAT CGG AAG AAG CCC GTG GTG TCG ATC CCT TAA TGA CTA ATC /3BHQ_1/ |
| RND-2_O3 | /5Cy5/GAT TAG TCA TTA AGG GAT CGA CAC CAC GGG CTT CTT CCG A |
| | |
| Network RND-3 | |
| RND-3_O1 | /5Cy3/TCC TAT GTA CAG TCG TAC GGA CTA TTG CGG AAC CCT GAG ATC TCC ATG |
| RND-3_O2 | CAT GGA GAT CTC AGG GTT CCG CAA TAG TCC GTA CGA CTG TAC ATA GGA /3BHQ_1/ |
| RND-3_O3 | /5Cy5/TCC TAT GTA CAG TCG TAC GGA CTA TTG CGG AAC CCT GAG A |

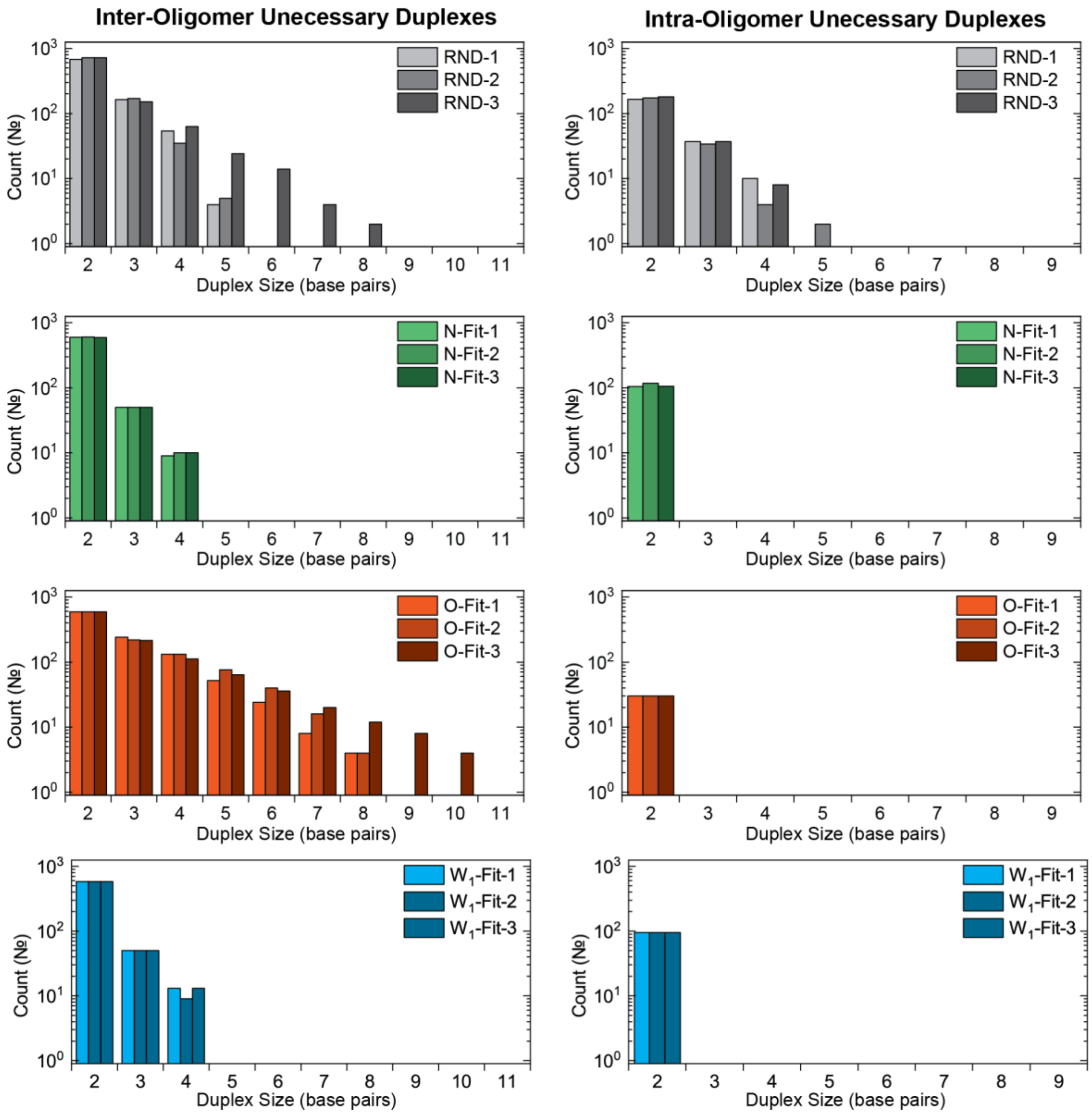


**Figure S19.** Profile of the unnecessary duplexes for each of the generated networks.

**Fluorescence Data and Fits**

All oligomers were ordered HPLC purified from Integrated DNA Technologies (www.idtdna.com). Strands $S_1$,$S_2$, and $S_3$ are labeled $O_1$, $O_2$ and $O_3$, respectively. Working stocks of reactants $O_1$, $O_2$, and $O_3$ were diluted to approximately 1 micromolar concentrations in 1x TE buffer. The concentration of these working stocks were calculated using absorbance at 260 nm (measured using a Thermo Nanodrop One) and the extinction coefficient reported by IDT. A working stock of the $S_2$ complex was prepared by combining stoichiometric amounts of $O_2$ and $O_3$ in 1x TE (1M NaCl added) and allowing to react for at least 24 hours. For fluorescent measurement, reactants were combined at 10 nanomolar concentrations in 1x TE containing 1M NaCl. Each sample was approximately 1 mL of buffer/strand solution in a 1cm x 1cm x 4cm disposable cuvette. Cuvettes were pre-treated with blocking buffer to prevent DNA adhesion to container walls.

Each report below consists of the following:

- Top Left: A plot of the recorded fluorescence for each sample. Samples began with oligomer "$O_1$" slightly above 10nM concentration. Sample fluorescence was monitored as the sample came to the same temperature as the sample holder. This was referred to as the first stage of the experiment – Stage A. Samples were then removed from the machine, during which the fluorescence dropped to approximately zero. While samples were removed, either the second reactant or an equivalent amount of buffer were added and the sample was mixed using a pipette. Following injection and mixing, reactant concentrations were 10nM. This was referred to as the second stage of the reaction – Stage B. During this stage a decrease in fluorescence is observed as the fluorescent dye localizes with the quenching molecule. This is expected to occur for both strand hybridization and strand displacement reactions, but not the control sample. The stability of the control sample provided confidence that the spectrophotometer and temperature controller is working as expected.
- Top Middle: Fluorescence vs time of the first stage of the experiment (Stage A). Fluorescence change during this stage is due to a change sample temperature while it equilibrates with the sample holder.
- Top right: Fluorescence vs time for the second stage of the reaction (Stage B). Fluorescence change during this stage is a result of the target reaction.
- Bottom Left: Plot of inverse strand concentration versus time for the second stage of the reaction. Linear fits were applied to the first 5s, 10s, 20s, 50s, 100s, 200s, and 500s of data (colored lines).
- Bottom Middle: $R^2$ for the seven linear fits. The slope of the fit with the largest $R^2$ was taken to be the measured rate.
- Bottom Right: Comparison of the best fit model (line) to the experimental data (points).

**Table S5.** Experiment number assignments.

| **Network** | **Temperature (°C)** | **Experiment No.** |
|---|---|---|
| RND-1 | 10 | 1 |
| | 20 | 2 |
| | 30 | 3 |
| | 40 | 4 |
| | 50 | 5 |
| | 60 | 6 |
| RND-2 | 10 | 7 |
| | 20 | 8 |
| | 30 | 9 |
| | 40 | 10 |
| | 50 | 11 |
| | 60 | 12 |
| RND-3 | 10 | 13 |
| | 20 | 14 |
| | 30 | 15 |
| | 40 | 16 |
| | 50 | 17 |
| | 60 | 18 |
| $W_1$-Fit-1 | 10 | 19 |
| | 20 | 20 |
| | 30 | 21 |
| | 40 | 22 |
| | 50 | 23 |
| | 60 | 24 |
| $W_1$-Fit-2 | 10 | 25 |
| | 20 | 26 |
| | 30 | 27 |
| | 40 | 28 |
| | 50 | 29 |
| | 60 | 30 |
| $W_1$-Fit-3 | 10 | 31 |
| | 20 | 32<br>33<br>34<br>35 |

| Network | Temperature (°C) | Experiment No. |
|---|---|---|
| | | 36<br>37 |
| | 30 | 38 |
| | 40 | 39<br>40<br>41<br>42<br>43<br>44 |
| | 50 | 45 |
| | 60 | 46 |
| O-Fit-1 | 10 | 47 |
| | 20 | 48 |
| | 30 | 19 |
| | 40 | 50 |
| | 50 | 51 |
| | 60 | 52 |
| O-Fit-2 | 10 | 53 |
| | 20 | 54 |
| | 30 | 55 |
| | 40 | 56 |
| | 50 | 57 |
| | 60 | 58 |
| O-Fit-3 | 10 | 59 |
| | 20 | 60 |
| | 30 | 61 |
| | 40 | 62 |
| | 50 | 63 |
| | 60 | 64 |
| N-Fit-1 | 10 | 65 |
| | 20 | 66 |
| | 30 | 67 |
| | 40 | 68 |
| | 50 | 69 |
| | 60 | 70 |
| N-Fit-2 | 10 | 71 |
| | 20 | 72 |

| Network | Temperature (°C) | Experiment No. |
|---|---|---|
| | 30 | 73 |
| | 40 | 74 |
| | 50 | 75 |
| | 60 | 76 |
| N-Fit-3 | 10 | 77 |
| | 20 | 78 |
| | 30 | 79 |
| | 40 | 80 |
| | 50 | 81 |
| | 60 | 82 |

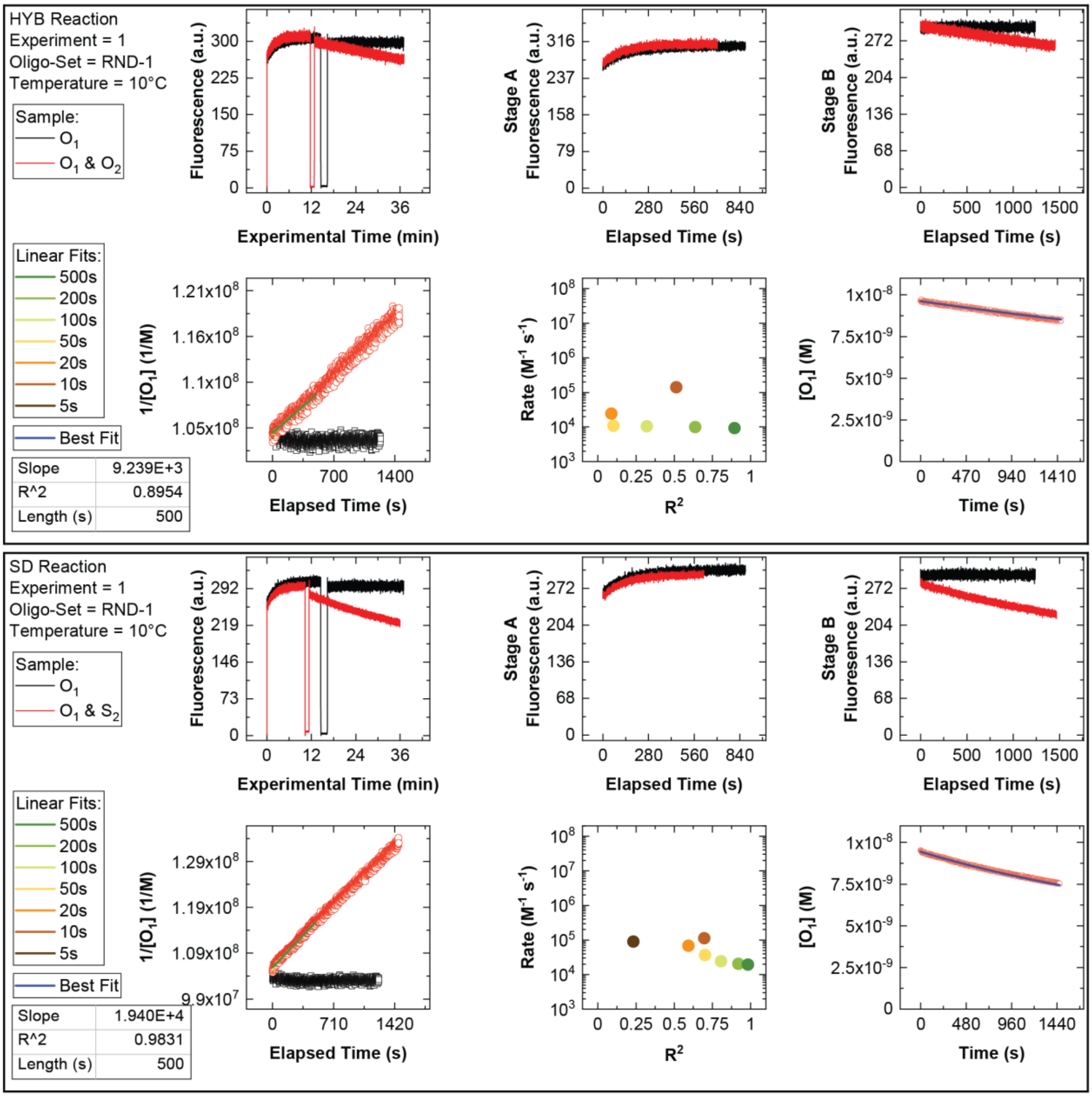


**Figure S20.** Report from experiment 1.

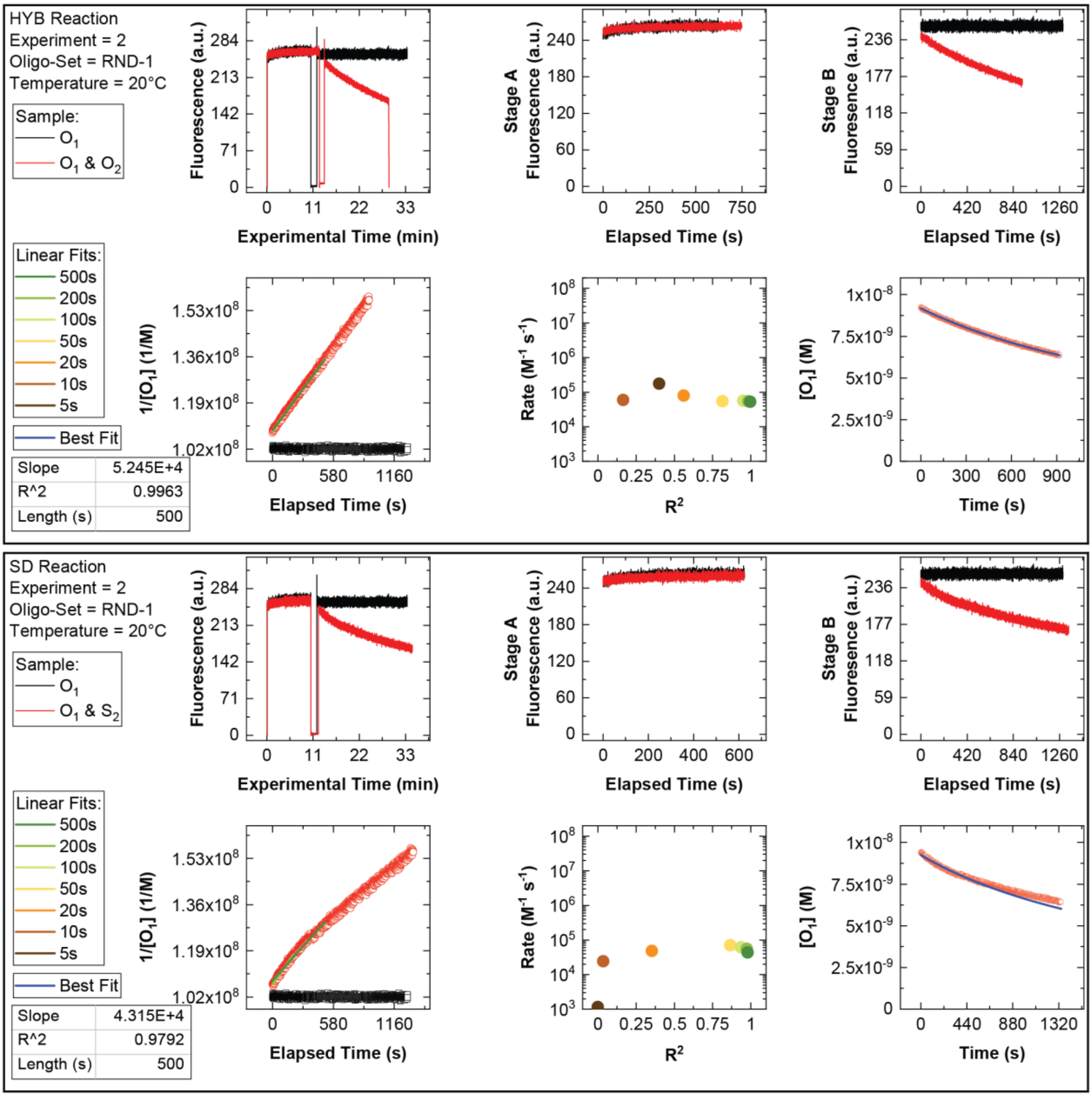


**Figure S21.** Report from experiment 2.

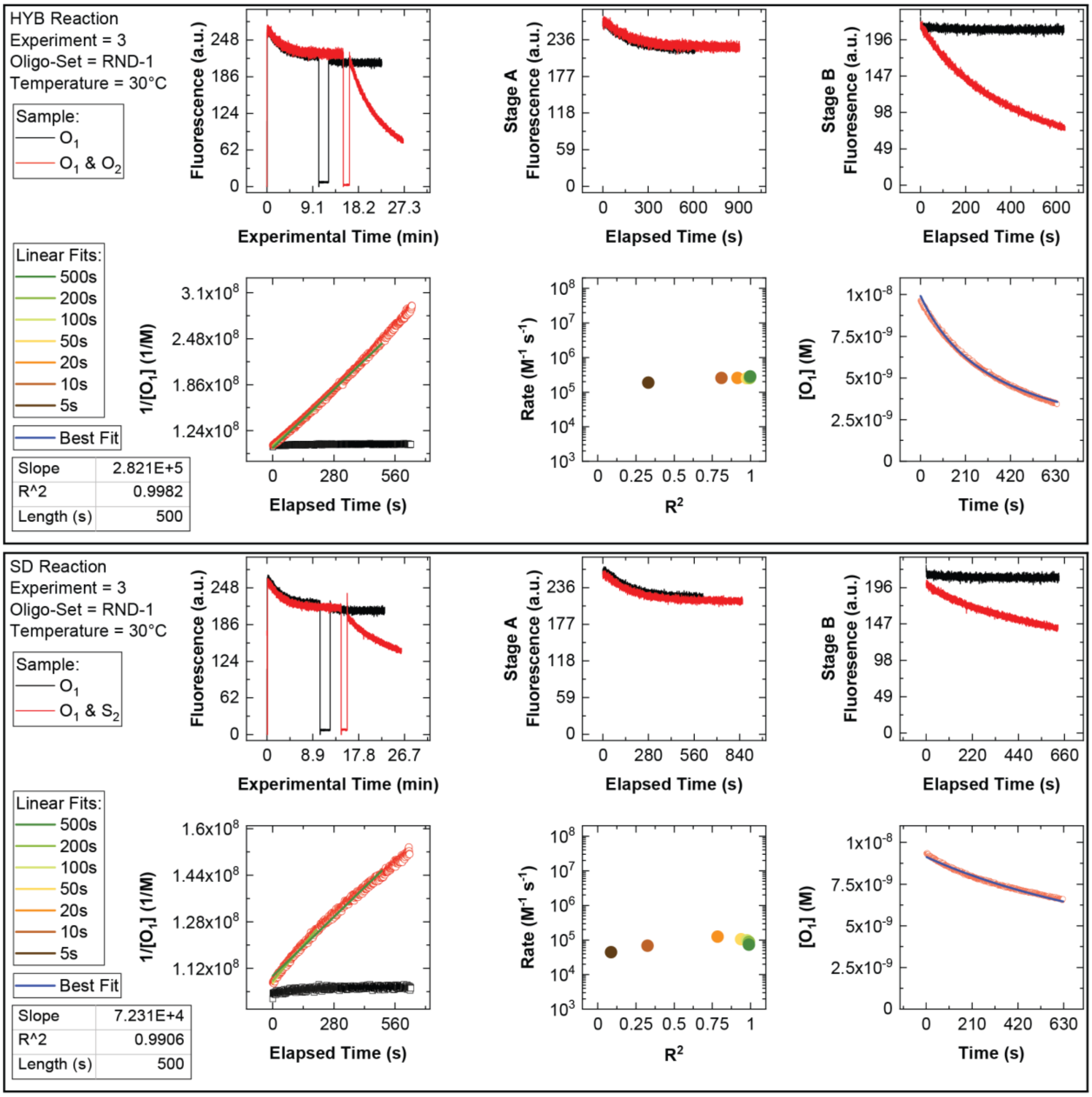


**Figure S22.** Report from experiment 3.

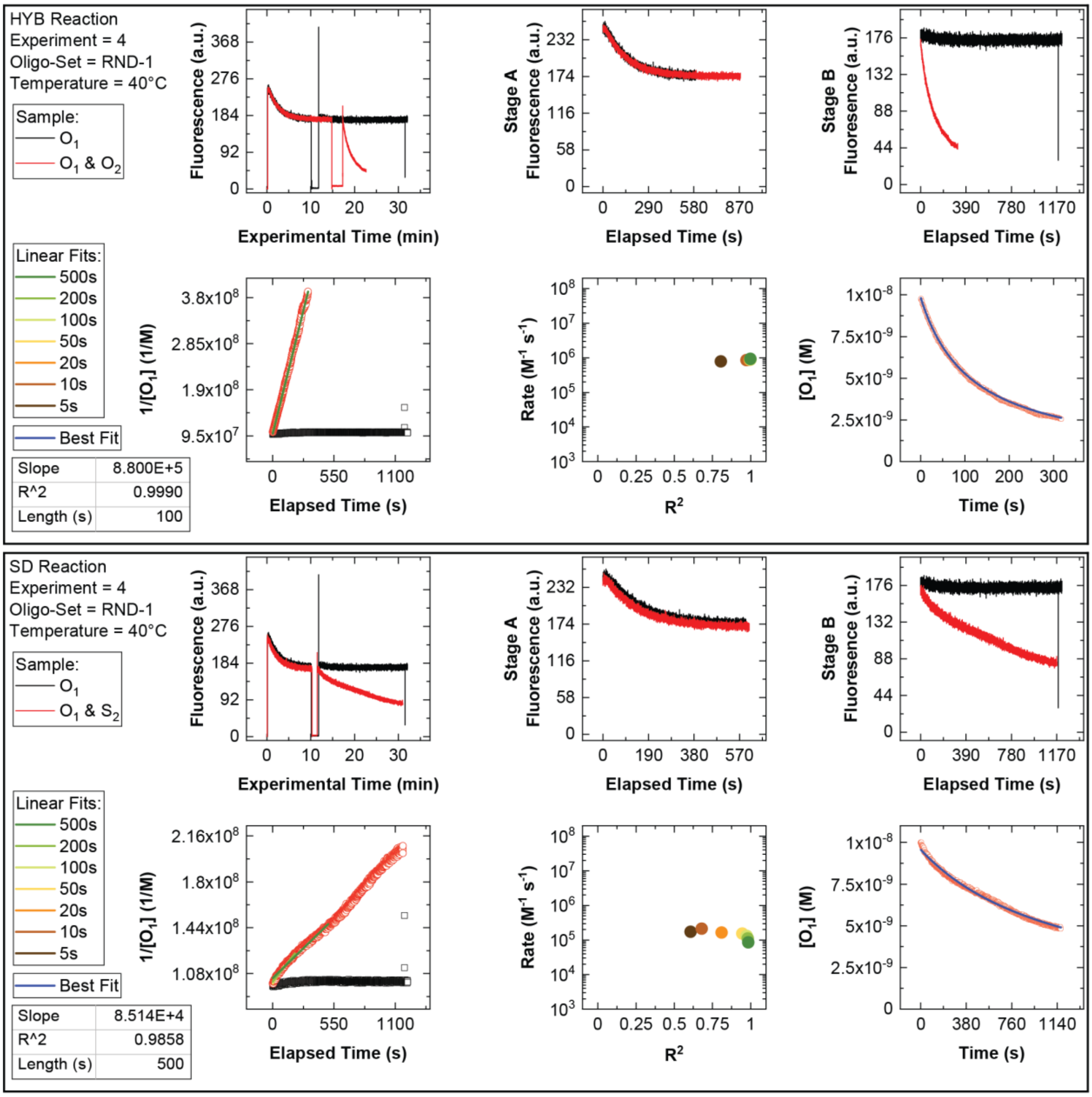


**Figure S23.** Report from experiment 4.

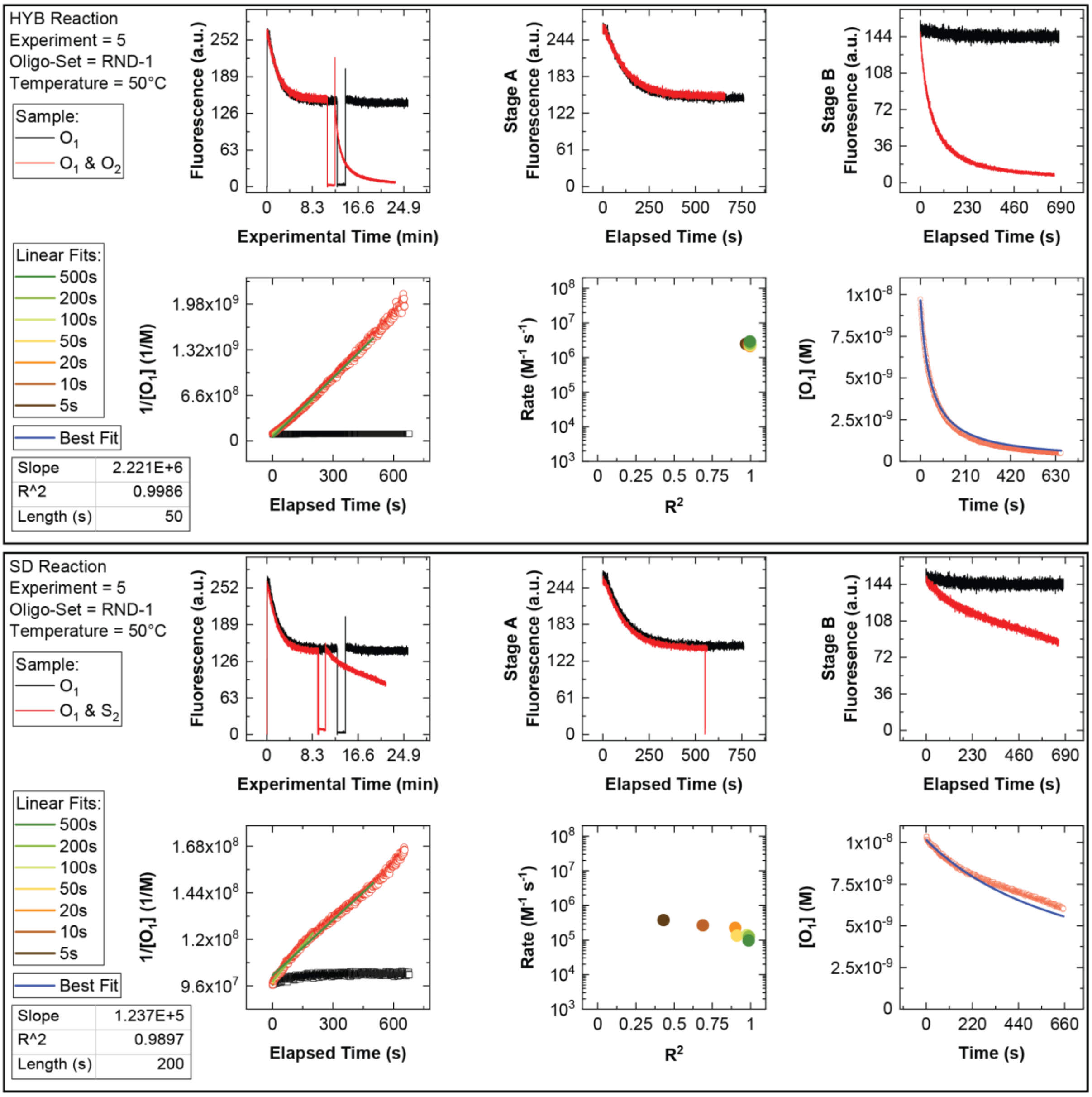


**Figure S24.** Report from experiment 5.

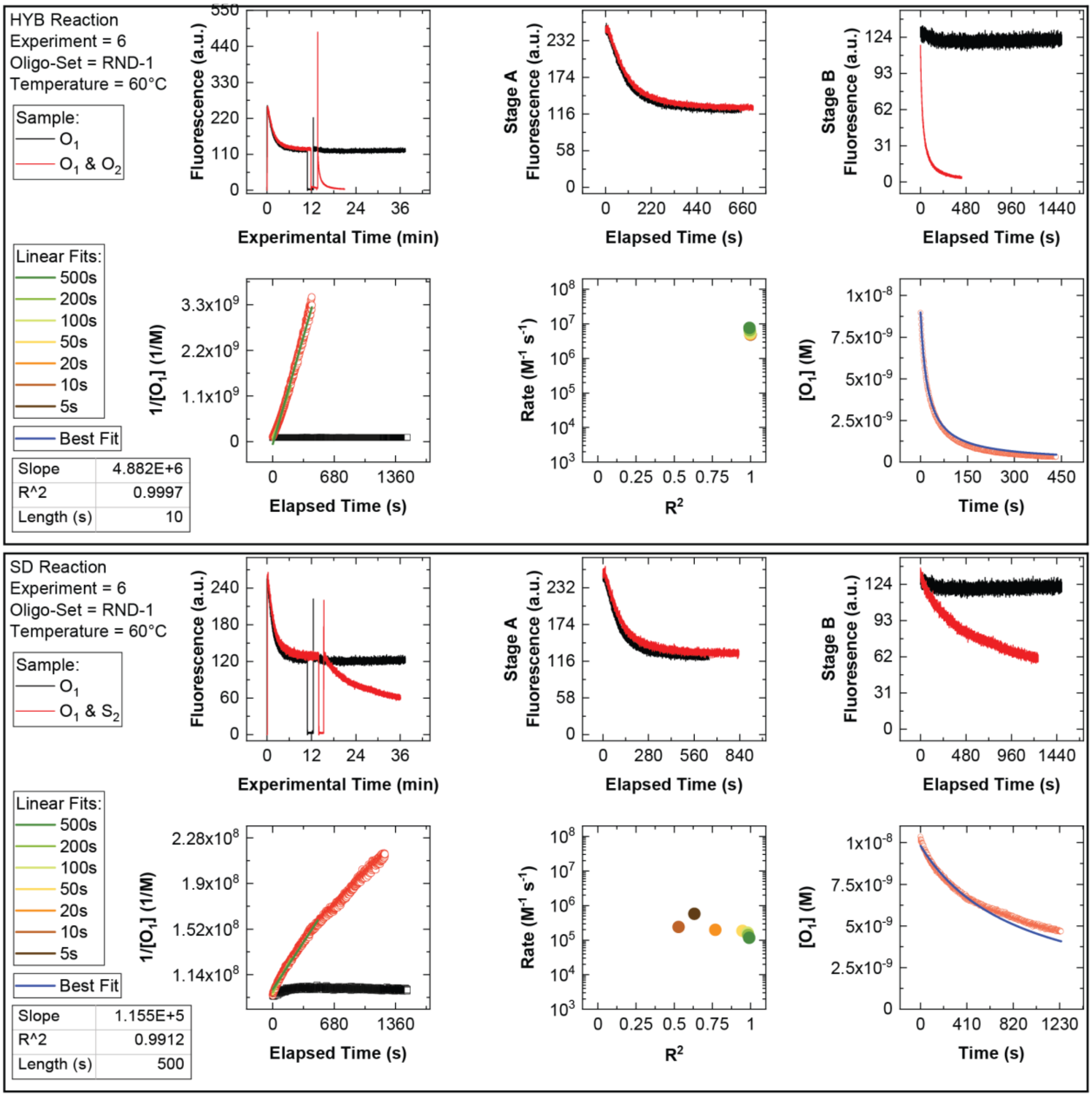

**Figure S25.** Report from experiment 6.

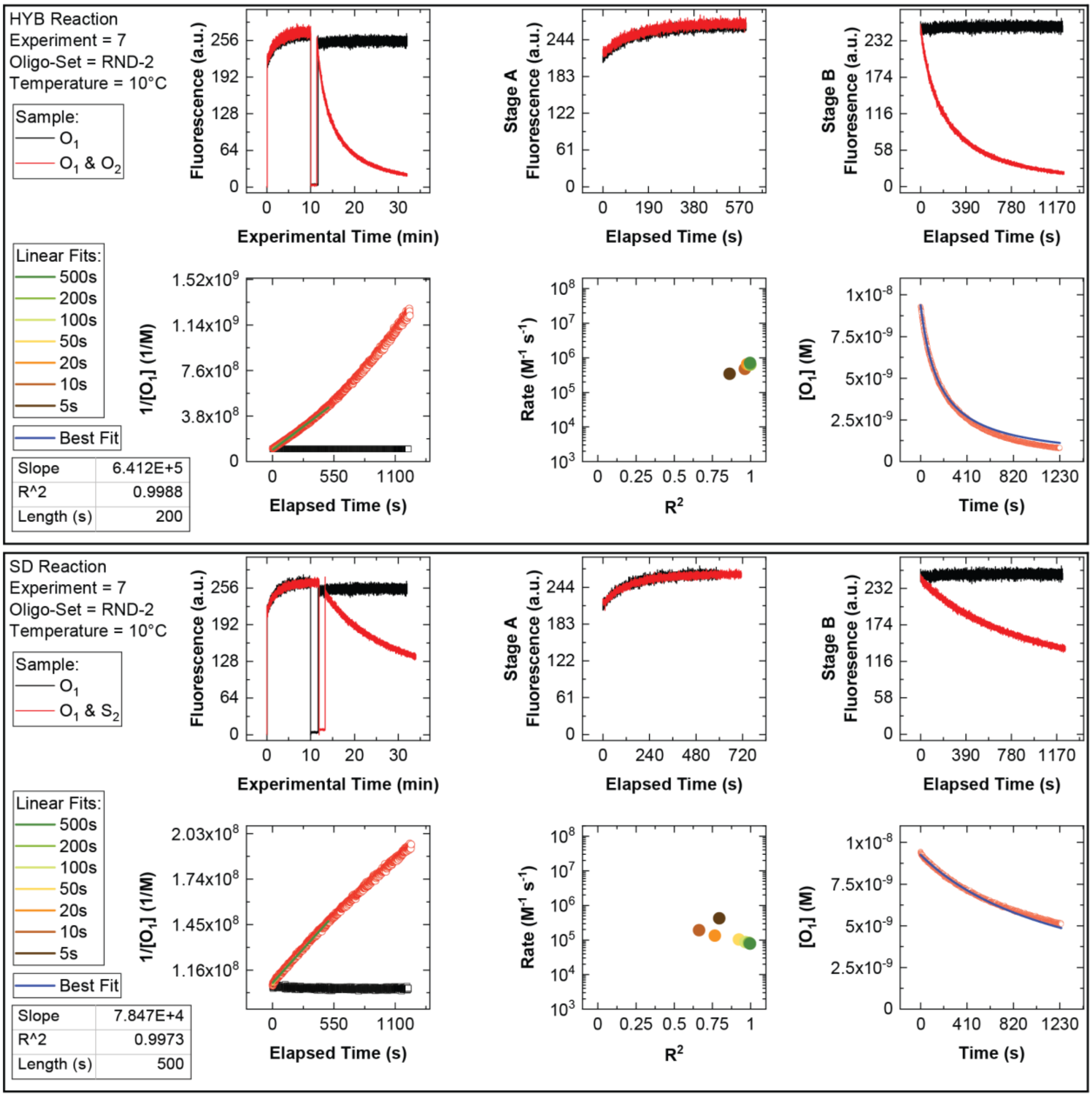


**Figure S26.** Report from experiment 7.

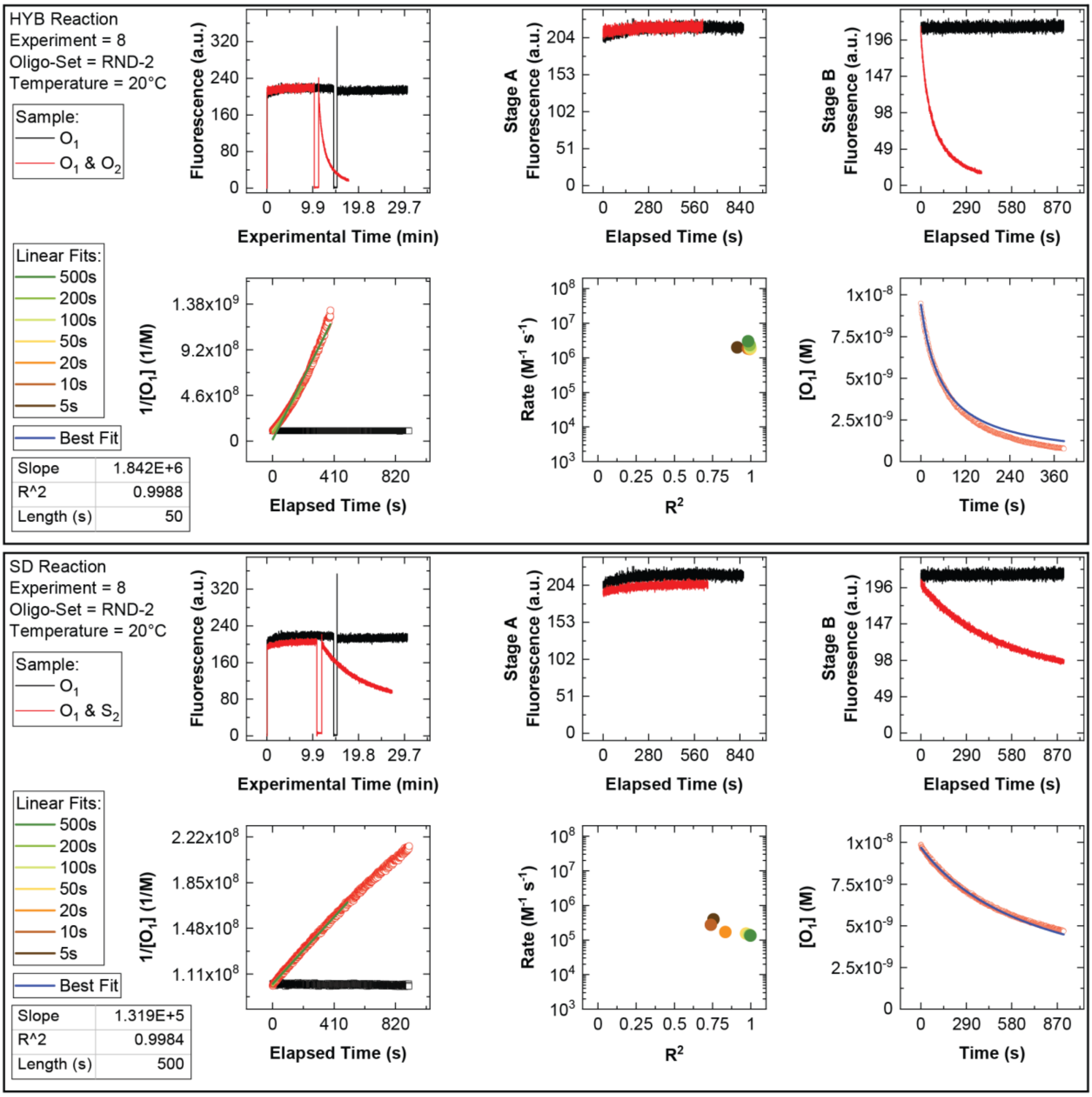


**Figure S27.** Report from experiment 8.

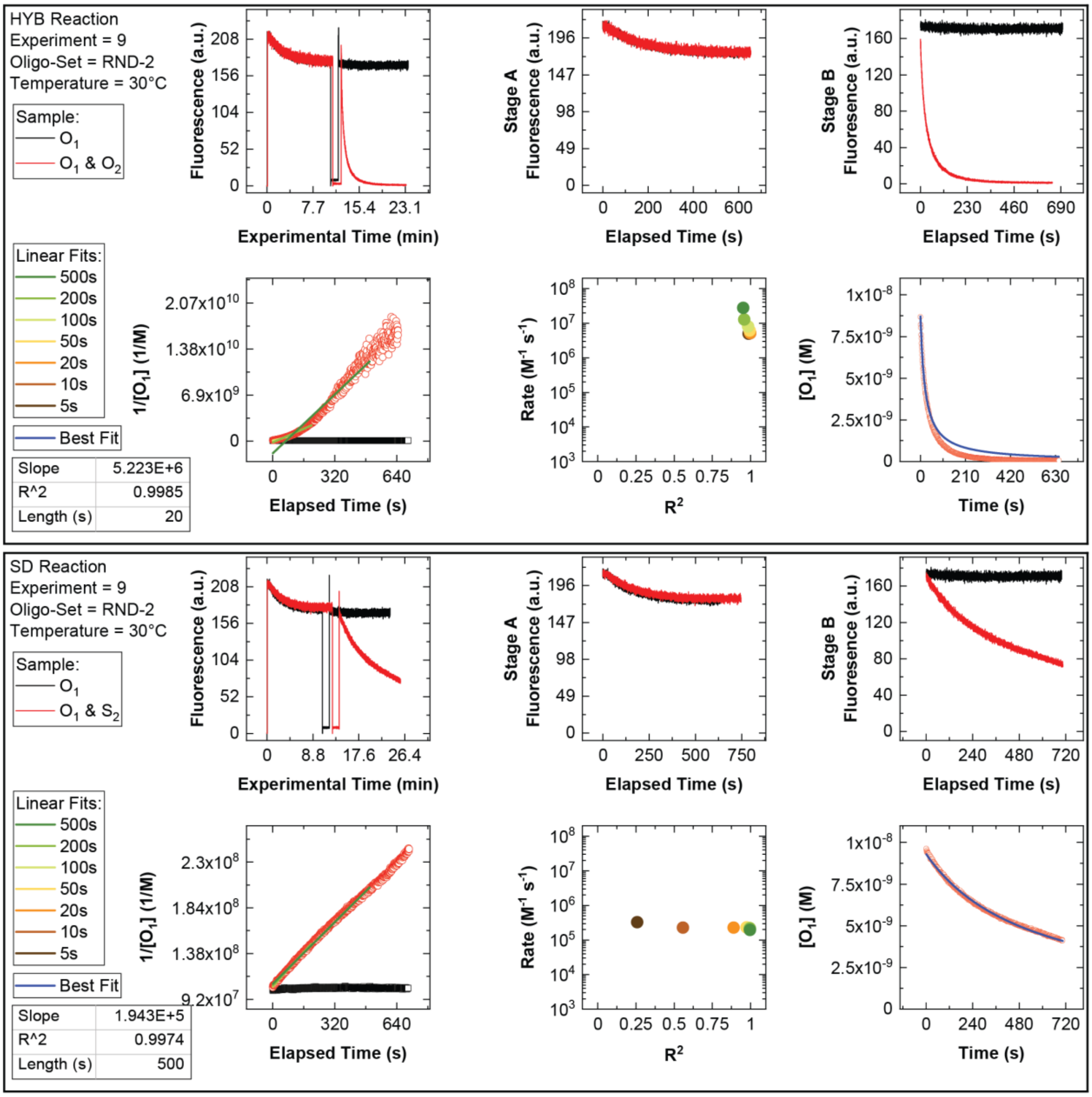


**Figure S28.** Report from experiment 9.

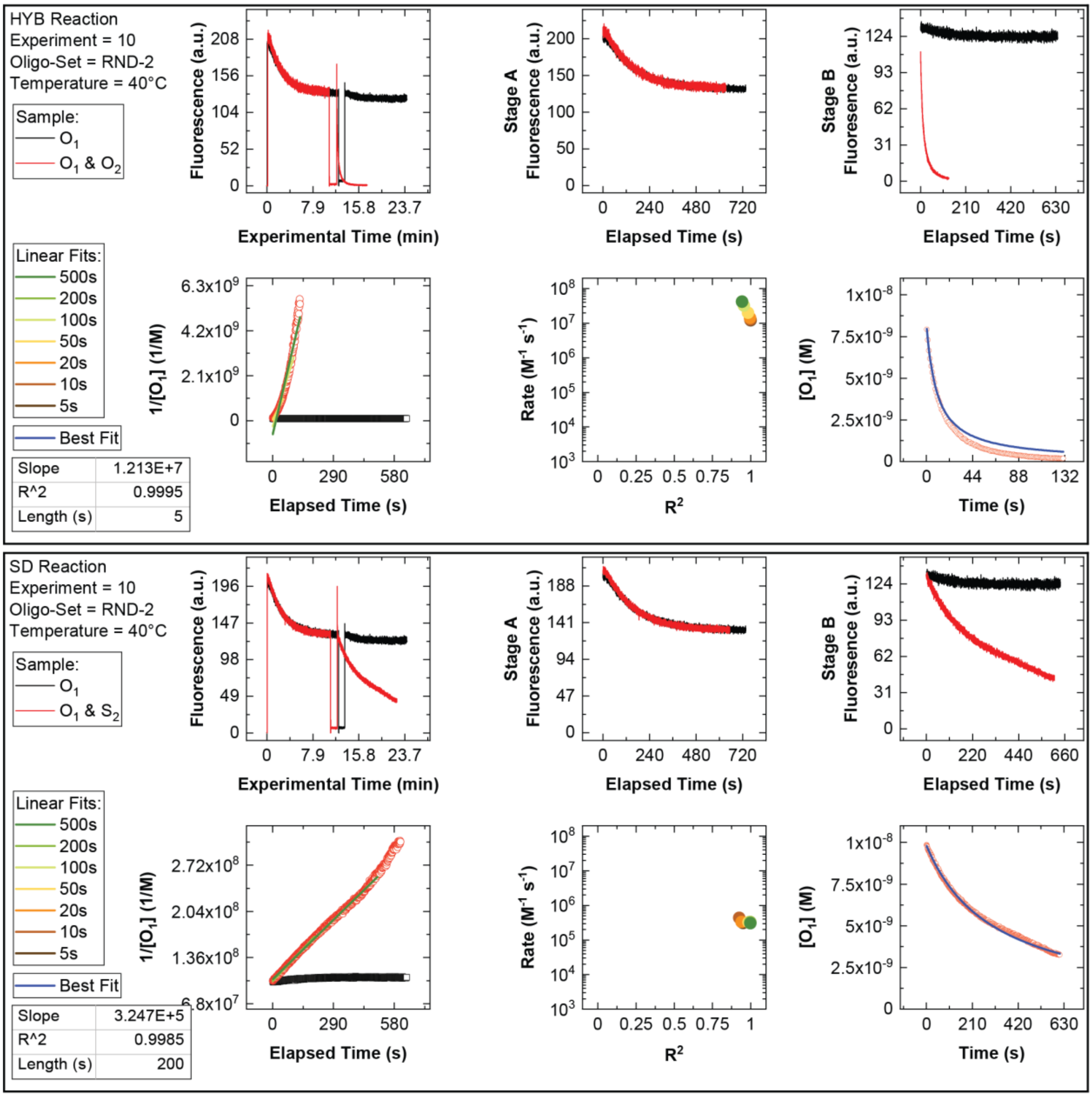


**Figure S29.** Report from experiment 10.

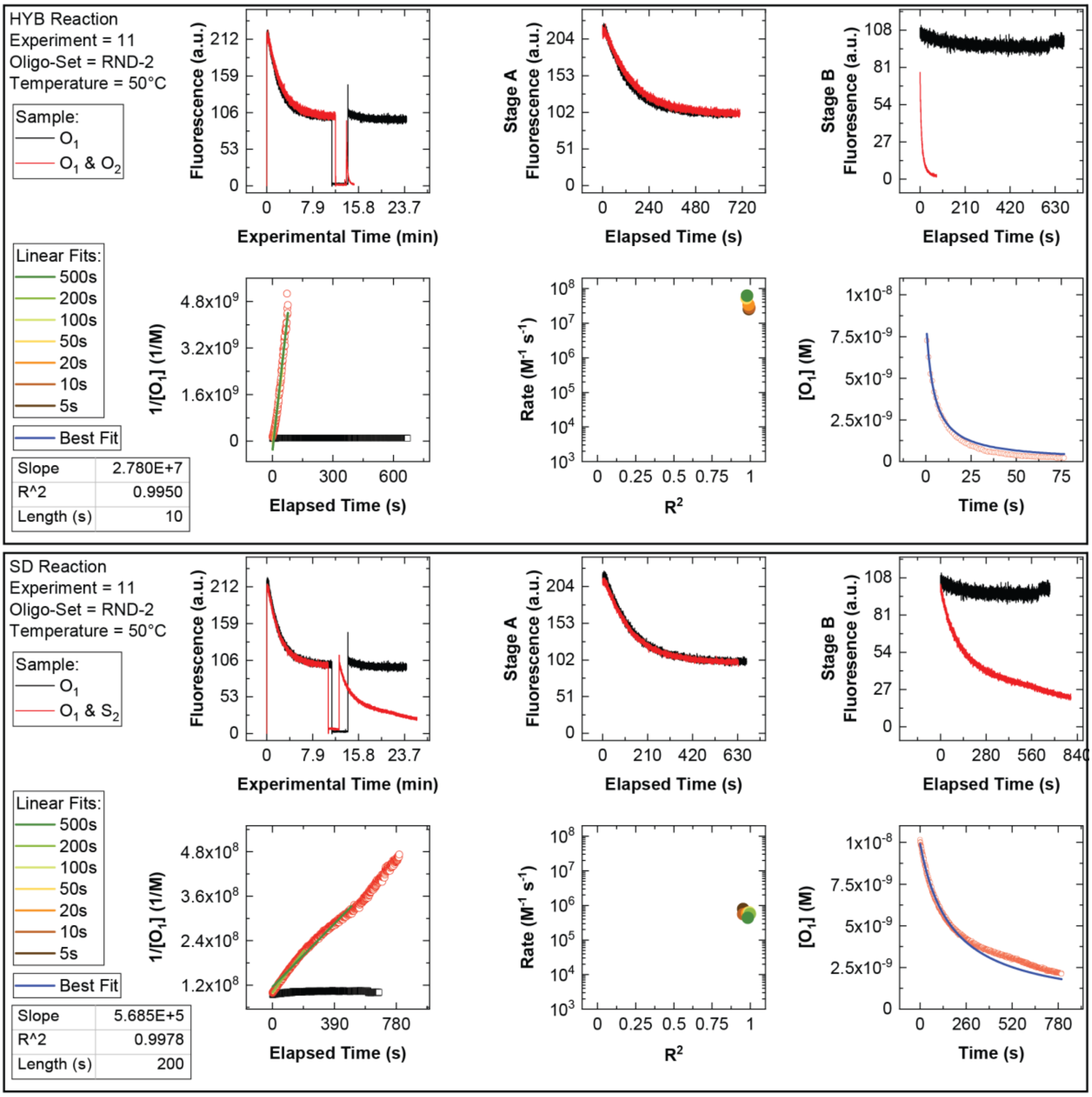


**Figure S30.** Report from experiment 11.

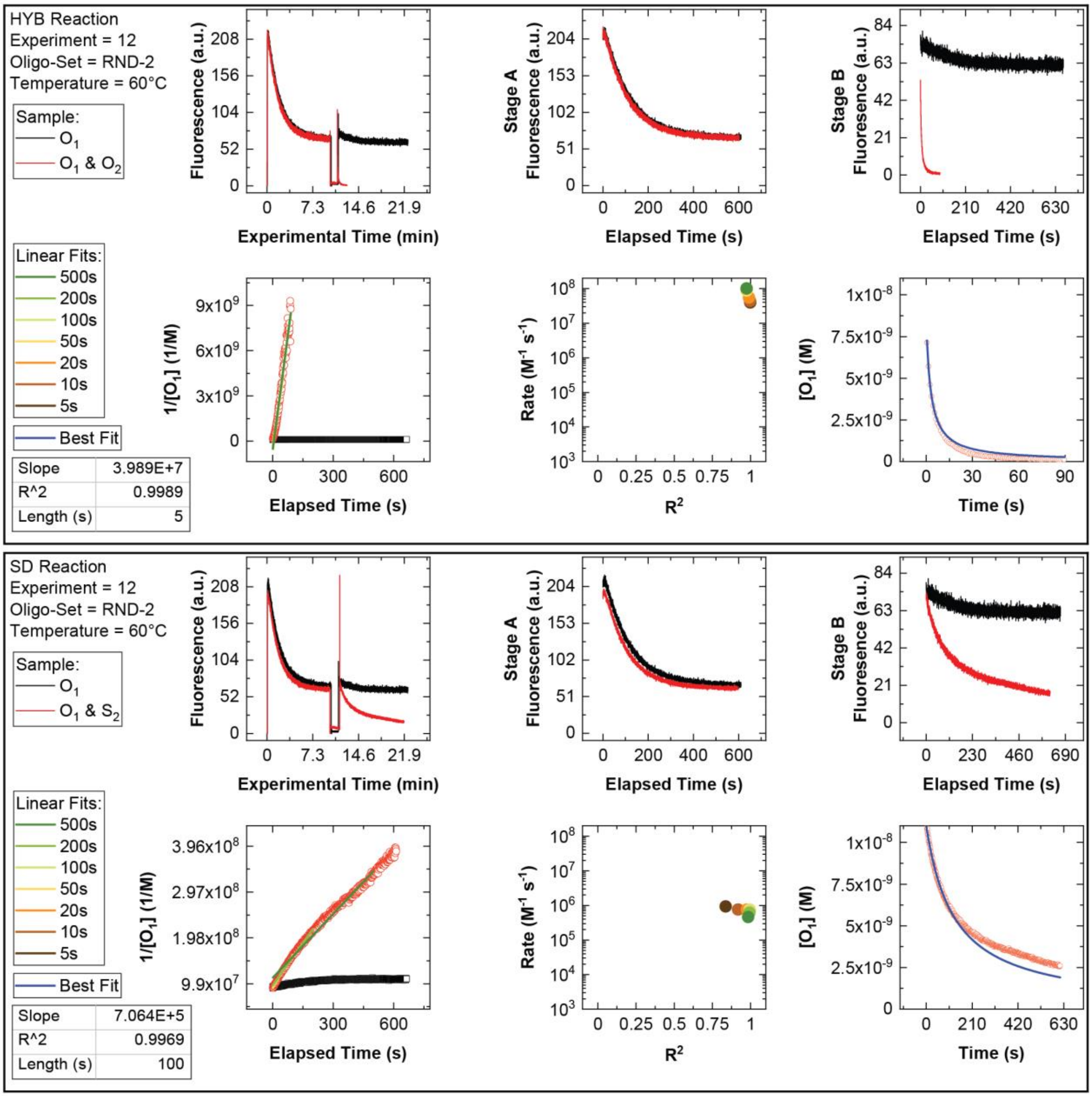


**Figure S31.** Report from experiment 12.

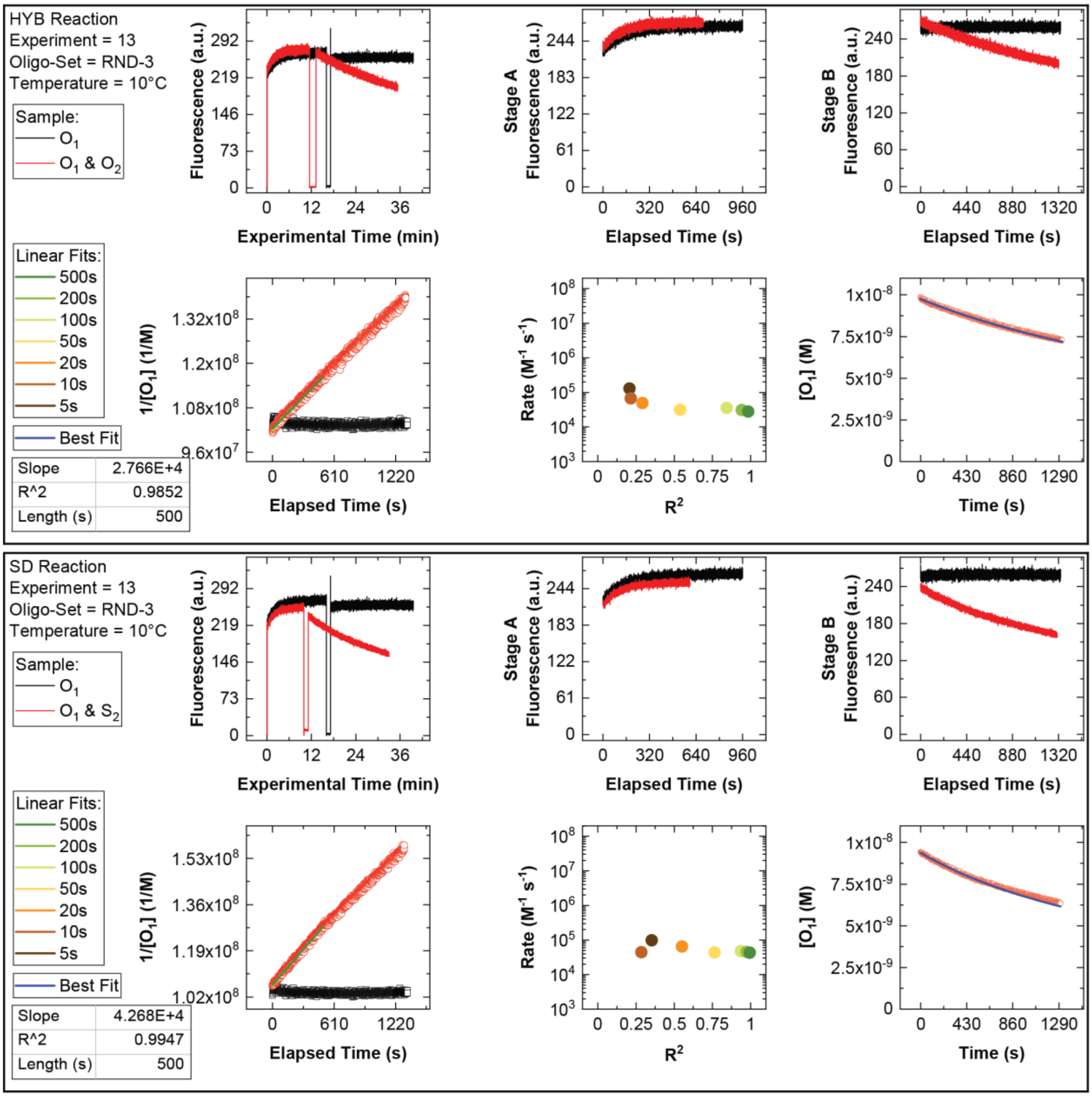


**Figure S32.** Report from experiment 13.

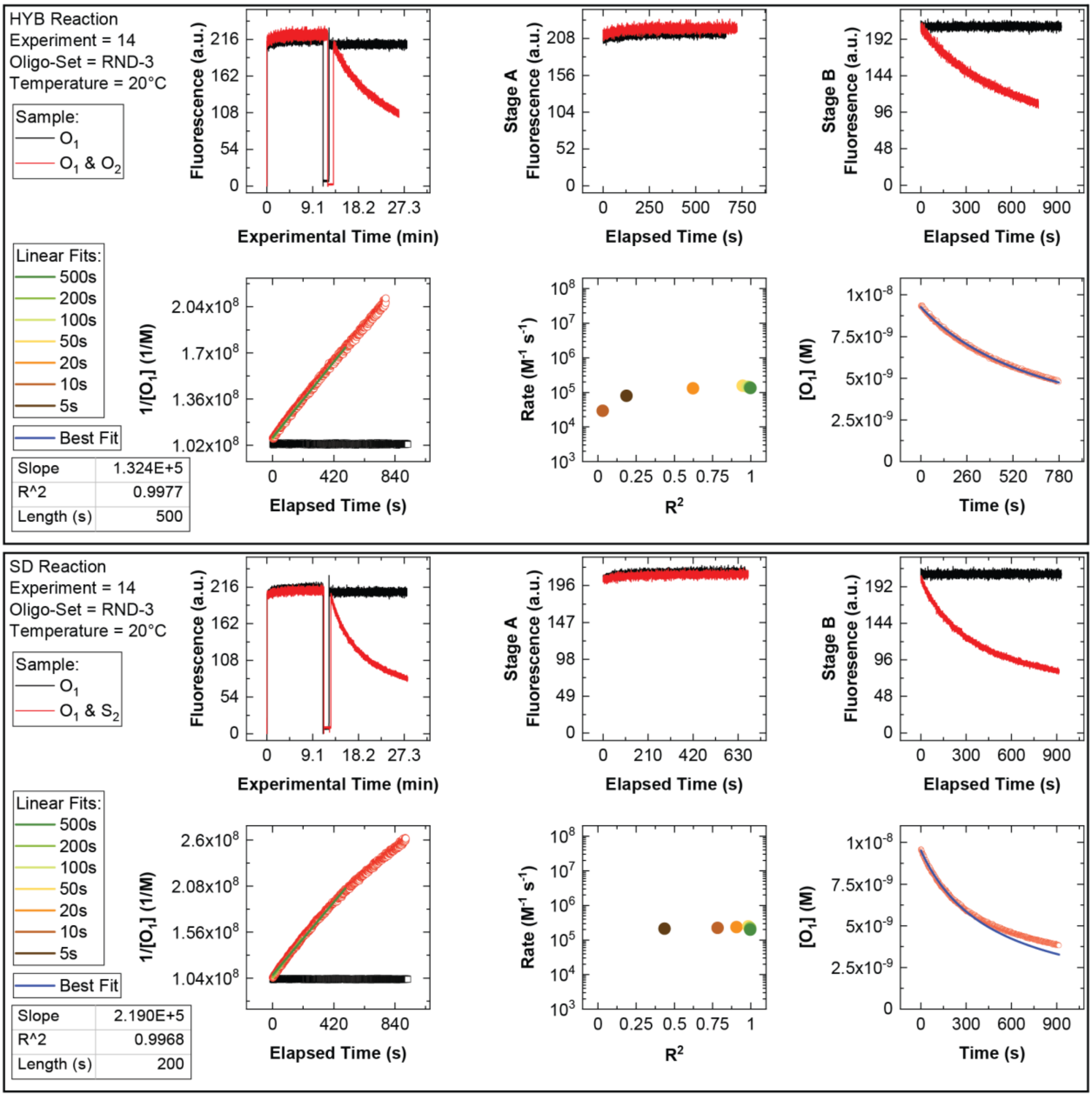


**Figure S33.** Report from experiment 14.

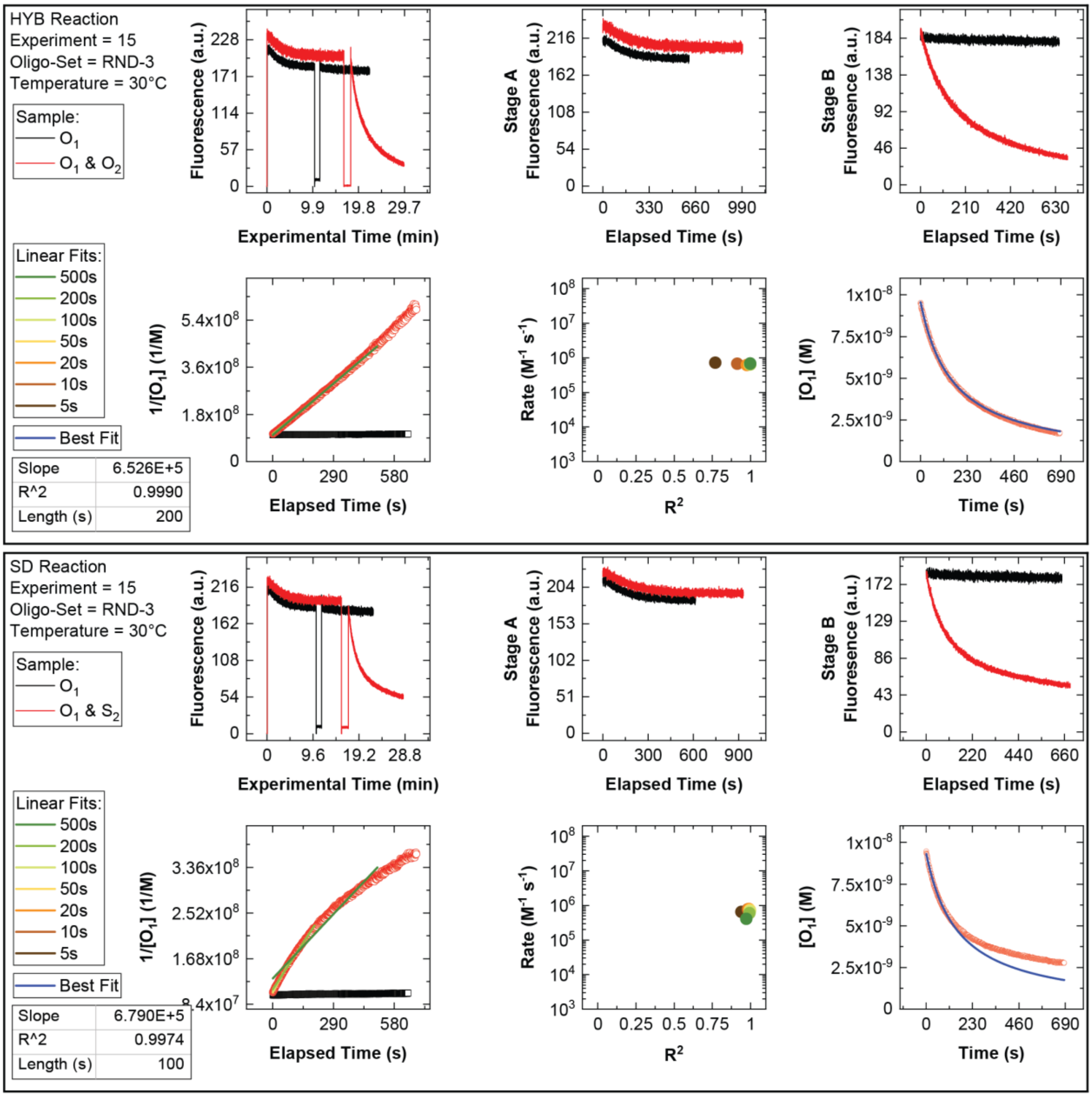


**Figure S34.** Report from experiment 15.

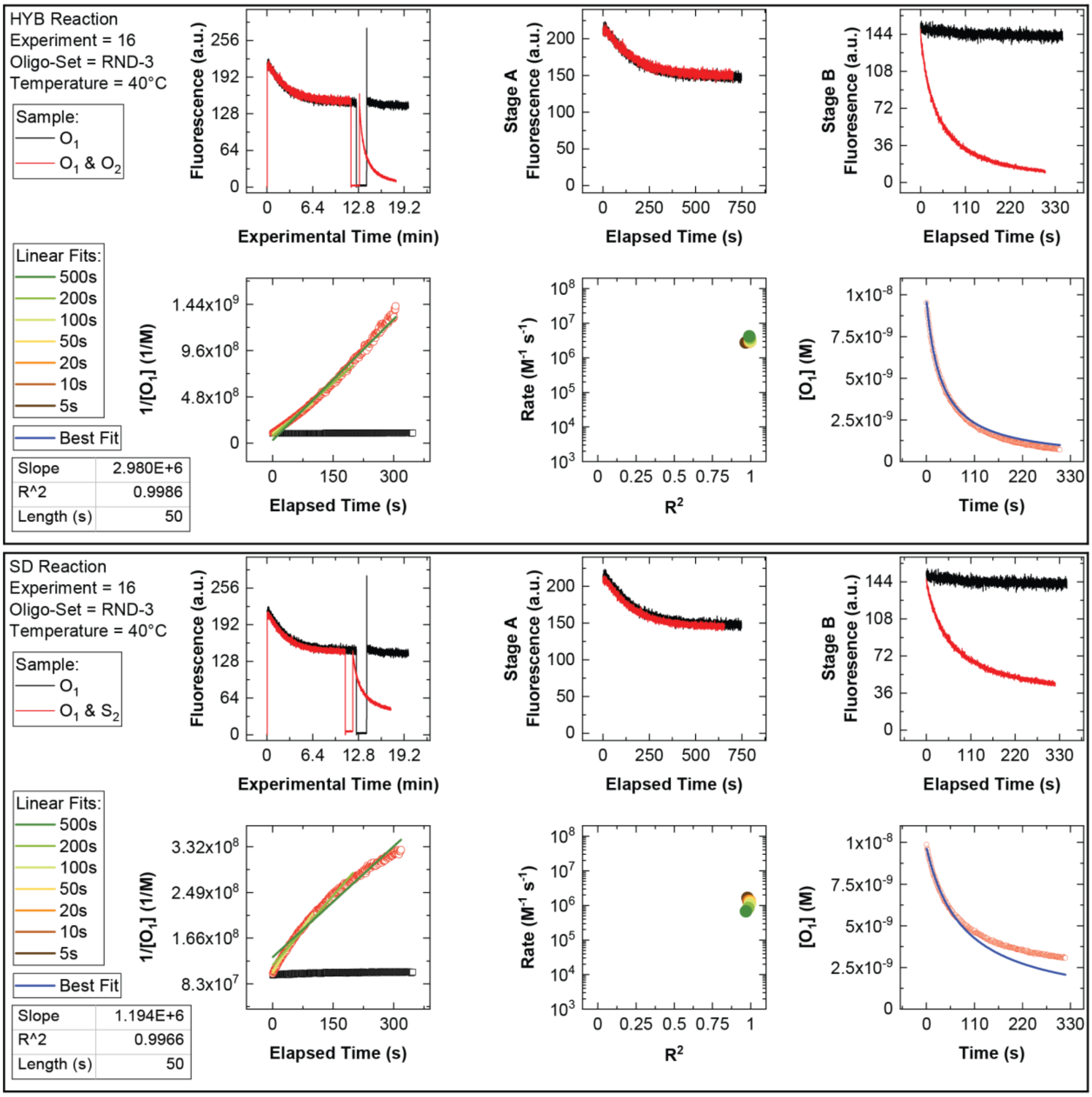


**Figure S35.** Report from experiment 16.

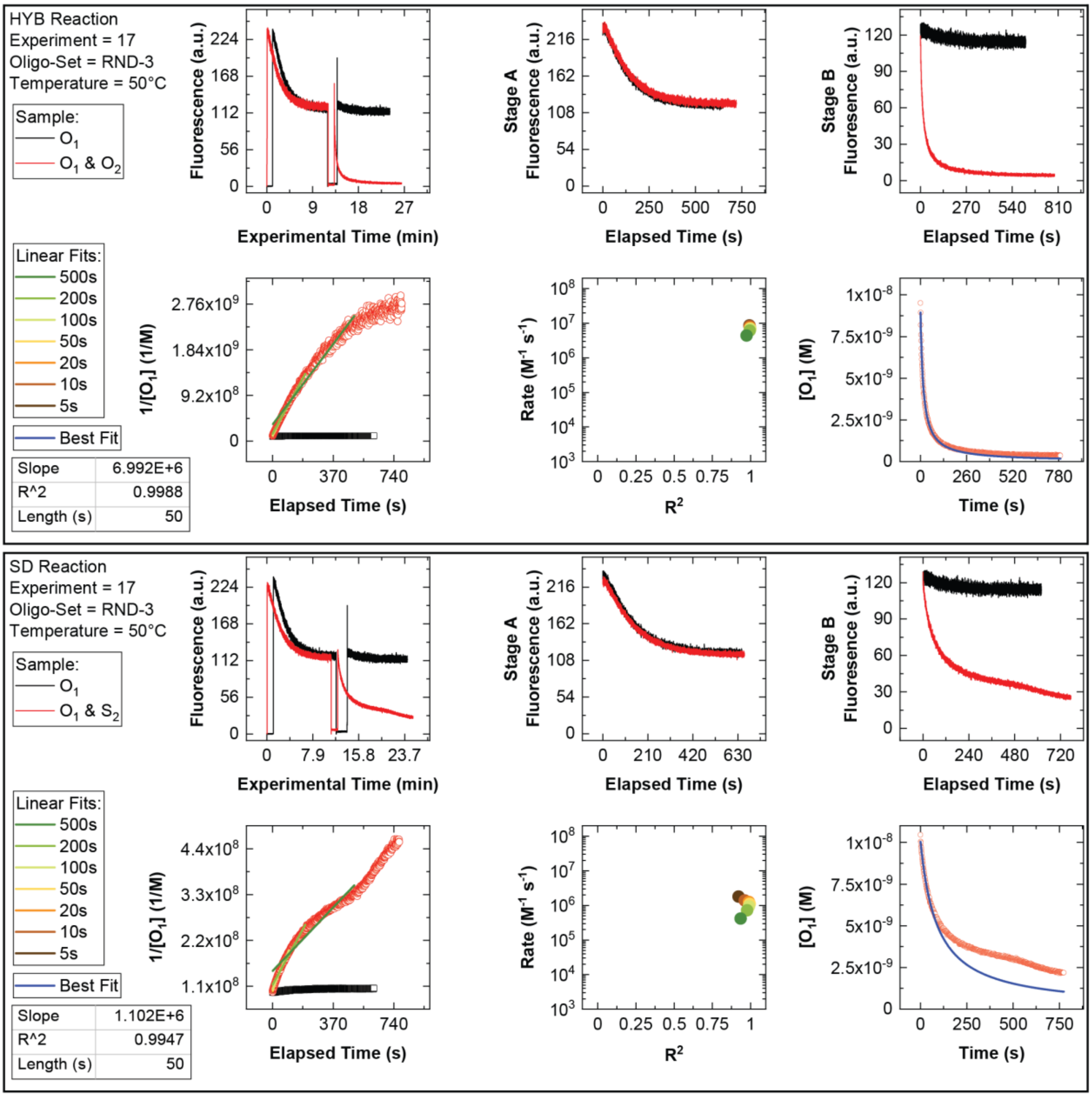


**Figure S36.** Report from experiment 17.

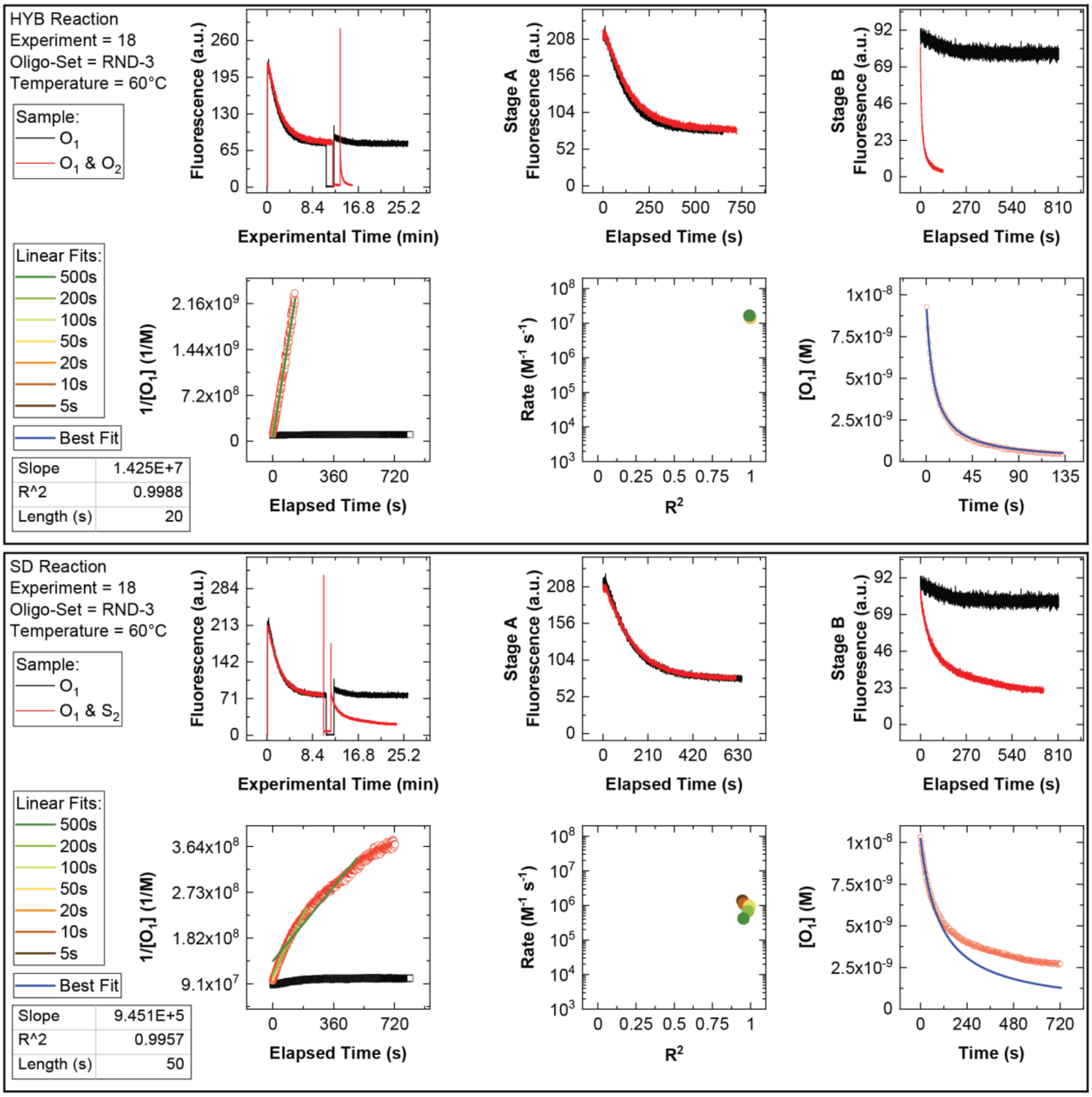


**Figure S37.** Report from experiment 18.

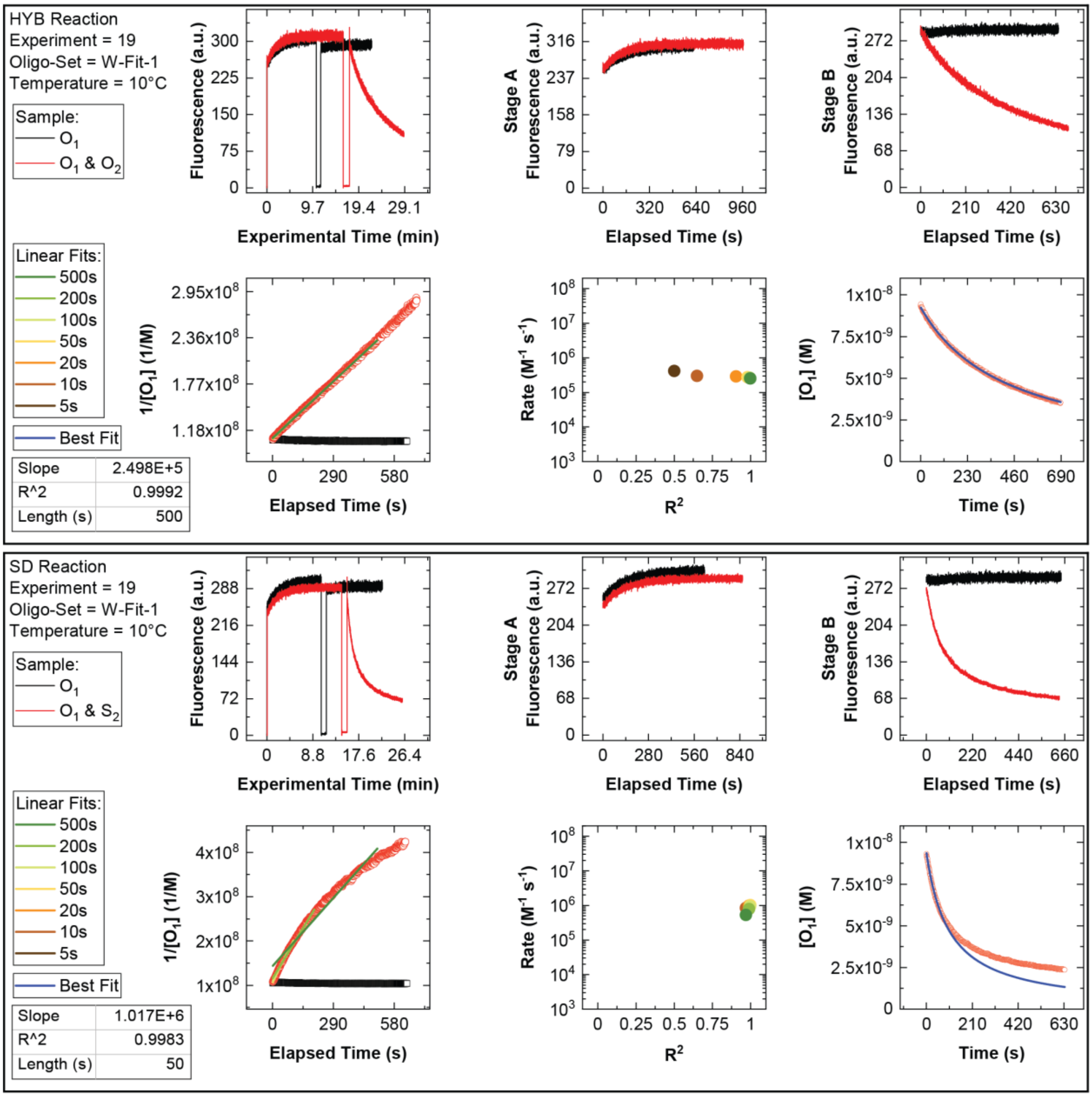


**Figure S38.** Report from experiment 19.

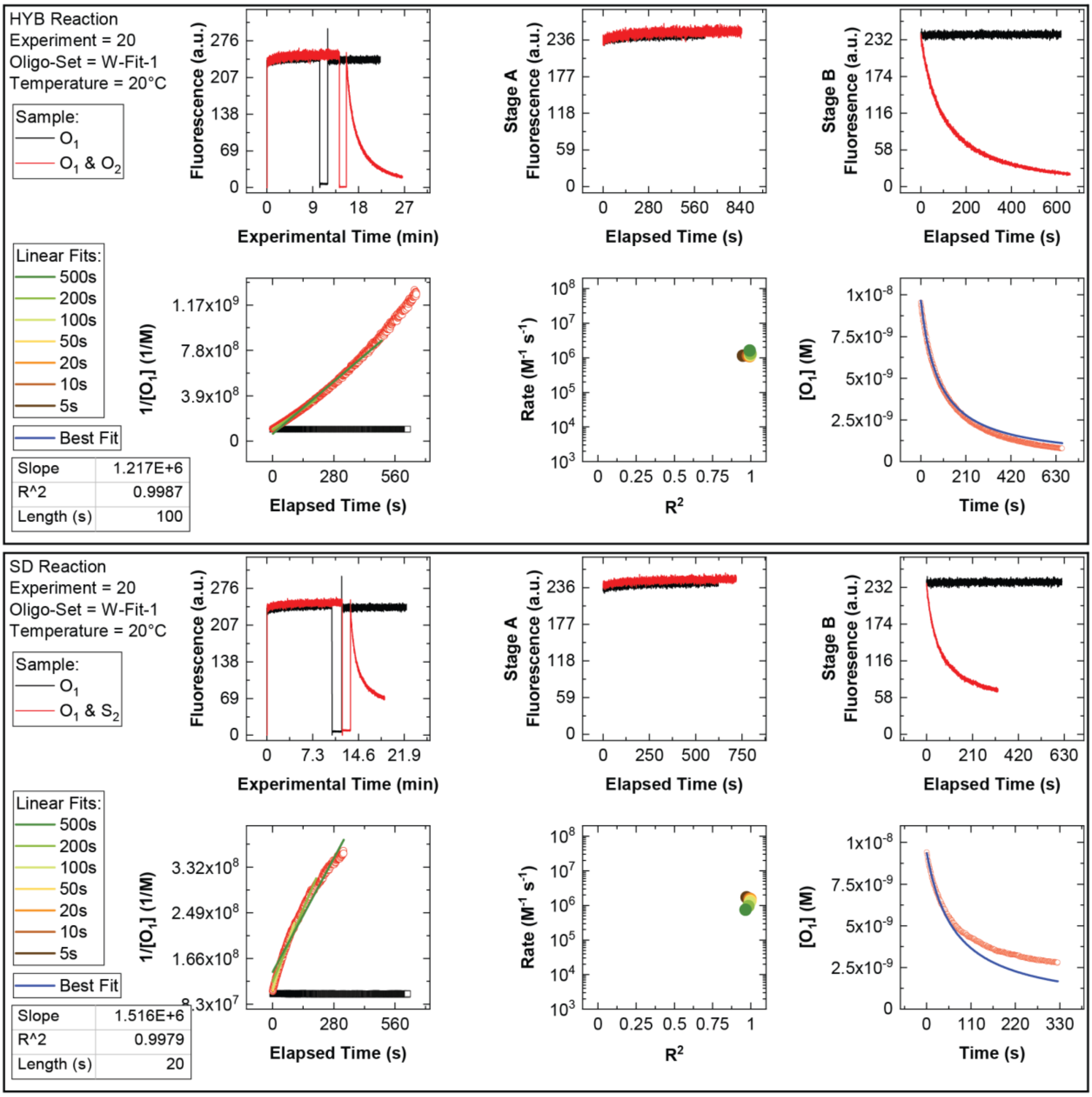


**Figure S39.** Report from experiment 20.

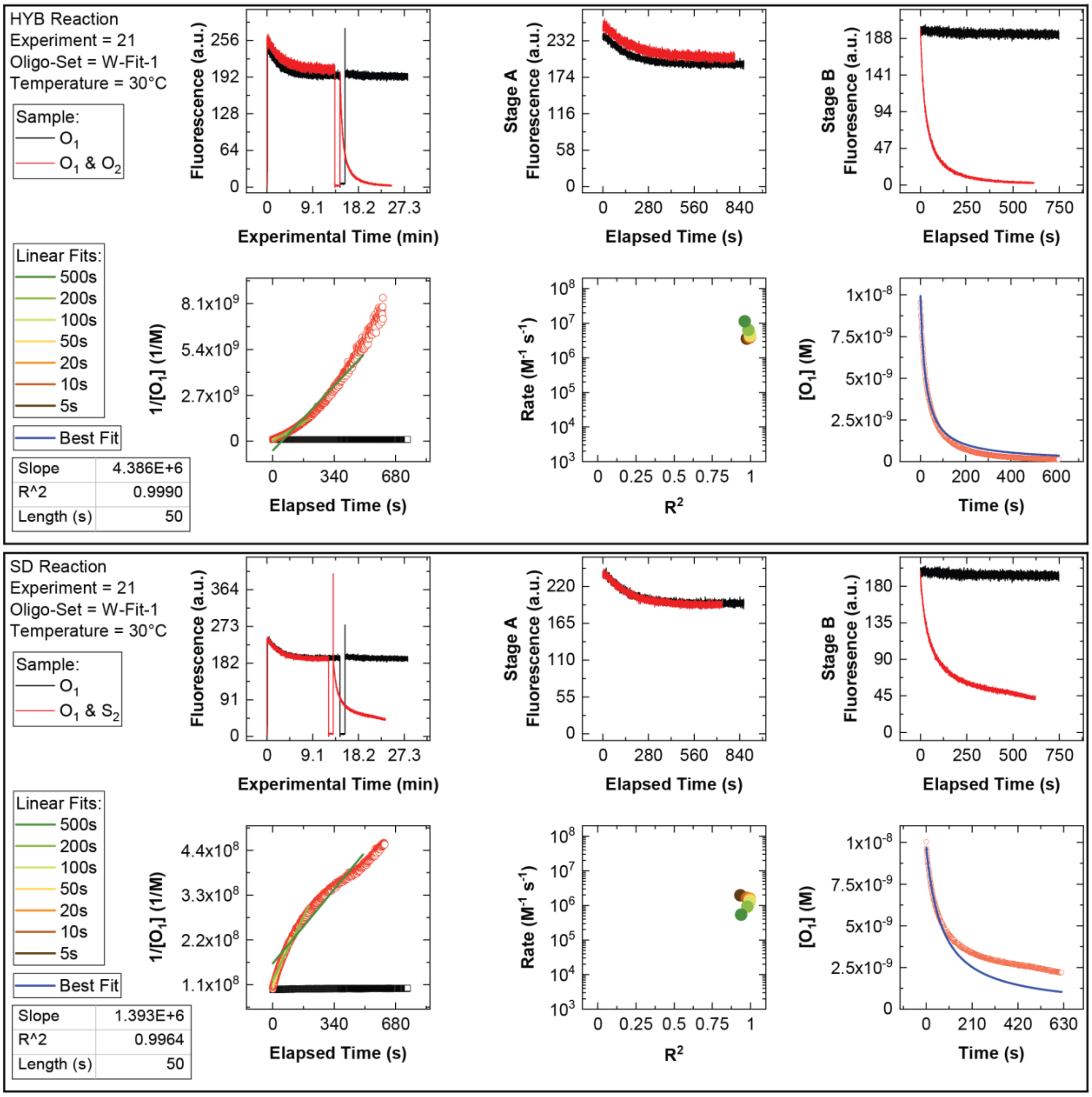


**Figure S40.** Report from experiment 21.

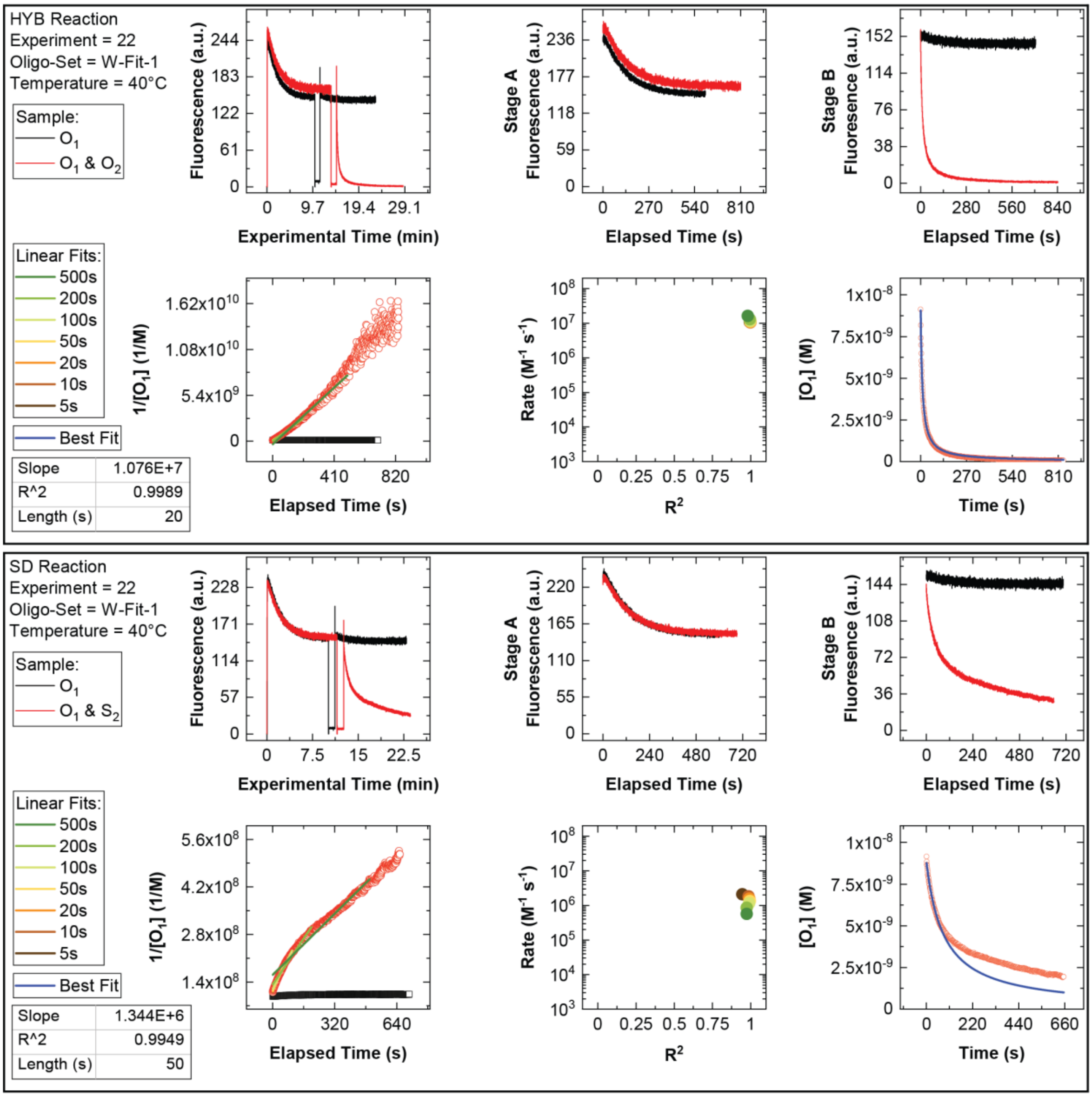


**Figure S41.** Report from experiment 22.

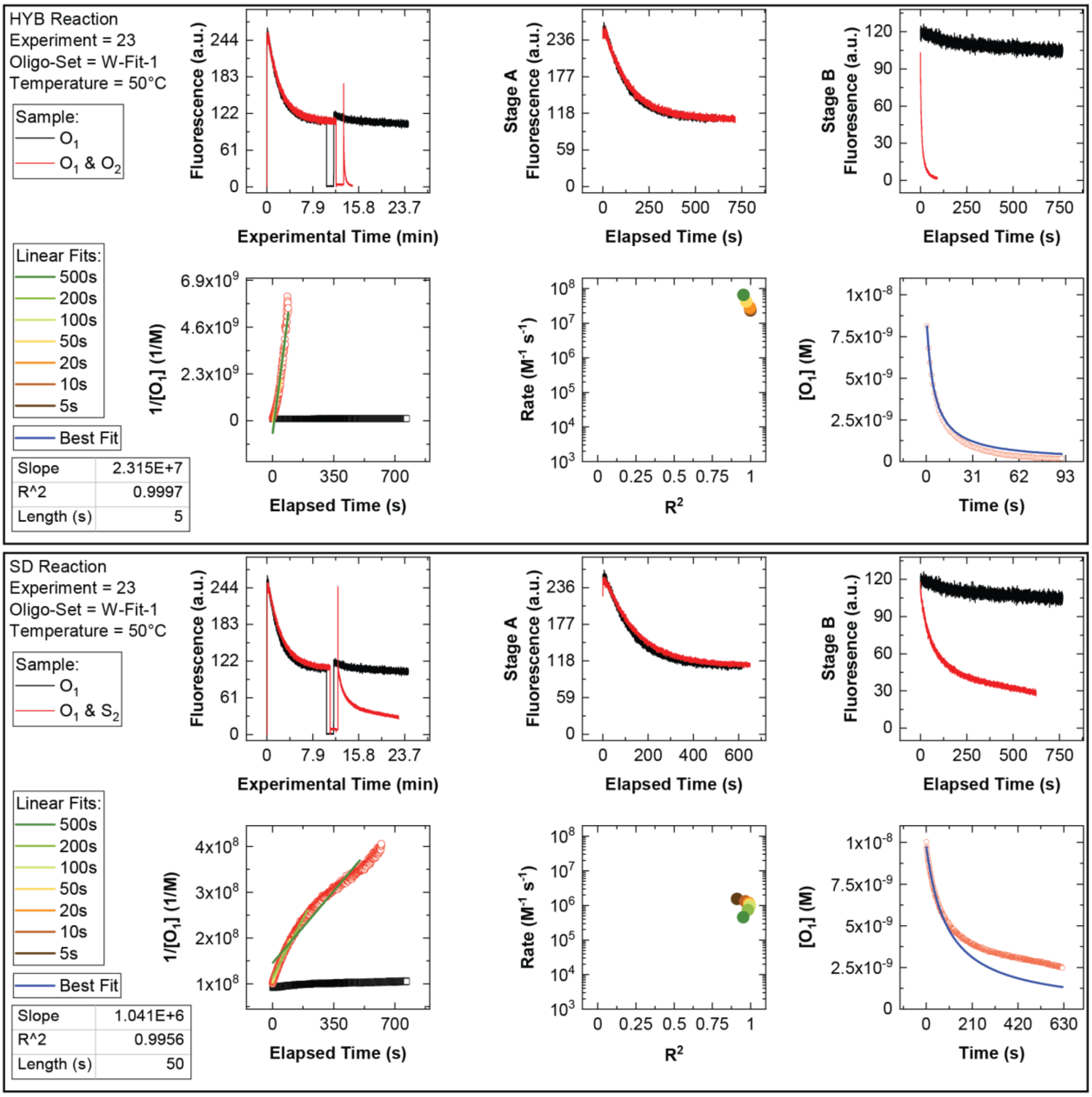


**Figure S42.** Report from experiment 23.

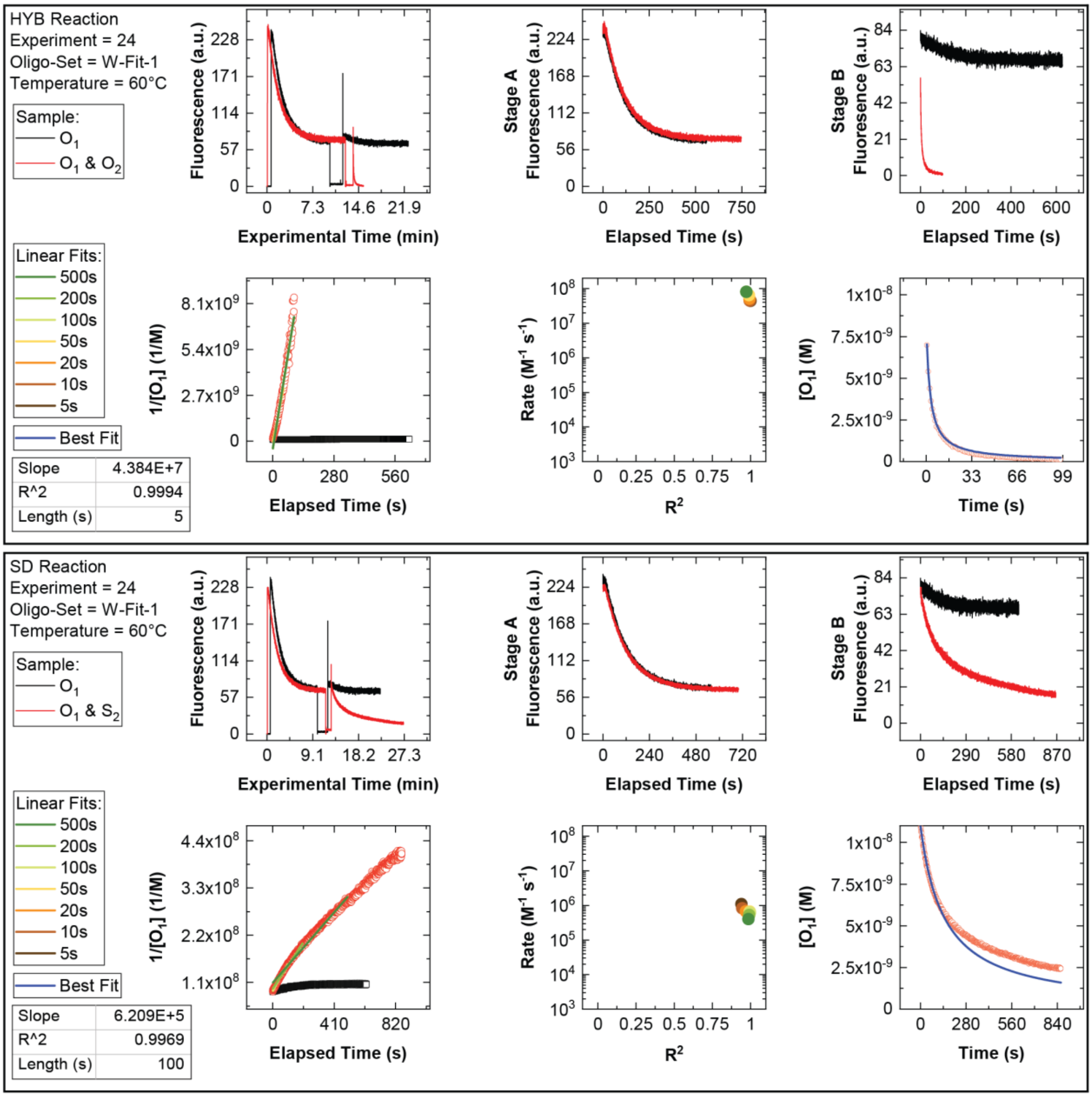


**Figure S43.** Report from experiment 24.

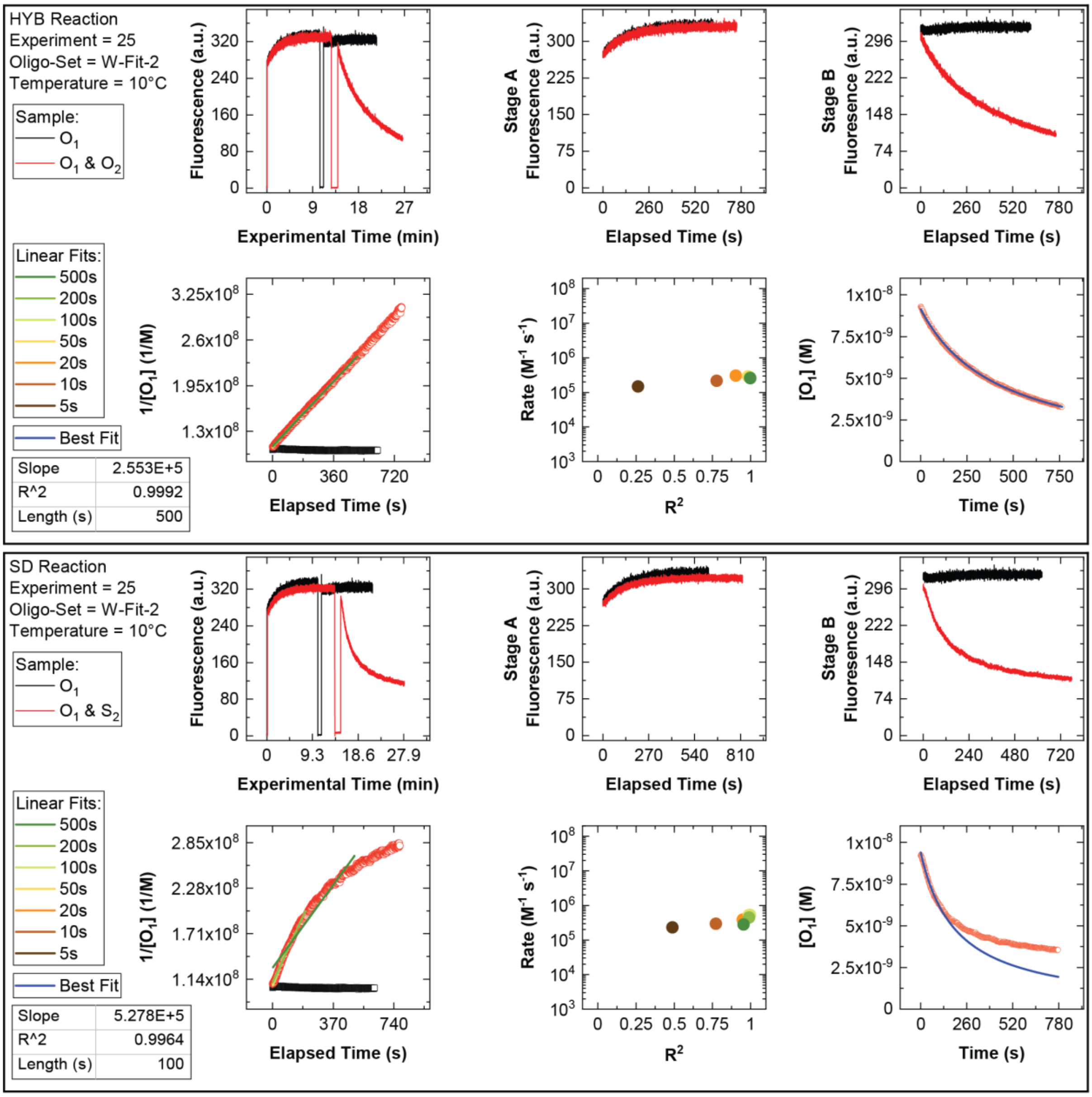


**Figure S44.** Report from experiment 25.

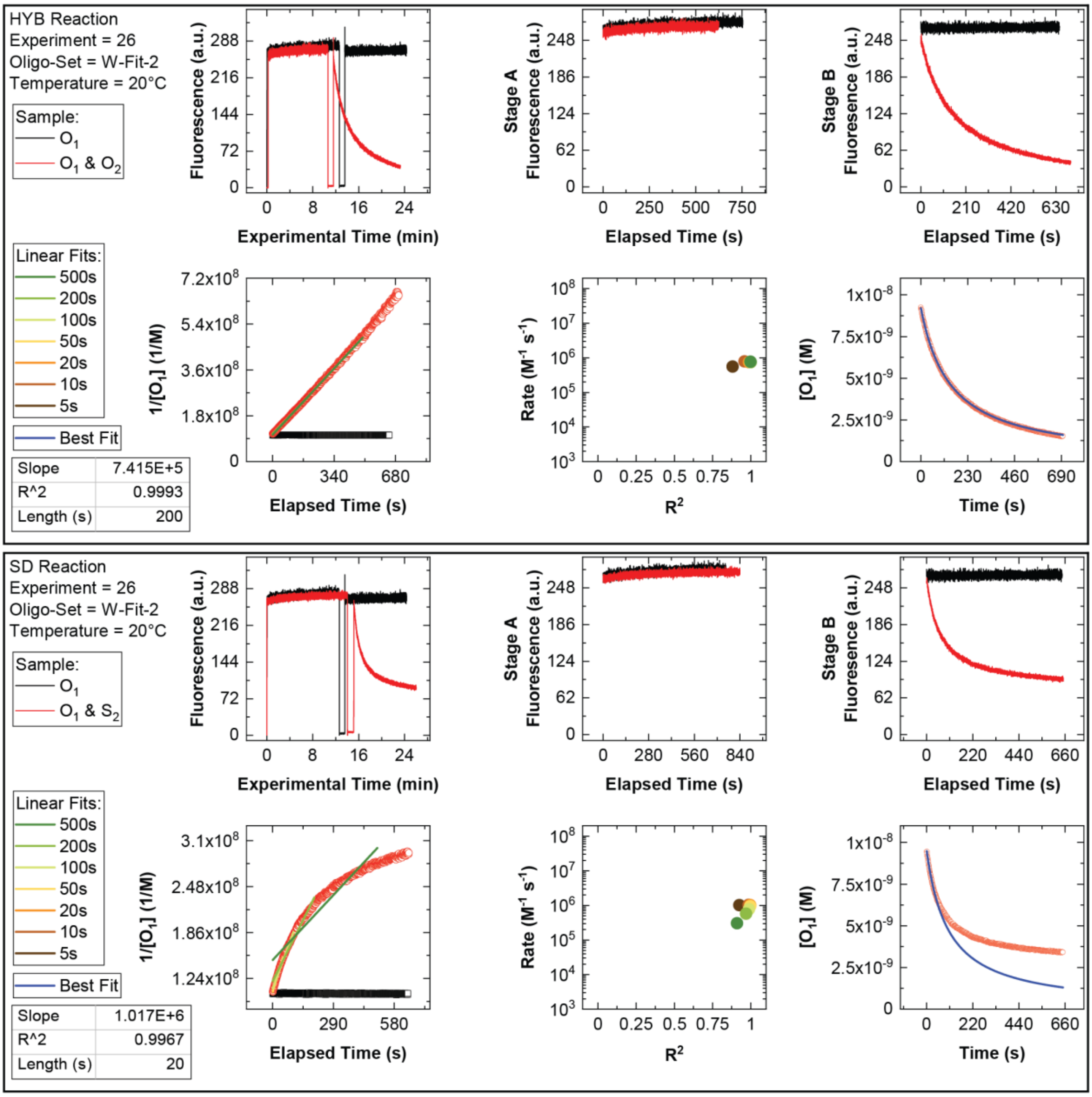


**Figure S45.** Report from experiment 26.

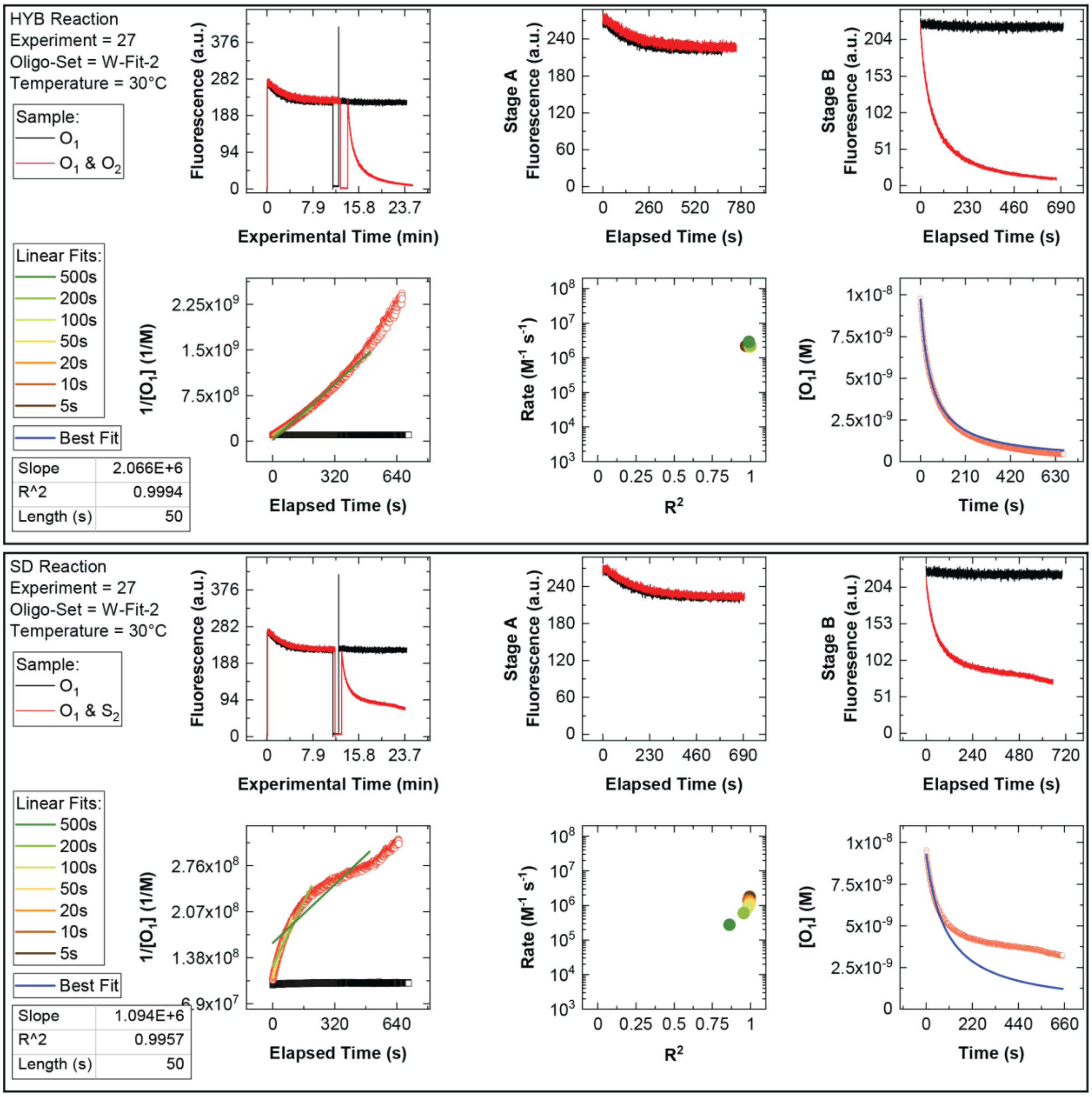


**Figure S46.** Report from experiment 27.

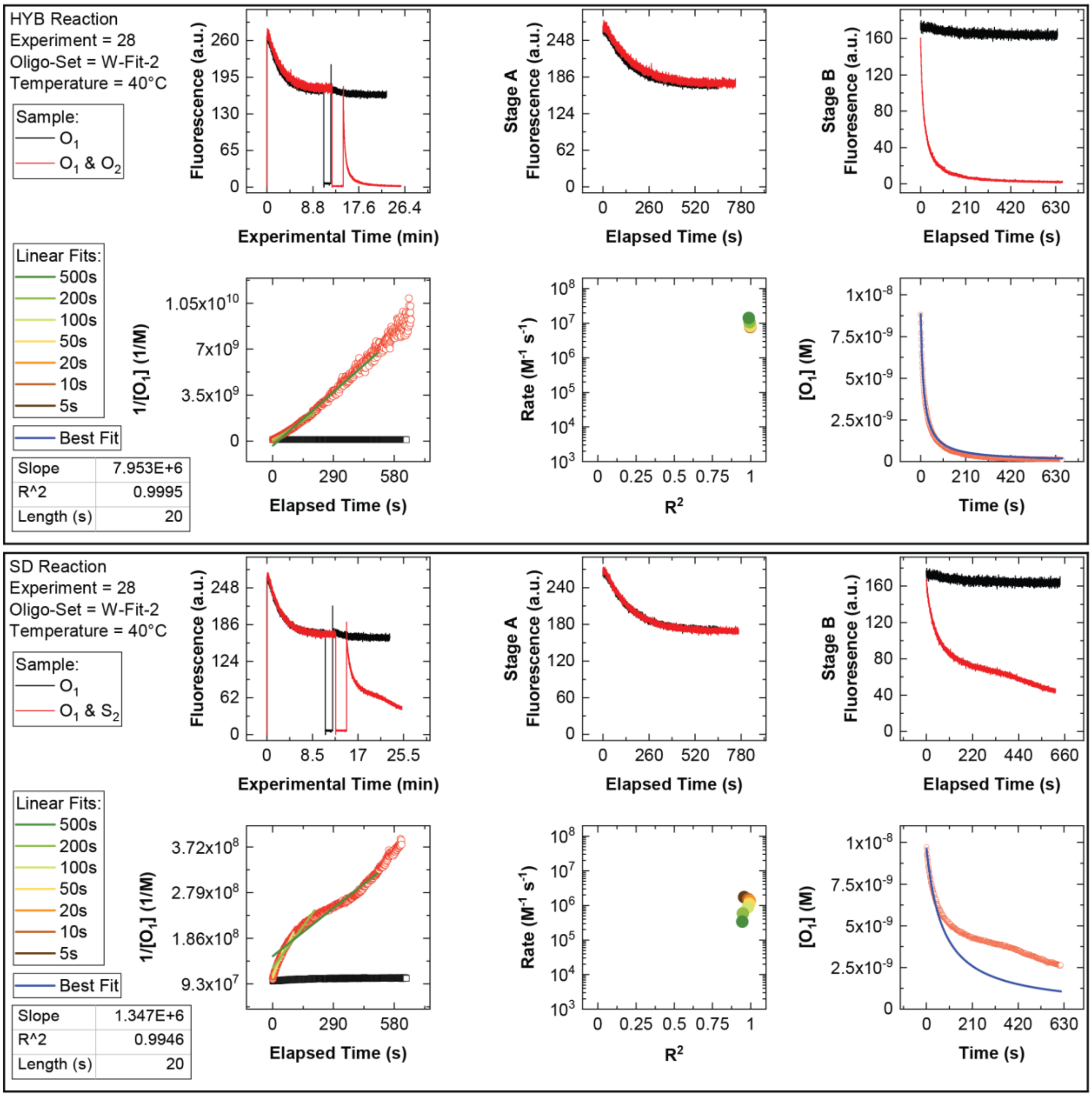


**Figure S47.** Report from experiment 28.

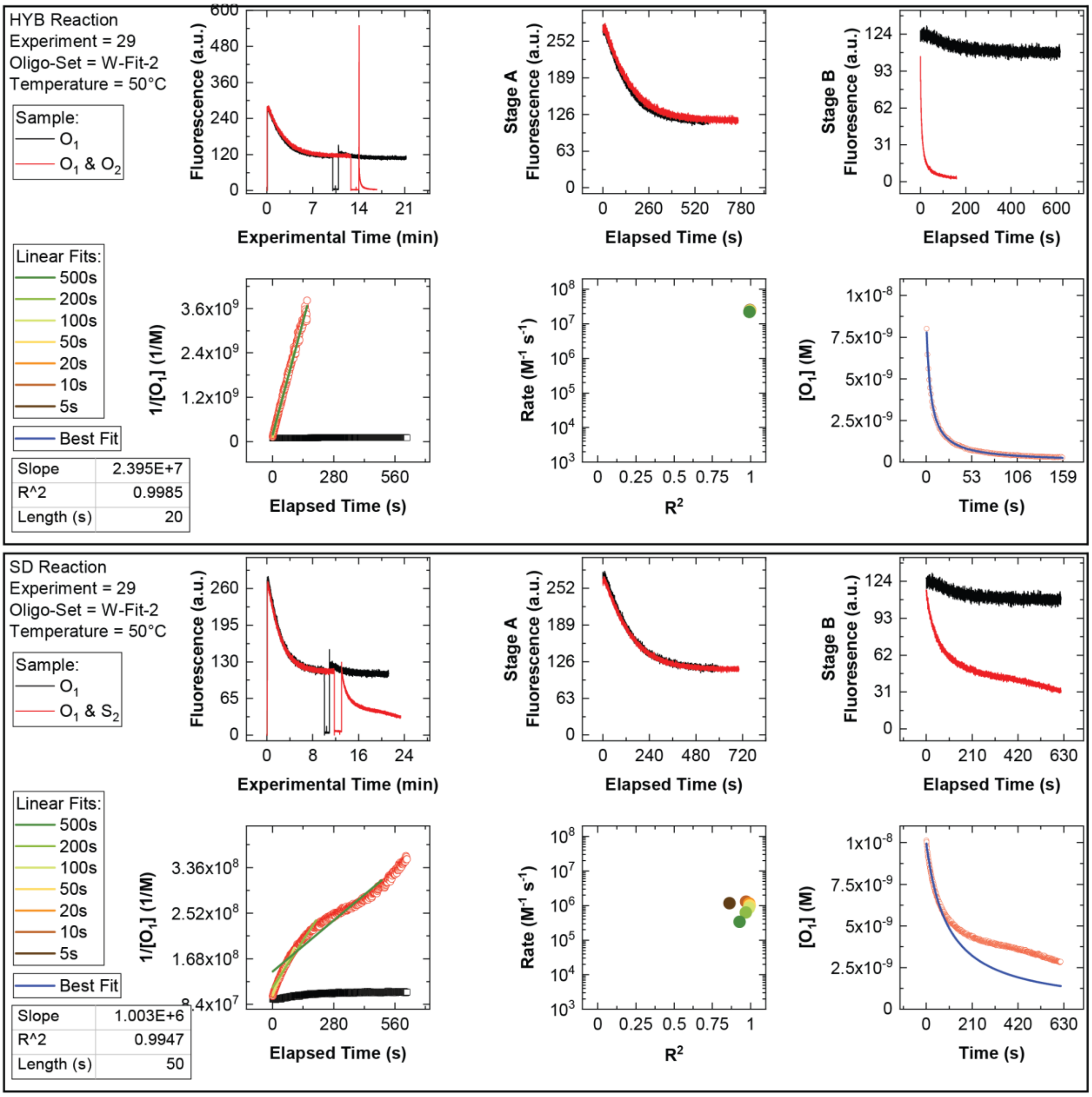


**Figure S48.** Report from experiment 29.

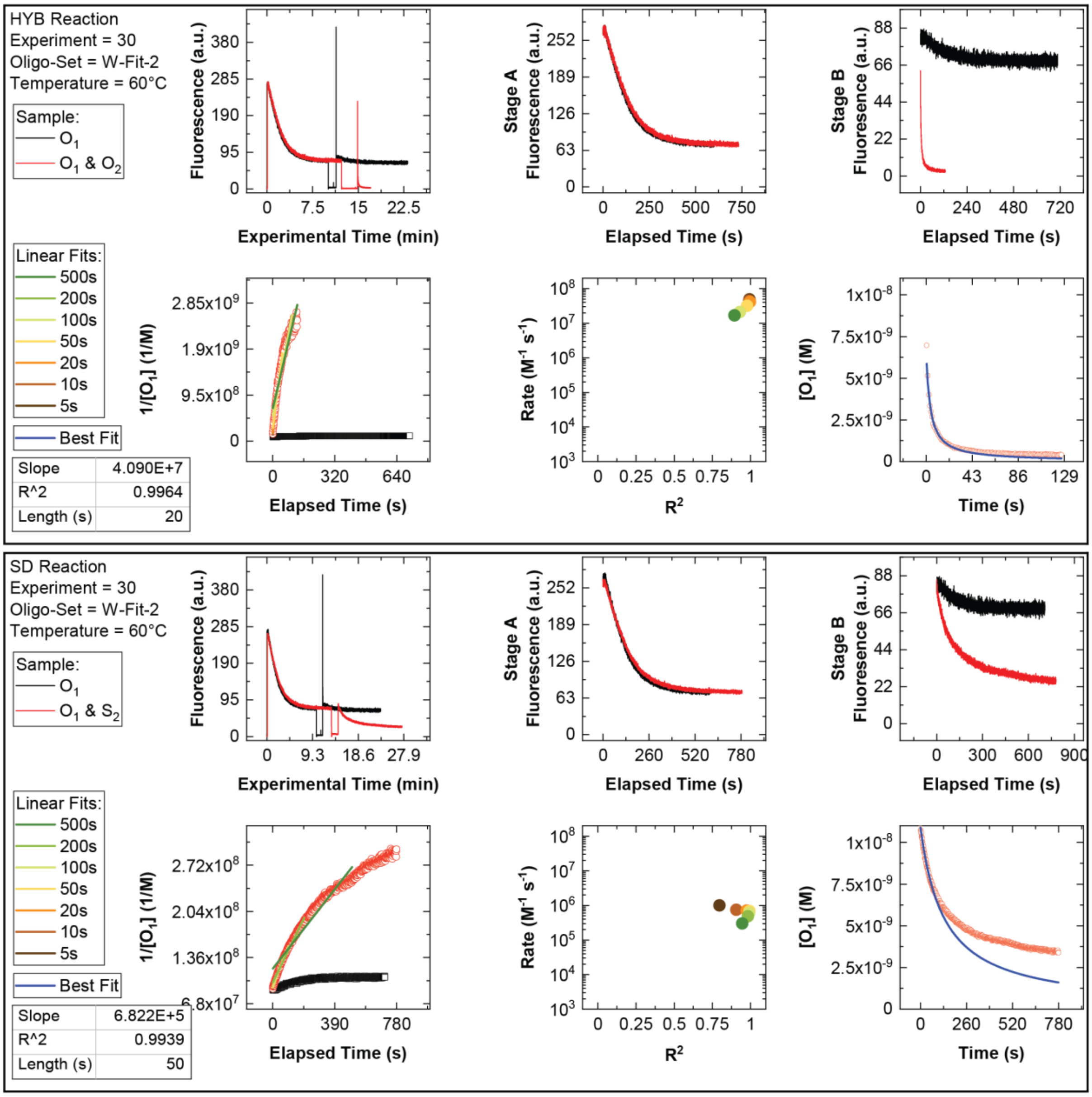


**Figure S49.** Report from experiment 30.

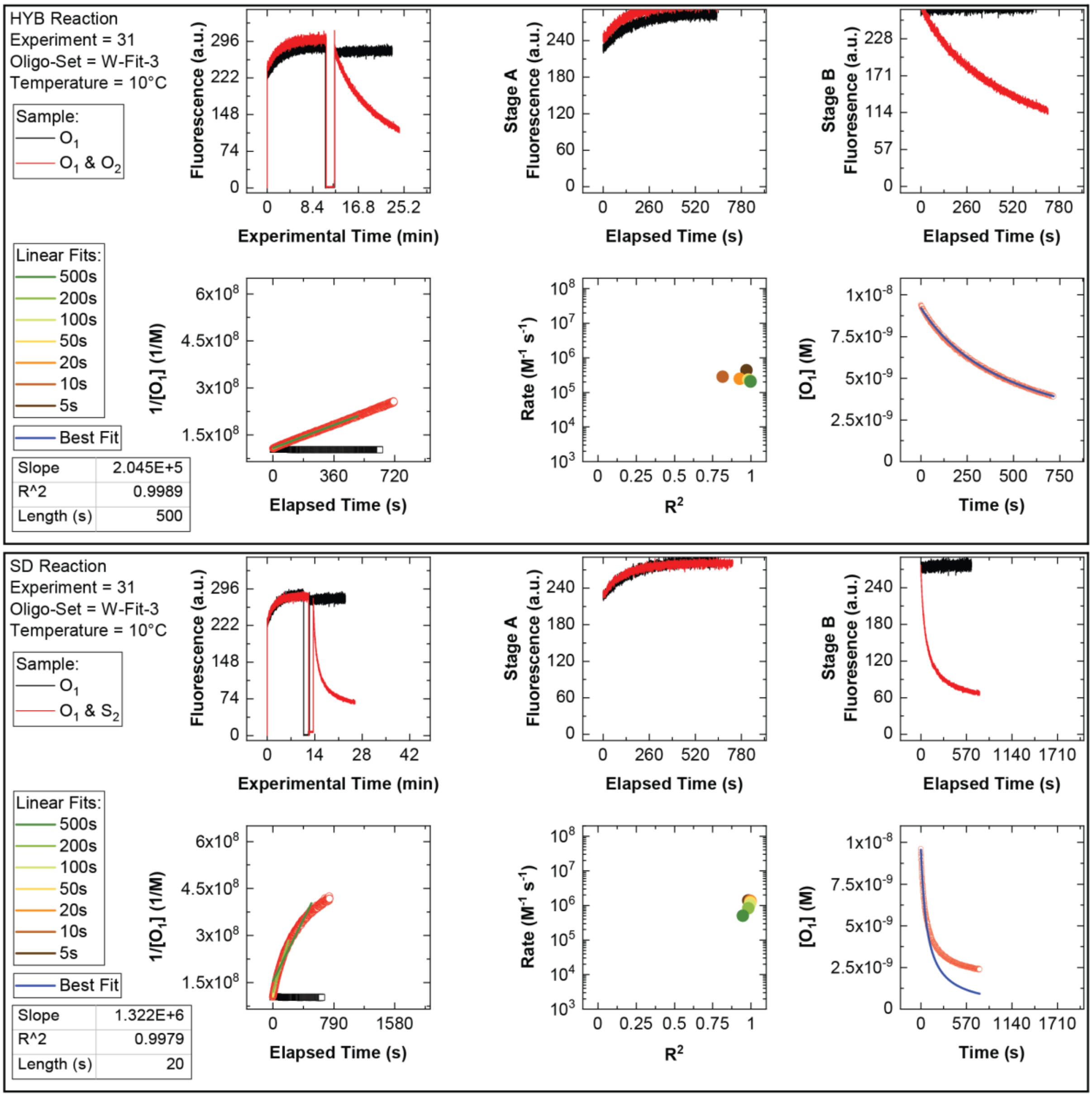


**Figure S50.** Report from experiment 31.

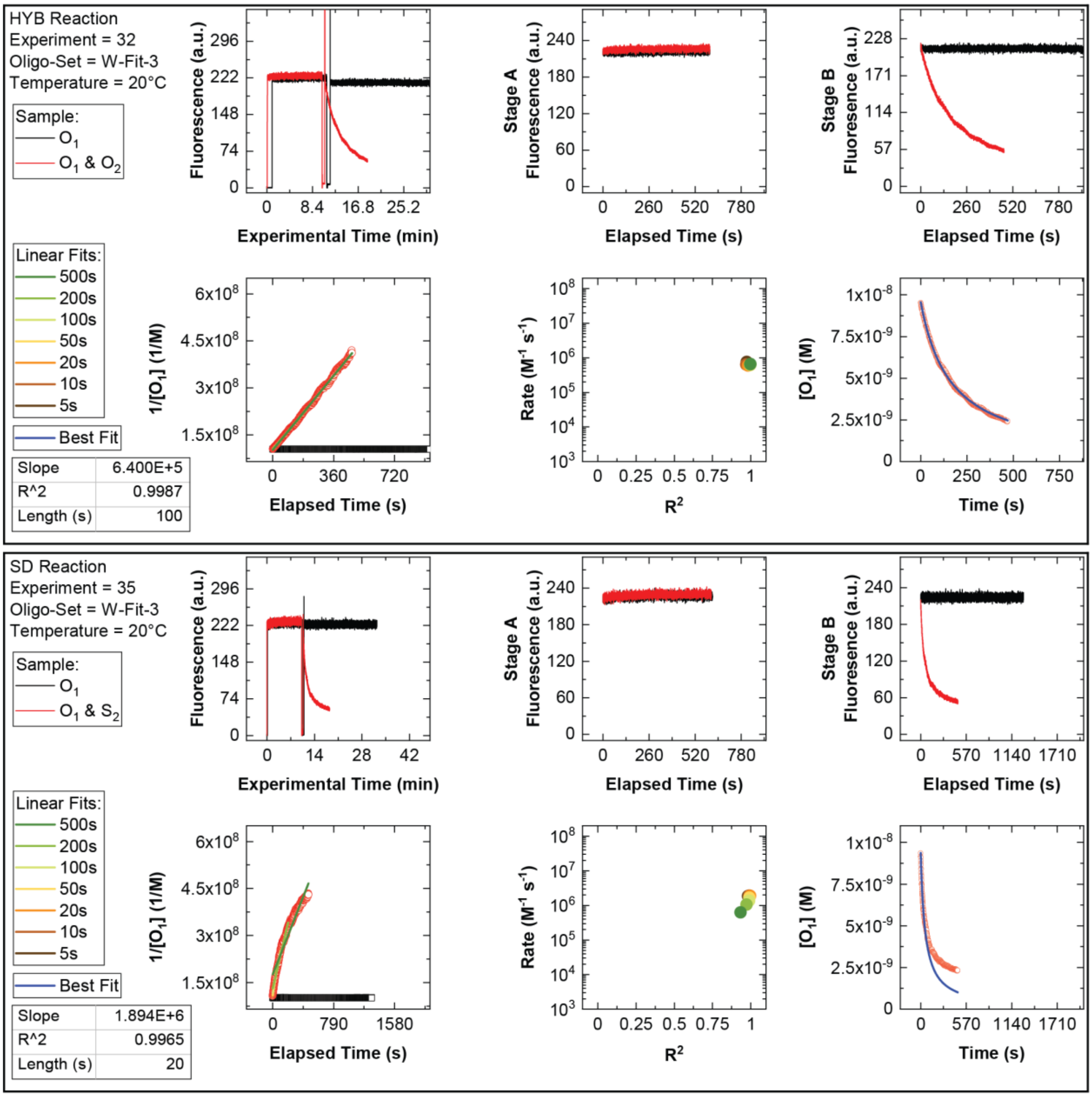


**Figure S51.** Report from experiments 32 and 35.

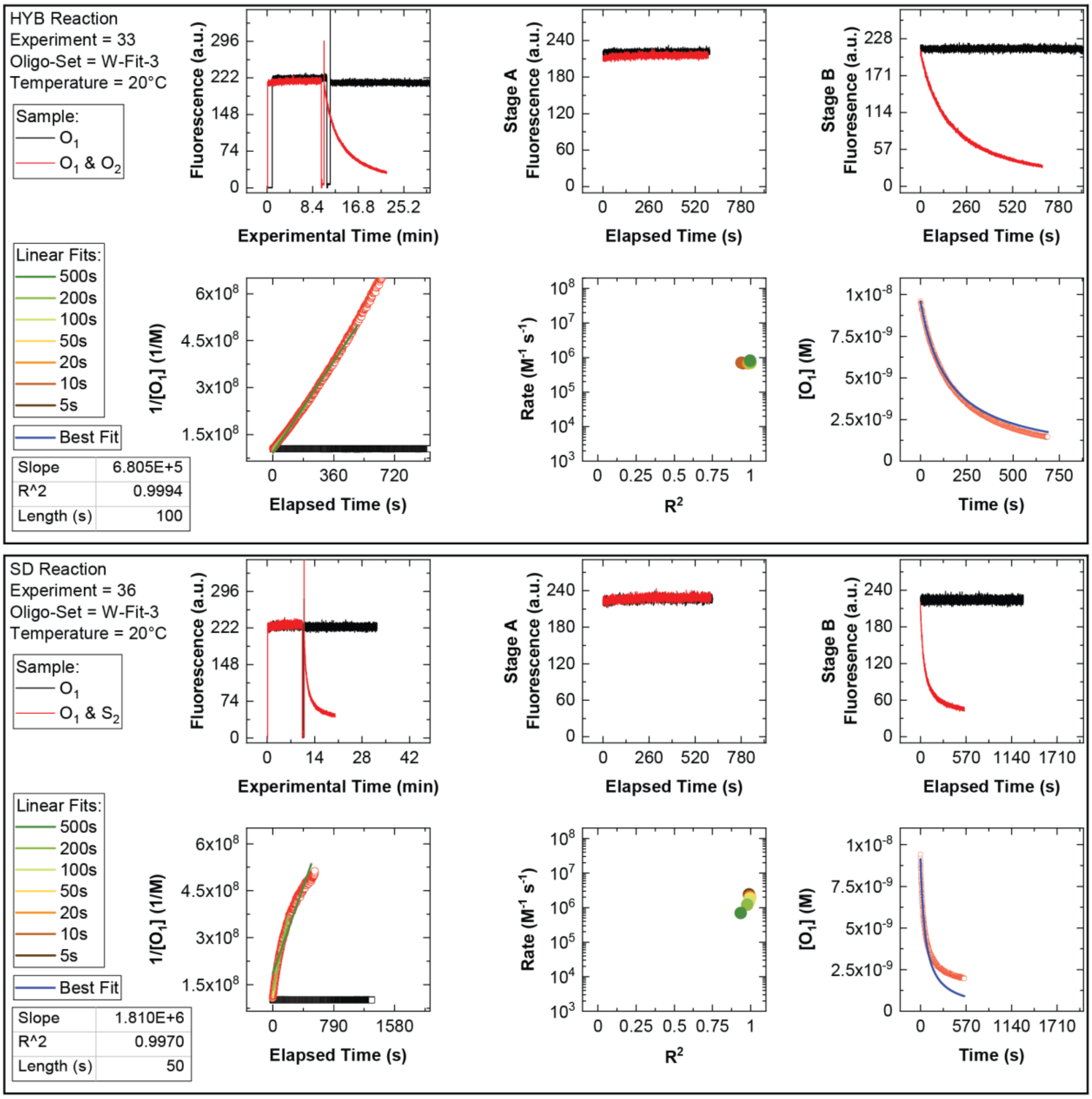


**Figure S52.** Report from experiments 33 and 36.

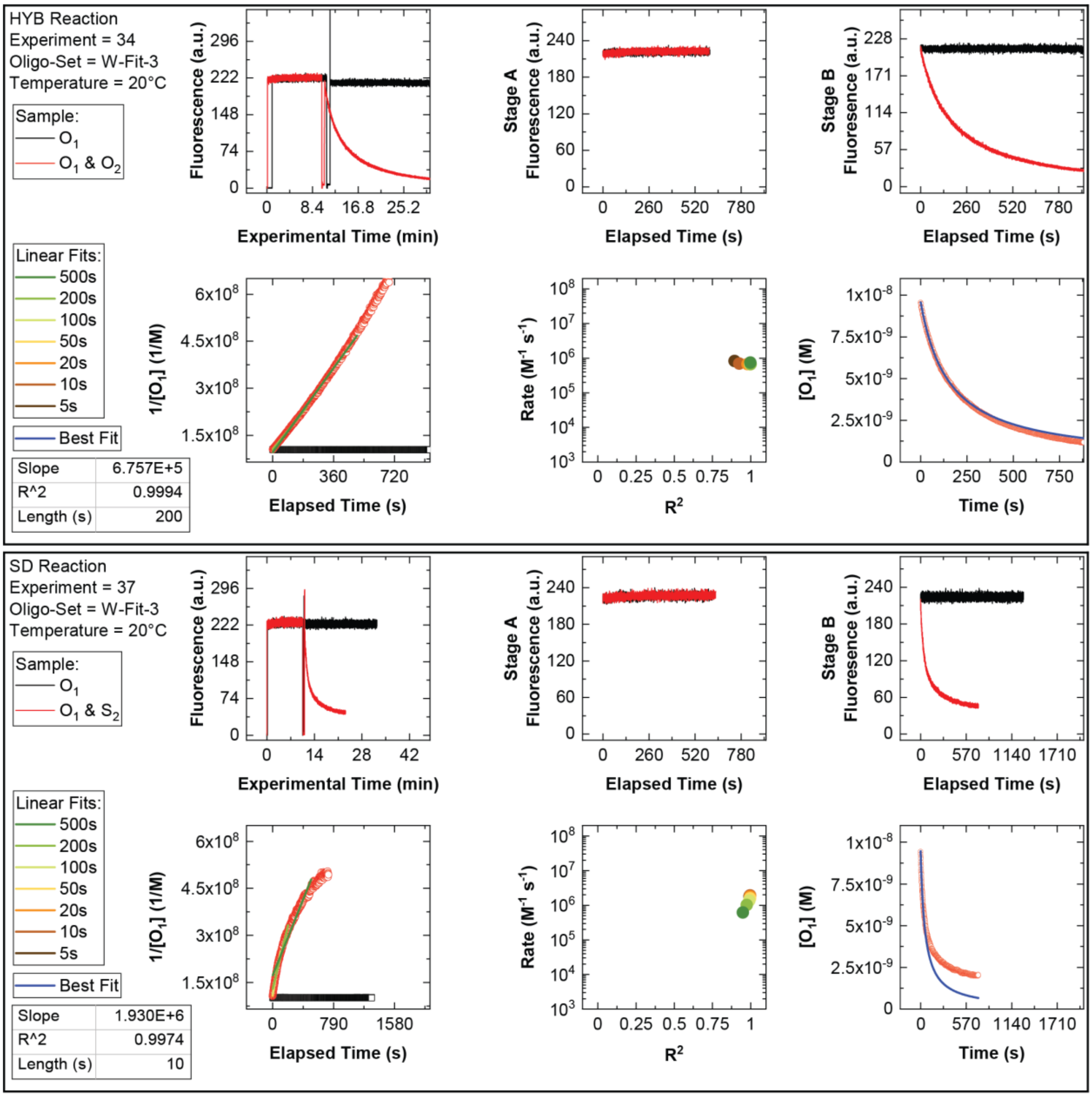


**Figure S53.** Report from experiments 34 and 37.

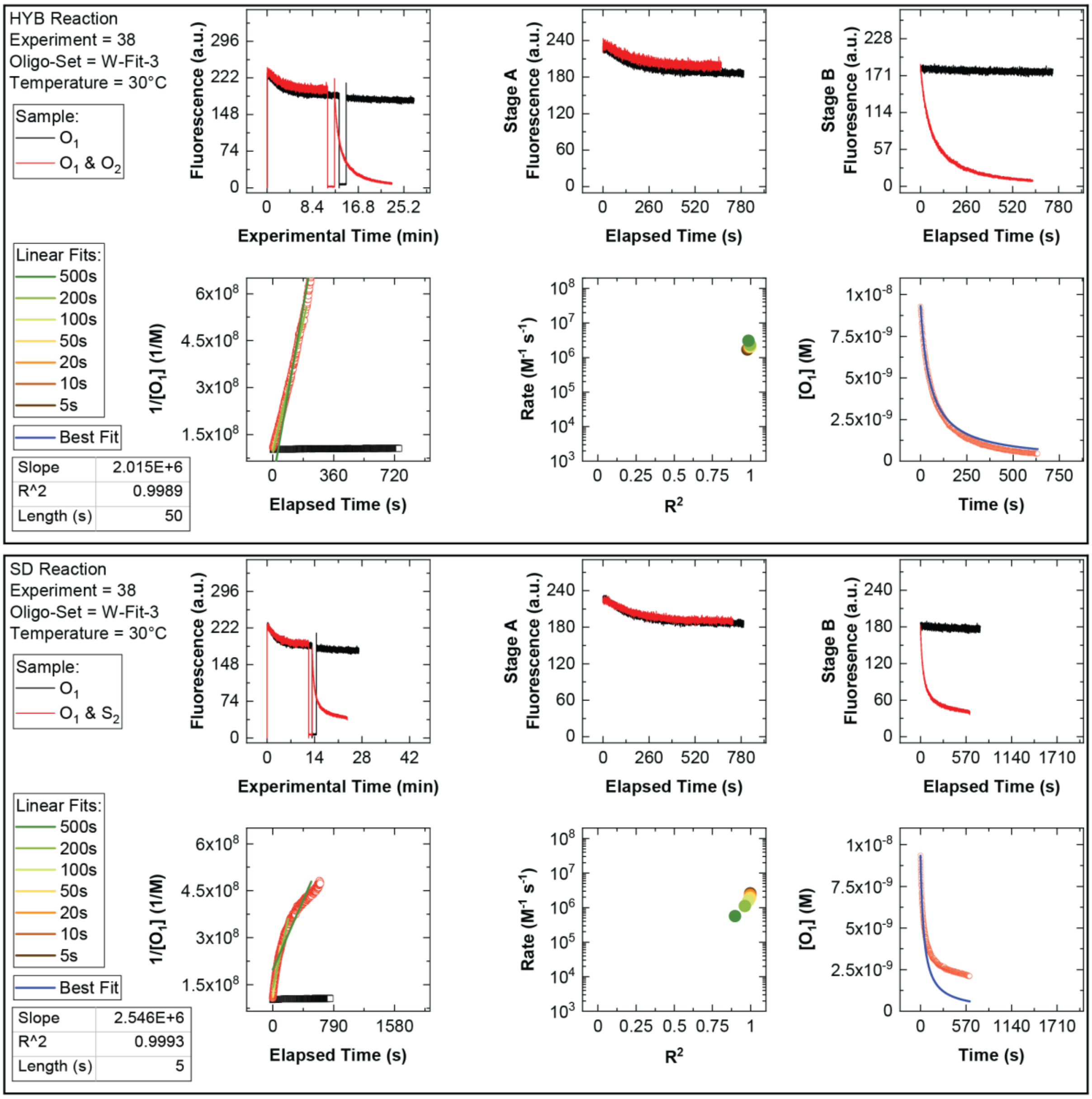


**Figure S54.** Report from experiment 38.

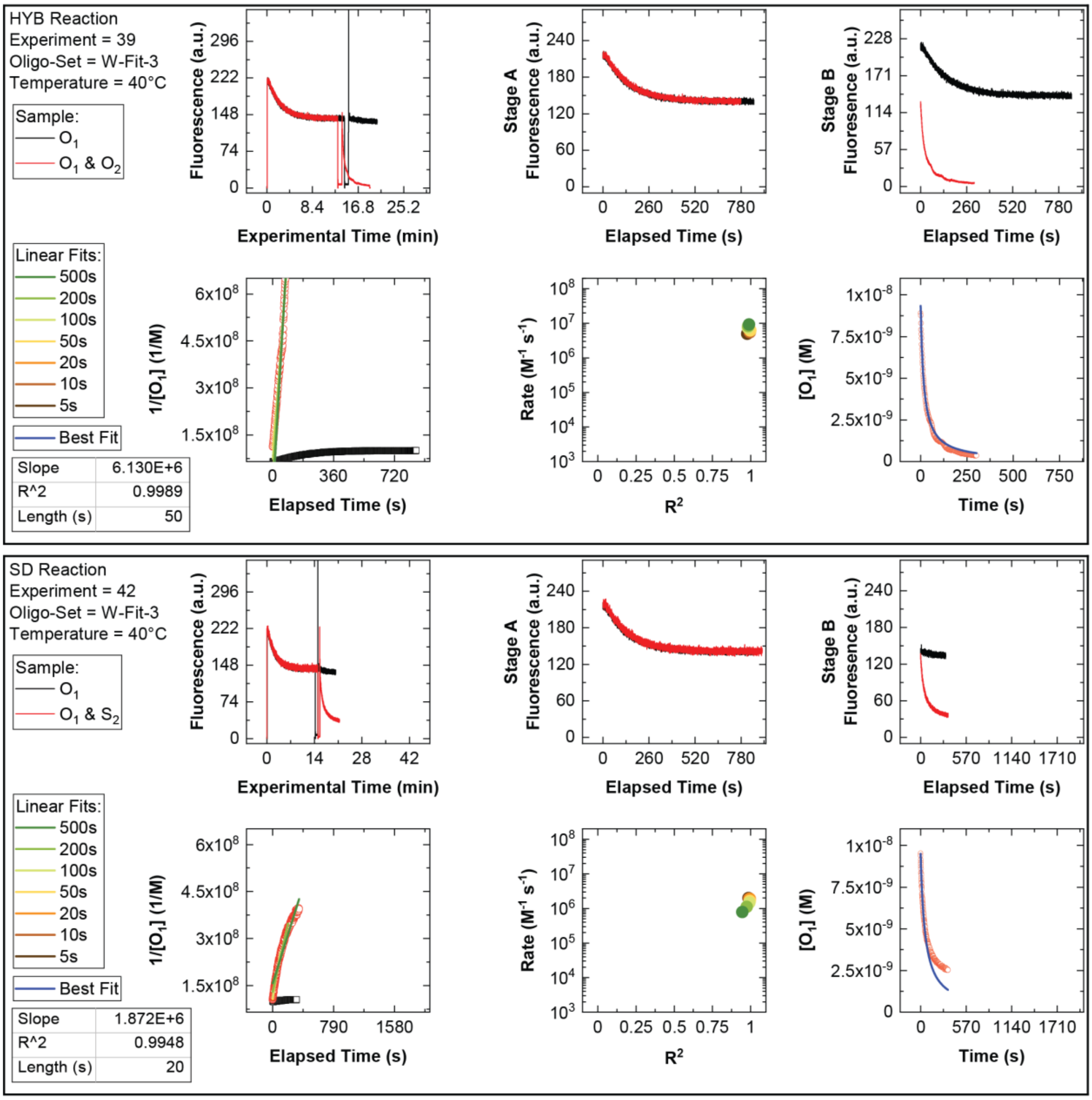


**Figure S55.** Report from experiments 39 and 42.

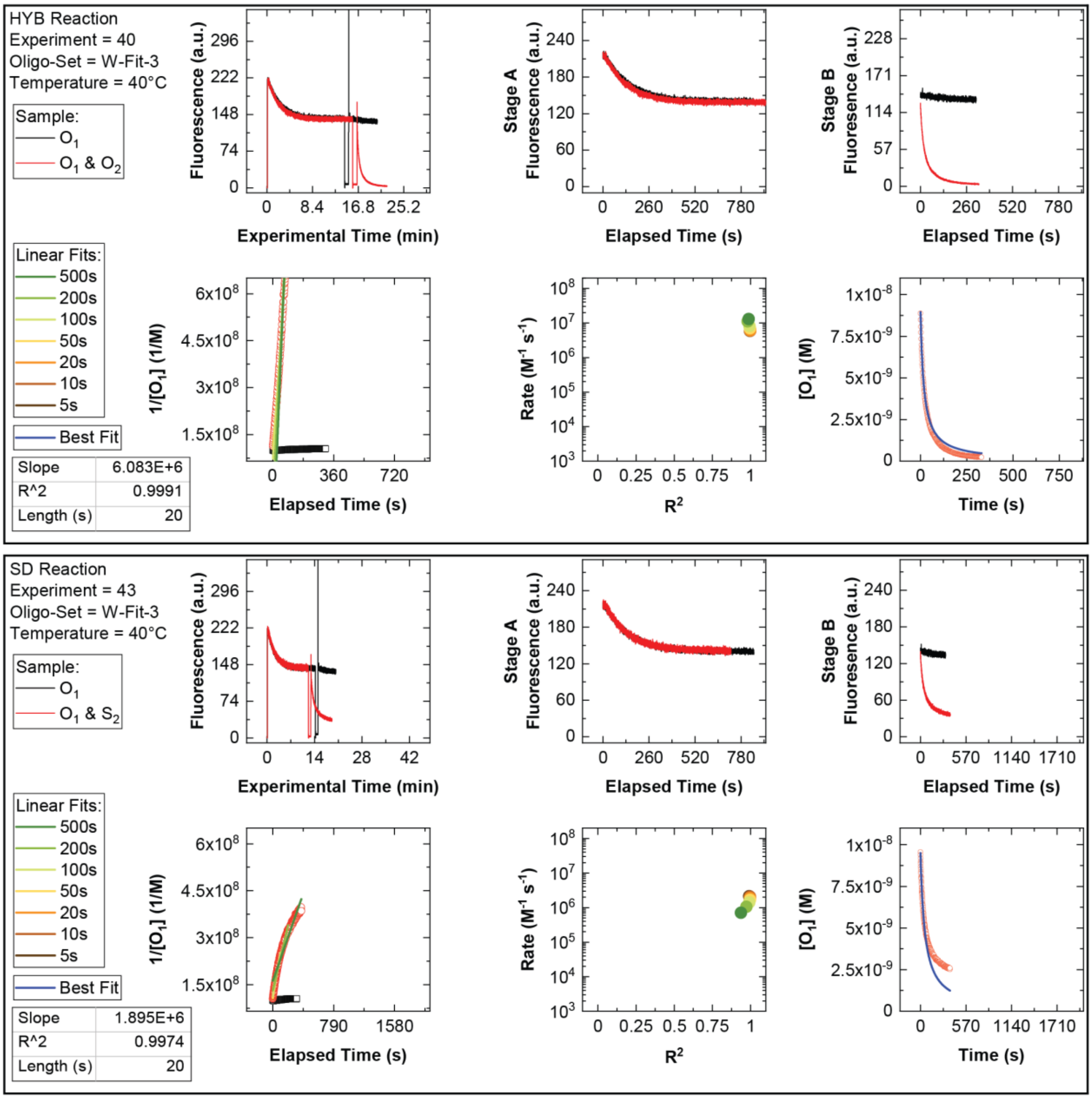


**Figure S56.** Report from experiments 40 and 43.

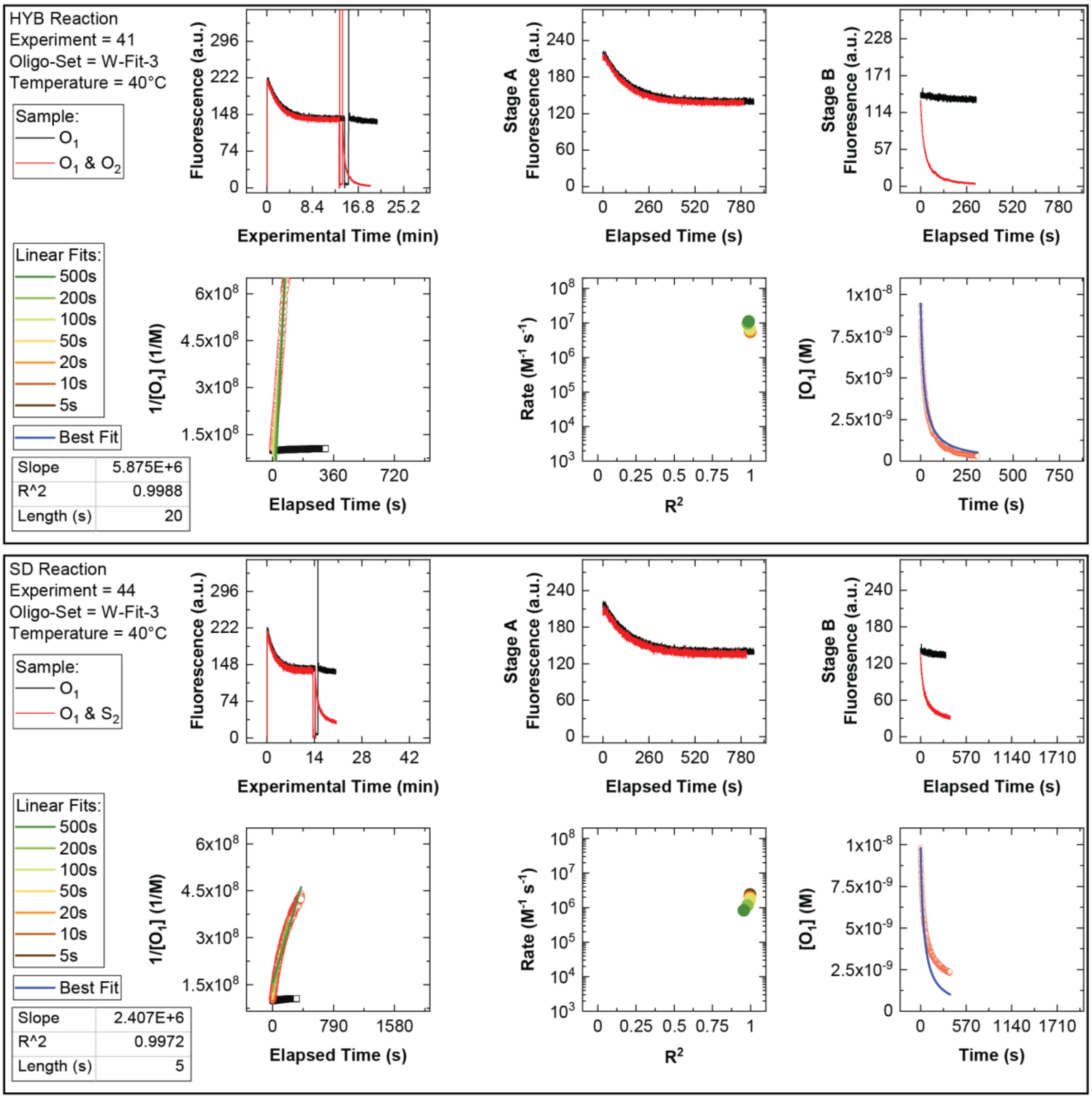


**Figure S57.** Report from experiments 41 and 44.

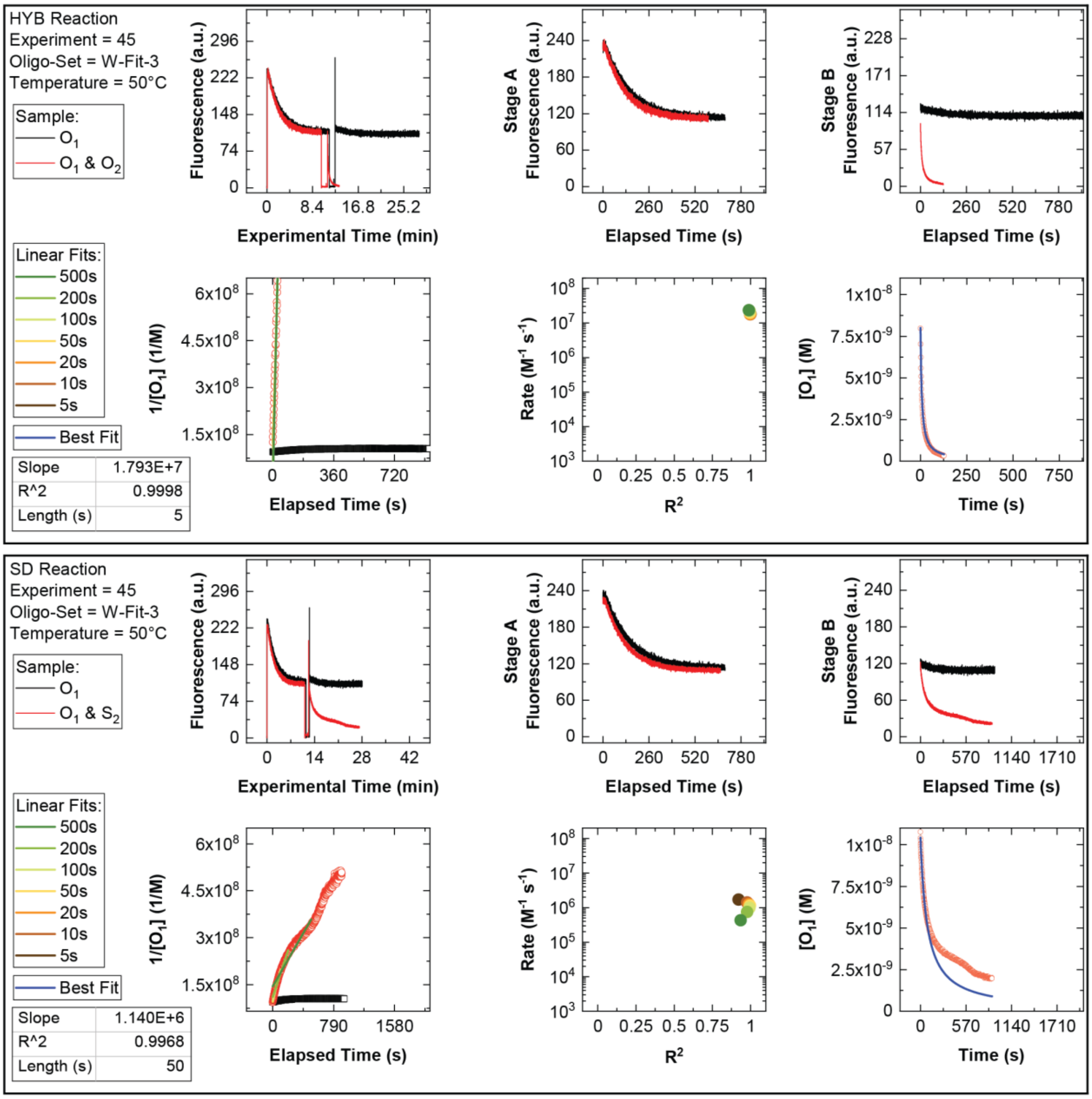


**Figure S58.** Report from experiment 45.

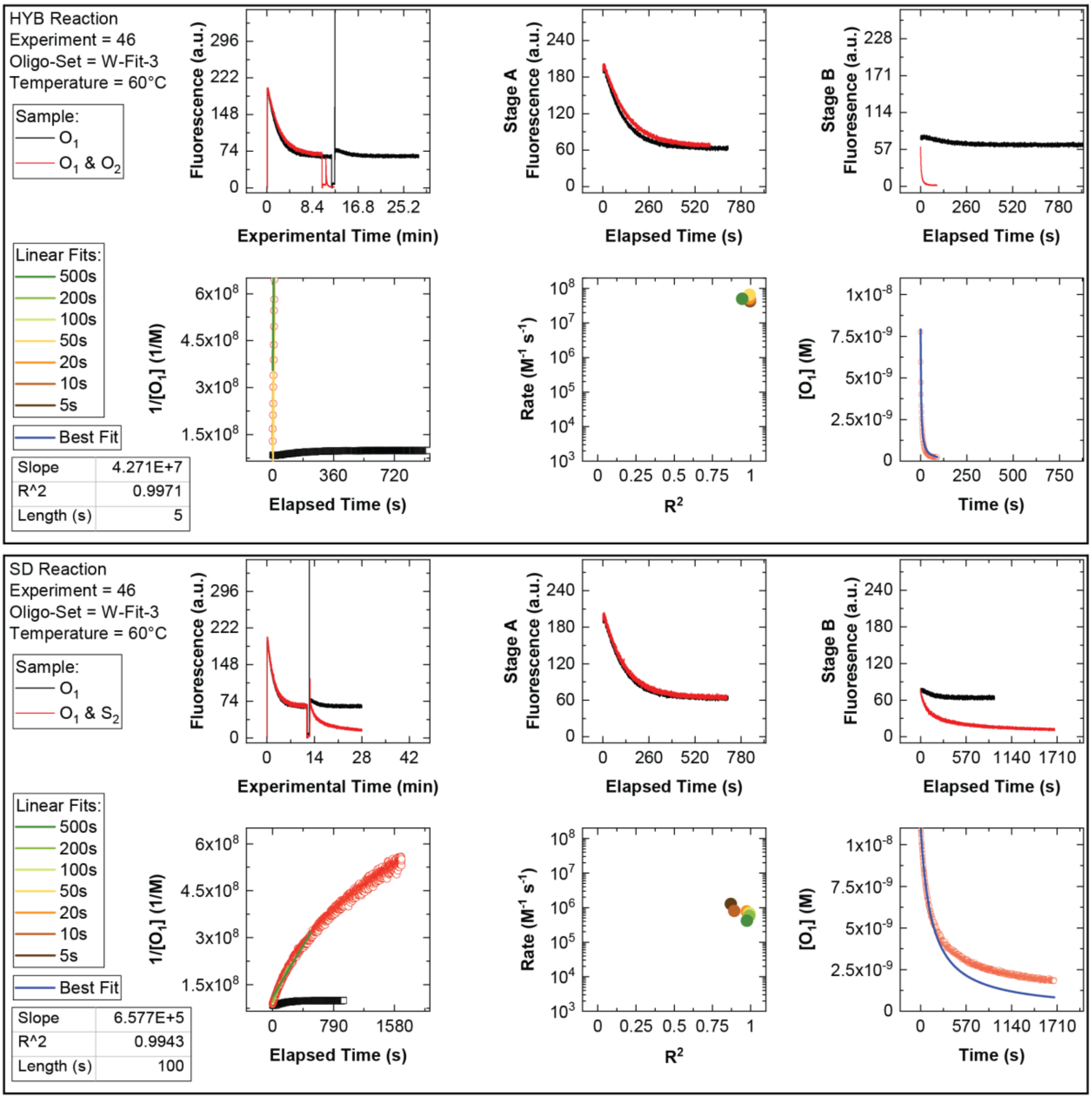


**Figure S59.** Report from experiment 46.

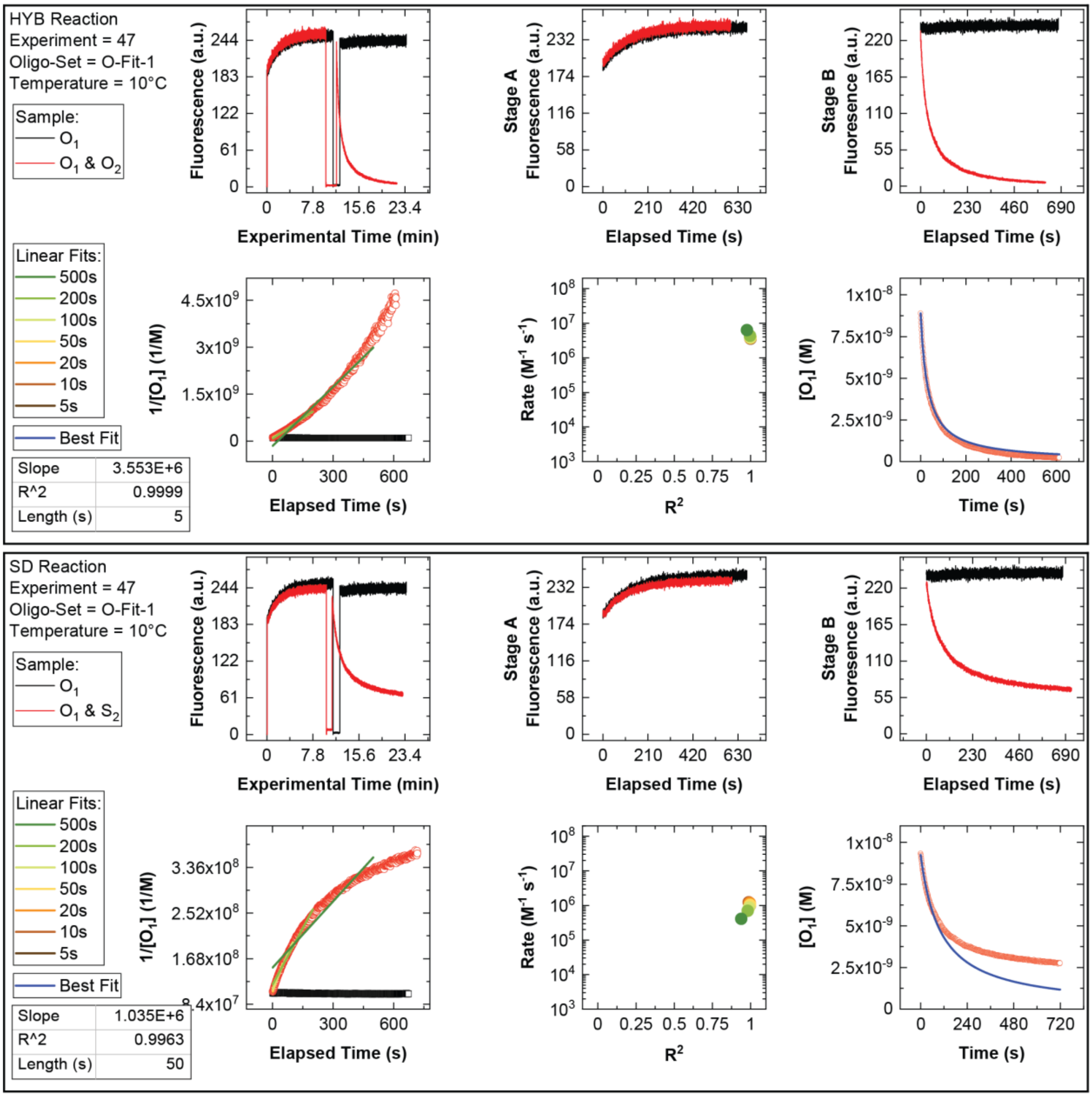


**Figure S60.** Report from experiment 47.

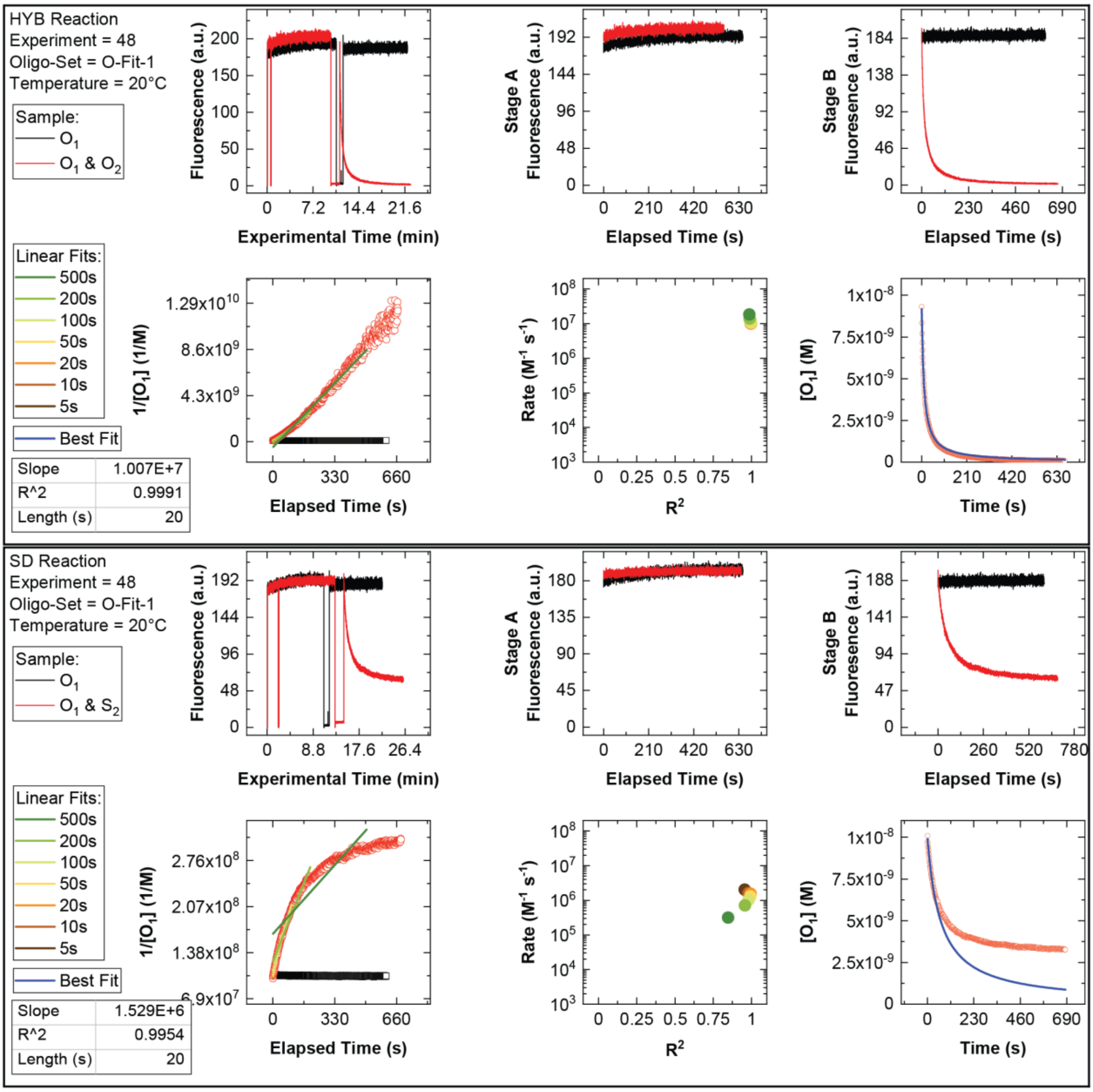


**Figure S61.** Report from experiment 48.

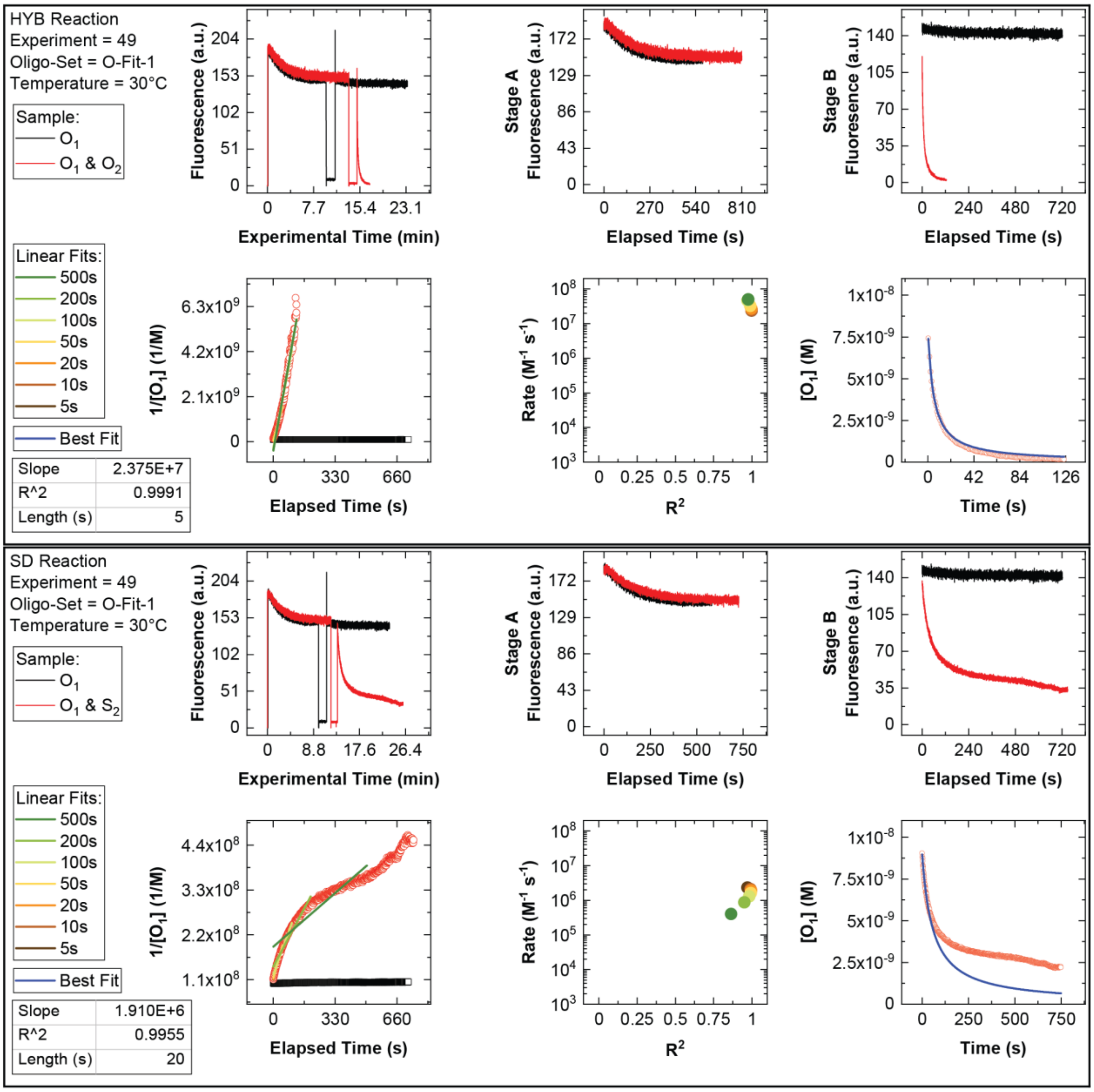


**Figure S62.** Report from experiment 49.

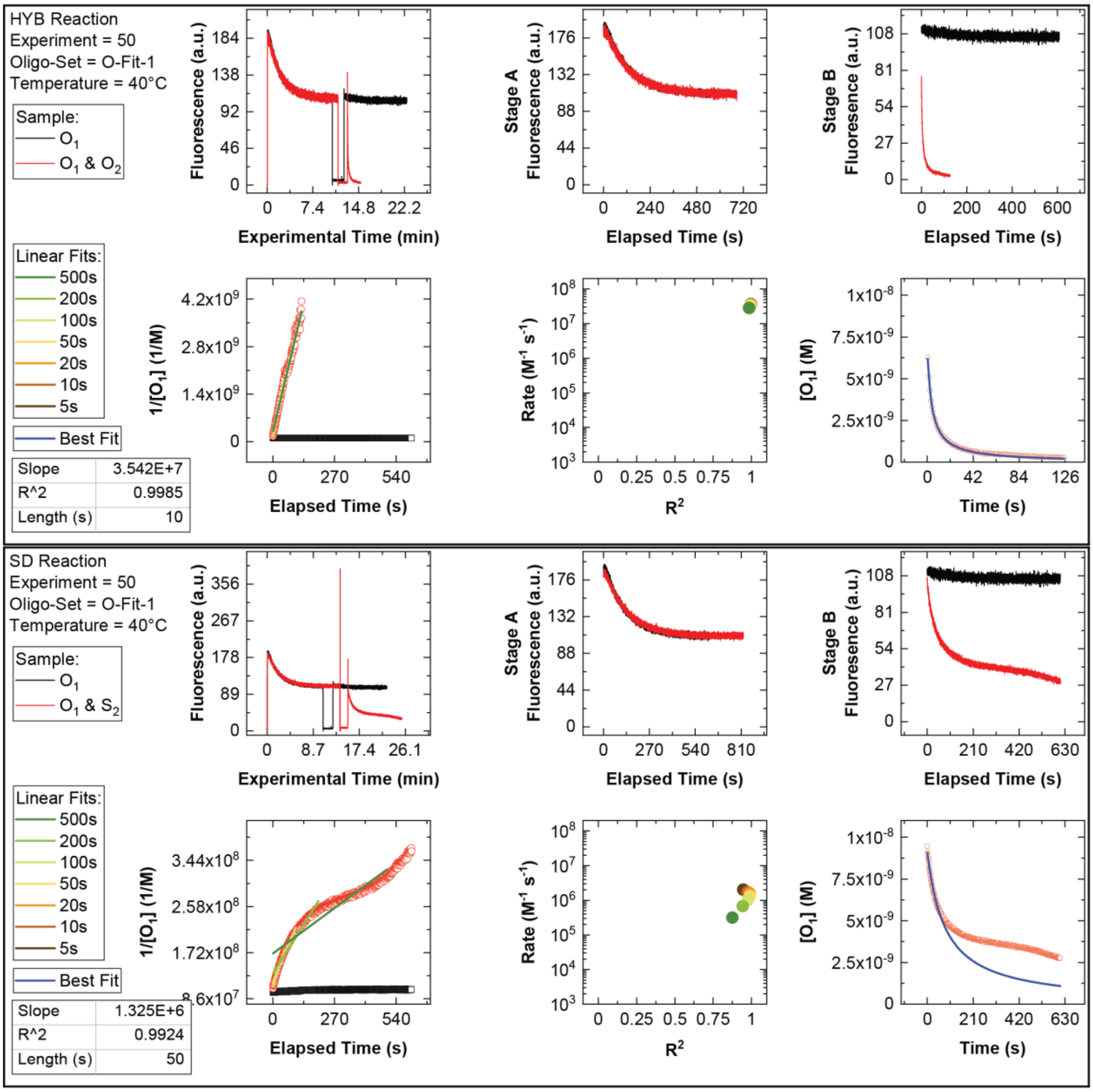


**Figure S63.** Report from experiment 50.

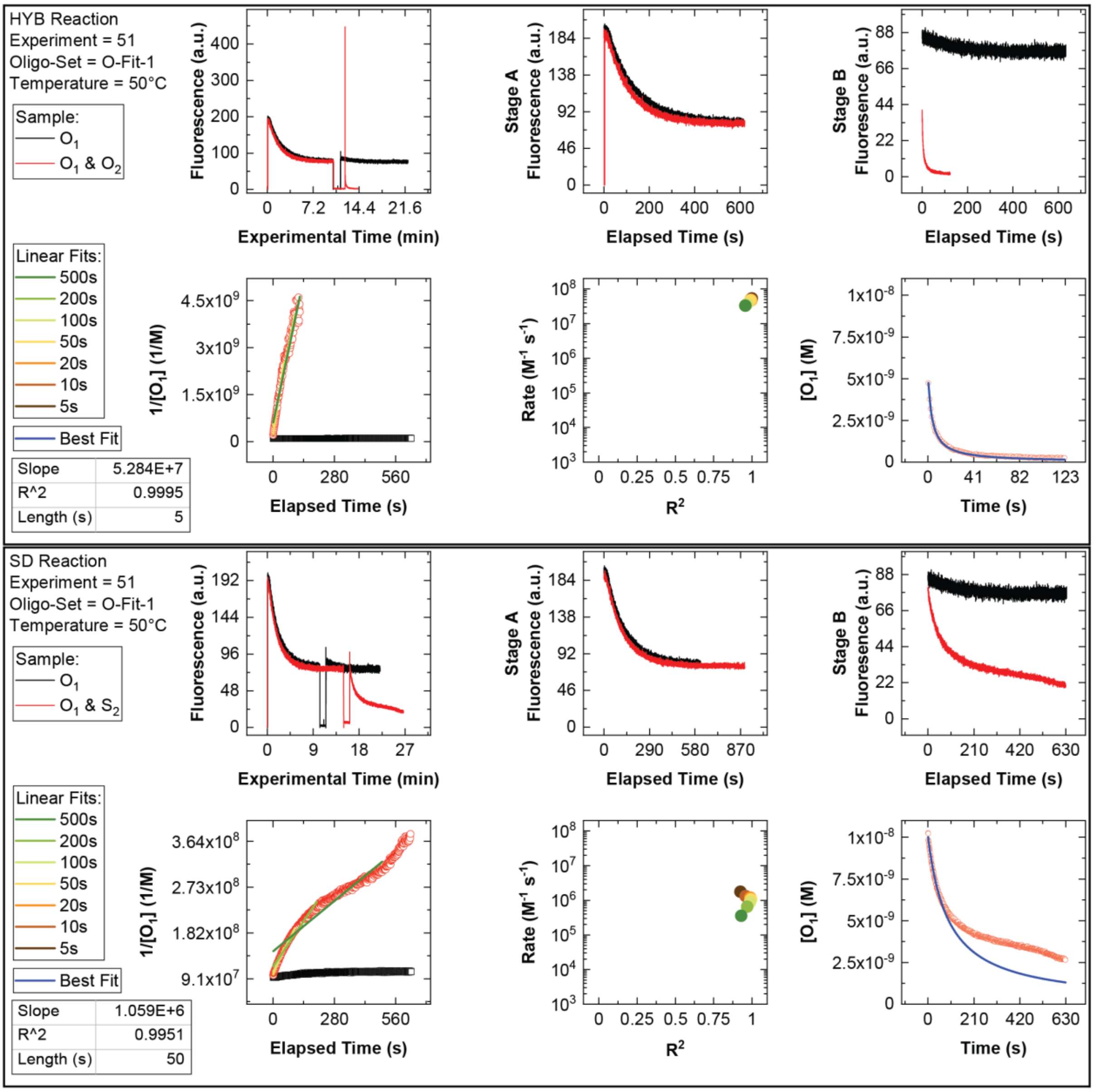


**Figure S64.** Report from experiment 51.

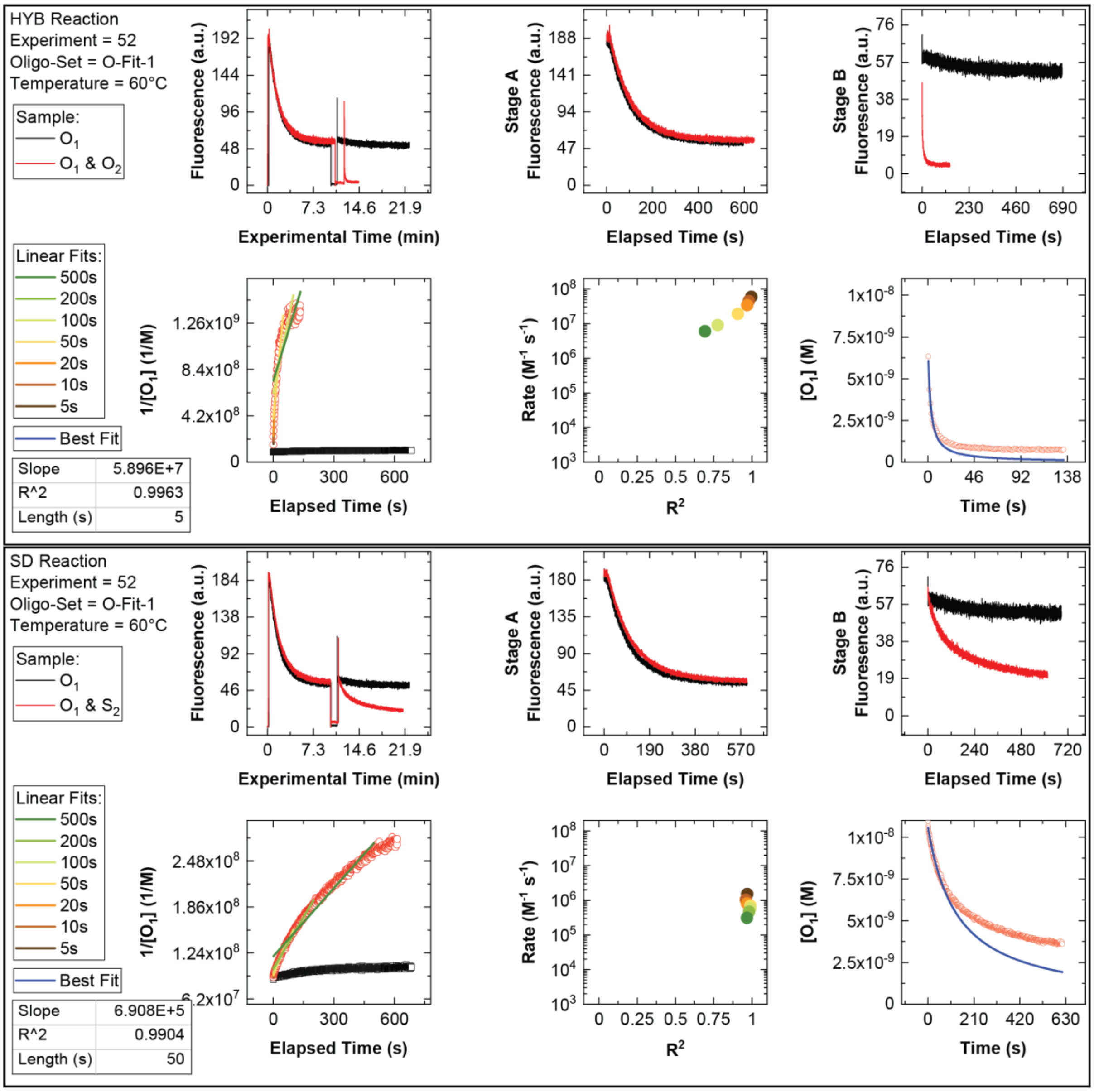


**Figure S65.** Report from experiment 52.

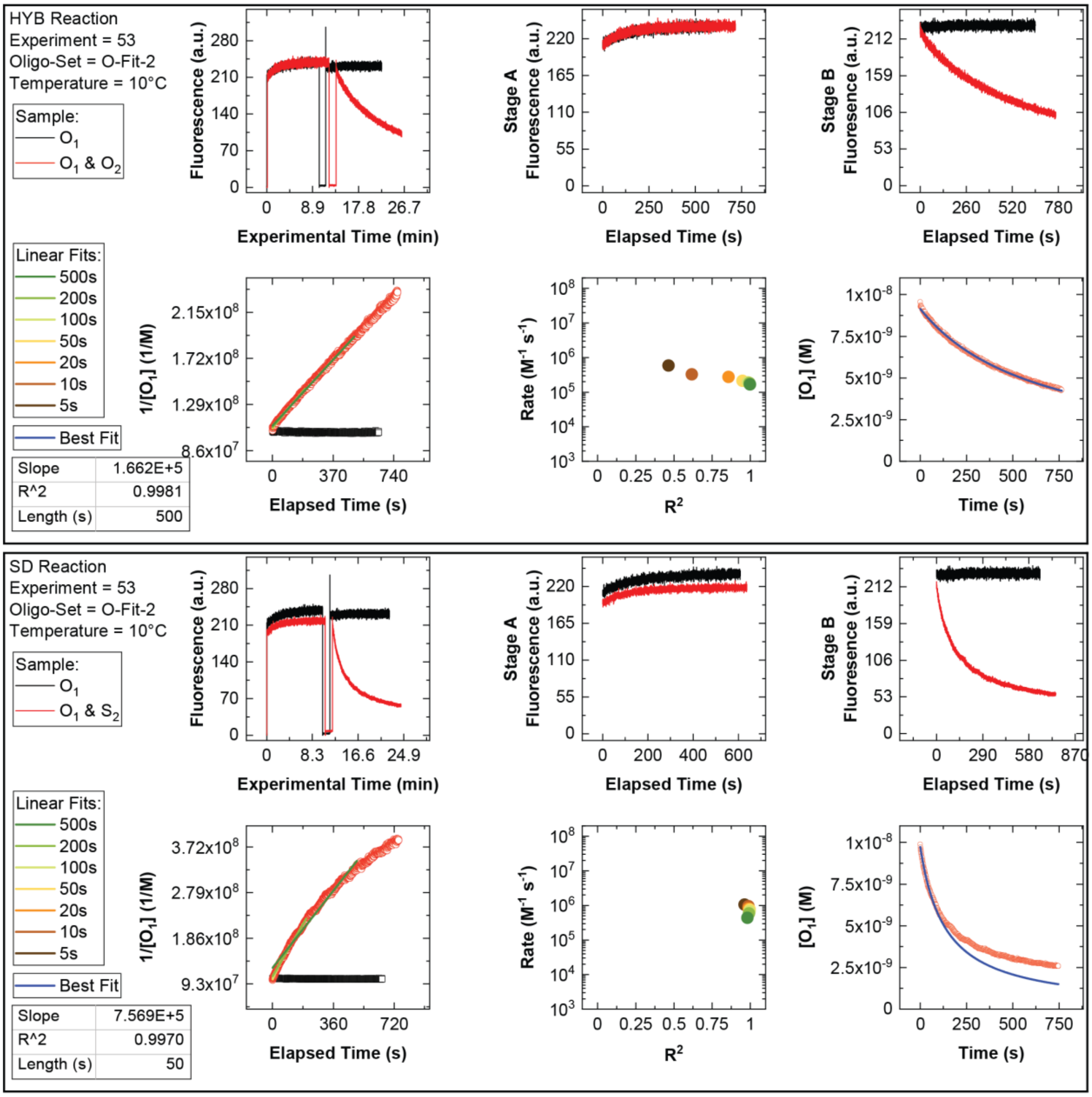


**Figure S66.** Report from experiment 53.

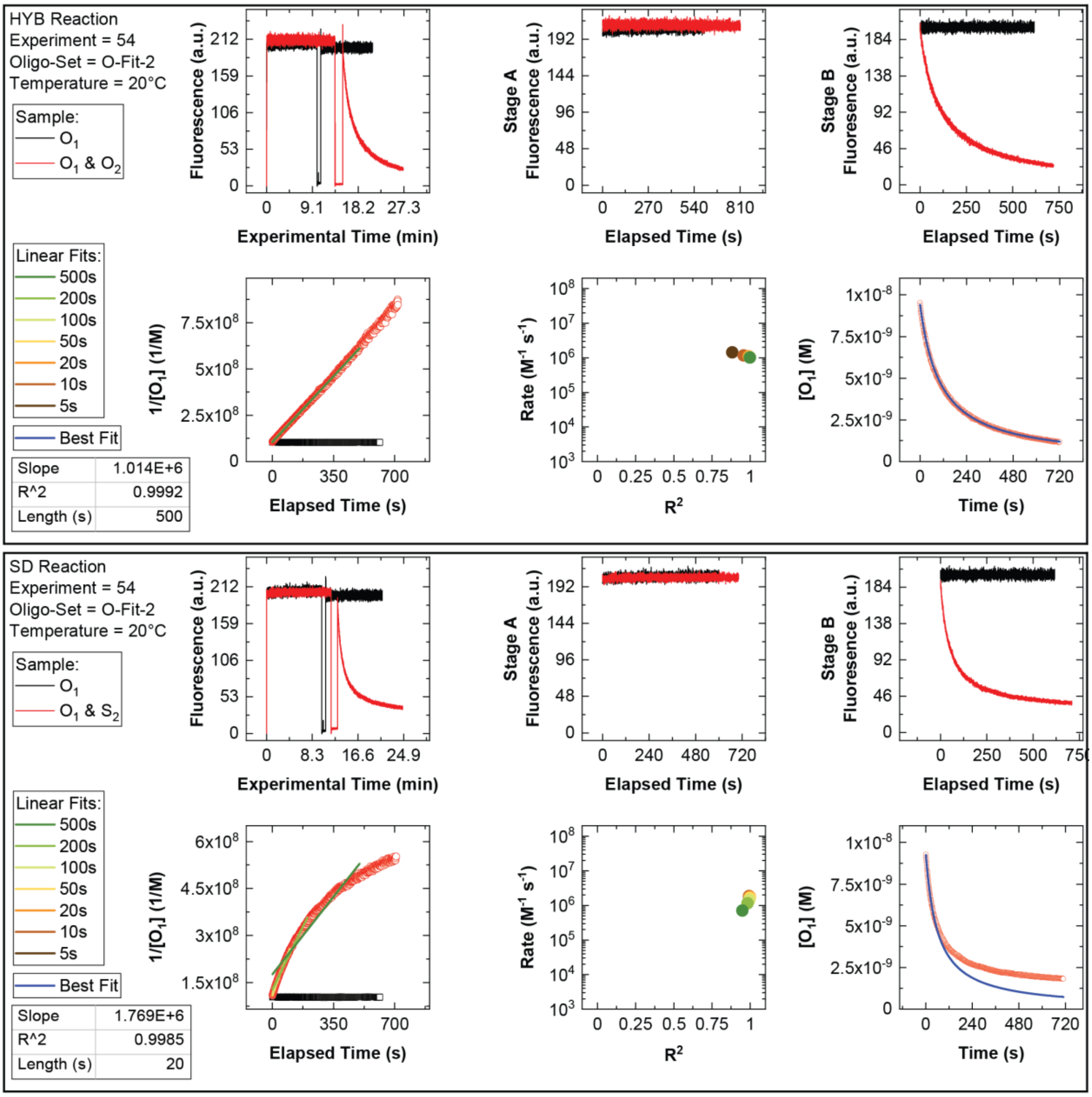


**Figure S67.** Report from experiment 54.

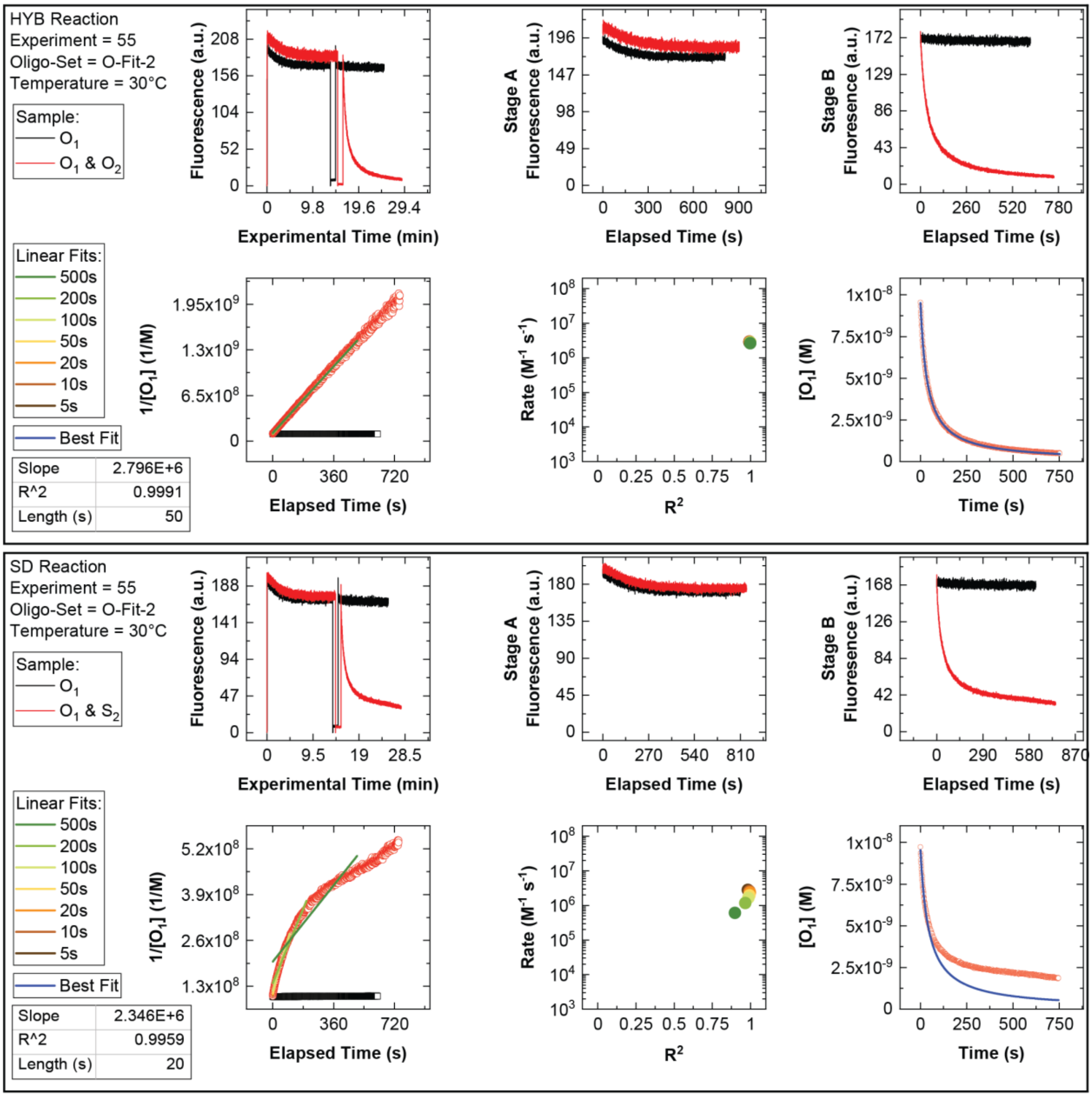


**Figure S68.** Report from experiment 55.

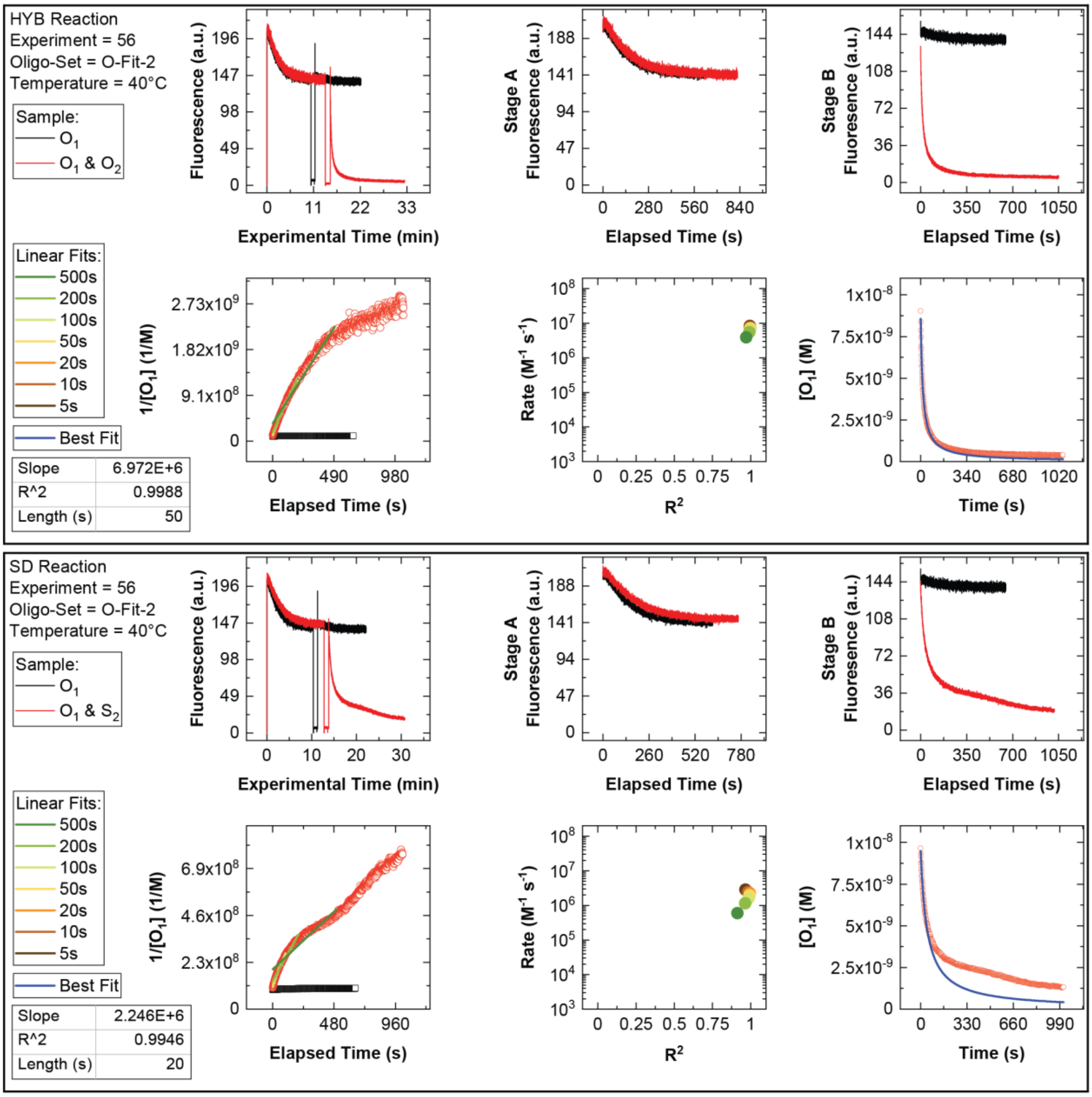


**Figure S69.** Report from experiment 56.

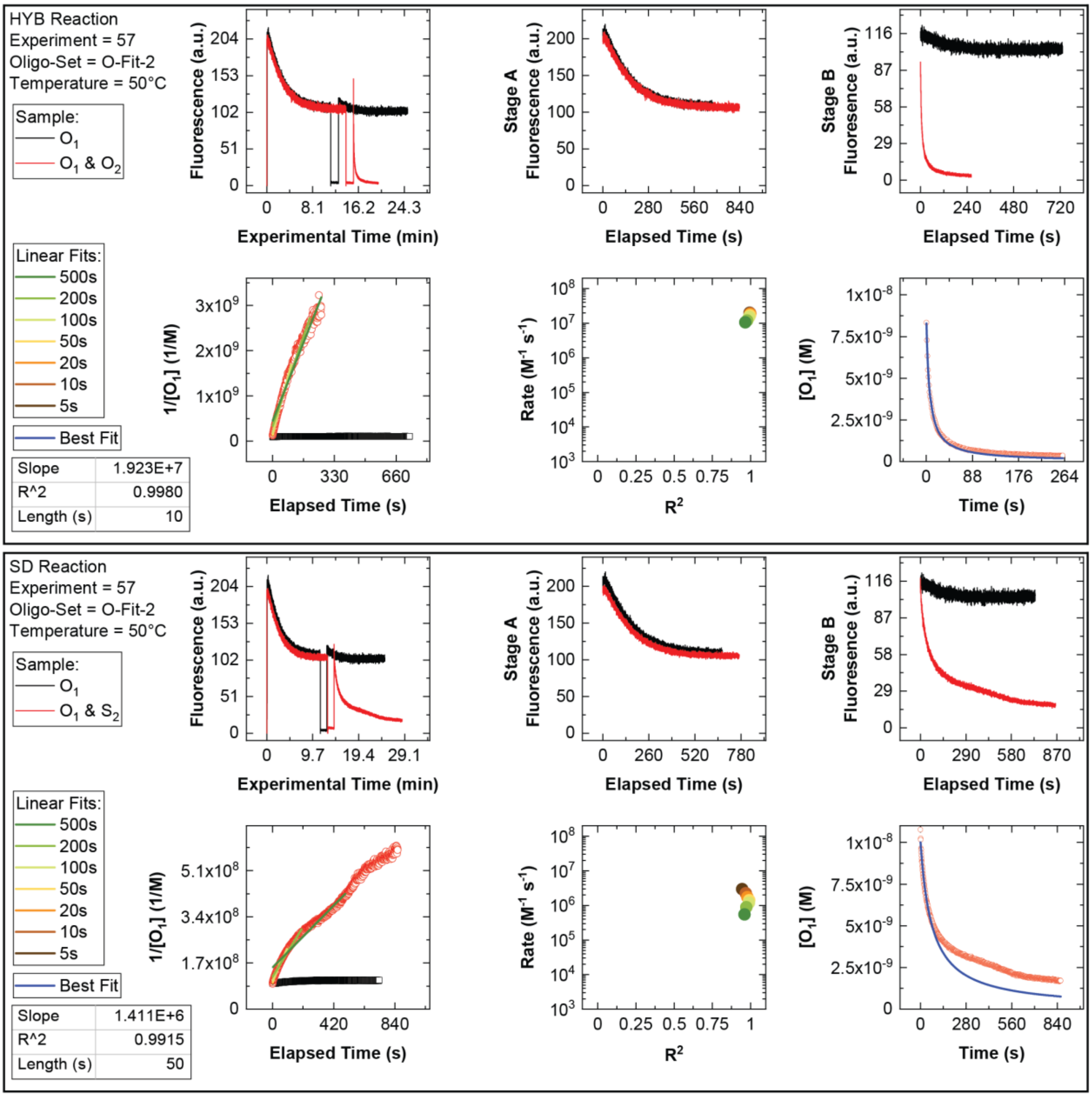


**Figure S70.** Report from experiment 57.

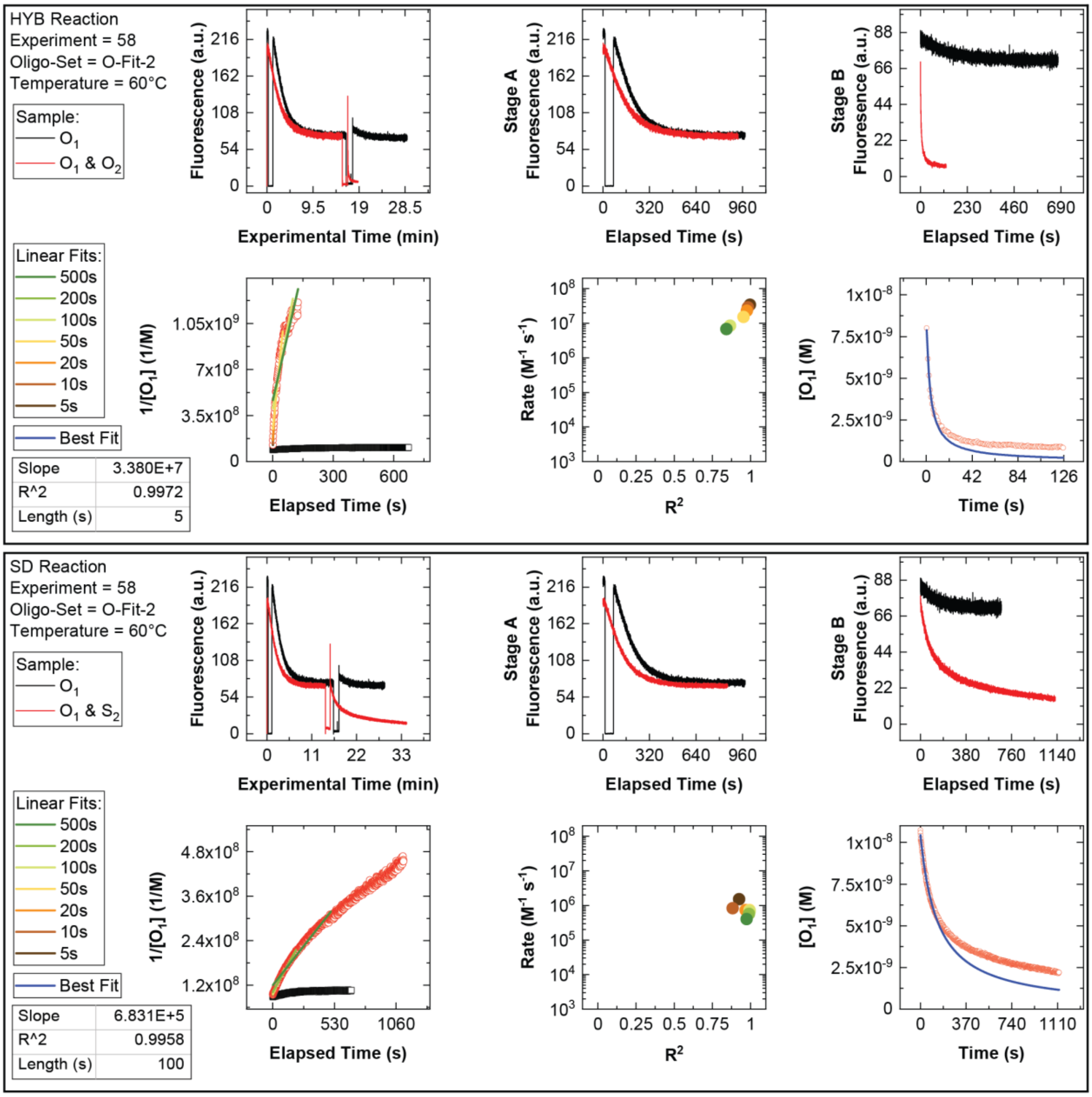


**Figure S71.** Report from experiment 58.

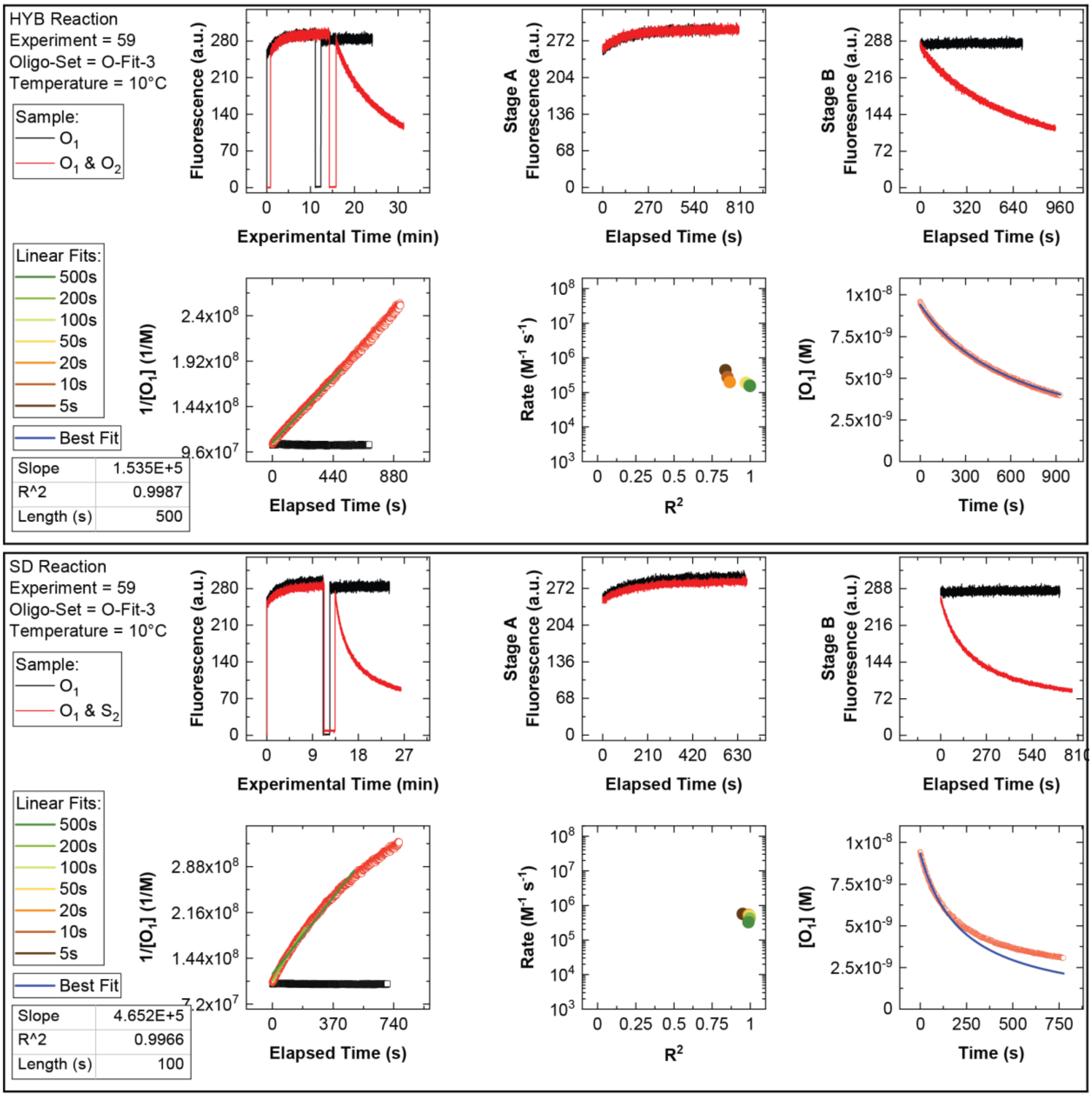


**Figure S72.** Report from experiment 59.

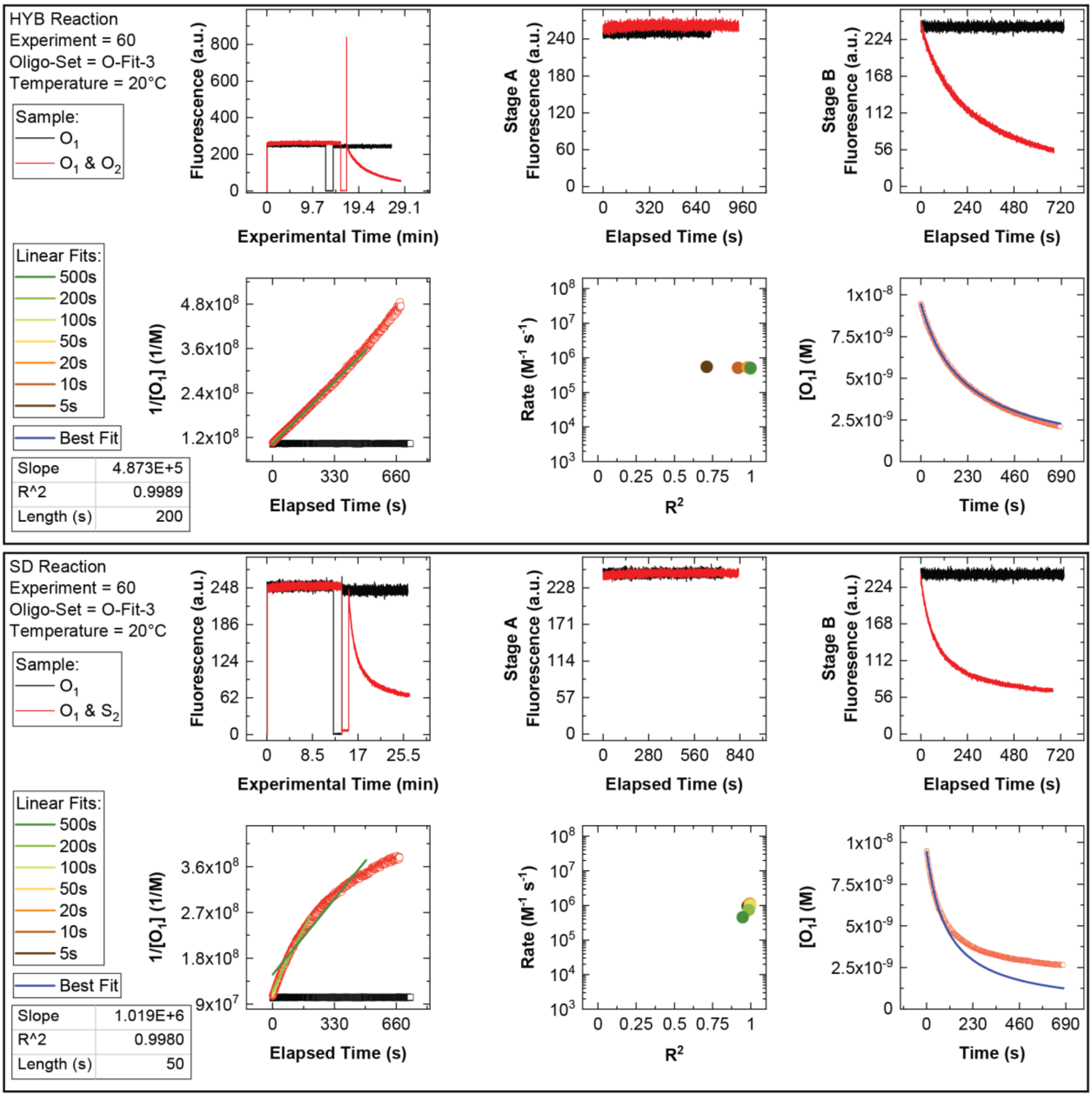


**Figure S73.** Report from experiment 60.

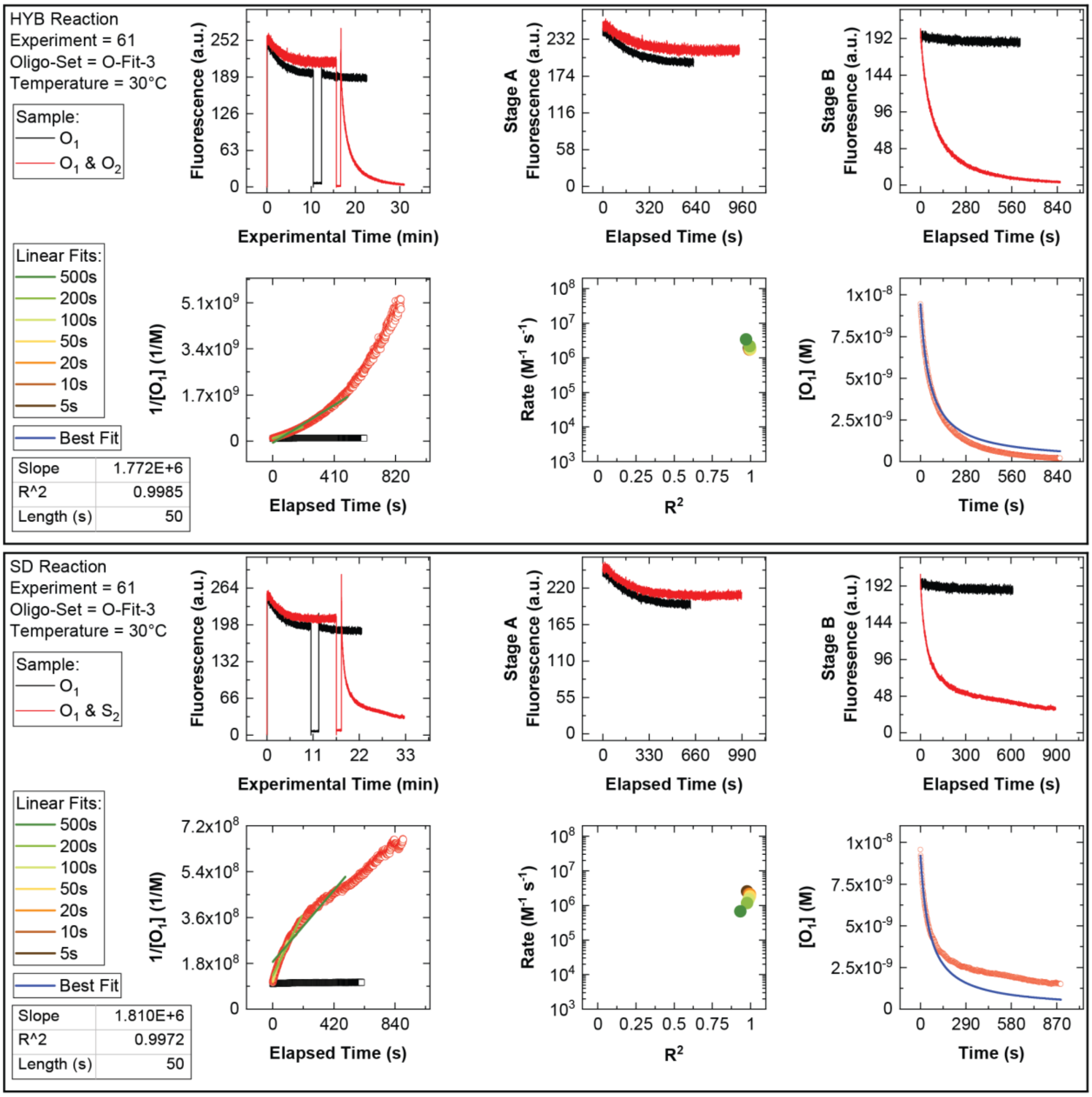


**Figure S74.** Report from experiment 61.

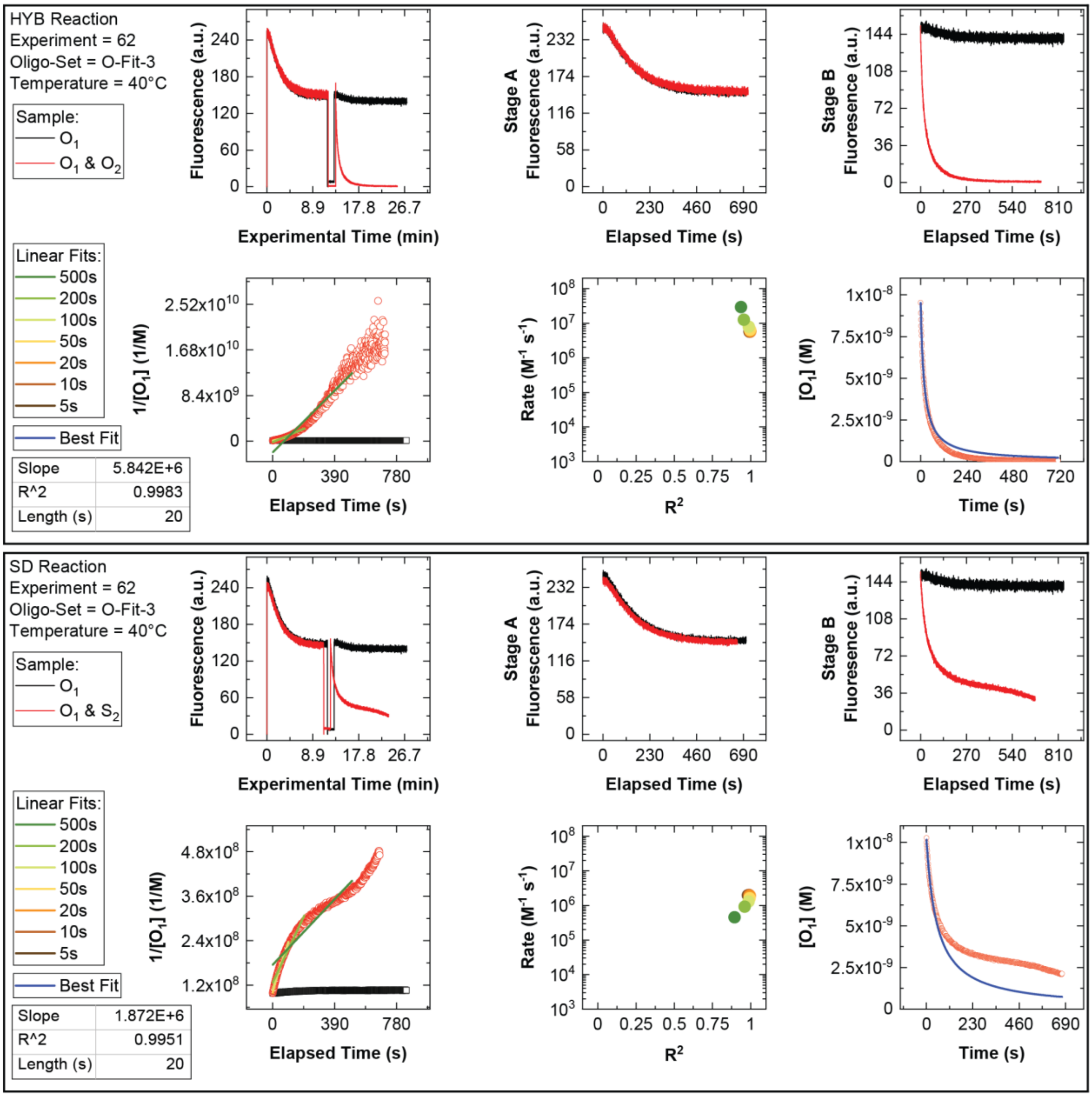


**Figure S75.** Report from experiment 62.

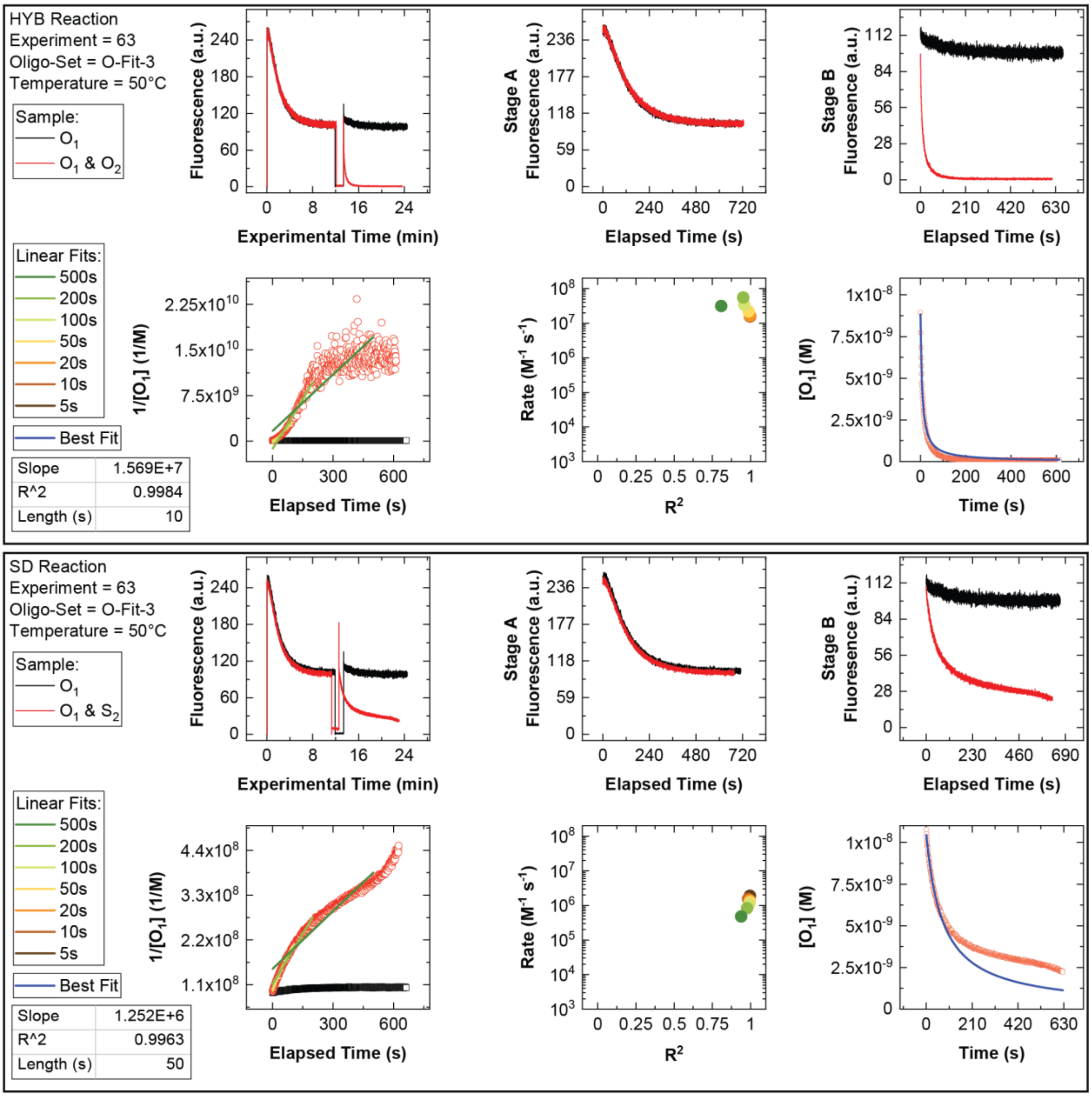


**Figure S76.** Report from experiment 63.

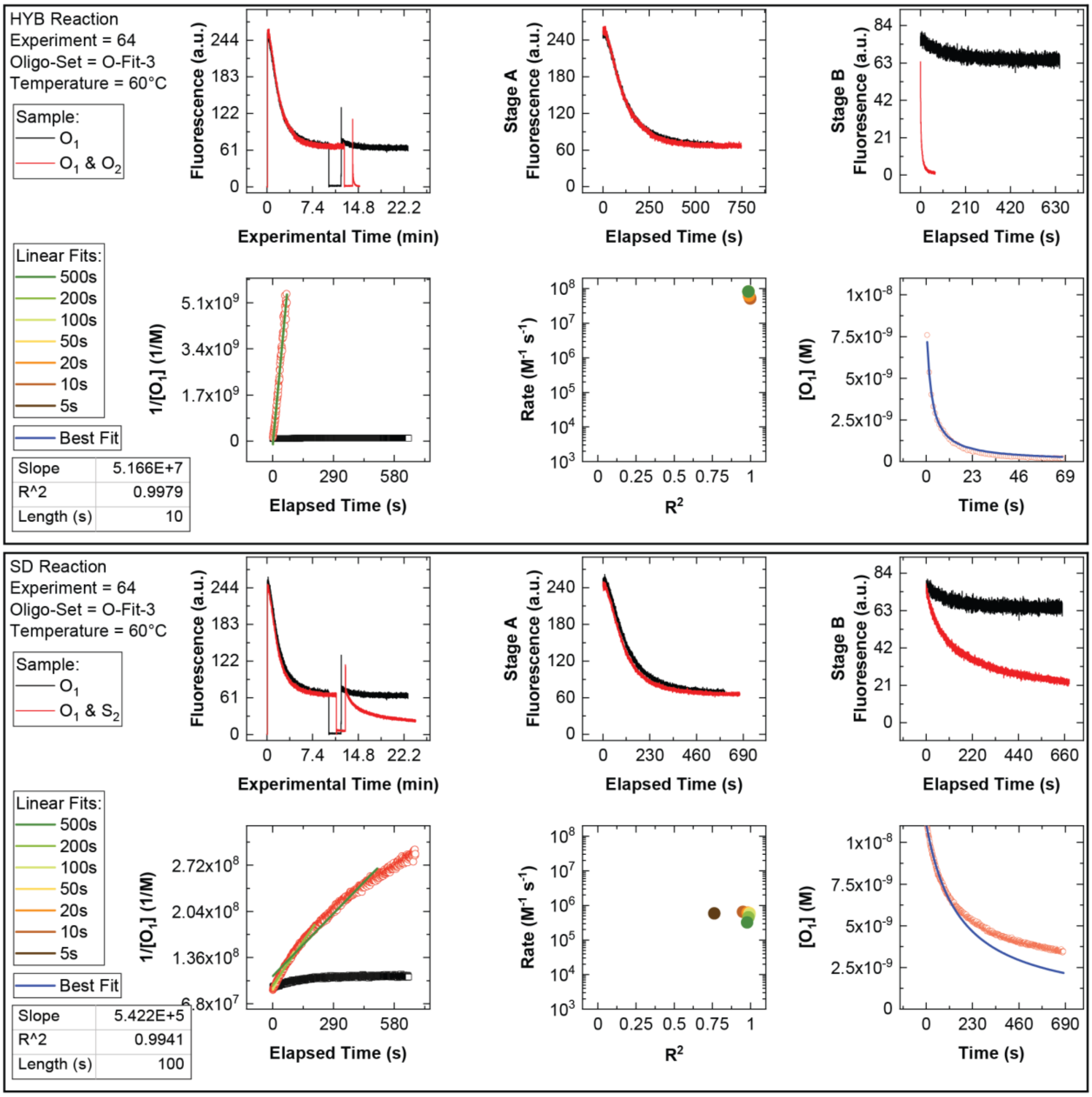


**Figure S77.** Report from experiment 64.

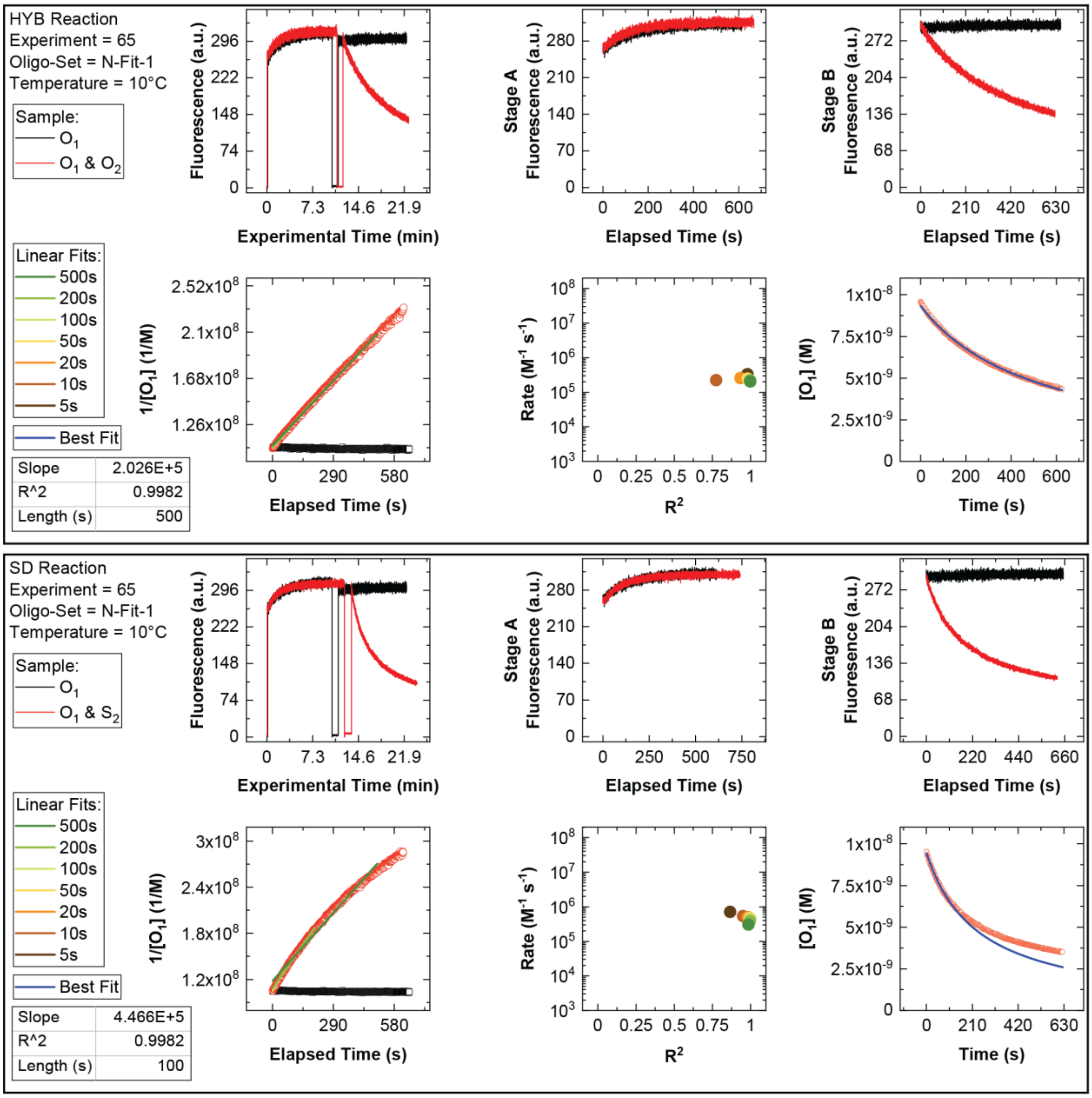


**Figure S78.** Report from experiment 65.

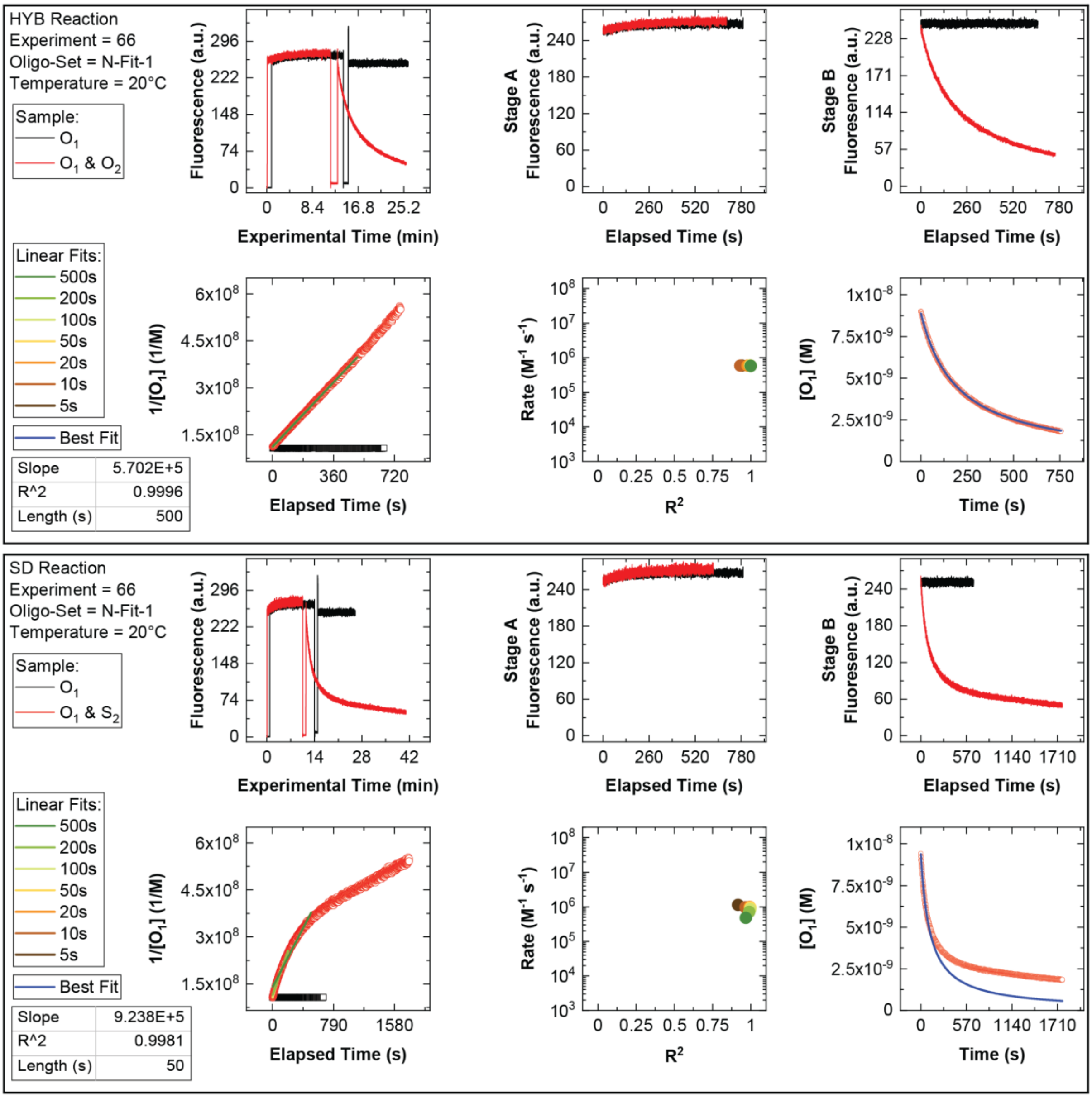


**Figure S79.** Report from experiment 66.

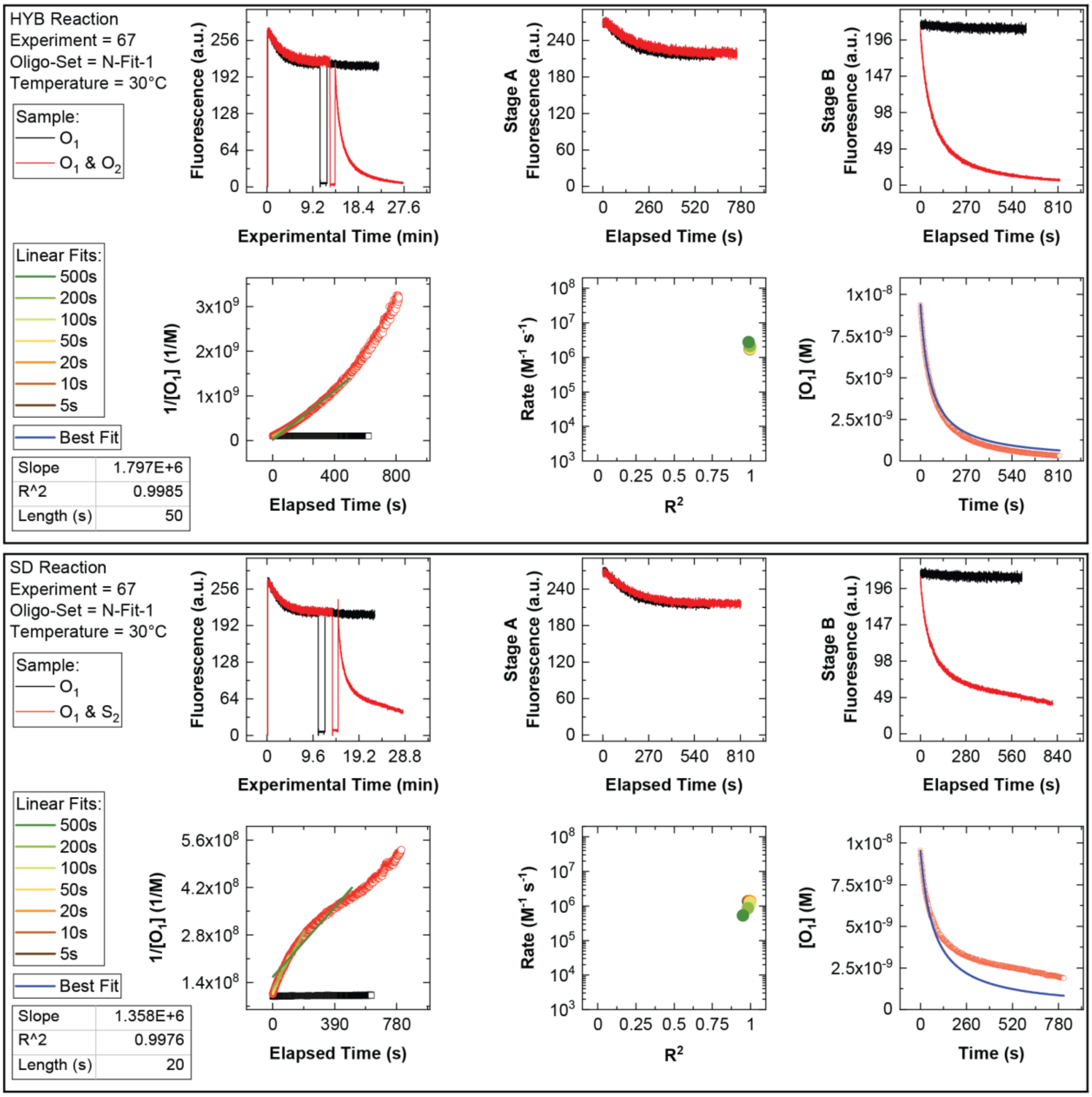


**Figure S80.** Report from experiment 67.

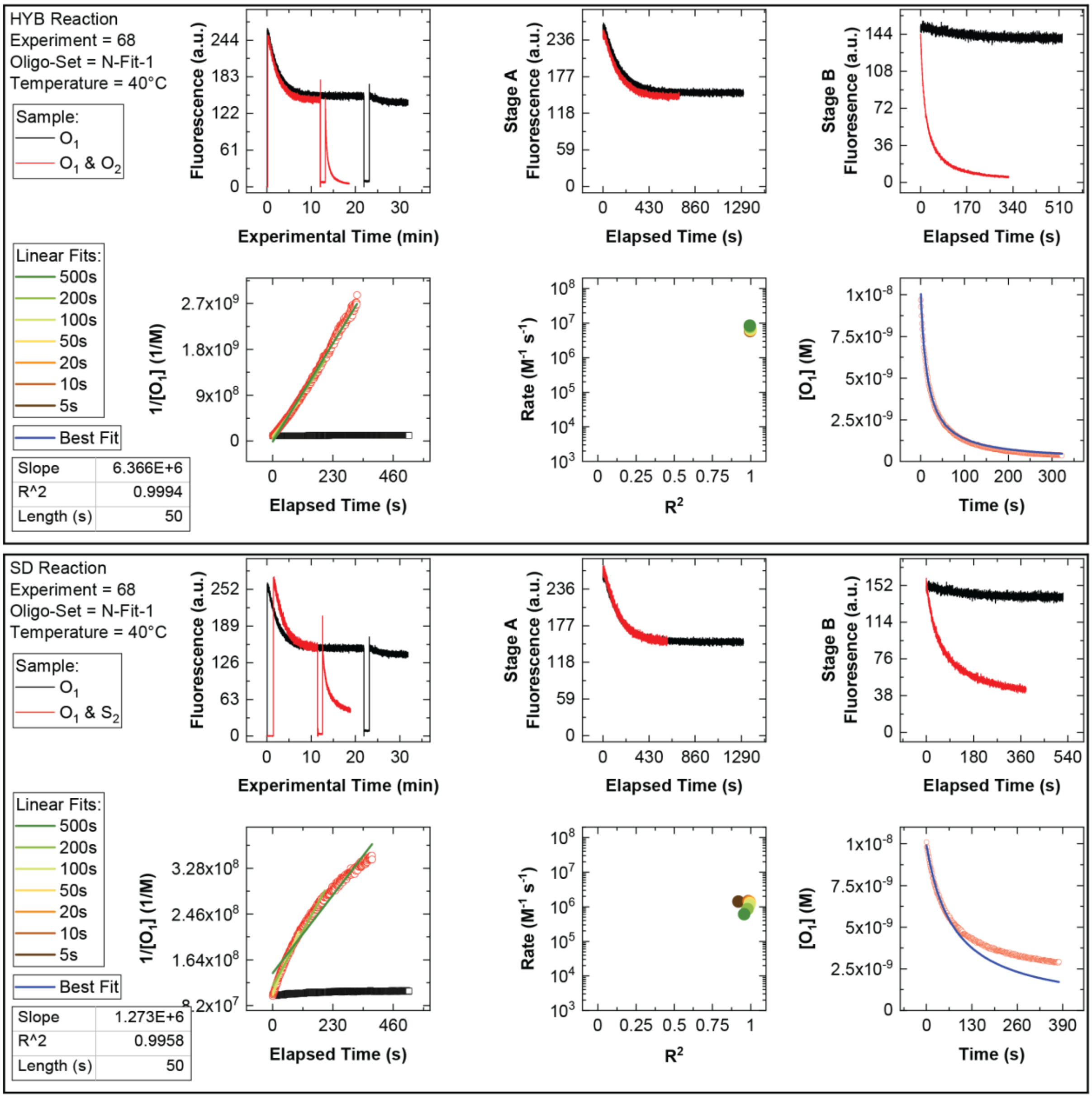


**Figure S81.** Report from experiment 68.

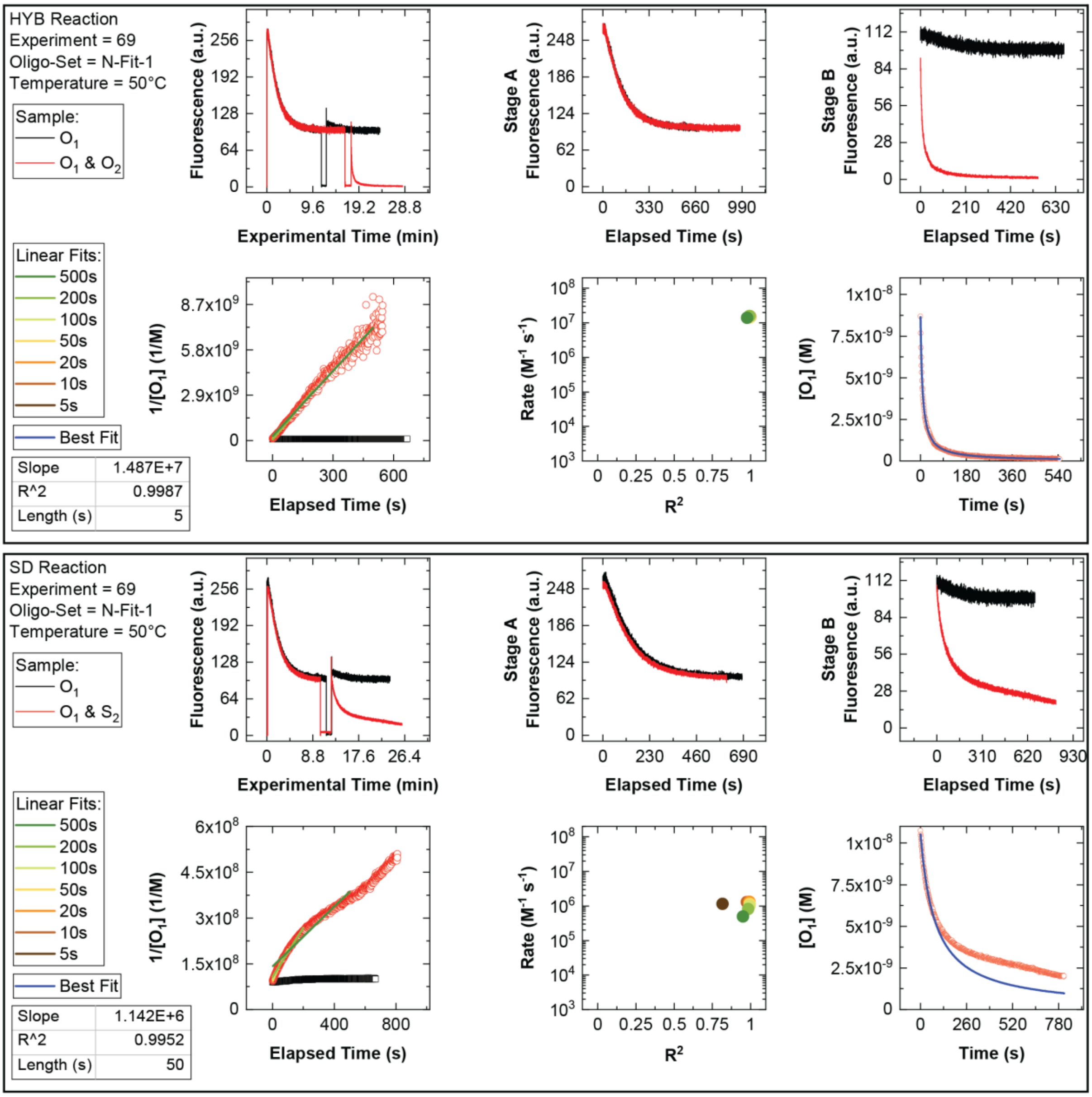


**Figure S82.** Report from experiment 69.

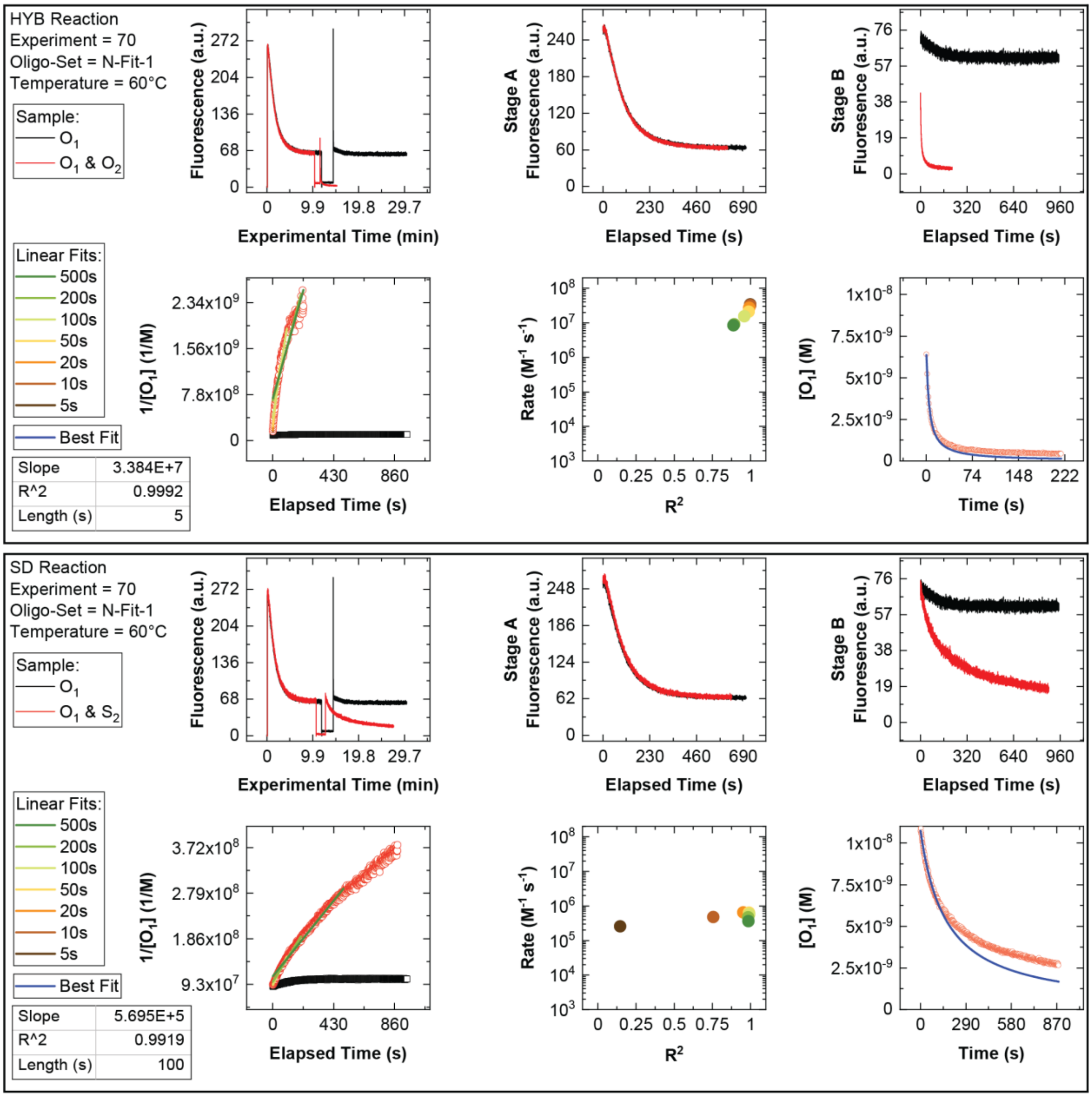


**Figure S83.** Report from experiment 70.

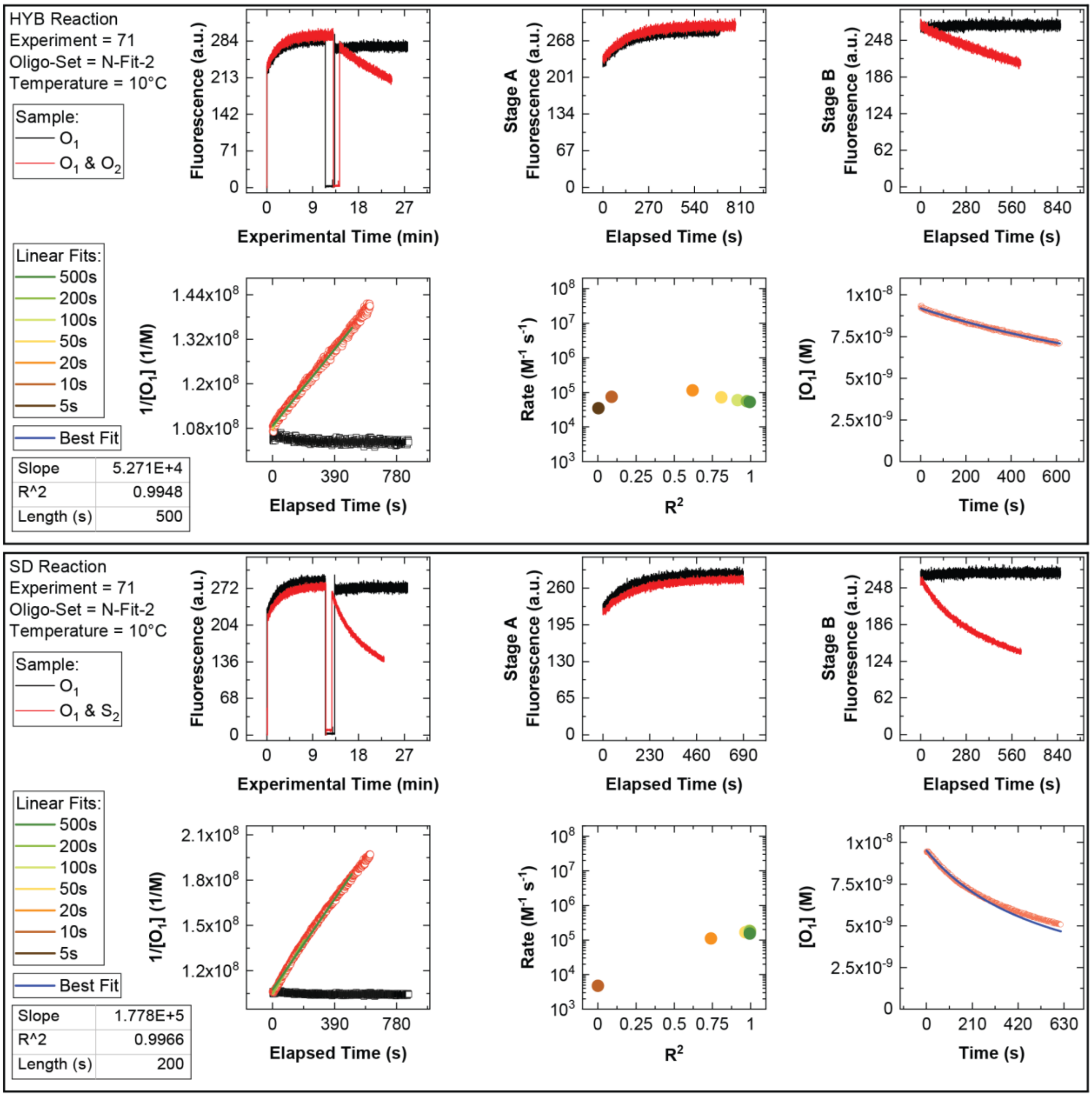


**Figure S84.** Report from experiment 71.

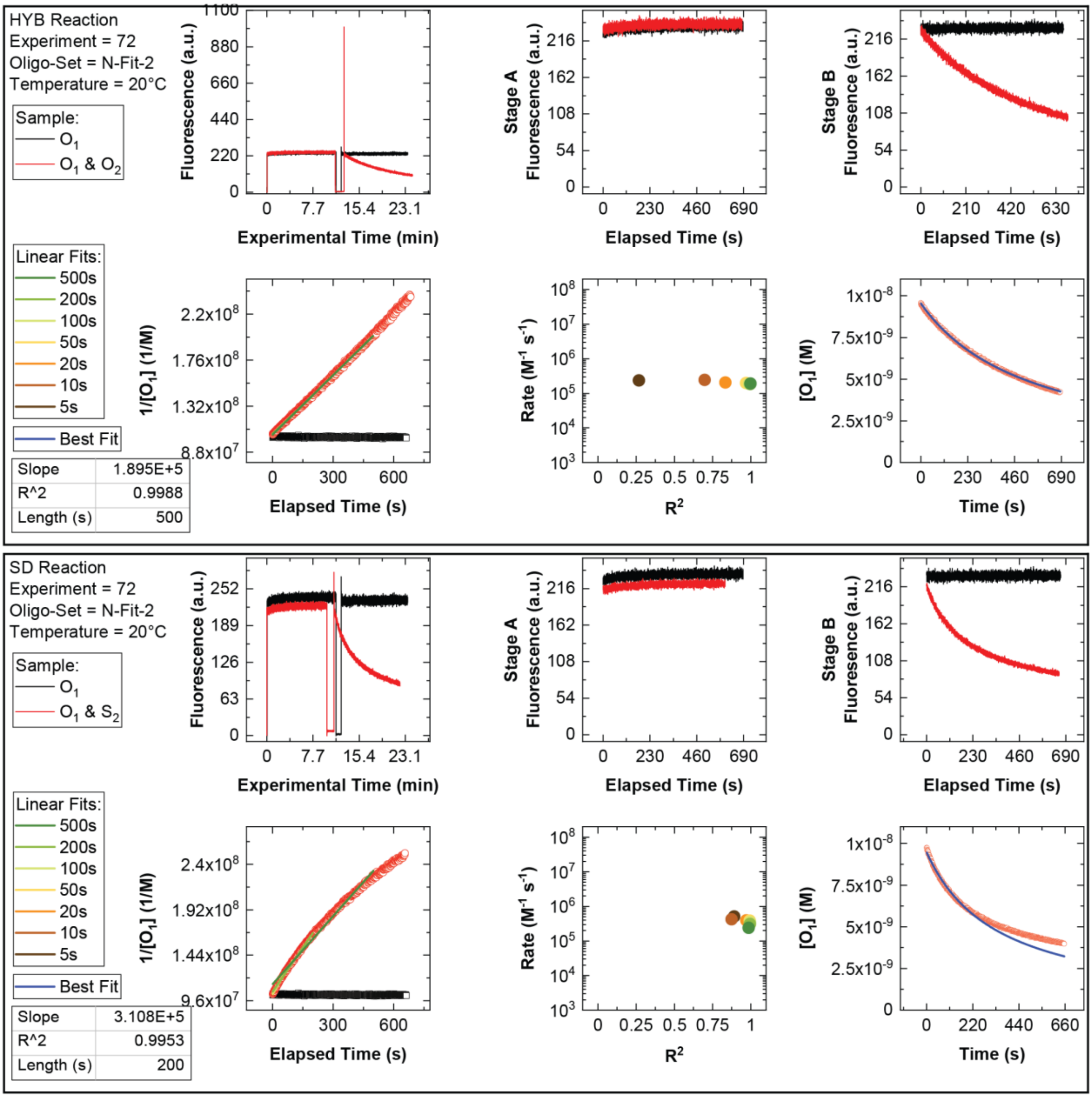


**Figure S85.** Report from experiment 72.

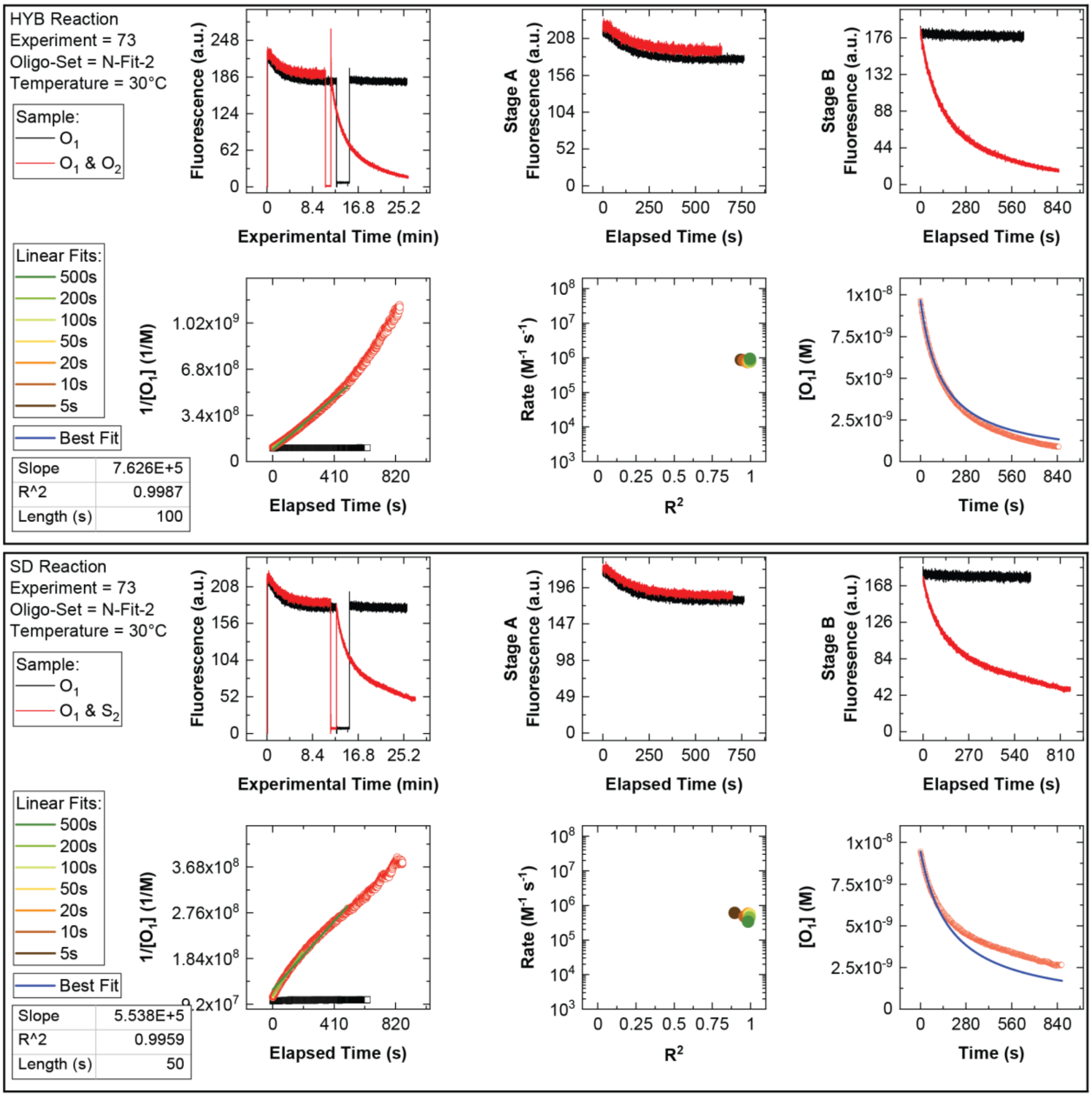


**Figure S86.** Report from experiment 73.

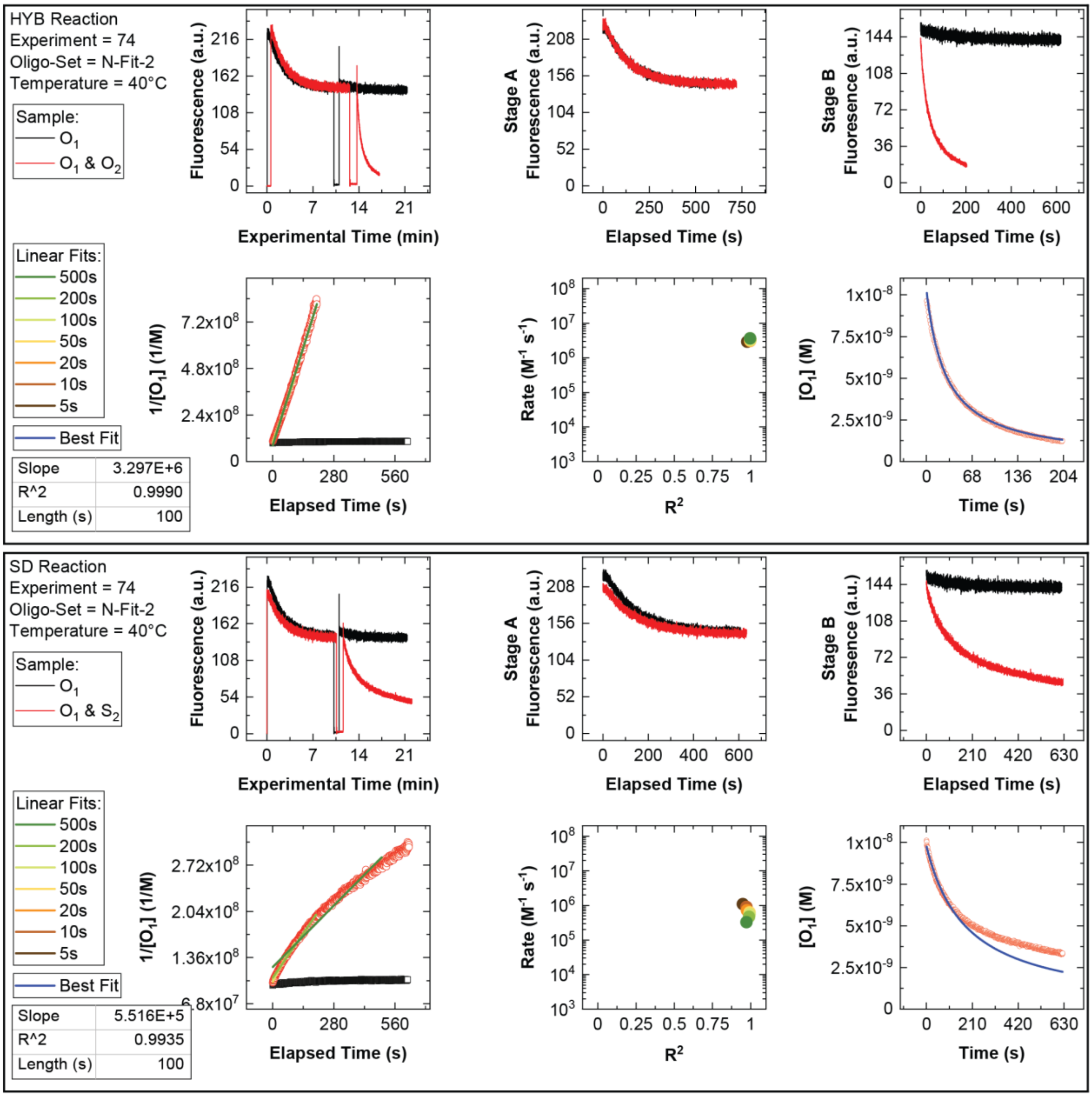


**Figure S87.** Report from experiment 74.

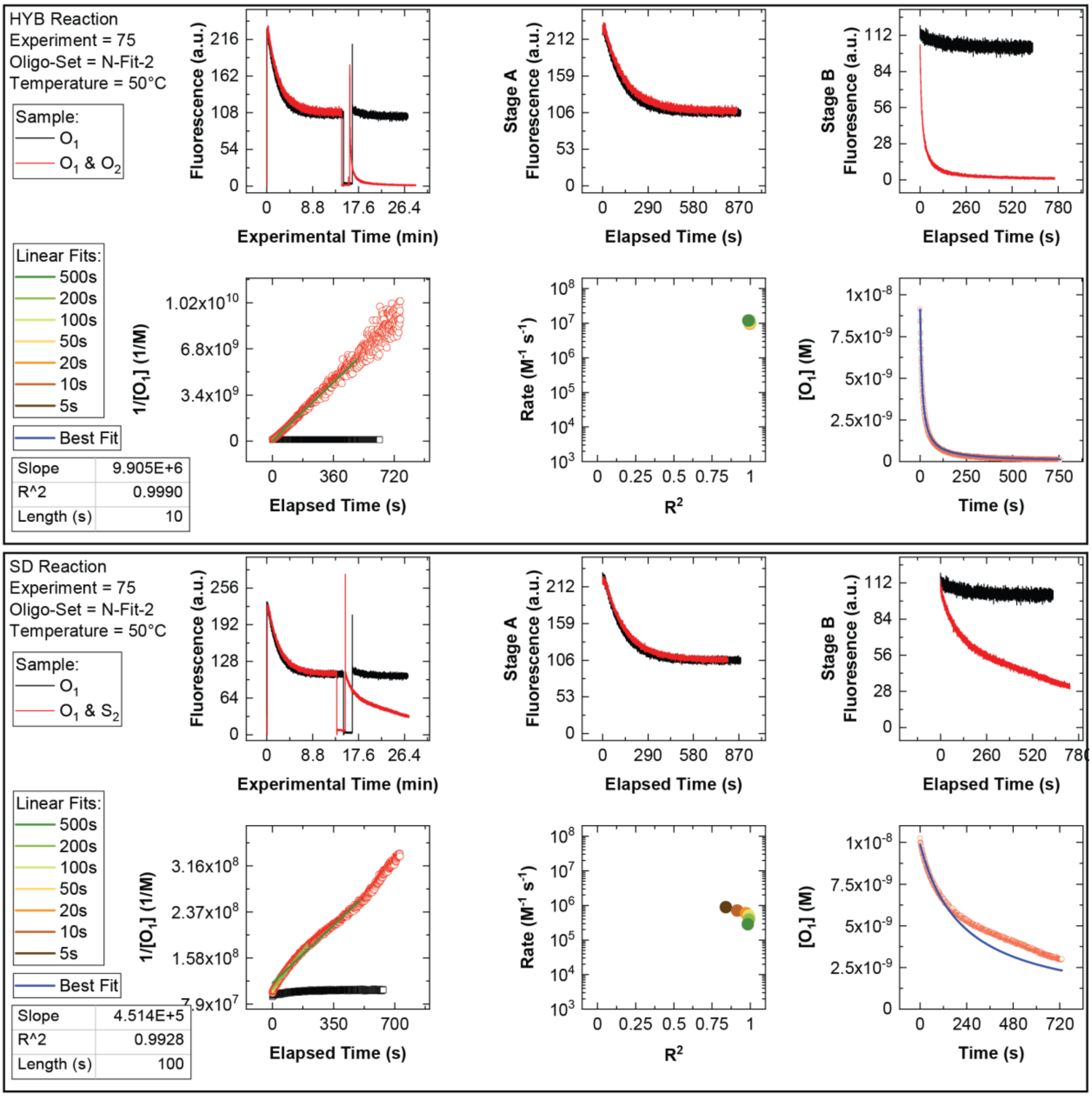


**Figure S88.** Report from experiment 75.

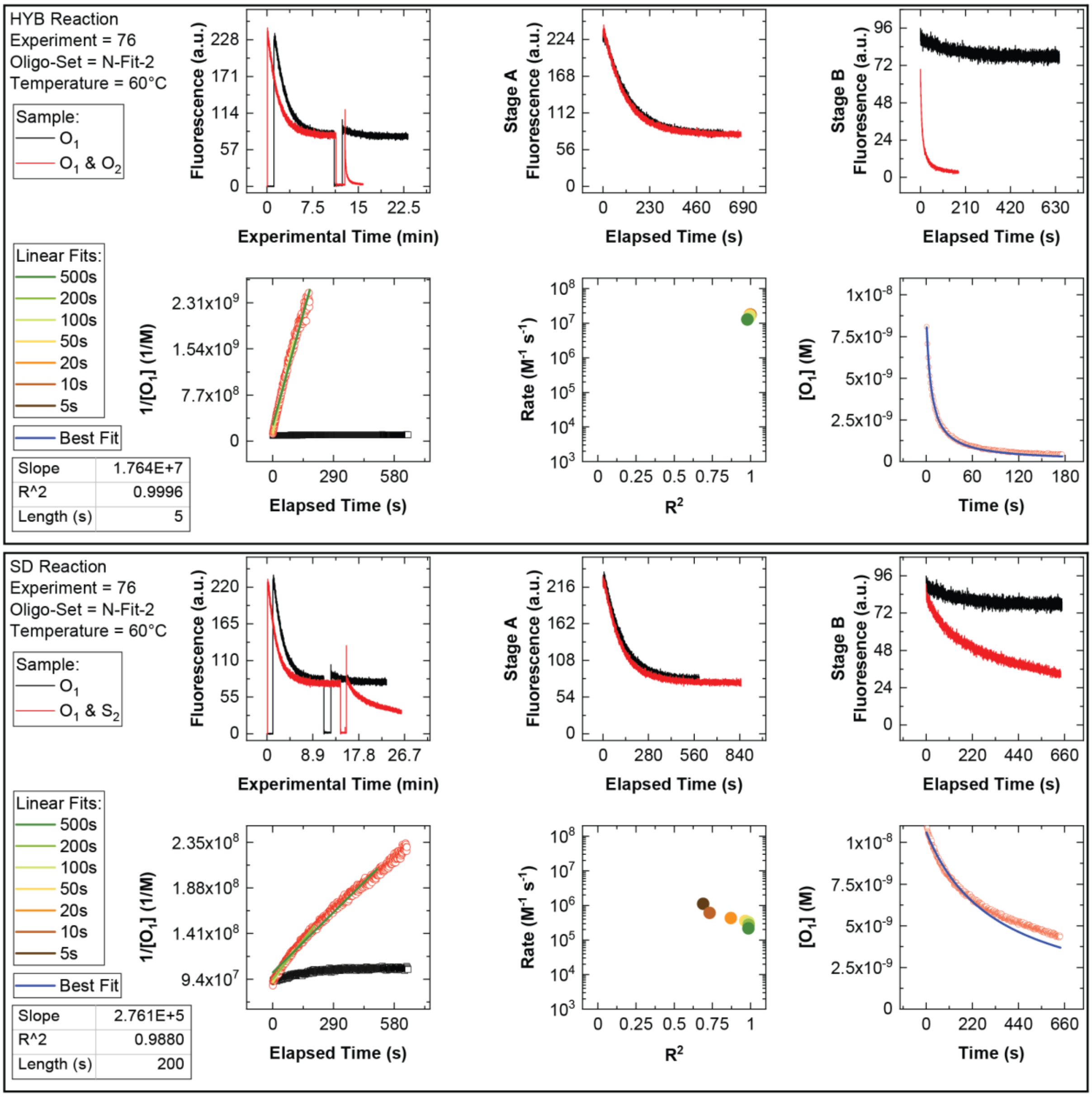


**Figure S89.** Report from experiment 76.

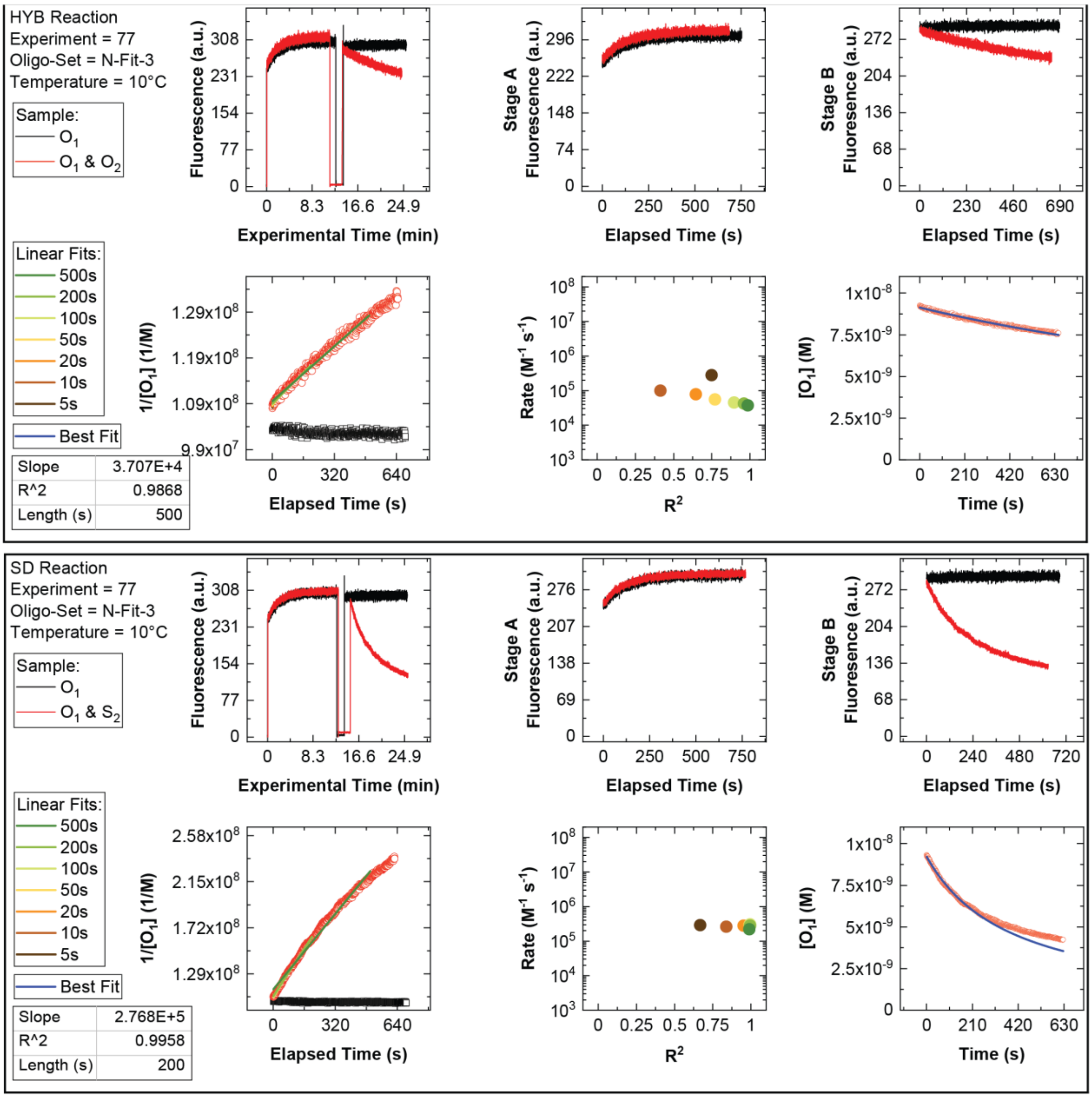


**Figure S90.** Report from experiment 77.

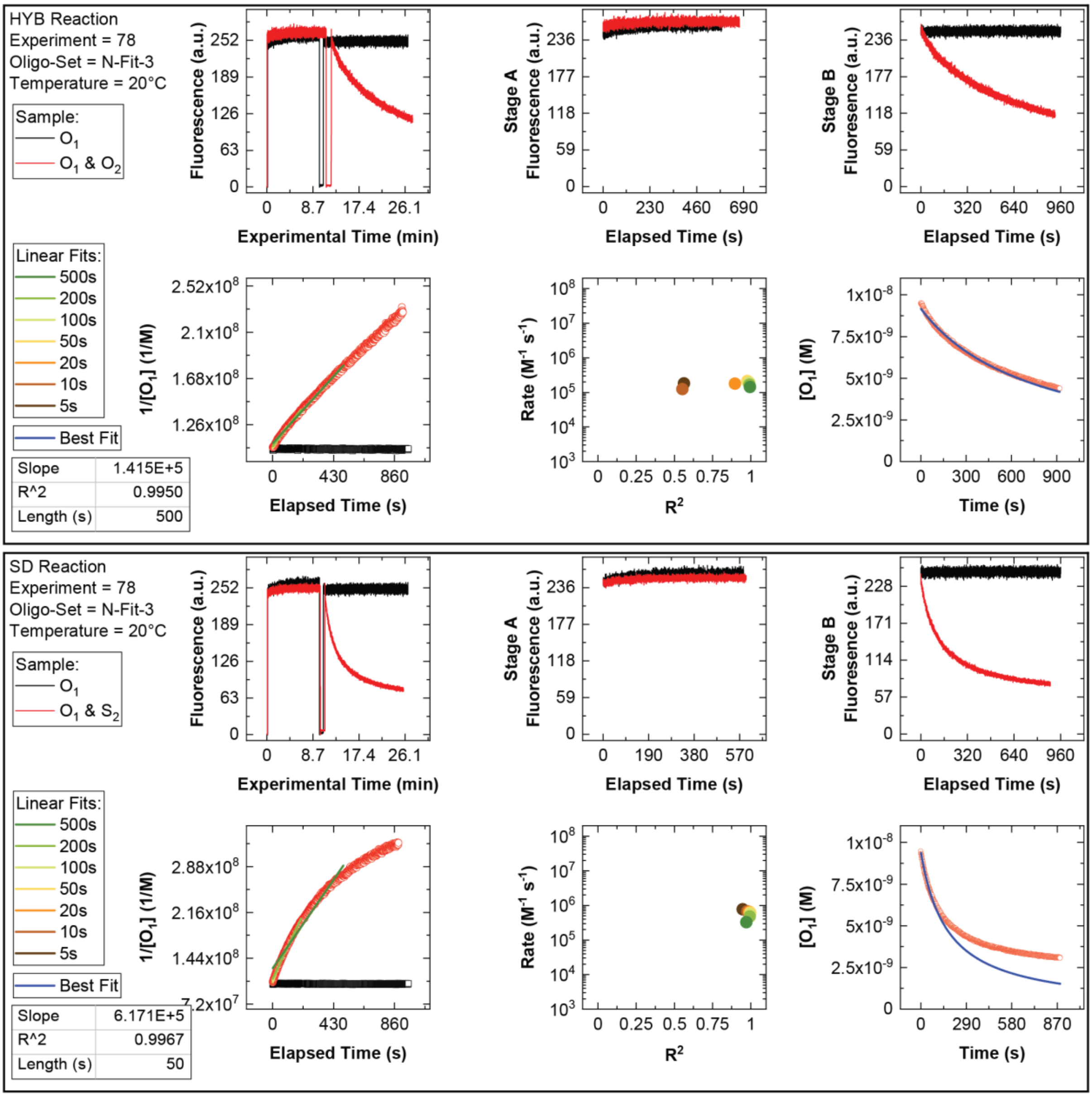


**Figure S91.** Report from experiment 78.

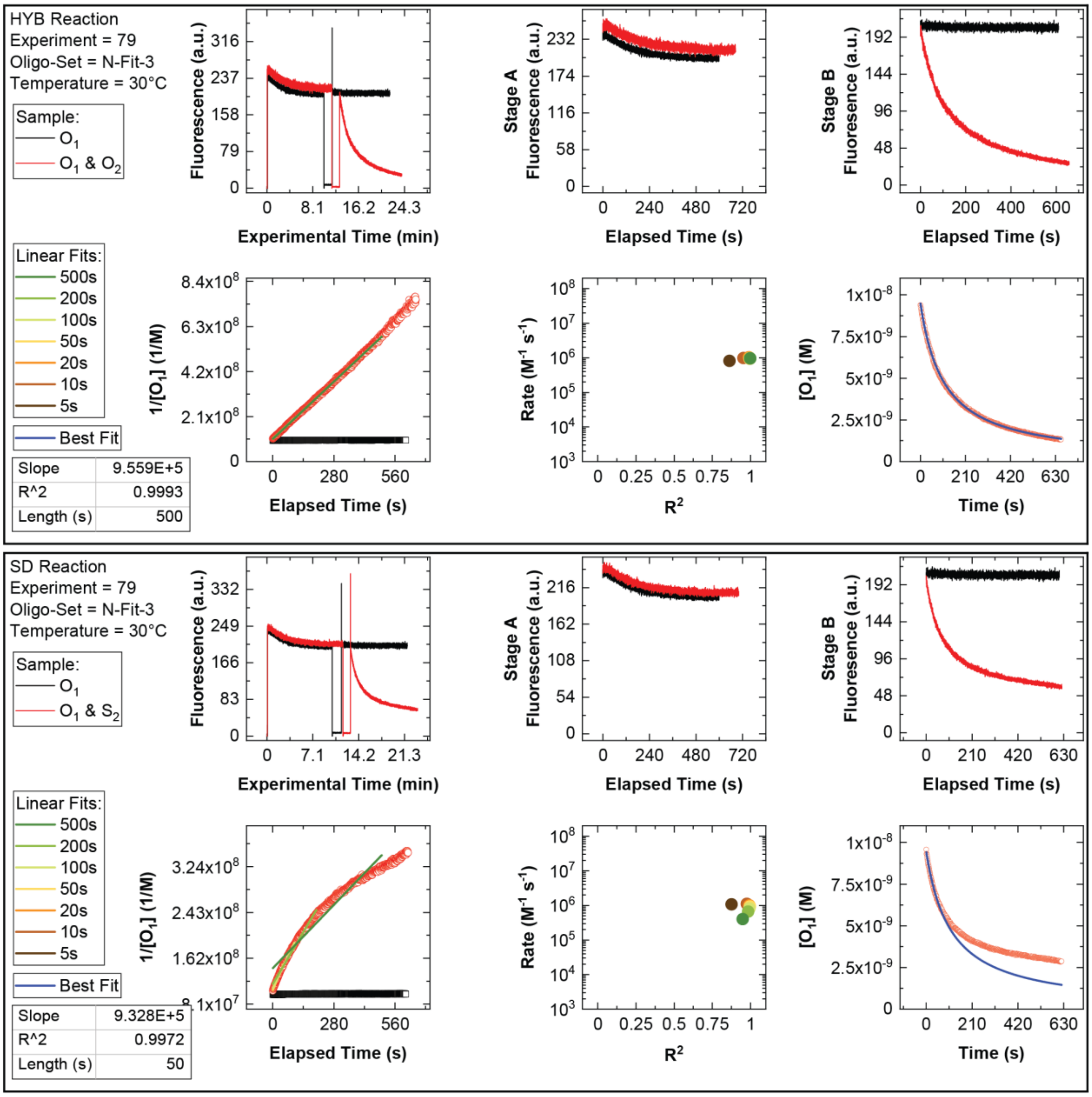

**Figure S92.** Report from experiment 79.

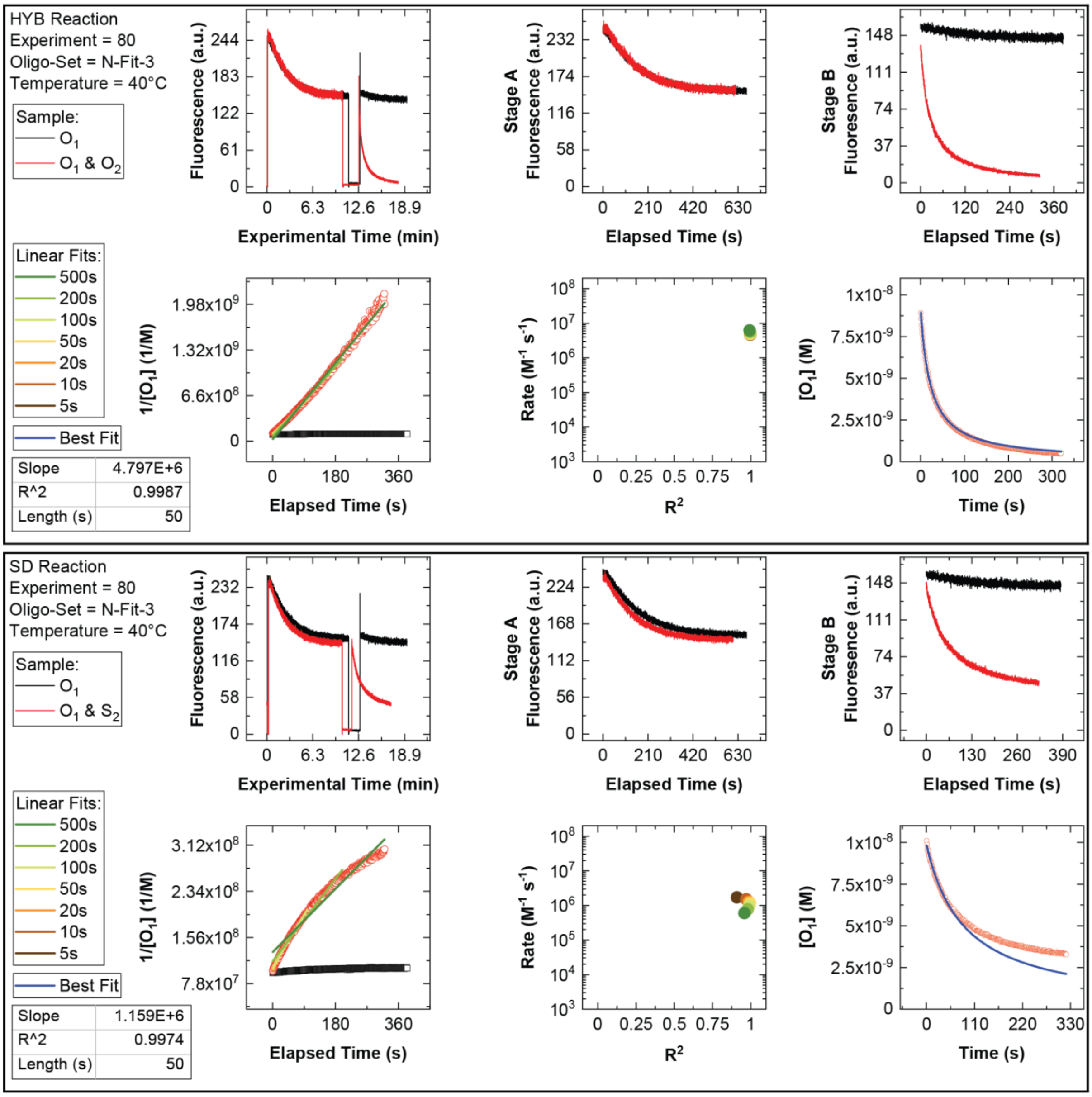


**Figure S93.** Report from experiment 80.

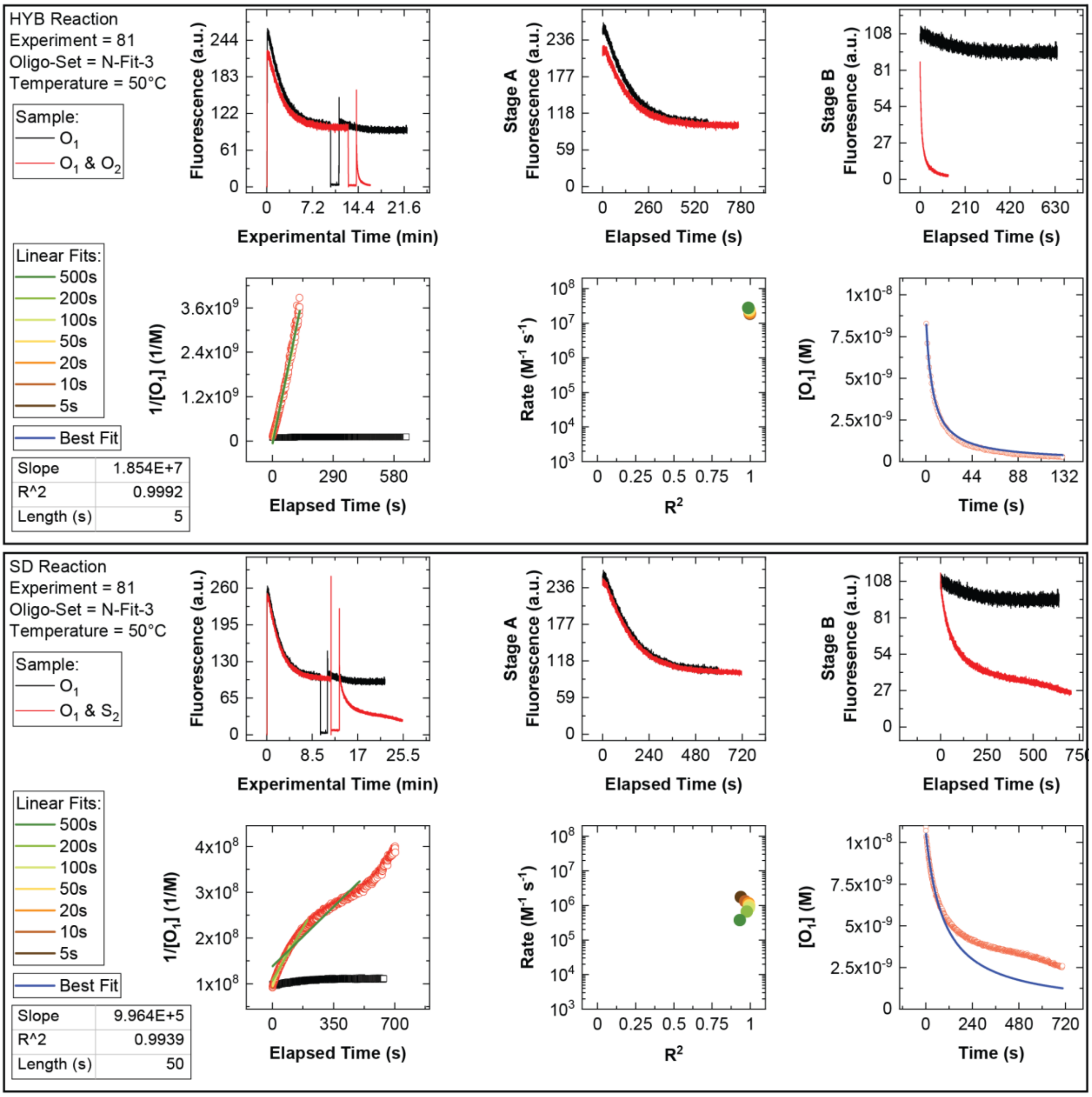


**Figure S94.** Report from experiment 81.

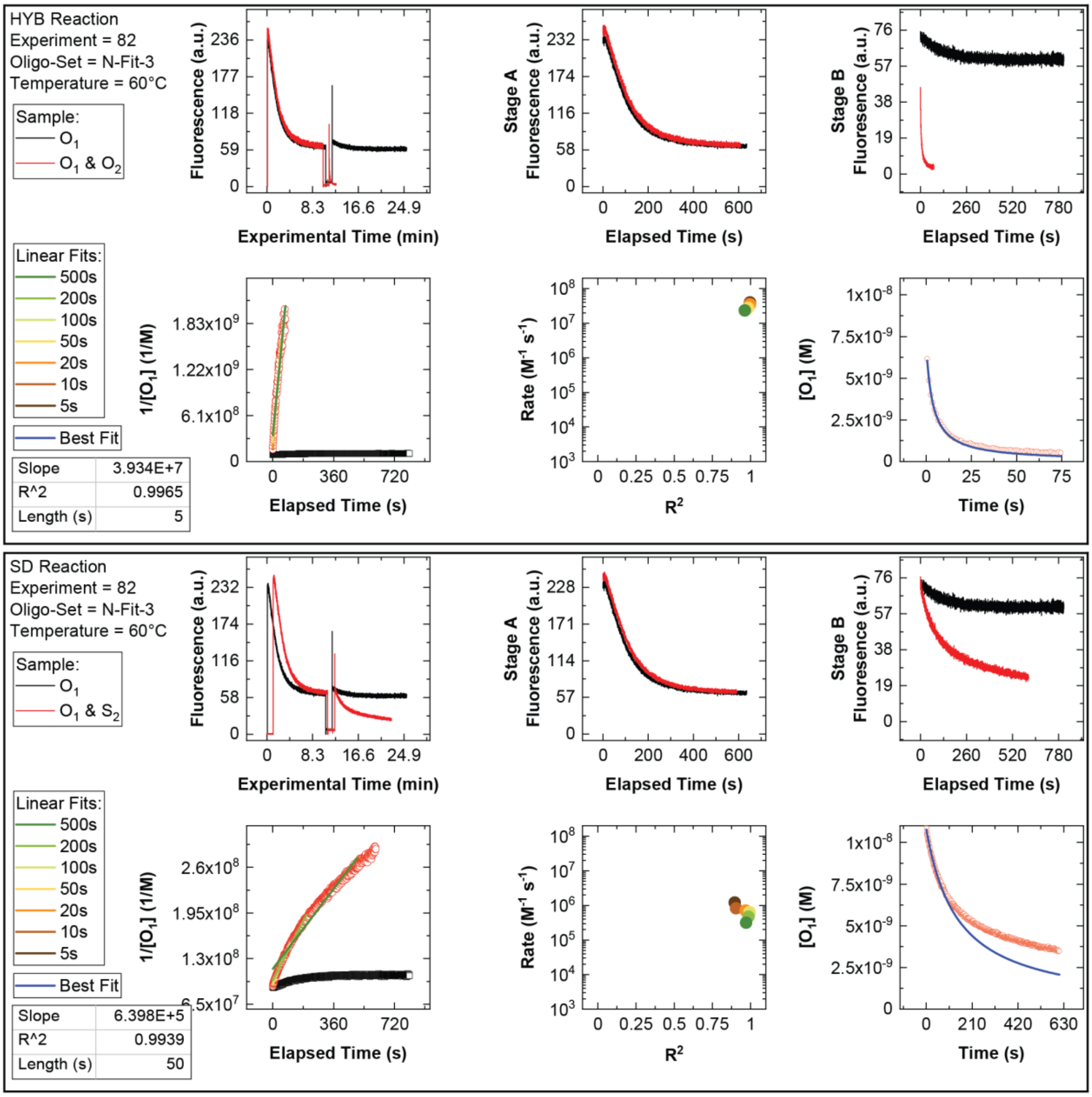


**Figure S95.** Report from experiment 82.

## Supplementary Note 5: New Oligomers for Existing Designs

**Table S6.** Unncessary duplexes in 10x10x10 DNA brick reported by Ke *et al.*.

| | **Unnecessary intra-oligomer duplexes (count)** | | **Unnecessary inter-oligomer duplexes (count)** | |
|---|---|---|---|---|
| **Size** | As-published | Optimized | As-published | Optimized |
| 1 bp | 69479 | 69479 | 36915671 | 36915671 |
| 2 bp | 15118 | 11775 | 8632911 | 8620755 |
| 3 bp | 3197 | 67 | 2021642 | 2010751 |
| 4 bp | 687 | | 478168 | 465815 |
| 5 bp | 143 | | 114059 | 104831 |
| 6 bp | 28 | | 27783 | 22261 |
| 7 bp | 6 | | 5868 | 3846 |
| 8 bp | 1 | | 1612 | 339 |
| 9 bp | | | 593 | 63 |
| 10 bp | | | 160 | 2 |
| 11 bp | | | 46 | |
| 12 bp | | | 21 | |
| 13 bp | | | 13 | |
| 14 bp | | | 12 | |
| 15 bp | | | 12 | |
| 16 bp | | | 12 | |
| 17 bp | | | 12 | |
| 18 bp | | | 3 | |
| 19 bp | | | 2 | |
| 20 bp | | | 2 | |
| 21 bp | | | 2 | |
| 22 bp | | | 2 | |
| 23 bp | | | 2 | |
| 24 bp | | | 2 | |
| 25 bp | | | 2 | |

**Table S7.** New oligomers for the 10x10x10 DNA brick design reported by Ke *et al.*.

| Oligo Name | Oligo Sequence (5' - 3') |
|---|---|
| Strand-1 | CAAATGCTCCGAAACCCGTGCGTGATCCTGAA |
| Strand-3 | ATGTCAAAACCTCTCGCAGTCGTAAGTAAGTC |
| Strand-6 | GGCTTCTGGGCGCGAGGCTTCCCGATTGACAC |
| Strand-7 | TCCGCAACGTCTGTGTCCGAATATGTGAGGCC |
| Strand-8 | TTATGCGTGGCTGTGGCTTGTTGAGACCCGAA |
| Strand-11 | GTTGTGCAGAGGTCCATCCCGCTTAGGCTTAG |
| Strand-12 | CGTGTCGAAAGGAAGTAGAGGTTGAGATGAGC |
| Strand-13 | TGTCGCCGTATCTGTATGCACTGGTAAATGGT |
| Strand-16 | ATCTTTGCGGAATGTTAATGACCTTTGTGTGT |
| Strand-17 | AGGTCGAACATCAGTCTTGCTTGGAATCACAC |
| Strand-18 | GCCCTCGAATAGTGCCCTTTAATAGTCTCATG |
| Strand-23 | GCCCAGATCGTAAAGCCGGTGTATTCAAGCAT |
| Strand-25 | ACAATAGGGCGGCAGGCGTCTCTCTTCGGGCA |
| Strand-27 | GAGCTATTTGGTAAGTGCGGTTGGAAGTATCT |
| Strand-28 | ATCACATCTTCCACTCGGTATTAAACATTCGG |
| Strand-29 | ATATCACCCAGAGAAACACTACGTCATCCTTA |
| Strand-30 | TTGGGAAAGCATCGACTCTAGCCGCACTGTAC |
| Strand-31 | CTTAGCAATAGGGCGGCGTCATCTCGAAATAC |
| Strand-32 | TGAAAGTTATGAGACAATGCCAAGAAGCGAGT |
| Strand-33 | TTTGACACACCGATGGCACTGATTAGGCGAGG |
| Strand-34 | TCTGCGAATACGACATAGTGAGCCTGGTTAAA |
| Strand-35 | AGACCGTTGTTAGTAGAAATCCTTGCATGAAA |
| Strand-36 | GCCAGAATATGCCGCCGTAGAAGAGTCGCCAA |
| Strand-37 | TATGACAACTCCCTATTATCGGTGGTGAACGT |
| Strand-38 | AAAGACTAACGAAAGCGGCAGACATAATTGAC |
| Strand-39 | ATCTCCGGTAAACATGGTATTGACGCTATCTT |
| Strand-40 | CCGTTTGCAGAAGCCAGGGAATCGCCCACTCC |
| Strand-41 | TTACACATGCGTTCGTTATACTGATTTTCTCC |
| Strand-42 | TGGCGTCCTAGTCTGAATCTGCTGTATAATCT |
| Strand-46 | TCACCCTAGTTTTGTAAGAGTCCTGTCCATAA |
| Strand-48 | ACCTCCTGGGCTGCTGATAGTTCCTCTCTGAT |
| Strand-49 | TCCTGTACGATGGCGATATAACCTCCTAACTG |
| Strand-50 | GTTGAGTGGTGAATGGGCCTGAATAGTCGGTT |
| Strand-51 | GTAGTAGTATCCATTAAGTTCTGTATCATCAA |
| Strand-52 | TAGGGACCAAATCATGCTGTGCGACCGAGGCG |
| Strand-53 | CCACGGTAGCCACTGTCTGTATTATGAAGCGG |
| Strand-54 | GCCTCCTAATGATGCTATACAAACAGTCGCGG |
| Strand-55 | GCGTGGAGAAGCGTCGTTATACTAGCCTGCAT |
| Strand-56 | CTAATTTGAGATCGTTTTGACTATGGGAGGCA |
| Strand-57 | TGTCGGAGGCCACGCTTAGTCACGCAACCAAC |
| Strand-58 | CGTATGGCGGGATTGTGCGGCGGACCTGGGCG |
| Strand-59 | TTCATGTATCAAGGAGTTAAAAGTCTGTATCG |
| Strand-60 | AGGTCACAAATCTCGGCCACAACACTACACTC |
| Strand-61 | TCTCAGCATCTCTATATTTTAAGTACACCACA |
| Strand-62 | TACACAGGCTTACCAGGGACGTTCTCTTATCG |
| Strand-63 | GTTATGCAAAGGTTCAATCCAAATCGTCAAAA |
| Strand-64 | CCAGTCGCTACGTTGTTATTTTCCGGGCCATG |
| Strand-67 | TAGAAGCATCCCATCAACAAAGAACGTATTAG |
| Strand-68 | TACATTTCGGGTGGCGGGTCGGCAGCTCCTCA |
| Strand-70 | ACGAGTCCTTATAGCACGCCATCTTACCGCAG |
| Strand-72 | CAAACCGTGGCAGTGATACCCTAGATGGAGAG |
| Strand-73 | CTGCTAACGTAACAAGGAAAGAAATATGATGA |
| Strand-74 | GATTCTGGTACACGATTTCATTGGGTTAGCAC |
| Strand-75 | TACTGCGGATGGAGCGACGAACTTGGACCTTA |
| Strand-76 | TACTGTCTAACGGGTCAAAGGGCTGCTCGGAT |
| Strand-77 | GAAAGACAAACGCTAAGGTATCGGAATCAAGT |
| Strand-78 | GATCGGTCCTTCTAGTTCGGCAGTACGGTTCA |

| | |
|---|---|
| Strand-79 | CGGGACAAACGCTCTTAACGGGACTCTATGCC |
| Strand-80 | GCTCGGACTCAGACGCGCTTAAAATGGTGCCT |
| Strand-81 | CCGATTTATAGACTTCCGTTACAGAGCACAGC |
| Strand-82 | TTAGCAACGGGTCACGGATGTCGCAGATGTCG |
| Strand-83 | CACATCCATCCCAGACGTAATCGGTTGAGAGA |
| Strand-84 | AGTCCATCGGCGCGGTAGTTCACGGTCAGTCA |
| Strand-85 | GGCAGCTTTATCCGAACCCTCACTCCGTCAAC |
| Strand-86 | ACACACGCGGGTCATGCCAAAGCCAGTCCAAT |
| Strand-87 | TACAGTGATTCTTGATTATGGCTTGGACGGCT |
| Strand-91 | CTGTGGGAGCGATAGGTAATGTGATGTTGCCG |
| Strand-93 | ATGGATACAATGGGATGAGTCTTACAGCTTCT |
| Strand-94 | TTCACAAGCCCTAAAGTACGTCGCCTATATGC |
| Strand-95 | ATGCGGCCCTGTGAATTTAACGACTGATCCAA |
| Strand-96 | CGCTGTTACCCAATACGAGAGATCCCTACGCT |
| Strand-97 | TTAGAAAGTCATTATCAAAGGTACACAGCGAG |
| Strand-98 | ATCAGTTAAGTCGAAGGAAGAGCCCGCCAGTT |
| Strand-99 | GAATAGAACGCGCAGCTTAATGATCCCGGCCG |
| Strand-100 | ACTATTGGCCCGCACGTTGTATCGCTAAGACT |
| Strand-101 | TATATTCTAAAACTCGTGCGGTTCAGGCGGCG |
| Strand-102 | CAGTTTTCCATGATCGCGCAAGAGAGTATAGT |
| Strand-103 | CCTGGCTGGCTAGACAAACTCGCGGGCAATAC |
| Strand-104 | TATATGTAATGAATGTGCTCCTCCAGTAGATG |
| Strand-105 | AGTAAACCAGCGGAGTCACCCATCAGGCCATT |
| Strand-106 | CCGCGACGTTCATCACTCAGGCTAATTTCCAC |
| Strand-107 | AGGGACCACTGCTAGGGCGTTCGACAATTACC |
| Strand-108 | ACATTGCGCTCATCACCTCGTCTAGTACCGGA |
| Strand-109 | TGGCGCTTGGACTACTTCATGGGATACACGAC |
| Strand-112 | GTCCCTAGAAATCCTCGTTGAATCCGCCGTCG |
| Strand-113 | CCCATAACAGGCATACGGGAGGTGAGAATTTT |
| Strand-115 | TGATCTGACGAGCGCCAATTCTGTTCTGAGCA |
| Strand-117 | GATAGGTGACAAGGATAAAGGCTGAATACTGC |
| Strand-118 | ACACAGCTATGCGGGTCCTTGATGCGTCCACT |
| Strand-119 | TGAGATATTGAGTACGGTGTTTGAAGTTGTTA |
| Strand-120 | GATACGTGAAAGAGATGGGTTGAGTCGAAATT |
| Strand-121 | GAACATCGTCTGAAAGTAGGAACATTATCTGA |
| Strand-122 | TGCAAATCAGGAGCTACTACTATATCTTAATG |
| Strand-123 | ACAAGAACGGGAATACGACGAATCAAAGCAAG |
| Strand-124 | GACAGACCCTACCCGACTTCACCCGTGTTGGA |
| Strand-125 | AGCCGACCGTGGGATGTACTTCTGCCTGAGCC |
| Strand-126 | TATCCTTGGTGTAGCGGTAAACTCAGAAAATG |
| Strand-127 | TATAAGCAGTGTGTAACCTAGCCGCGTAAATG |
| Strand-128 | CTAAACGGGTGCCTTCCTACAGAGATTCATTC |
| Strand-129 | GCAGTTCGTGTCGCAAATACCTCGTCATCGTT |
| Strand-130 | ATTCTATTATTACATTATATTAAACCTGCGTG |
| Strand-131 | AGACTGCGACGATTACTAGAGACTTCATTTGT |
| Strand-132 | CGACGACAAGGCCATACTGCTGCTAAACTGCG |
| Strand-136 | GGTAAAGCCAGTCGATTCAGCGCCATAGATAA |
| Strand-138 | TGTCGAGCTTGATTAGGGCAGTCCATTCCACC |
| Strand-139 | CCGTCTAAGGGAAAGGAGTGCTGCCGTCATGG |
| Strand-140 | ATGTTAGAGAATGCCGTATCAATCGTTTTTTA |
| Strand-141 | AATCGAATCCAGTCAGTGTCTCTTTAGTGCCG |
| Strand-142 | CATTTATCGCCCGCAGAGTCAGTTGGTCGTCC |
| Strand-143 | TTCACGCCATTACGCAACGCTTACTTCAGACT |
| Strand-144 | CAAGAGGCTAACGGATGATAAAACTAAAATTC |
| Strand-145 | GCAATCGTAAAGGCGGGTCAAGCTAATCACTA |
| Strand-146 | CAATCAAAAGGCGTCGTGTGGTCAGCGGAGGG |
| Strand-147 | AGCGTATAGCCAATCCCTCGTTCACTCCACTG |
| Strand-148 | AGTATCTCTTGTCTAATCCCTATCTGGTAAAA |
| Strand-149 | CAGGGTAGAAACAAAATCCATGCTCGCTCGCT |
| Strand-150 | CTAGCATATCTTACTGTCAACCACTGTCTTTA |

| | |
|---|---|
| Strand-151 | GATCTGTTTAAGCATAGTCGGCGCGTGTCCCA |
| Strand-152 | TTGAGGAGCCGAGCGCAGCCACCAGAGCCATG |
| Strand-153 | GTATAGGTTAAGCCATGCGTTCAATCAGGAGT |
| Strand-154 | TGAAATACCAAAACAGAACAAACTCGCGTCGT |
| Strand-157 | TTGTCACTATATTAAACGGTCATGCAGCGGCC |
| Strand-158 | CAACTATTTAAGCTATCGGCCATTCTACTCTA |
| Strand-160 | GACCGTACATGGCTGGCGTTCACCCTCGATCC |
| Strand-162 | GAAGGTCAGCTCCCGTCCCGCAAGGCCACACT |
| Strand-163 | GGTCGTTGAGCATCCTTGAGTTGGCAGTAAAA |
| Strand-164 | CCTTCGGGCGGCTCTCCTTCTCGGGTTGGCAC |
| Strand-165 | GCCGCGCCTCAAACTATGTGTAAATCCGTGCA |
| Strand-166 | TGAGTGATCGAAGTAATAAAGCAGCATGGTCT |
| Strand-167 | TTGTAACCTCGTAGCCAGTCGGATAGAAGAAG |
| Strand-168 | GGGCGGAAGCGATCTCTAAGCGACCACGGACT |
| Strand-169 | CTCCAGGCTAAATCTTCCGCTTGTTGCAGGTG |
| Strand-170 | TCTTCGCAAATTCGGGCCTTAACGTAGTCAGC |
| Strand-171 | TATCCGCCAATGACACTTCGAGCTGCACGCCT |
| Strand-172 | TGAAGGACTAAAAGCACTCTATGTTGGTACTG |
| Strand-173 | ACGGAGGACTGGTGTTCATCGGACGGCATCGC |
| Strand-174 | AAGTCCTATACAAGCCCGCCTGCAATGAGAAA |
| Strand-175 | GTATCGTAGCGCCCGTGAATGACAGCCGTAGG |
| Strand-176 | GAAACAGAGTCCAGCAAATCGCGGCAAGTACG |
| Strand-177 | CACCTTCGTTATTATTCGGATTGCGGCCTGGG |
| Strand-181 | CAAGGTGAGGACAACAGCGCCCGGATCTAAGT |
| Strand-183 | TCTAAATGCTGACTGCGCCGGACCCTTGCCTT |
| Strand-184 | GATAACGTCTAATGAGCTTCCCAGGCGCAGGC |
| Strand-185 | TCTCATCTTAATCAGCTTGGCCTCGAATACTC |
| Strand-186 | ACACTCACGGGTAAGCGGACATCAACGGGCAT |
| Strand-187 | GTCAGGTCGCCGGATGCAGCCCACTTGTACGT |
| Strand-188 | ATTGGTAGGTAAAAACAGCCGATTCTCATTCC |
| Strand-189 | GCAAATTACGAACACGGACGAAACTGGAGCTG |
| Strand-190 | AACGTGCTAGAGAGACTTATGCTTCCAGTGCG |
| Strand-191 | TTGATGGTCCTTCTATTGTGCCCGATGCGCTG |
| Strand-192 | CCGTACCGCTAGTGTGCTCGCTCGTGTTCTTG |
| Strand-193 | GTTTCACTACTAAGATGCAGGACAATTCGTTC |
| Strand-194 | TCCTATCCTTTCAGAGCATTGTTGGGCGAGAG |
| Strand-195 | GATGCAACTGTGTCCCGTACCCGCGCCTGATG |
| Strand-196 | AGAGATTTACGTGATGATTAACTATATGAATT |
| Strand-197 | GTCCTCTATGAACGGCCCACTCTGTCTCTACG |
| Strand-198 | ATTTAGGCGCAACCACTAGGCAAGGCTGACCC |
| Strand-199 | TCACGTATGTAGACCACTTTAGCCCGATCCGC |
| Strand-202 | TTGCCTTACTACTCGGGATATTTGCCTACATT |
| Strand-205 | TCGCGCACGGGACTGGGAGGGTAGGACACACT |
| Strand-206 | CCCAAGAACTCCCAGCGGCGATGTGATGCGTC |
| Strand-209 | GCCCGAGTACCGCGAGCCCGAGAGTTGGACAC |
| Strand-210 | GCTCATACAAGACCCTAAATACGAAGATACGA |
| Strand-211 | TGGGTTTTGGATTTGGTTGGACATACTTCATA |
| Strand-214 | ATAGGACCAGGCGGCTCGATGTTGTGTGAAGG |
| Strand-215 | GCATACCTAACGGCTCTTGGACGAACTGGACT |
| Strand-216 | GAGCACCCTGTCCACCAAAATGTCTACGCCGC |
| Strand-219 | GACACGCCTGGACACTACATGATAACCCTACT |
| Strand-220 | CATCGACCACTAGCTGTACGCGCATAAAAAGA |
| Strand-221 | ACTGTGCCCTGGAAATAATGCTGCCCTGCGGT |
| Strand-227 | TTTTTTTTGTTATAGGTTGAGCGATTTTTTTT |
| Strand-230 | TTTTTTTTCTTACTCGGGACGCCATTTTTTTT |
| Strand-233 | TTTTTTTTGGGAGGCTTTGTCATATTTTTTTT |
| Strand-236 | TTTTTTTTCATATTGTAACTTTCATTTTTTTT |
| Strand-239 | TTTTTTTTCCACCCGCAATAGCTCTTTTTTTT |
| Strand-240 | CAGCGTGGTTTTTTTTTTTTTTTTGGTCGGTG |
| Strand-241 | CCTGCGAACGAAGCGAGAACTGAGACGGCCCA |

| | |
|---|---|
| Strand-243 | TTTCCCTTGTGAGTGATTGAGTTGACCAGTTC |
| Strand-244 | GCGAGACCCACGACCCACACTTGAGAGGACAG |
| Strand-245 | TTTTTTTTGGCGACATTGCTTCTATTTTTTTT |
| Strand-246 | ATACACCGTTTTTTTTTTTTTTTTGATGAGGG |
| Strand-247 | ACGTAGTGATGCTTGATGTGACCTTATAGAGA |
| Strand-249 | AGATGACGTGCCCGAACCTGTGTATGAACCTT |
| Strand-250 | CCAACCGCGTATTTCGTCGAGGGCAGATCCAG |
| Strand-251 | TTTTTTTTAGATACTTGCGACTGGTTTTTTTT |
| Strand-252 | TTAATACCTTTTTTTTTTTTTTTTCTTCGCGC |
| Strand-253 | GGCTCACTCCGAATGTCTCCACGCAACGATCT |
| Strand-255 | TCTTCTACGTACAGTGCTCCGACAACAATCCC |
| Strand-256 | CTTGGCATTTGGCGACCGGCGACAGTGAGACG |
| Strand-257 | TTTTTTTTACTCGCTTTACATGAATTTTTTTT |
| Strand-258 | AATCAGTGTTTTTTTTTTTTTTTTCAATGCTT |
| Strand-259 | GTCAATACCCTCGCCTCACTCAACTAATGGAT |
| Strand-261 | TCAGTATATTTCATGCGGTCCCTAACAGTGGC |
| Strand-262 | CACCGATAGGAGAAAAACGCATAATACGCTTG |
| Strand-263 | TTTTTTTTACGTTCACTAGGAGGCTTTTTTTT |
| Strand-264 | TGTCTGCCTTTTTTTTTTTTTTTTGAATAACG |
| Strand-265 | GAGCCGTGGTCAATTACTACTGCCTACAAAAC |
| Strand-267 | GCGTTACAGGAGTGGGTTTATAGACAGCAGCC |
| Strand-268 | CAGCAGATCGAAGAAGTTTGACATACAGGAAG |
| Strand-269 | TTTTTTTTAGATTATAGTACAGGATTTTTTTT |
| Strand-270 | TACCGACGTTTTTTTTTTTTTTTTGCTCTCTC |
| Strand-271 | AGACCTAAACCCTAACGCGGCAAACATAAAAG |
| Strand-272 | GTCAGCCAATGTGCCGTCCCTCCCGCGCAGTG |
| Strand-273 | AAGTCCGATCCAATGCTGAGGCGTAGTAGGCG |
| Strand-274 | AGGTTATATTTAGTTCGGCGGGCATTGAGCGT |
| Strand-275 | TTTTTTTTCAGTTAGGCCACCGTCTTTTTTTT |
| Strand-276 | ATTCAGGCTTTTTTTTTTTTTTTTGCTTTCGT |
| Strand-277 | AGGACTCTAACCGACTTGGATGTGACCGCGCC |
| Strand-278 | TCGCACAGTTATGGACCCGGAGATTGGCTTCT |
| Strand-279 | GGAACTATCGCCTCGGAAGCTGCCCATGACCC |
| Strand-280 | GTTTGTATATCAGAGAATGTGTAATCAGACTA |
| Strand-281 | TTTTTTTTCCGCGACTTCACTGTATTTTTTTT |
| Strand-282 | TAGTATAATTTTTTTTTTTTTTTTCCATCGGT |
| Strand-283 | ACAGAACTATGCAGGCGACCGATCAAGAGCGT |
| Strand-284 | CGTGACTATTGATGATTTCGCAGACTACTAAC |
| Strand-285 | TAATACAGGTTGGTTGGTCCGAGCGAAGTCTA |
| Strand-286 | ACTTTTAACCGCTTCAATTCTGGCATAGGGAG |
| Strand-287 | TTTTTTTTCGATACAGGTTGCTAATTTTTTTT |
| Strand-288 | TGTTGTGGTTTTTTTTTTTTTTTTGAGTGGAA |
| Strand-289 | ATAGTCAAGAGTGTAGGTTAGCAGATCGTGTA |
| Strand-290 | GAACGTCCTGCCTCCCGGTGATATGTCGATGC |
| Strand-291 | TCCGCCGCCGATAAGACCGCAGTAGACCCGTT |
| Strand-292 | GGAAAATACGCCCAGGTTGCTAAGTGTCTCAT |
| Strand-293 | TTTTTTTTCATGGCCCTGTCTTTCTTTTTTTT |
| Strand-294 | CGCTACGGTTTTTTTTTTTTTTTTGCTTTACG |
| Strand-295 | ACTTAAAAACGCACTAGAAATGTACTGGGACG |
| Strand-296 | CGGGAGGGTGTGGTGTGCCAACATCCTGCCGC |
| Strand-297 | ATTTGGATCGCTACATGGACTCGTTAATATGG |
| Strand-298 | TTCTTTGTTTTTGACGGCTTGCGGACTTACCA |
| Strand-299 | TTTTTTTTCTAATACGACGGTTTGTTTTTTTT |
| Strand-300 | CCGGCCTATTTTTTTTTTTTTTTTAACACATT |
| Strand-301 | ATCACGCTGTCTGCTATCAAAATCTGGTCACG |
| Strand-302 | CCGGCATCTGGCTCTCTATCGCATCTTGACAT |
| Strand-303 | CACTCTCTGAAGGGAAACGACACGCATTAGGG |
| Strand-304 | TGGGCACACTCGCACGGCTTTCGCTGATGGGA |
| Strand-305 | TTTTTTTTACAATCAACTAGGGACTTTTTTTT |
| Strand-306 | TGCCGACCTTTTTTTTTTTTTTTTCCGAGATT |

Strand-307 CCAATGAATGAGGAGCGGTTTACTGTGATGAA
Strand-308 AGATGGCGGTGCTAACTGCTGAGACTGGTAAG
Strand-309 AGCCCTTTCTGCGGTATGGTCCCTGTGATGAG
Strand-310 CTAGGGTAATCCGAGCTGCATAACACAACGTA
Strand-311 TTTTTTTTCTCTCCATAAGCGCCATTTTTTTT
Strand-312 TTTCTTTCTTTTTTTTTTTTTTTTCGACGCTT
Strand-313 GTCCCGTTTCATCATACCAATAGTCGAGTTTT
Strand-314 AAGTTCGTGGCATAGACAAATTAGAGCGTGGC
Strand-315 CTGTAACGTAAGGTCCGAAAACTGTGTCTAGC
Strand-316 CCGATACCGCTGTGCTGCCATACGCTCCTTGA
Strand-317 TTTTTTTTACTTGATTTACATATATTTTTTTT
Strand-318 ACTGCCGATTTTTTTTTTTTTTTTCCATTCAC
Strand-319 CGTGAACTTGAACCGTGGCCGCATGTATTGGG
Strand-320 TTTTAAGCTGACTGACACTACTACCATGATTT
Strand-321 GGCTTTGGAGGCACCACTTTCTAACTTCGACT
Strand-322 GCGACATCATTGGACTTACCGTGGAGCATCAT
Strand-323 TTTTTTTTCGACATCTTTCTATTCTTTTTTTT
Strand-324 CCGATTACTTTTTTTTTTTTTTTTCTGGACCG
Strand-325 TCGTAATCTCTCTCAAACAGTCGGCCTATCGC
Strand-326 AGTGAGGGTGCGTCCGTAGGGTGAGAGTTTAG
Strand-327 ACAGGCAGGTTGACGGCTCCGGCGATCCCATT
Strand-328 AAGCCATATCATCAGCCAGGAGGTTCGCCATC
Strand-329 TTTTTTTTAGCCGTCCCTTGTGAATTTTTTTT
Strand-330 TACAGTCATTTTTTTTTTTTTTTTCGGCAACG
Strand-331 ATTATACGGGTCGCCTCAACGCAACATGGTGG
Strand-332 GCAGCAGCCTACAGCAGTCCACAGTTCTAAGT
Strand-333 GGGCAGGGCGTCTCCACCTCTCCGTCTAATGT
Strand-334 GCGACGTAGGTTTTAATCAATCCGAGTTCCAT
Strand-335 TTTTTTTTGCATATAGCTTCACGATTTTTTTT
Strand-336 GTCGTTAATTTTTTTTTTTTTTTTGTCTGGGA
Strand-337 TCACATTATTGGATCACCGTTTAGTTGCGACA
Strand-338 GTACCTTTCGGCAACAGATGGACTTTCGGATA
Strand-339 TAAGACTCCTCGCTGTAATAGAATGTAATCGT
Strand-340 ATCATTAAAGAAGCTGGCGTGTGTATCAAGAA
Strand-341 TTTTTTTTCGGCCGGGTGTCGTCGTTTTTTTT
Strand-342 CGATACAATTTTTTTTTTTTTTTTACTAGAAG
Strand-343 GATCTCTCAGTCTTAGGTTCTTGTTCGGGTAG
Strand-344 CTCTTGCGAGCGTAGGTTGTCCCGGCGTCTGA
Strand-345 GGCTCTTCACTATACTGGTCGGCTCGCTACAC
Strand-346 GGAGGAGCAACTGGCGTAAATCGGCGTGACCC
Strand-347 TTTTTTTTCATCTACTTGCTTATATTTTTTTT
Strand-348 GATGGGTGTTTTTTTTTTTTTTTTCTTGTTAC
Strand-349 GAACCGCAAATGGCCTAGCTGTGTCGTACTCA
Strand-350 TCGAACGCCGCCGCCTCCAGAATCCGCTCCAT
Strand-351 CGCGAGTTGGTAATTGCACGTATCCTTTCAGA
Strand-352 TCCCATGAGTATTGCCAGACAGTATTAGCGTT
Strand-353 TTTTTTTTGTCGTGTAGATTTGCATTTTTTTT
Strand-354 AGCACACGTTTTTTTTTTTTTTTTCGCCACCC
Strand-355 TAGCCTGAAGCACGGAGTTATGGGAAGACAAT
Strand-356 GAACCGCCGTGGAAATCTCGAACCTGCTATAA
Strand-357 TAGACGAGAGTATTTTTCAGATCATTGTGTGG
Strand-358 GATTCAACTCCGGTACTTTCGCTGTCACTGCC
Strand-359 TTTTTTTTCGACGGCGCACCTATCTTTTTTTT
Strand-360 TTACGAGCTTTTTTTTTTTTTTTTGGTTCTGG
Strand-361 TAGACTTTAGAATTAGCGGAACGGAACATACA
Strand-362 GGTTTTTCCTGGTTCCCGCACTCGACACATTG
Strand-363 CCGACTTATGACGCTGTGATGTAGGGCTGTAG
Strand-364 CTGGAACTACGCGCTAAGAACATTGAGGATTT
Strand-365 TTTTTTTTCTTTTATGAGTGACAATTTTTTTT
Strand-366 CACCTCCCTTTTTTTTTTTTTTTTACTCCGCT

Strand-367 TCAAACACAAAATTCTTATGCTAGTATGCTTA
Strand-368 ACAGAATTTAACAACTCGTCGCGGCCTAGCAG
Strand-369 TGTTCCTATGCTCAGACTCCTCAAATGGCTTA
Strand-370 CAGCCTTTTCAGATAACGCAATGTAGTAGTCC
Strand-371 TTTTTTTTGCAGTATTGTATTTCATTTTTTTT
Strand-372 CATCAAGGTTTTTTTTTTTTTTTTCGTGCGGG
Strand-373 GGGTGAAGAGTGGACGACGATTGCCGACGCCT
Strand-374 CTCAACCCTCCAACACAGAATATACGATCATG
Strand-375 GAGTTTACAATTTCGATATACGCTTTAGACAA
Strand-376 TATAGTAGCATTTTCTCAGCCAGGACATTCAT
Strand-377 TTTTTTTTCATTAAGACTACCCTGTTTTTTTT
Strand-378 GATTCGTCTTTTTTTTTTTTTTTTATTCACAG
Strand-379 CGAGGTATCTTGCTTTTCTAACATCTGACTGG
Strand-380 CAGAAGTAAACGATGATAACAGCGGATAATGA
Strand-381 AGTCTCTAGGCTCAGGGATAAATGTGCGTAAT
Strand-382 CGGCTAGGACAAATGATAACTGATGCTGCGCG
Strand-383 TTTTTTTTCATTTACGGCCTCTTGTTTTTTTT
Strand-384 CTCTGTAGTTTTTTTTTTTTTTTTCATGGTTT
Strand-385 GGGTAGCGGAATGAATAGAGGTAGATCGACTG
Strand-386 TTTAATATCTTCAACTTCCCACAGGTACATCT
Strand-387 TAATAACTCACGCAGGCTCAAGGGCTAATCAA
Strand-388 AGCAGCAGGGCAACGAGTATCCATCTTTAGGG
Strand-389 TTTTTTTTCGCAGTTTTTAGACGGTTTTTTTT
Strand-390 AGTATTAATTTTTTTTTTTTTTTTGTATGGAA
Strand-391 ACATGGTTCAAGACGTTTCCTGGCGACACACT
Strand-392 TTTTCAAGGTATTCGCTATCAACTGTAACTCT
Strand-393 GGCGGGATGCACCACGACTACAATTTTCTACG
Strand-394 GCAGCACTTTCCAGCGACGACTTCTTTCAGGG
Strand-395 TTTTTTTTCCATGACGATACTTAATTTTTTTT
Strand-396 GATTGATATTTTTTTTTTTTTTTTGAAGGCAC
Strand-397 GGCGCTGATAAAAAACTCCTCCGTGGCTTGTA
Strand-398 AACTGACTTTATCTATCGAACTGCAATGTAAT
Strand-399 GGACTGCCGGACGACCTACGATACTGCTGGAC
Strand-400 GTTTTATCGGTGGAATCGCAGTCTTATGGCCT
Strand-401 TTTTTTTTGAATTTTACGAAGGTGTTTTTTTT
Strand-402 AGCTTGACTTTTTTTTTTTTTTTTGTATTCCC
Strand-403 AAGAGACATAGTGATTTTCCGCCCAAGATTTA
Strand-404 TGAACGAGCGGCACTAGGTCTGTCCATCCCAC
Strand-405 GTAAGCGTCAGTGGAGTGCGAAGAGTGTCATT
Strand-406 AGCATGGAAGTCTGAACAAGGATATTACACAC
Strand-407 TTTTTTTTAGCGAGCGGTCCTTCATTTTTTTT
Strand-408 GTGGTTGATTTTTTTTTTTTTTTTACCCGCAT
Strand-409 TGACCACATAAAGACACAACGACCGAGAGCCG
Strand-410 TGGTGGCTCCCTCCGCATATCTCAATCTCTTT
Strand-411 GATAGGGACATGGCTCGGCGCGGCTTACTTCG
Strand-412 AGTTTGTTTTTTACCACGATGTTCTAGCTCCT
Strand-413 TTTTTTTTACGACGCGGGTTACAATTTTTTTT
Strand-414 GGTGCTTGTTTTTTTTTTTTTTTTGTATGCCT
Strand-415 GCGCCGACGTGCTTGAAATAGTTGGATGACCT
Strand-416 CTGAAACTTGGGACACACGGCATGGGCGCTCG
Strand-417 TTGAACGCGATATTAAGTACGGTCCCTGCTCT
Strand-418 CATGACCGACTCCTGACTGAACTAATCCTTGT
Strand-419 TTTTTTTTGGCCGCTGTGACCTTCTTTTTTTT
Strand-420 TAGAGCAATTTTTTTTTTTTTTTTCCCAGTGA
Strand-421 ATGATGATTAACCACCCACCGCTGAGGACCGT
Strand-422 CCGTAATGACCCTATATCGACAGACCGCGCTC
Strand-423 TAACTCTAGGCTGGCATGTGAAAACTCTGACT
Strand-424 GCCTGCTGACACCGCCGCTCCACTTTTAATAT
Strand-425 TTTTTTTTCACGATACTAAGGCAATTTTTTTT
Strand-426 AATGGCCGTTTTTTTTTTTTTTTTCAGTAAGA

| | |
|---|---|
| Strand-427 | CCGAGAAGTAGAGTAGGTTGCATCCATCACGT |
| Strand-428 | GGTGAACGGTGCCAACAACAGATCGCGCTCGG |
| Strand-429 | CTGCTTTAGGATCGAGTAGAGGACGTGGTTGC |
| Strand-430 | CTTGCGGGAGACCATGACCTATACCTGTTTTG |
| Strand-431 | TTTTTTTTAGTGTGGCATACGTGATTTTTTTT |
| Strand-432 | CCAACTCATTTTTTTTTTTTTTTTCCGCCTTT |
| Strand-433 | ACAAGCGGTTTTACTGAGCACGTTATAGAAGG |
| Strand-434 | TTTACACACACCTGCATTTGATTGGGATTGGC |
| Strand-435 | AGCTCGAATGCACGGACGGTACGGATCTTAGT |
| Strand-436 | ATCCGACTAGGCGTGCGAGATACTTTTTGTTT |
| Strand-437 | TTTTTTTTCTTCTTCTGGATAGGATTTTTTTT |
| Strand-438 | GTCGCTTATTTTTTTTTTTTTTTTCGGCATTC |
| Strand-439 | TGCAGGCGAGTCCGTGAGATGAGAGCTTACCC |
| Strand-440 | CGTTAAGGTTTCTCATATTCGATTCTGCGGGC |
| Strand-441 | CCGCGATTGCTGACTAGACCTGACGTTTTTAC |
| Strand-442 | ACATAGAGCGTACTTGGGCGTGAAATCCGTTA |
| Strand-443 | TTTTTTTTCAGTACCATAATTTGCTTTTTTTT |
| Strand-444 | GTCCGATGTTTTTTTTTTTTTTTTAAGATTCA |
| Strand-445 | TTTCCTGAGCGATGCCTAGCCGTATGTTGTCC |
| Strand-446 | TGTCATTCTACTTTACGCTTTACCATATATTA |
| Strand-447 | TTCTCGCACCTACGGCCTATCTAAGCAGTCAG |
| Strand-448 | GCAATCCGGCTGGCTGGCTCGACACCTTTCCC |
| Strand-449 | TTTTTTTTCCCAGGCCACGTTATCTTTTTTTT |
| Strand-450 | ATTGTAGCTTTTTTTTTTTTTTTTGCACCGCG |
| Strand-452 | GGAACTAATGCACCTCGATGACACGGGCTGAT |
| Strand-454 | CTGGGAAGACTACTATGACTGGACGGGATTTG |
| Strand-455 | TTTTTTTTGCCTGCGCCTTGGAACTTTTTTTT |
| Strand-456 | GAGGCCAATTTTTTTTTTTTTTTTAACACCAG |
| Strand-458 | GTGGGCTGACTTAGATTAGGACTTACGGGCGC |
| Strand-460 | GTTTCGTCAAGGCAAGTCTGTTTCAATAATAA |
| Strand-461 | TTTTTTTTCAGCTCCATTCCACGATTTTTTTT |
| Strand-462 | AAGCATAATTTTTTTTTTTTTTTTGAGATCGC |
| Strand-464 | CGAGCGAGATGCCCGTGCCTGGAGCCCGAATT |
| Strand-466 | CAACAATGGGAATGAGGCGGATATGCTTTTA |
| Strand-467 | TTTTTTTTCTCTCGCCGTATCTCATTTTTTTT |
| Strand-468 | GCGGGTACTTTTTTTTTTTTTTTTAGGATGCT |
| Strand-470 | CAGAGTGGCAGCGCATCCCGAAGGTAGTTTGA |
| Strand-472 | GGCTAAAGGAACGAATATCACTCAGGCTACGA |
| Strand-473 | TTTTTTTTGCGGATCGTAACTGACTTTTTTTT |
| Strand-474 | ACCCGCCGTTTTTTTTTTTTTTTTATAGCTTA |
| Strand-476 | AAACTCCTAATTCATACTAAACTTCCAGCCAT |
| Strand-478 | CAAATATCGGGTCAGCGGAAATGCACGGGAGC |
| Strand-479 | TTTTTTTTAATGTAGGTAGCAATCTTTTTTTT |
| Strand-480 | GGACTCACTTTTTTTTTTTTTTTTCATCGCTT |
| Strand-483 | CAGCTACGTTTTTTTTTTTTTTTTGGGACACA |
| Strand-485 | TGAAACGATATGAAGTGCCTAAATTGGTCTAC |
| Strand-486 | ATAATCGGTTTTTTTTTTTTTTTTGTCTCTCT |
| Strand-488 | TGGAATCTGCGGCGTAAGTGAAACCTCTGAAA |
| Strand-489 | TGATATACTTTTTTTTTTTTTTTTGCTGATTA |
| Strand-491 | GCTCCAAGACCGCAGGCTACCAATCGTGTTCG |
| Strand-492 | GAGGGATGTTTTTTTTTTTTTTTTCTTAGTTG |
| Strand-494 | TTCAGGTAGGTAGCTTCATTTAGACTCATTAG |
| Strand-1305 | ACCTGTGACTTCGGAG |
| Strand-1307 | AGCGTGCCCGGCCCTC |
| Strand-1309 | CCACGCTGTCGCTTCG |
| Strand-1312 | ACGCCTACGAGAGAGC |
| Strand-1319 | TAGGCCGGTAGCAGAC |
| Strand-1322 | TTTGCCGCCGTTGCCG |
| Strand-1329 | GCTCGTAACTAATTCT |
| Strand-1332 | TTGCGTTGTTCCATAC |

| | |
|---|---|
| Strand-1339 | TTGCTCTAGGTGGTTA |
| Strand-1342 | GCCAGGAACGCGGTGC |
| Strand-1349 | GTGAGTCCTTATACCG |
| Strand-1357 | GTACAATCGGTTTCGG |
| Strand-1361 | ACAACAGTCTCGCGCC |
| Strand-1365 | AGACACGTTGGACCTC |
| Strand-1369 | TCCACTTCAACATTCC |
| Strand-1373 | CCCAGCGCGAGGGCCG |
| Strand-1375 | GCAAAGCCCGGTATAA |
| Strand-1379 | CTCTCGGGTATCCAGG |
| Strand-1383 | CAACATCGCATCTAAT |
| Strand-1387 | TATCATGTTGGGTTCC |
| Strand-1391 | CTGACCGAGGCAACCG |
| Strand-1393 | TACATTGGGCCTTGGG |
| Strand-2207 | GATTGTACCGTTATTCTTTTTTTTGGGATGAG |
| Strand-2221 | TTTTTTTTCTTCCTGTGATGCGAGCCTATAAC |
| Strand-2227 | ACTGTTGTAAGCATTGTTTTTTTTGAGCCCTG |
| Strand-2241 | TTTTTTTTCAAGCGTAGTCCCAGCCGAGTAAG |
| Strand-2247 | ACGTGTCTGCGCGAAGTTTTTTTTGCTAGTTT |
| Strand-2261 | TTTTTTTTCGTCTCACAATATACCAGCCTCCC |
| Strand-2267 | GAAGTGGACCCTCATCTTTTTTTTGCTCATAG |
| Strand-2281 | TTTTTTTTCTGGATCTCACACGTCACAATATG |
| Strand-2287 | GCGCTGGGCACCGACCTTTTTTTTAGATACCG |
| Strand-2293 | TTTTTTTTCTGTCCTCGTGCAATAGCGGGTGG |
| Strand-3018 | TCTGACACTTTTTTTTCGTAGCTGCCTGGATA |
| Strand-3036 | GATTGCTAGTGTCGGTTCGTTTCATTTTTTTT |
| Strand-3038 | ACCAACGATTTTTTTTCCGATTATATTAGATG |
| Strand-3056 | GTCAGTTATACACATTAGATTCCATTTTTTTT |
| Strand-3058 | TTTGCGAATTTTTTTTGTATATCAGGAACCCA |
| Strand-3076 | TGAGATACATACTACCCTTGGAGCTTTTTTTT |
| Strand-3078 | TTGGTTTGTTTTTTTTCATCCCTCCGGTTGCC |
| Strand-3096 | TCGTGGAAGTGCTCTATACCTGAATTTTTTTT |
| Strand-3707 | CGCCTCATAGCCCGTCTTTTTTTTCCGCACCT |
| Strand-3789 | TTTTTTTTGACGCATCCTTGACCACCGAGTAG |
| Strand-3826 | GAAATGGACGGAATAAACTTGCTAACACGGAAACTAATACACTTGCTG |
| Strand-3859 | TCAAGTGTCCTGTCATAACCGAGAATCATCCTGCCGCGCCAAATCAAT |
| Strand-3861 | ATGTTGGCAGGAGCTTTTCGCAGGTCCGTAACTTGTAATATCACTCAC |
| Strand-3862 | CCGCAAGCTCTCGTCAAAGGGAAAGGGTCGTGGGTCTCGCATGTCGCC |
| Strand-3894 | CGGGCTACCACTGCGCGGGAGGGAACGGATAACACGGCTCGGGCGGCT |
| Strand-3896 | TCGCTCAAACGCTCAATGCCCGCCTCTAATGGTGTAACGCCTTCTTCG |
| Strand-3897 | GGCAGTAGCGGTCCAGCGTCGGTAGTTAGGGTTTAGGTCTCGGCACAT |
| Strand-3898 | TCTATAAACTAAACTCTGGCTGACGCATTGGATCGGACTTGAACTAAA |
| Strand-3929 | ATGCGATATGGGCCGTCTCAGTTCAATGTGTTCCGTAGCGTAGTGCGT |
| Strand-3931 | GCGAAAGCGAACTGGTCAACTCAAATGTCAAGCCCTCCCGATGTAGCG |
| Strand-3933 | GGTTCGAGCGTCCCAGAGCGTGATGAGAGCCAGATGCCGGTTCCCTTC |
| Strand-3934 | CAGCGAAACCATATTAAGAGAGTGCGTGCGAGTGTGCCCATTGATTGT |
| Strand-3966 | ACGCCTCAACTTAGAACTGTGGACCTTTTATGGATTACGACGGACGCA |
| Strand-3968 | GACGGTGGATGGAACTCGGATTGACGCCTACTCTGCCTGTGCTGATGA |
| Strand-3969 | CCGACTGTAAACCATGTGACTGTAAGGCGACCCGTATAATTGCTGTAG |
| Strand-3970 | CGCCGGAGAGATGTACGCTGCTGCTGGAGACGCCCTGCCCTTAAAACC |
| Strand-4001 | CGAGTGCGCGTGACCAGATTTTGACCAGAACCCGTGTGCTTCCGTGCT |
| Strand-4003 | AATGTTCTCCCTAATGCGTGTCGTCAATGTGTGGCGGTTCAAAATACT |
| Strand-4005 | CATGCCGTATTGTCTTAAAGTCTAGGAACCAGGAAAAACCCAGCGTCA |
| Strand-4006 | TAGTTCAGCCACACAATAAGTCGGTAGCGCGTAGTTCCAGCATAAAAG |
| Strand-4038 | CGGAGAGGAGAGTTACAGTTGATACCACCATGCGCTACCCAGTTGAAG |
| Strand-4040 | TCGTGAAGCCCTGAAAGAAGTCGTACATTAGAAGTTATTATCGTTGCC |
| Strand-4041 | CTACCTCTTGAATCTTTTAATACTACGTCTTGAACCATGTGCGAATAC |
| Strand-4042 | CCCTTGAGTAATATATCTTGAAAACGTGGTGCATCCCGCCCGCTGGAA |
| Strand-4073 | TCTGTCGATGTATGTTCCGTTCCGTCACTGGGCAAGCACCTCAAGCAC |
| Strand-4075 | AGTGGAGCCTACAGCCCTACATCAGAGCGCGGAGTTTCAGTTAATATC |

| | |
|---|---|
| Strand-4077 | AAGTTTAGAGGTCATCATCATCATTATAGGGTCATTACGGTGCCAGCC |
| Strand-4078 | GCATTTCCAGAGCAGGTAGAGTTAGGCGGTGTCAGCAGGCGTATCGTG |
| Strand-4110 | ATTGTAGTATCAGCCCGTGTCATCAGTGTGTCTCAGGAAAGTAAAGTA |
| Strand-4112 | TTAAGTATCAAATCCCGTCCAGTCCGTAGAAATGCGAGAACAGCCAGC |
| Strand-4113 | TACGGCTACAACTAAGGCTACAATGACGGGCTATGAGGCGGAGGTGCA |
| Strand-4114 | TTAGATAGTGTGATAGTTAGTTCCGCCAGGTAGTAGCCCGATAGTAGT |
| Strand-4145 | CCACAACTACGGTCCTCAGCGGTGAAGCGATGCGGCGGGTCTATACGA |
| Strand-4147 | TGGTCAAGAGTCAGAGTTTTCACAAGCTAATAAGGAGTTTCAGATCAT |
| Strand-4149 | GCTTGCTGCGACCTGCGGCTTTGCGACCCACCTAACCTGATGACTGCG |
| Strand-4182 | AATAAGTTCCCAAGGCCCAATGTAAGGTGCGGTCGGTCAGCTCTCCCA |
| Strand-4184 | GTTCCAAGAGTGTCGCGTGCCAGAGATCATGTTAATTCCCAAGCTACC |
| Strand-4185 | TAGCAAGTCTCCGAAGTCACAGGTCTCATCCCGTAGGCGTTTATCCGT |
| Strand-4187 | CTCGCATCCAGCAAGTGTATTAGTTTCCGTGTGTAGCCCGCCATTAGA |
| Strand-4188 | CGATTCCCAGCCGCCCAGCATTTGTTATTCCGTCCATTTCCGAGAGGT |
| Strand-4189 | ATATTCGGTTCAGGATCACGCACGCAGGGCTCTAGTCTTTCATGTTTA |
| Strand-4191 | GCTGGGACGACTTACTTACGACTGGGCCTCACGCAAACGGACGAACGC |
| Strand-4192 | AAGGATTTAAGATAGCCAGAAGCCACACAGACGTTGCGGACCACAGCC |
| Strand-4193 | CAACCTCTGTGTCAATCGGGAAGCAAACTAGCGTGTCAAAATGTCGTA |
| Strand-4195 | GGTATATTTTCGGGTCTCAACAAGGCTCATCTAACGGTCTGGCGGCAT |
| Strand-4196 | CGGCTAGATTTAACCATGCACAACACTTCCTTTCGACACGTACAGATA |
| Strand-4197 | CCAAGCAACTAAGCCTAAGCGGGACTATGAGCGATGTGATTTTCTCTG |
| Strand-4199 | GACGTGTGACCATTTACCAGTGCAGTGTGATTTTTCCCAACCGCCCTA |
| Strand-4200 | GAGAGACGTAAGGATGGCAAAGATGACTGATGTTCGACCTGGCACTAT |
| Strand-4201 | GGCGCGGCACACACAAAGGTCATTCGGTATCTATCTGGGCAAGCTCCT |
| Strand-4203 | TATTGCACCATGAGACTATTAAAGATTGATTTCCTATTGTTGACGAGA |
| Strand-4204 | TATTACAAGTTACGGAGGCACGCTAGGATGATTCTCGGTTATGACAGG |
| Strand-4431 | ACATCGCCCGCAGTCATCAGGTTAGGTGGGTCAGTTGTGGTATTAGCT |
| Strand-4433 | TAGTTAATTCGTATAGGTGTCAGAGCAGGTCGCAGCAAGCCCAGTCCC |
| Strand-4434 | CTTGCCTAATGATCTGGTGCGCGAGCTGGGAGTTCTTGGGACCGACAC |
| Strand-4436 | ATGTCCAAAGTGTGTCCTACCCTCGTGTCCAAAAATCTCTGCCGTTCA |
| Strand-4438 | CGGGCACACATCAGGCTCGTTGGTCTCGCGGTACTCGGGCAGGGTCTT |
| Strand-4439 | TGTCCTGCCGTAGAGAGTATGAGCCCAAATCCAAAACCCAAATGTGTA |
| Strand-4441 | GACATTTTTCGTATCTTCGTATTTCCTTCACAACCATCAACACACTAG |
| Strand-4443 | TGATGTCCCGCACTGGTTCGCAAAAGCCGCCTGGTCCTATGAGCCGTT |
| Strand-4444 | AATCGGCTCAAGAACAAGGTATGCGGTGGACAGGGTGCTCGGTAGTAT |
| Strand-4446 | GCAGCATTAGTCCAGTTCGTCCAAAGTAGGGTGTGAGTGTCATCCGGC |
| Strand-4448 | CCGGGCGCGAGTATTCCAAACCAAAGTGTCCAGGCGTGTCCAGCTAGT |
| Strand-4449 | GGTCCGGCACGTACAAGGTCGATGATTTCCAGGGCACAGTTAGAGCAC |
| Strand-4451 | GGGAATTATCTTTTTATGCGCGTATGGGAGAGTCACCTTGCTATCACA |
| Strand-4454 | CGGGCTACTACCTGGCAACTTATTACATGATCTCTGGCACGCGACACT |

**Table S8.** Unecessary duplexes in the "four-input OR gate" seesaw-gate network reported by Qian *et al.*.

| | **Unnecessary intra-oligomer duplexes (Count)** | | **Unnecessary inter-oligomer duplexes (count)** | |
|---|---|---|---|---|
| **Size** | As-published | Optimized | As-published | Optimized |
| 1 bp | 3004 | 3004 | 182624 | 182624 |
| 2 bp | 308 | 218 | 46094 | 42771 |
| 3 bp | 10 | | 13915 | 12998 |
| 4 bp | 1 | | 4375 | 3422 |
| 5 bp | | | 1438 | 680 |
| 6 bp | | | 635 | 204 |
| 7 bp | | | 319 | 182 |
| 8 bp | | | 283 | 182 |
| 9 bp | | | 85 | 11 |
| 10 bp | | | 20 | |
| 11 bp | | | 16 | |
| 12 bp | | | 15 | |
| 13 bp | | | 14 | |
| 14 bp | | | 14 | |
| 15 bp | | | 14 | |
| 16 bp | | | 14 | |
| 17 bp | | | 14 | |
| 18 bp | | | 14 | |
| 19 bp | | | 14 | |
| 20 bp | | | 9 | |
| 21 bp | | | 6 | |
| 22 bp | | | 2 | |

**Table S9.** New oligomers for the "four-input OR gate" seesaw-gate network design reported by Qian *et al.*.

| Oligo Name | Oligo Sequence (5' - 3') |
|---|---|
| w5,6 | CAACCACAATAATCATCTCACCTCTAACCAACA |
| G5-b | TGAGATGTTGGTTAGAGGTGAGATG |
| w5,7 | CAACTCTATAAATCATCTCACCTCTAACCAACA |
| Th2,5:5-t | CACCTCTAACCAACA |
| Th2,5:5-b | TGTTGGTTAGAGGTGAGATGTGTTGAGTTTT |
| w2,5 | CACCTCTAACCAACATCTCAAAAACTCAACACA |
| G2-b | TGAGATGTGTTGAGTTTTTGAGATG |
| w1,2 | CAAAAACTCAACACATCTCATTCTCCTACACCA |
| G1-b | TGAGATGGTGTAGGAGAATGAGATG |
| w1,10 | CAAACAACTCTTACATCTCATTCTCCTACACCA |
| Th4,1:1-t | CATTCTCCTACACCA |
| Th4,1:1-b | TGGTGTAGGAGAATGAGATGGGTGTTTTAGT |
| w4,1 | CATTCTCCTACACCATCTCAACTAAAACACCCA |
| G4-b | TGAGATGGGTGTTTTAGTTGAGATG |
| w3,2 | CAAAAACTCAACACATCTCAATCCACACTATCA |
| G3-b | TGAGATGATAGTGTGGATTGAGATG |
| w3,11 | CACTTACAAACTACATCTCAATCCACACTATCA |
| Th12,3:3-t | CAATCCACACTATCA |
| Th12,3:3-b | TGATAGTGTGGATTGAGATGAGGATTTTGTG |
| w12,3 | CAATCCACACTATCATCTCACACAAAATCCTCA |
| G12-b | TGAGATGAGGATTTTGTGTGAGATG |
| G8-b | TGAGATGTTATTTGGTGATGAGATG |
| w8,15 | CAAATCTACTCTACATCTCATCACCAAATAACA |
| Th16,8:8-t | CATCACCAAATAACA |
| Th16,8:8-b | TGTTATTTGGTGATGAGATGAAGATTAGGTT |
| w16,8 | CATCACCAAATAACATCTCAAACCTAATCTTCA |
| G16-b | TGAGATGAAGATTAGGTTTGAGATG |
| G17-b | TGAGATGGTAGAAGTTTATGAGATG |
| w17,19 | CAACAACTCTCTACATCTCATAAACTTCTACCA |
| Th20,17:17-t | CATAAACTTCTACCA |
| Th20,17:17-b | TGGTAGAAGTTTATGAGATGGAGTTAGTATG |
| w20,17 | CATAAACTTCTACCATCTCACATACTAACTCCA |
| G20-b | TGAGATGGAGTTAGTATGTGAGATG |
| w21,20 | CACATACTAACTCCATCTCACTCTAAACAAACA |
| w22,20 | CACATACTAACTCCATCTCACTTTCATTTCACA |
| w18,16 | CAAACCTAATCTTCATCTCACTACTCTATATCA |
| w9,4 | CAACTAAAACACCCATCTCACTACAAACAATCA |
| w13,12 | CACACAAAATCCTCATCTCACTCTCTACAAACA |
| w14,12 | CACACAAAATCCTCATCTCACTCTATCTAAACA |
| w8,2 | CAAAAACTCAACACATCTCATCACCAAATAACA |
| w17,2 | CAAAAACTCAACACATCTCATAAACTTCTACCA |
| w23,4 | CAACTAAAACACCCATCTCACTCTCTACAATCA |
| w24,16 | CAAACCTAATCTTCATCTCACTCTCTCTATACA |
| Rep6-t | CAACCACAATAATCA |
| Rep6-b | TGATTATTGTGGTTGAGATG |

**Table S10.** Uneccessary duplexes in the autocatalytic-four-arm-junction network reported by Kotani *et al.*.

| | Unnecessary intra-oligomer duplexes (Count) | | Unnecessary inter-oligomer duplexes (count) | |
|---|---|---|---|---|
| **Size** | As-published | Optimized | As-published | Optimized |
| 1 bp | 4051 | 4051 | 43874 | 43874 |
| 2 bp | 779 | 573 | 10395 | 10272 |
| 3 bp | 157 | | 2516 | 2269 |
| 4 bp | 26 | | 622 | 666 |
| 5 bp | 1 | | 137 | 133 |
| 6 bp | | | 46 | 16 |
| 7 bp | | | 13 | |
| 8 bp | | | 11 | |
| 9 bp | | | 11 | |
| 10 bp | | | 11 | |
| 11 bp | | | 11 | |
| 12 bp | | | 11 | |
| 13 bp | | | 7 | |
| 14 bp | | | 3 | |
| 15 bp | | | 3 | |
| 16 bp | | | 3 | |
| 17 bp | | | 3 | |
| 18 bp | | | 1 | |
| 19 bp | | | 1 | |
| 20 bp | | | 1 | |
| 21 bp | | | 1 | |
| 22 bp | | | 1 | |
| 23 bp | | | 1 | |
| 24 bp | | | 1 | |
| 25 bp | | | 1 | |
| 26 bp | | | 1 | |
| 27 bp | | | 1 | |
| 28 bp | | | 1 | |
| 29 bp | | | 1 | |
| 30 bp | | | 1 | |
| 31 bp | | | 1 | |
| 32 bp | | | 1 | |
| 33 bp | | | 1 | |
| 34 bp | | | 1 | |
| 35 bp | | | 1 | |
| 36 bp | | | 1 | |
| 37 bp | | | 1 | |
| 38 bp | | | 1 | |
| 39 bp | | | 1 | |
| 40 bp | | | 1 | |
| 41 bp | | | 1 | |
| 42 bp | | | 1 | |
| 43 bp | | | 1 | |

| | |
|---|---|
| 44 bp | 1 |
| 45 bp | 1 |

**Table S11.** New oligomers for the autocatalytic-four-arm-junction network reported by Kotani *et al.*.

| Oligomer | Oligomer Sequence (5' -> 3') |
|---|---|
| A1x | GGTGTAGCGTGCGTAGAGATGCGTGTGTCAAGGTAAGCGGTAGGTTC GTCCAAAGGTG |
| fA2au | CGCTTACCTTGGACCGGACCTGGGCTGACCTGAACACACGCATCTCTA CGCACGCTACACCTC |
| fA3au | CAGCAGTCCCATTCCCAGCCAGTCAGGTCAGCCCAGGTCCGG TCTTACACACGCATCTCTACGCACGC |
| fA4au | GACGAACCTACCGCTTACCTTGCTGGCTGGGAATGGGACTGCTGCTAC TGCTCTCACTCA |
| B1x | CACCTTTGGACGAACCTACCGCTTACCTTGACACACGCATCTCTACGC ACGC |
| fB2au | GAGGTGTAGCGTGCGTAGAGATGCGTGTGTTCAGGTCAGCCCAGGTC CGGTCCAAGGTAAGCG |
| fB3au | CACCTTTGGACGAACCTACGACGAACCTACGACCGGACCTGGGCTGA CCTGACTGGCTGGGAATGGGACTGCTG |
| fB4au | TTCTCCATCCACATCATTCAGCAGTCCCATTCCCAGCCAGCAAGGTAAG CGGTAGGTTCGTCCAAAGG |
| dye | CTTTCTCCATCCACATCACTACTG |
| quencher | TGAGTGAGAGCAGTAGTGATGTGGATGGAGAAAG |

**Table S12.** Unecessary duplexes in the autotacalytic network reported by Zhang *et al.*.

| Size | Unnecessary intra-oligomer duplexes (Count) | | Unnecessary inter-oligomer duplexes (count) | |
|---|---|---|---|---|
| | As-published | Optimized | As-published | Optimized |
| 1 bp | 843 | 843 | 6657 | 6657 |
| 2 bp | 146 | 67 | 1542 | 1372 |
| 3 bp | 22 | | 328 | 206 |
| 4 bp | 3 | | 77 | |

**Table S13.** New oligomers for the autocatalytic network published by Zhang *et al.*.

| Oligomer Name | Oligomer Sequence (5’ - 3’) |
|---|---|
| Catalyst | ACCATTACTACACGCTTCCACTTATTCAGACGAC |
| Signal | TCTCTATCAACAAACTCCTCACCATTACTACACGCT |
| Backbone | AGTGGAAGCGTGTAGTAATGGTGAGGGTCGTCTGAATAAGTGGAAGCGTG |
| Fuel | CACGCTTCCACTTATTCAGACGACCCTCACCATTACTACACGCT |
| Dye | TCTCTATCAACAAACTCCTC |
| Quencher | AGCGTGTAGTAATGGTGAGGAGTTTGTTGATAGAGA |

## Supplementary Note 6: Completion Time Calculations

Reaction completion times were calculated as follows. These calculations assume stoichiometric reactants and no initial product.

The rate equations for the kinetic model are:

$$S_1 + S_2 \xrightarrow{k_f} D_1 \tag{1}$$

$$\frac{d[D_1]}{dt} = k_f[S_1][S_2] \tag{2}$$

Assuming equal reactant concentrations and no product $D_1$ at initial time $t_0$ implies:

$$[D_1]_0 = 0 \tag{3}$$

$$[S_1]=[S_2] = [S_1]_0 - [D_1] \tag{4}$$

Equation 4 can be substituted into equation 2 to yield:

$$\frac{d[D_1]}{dt} = k_f([S_1]_0 - [D_1])^2 \tag{5}$$

Equation 5 can be integrated between arbitrary time *t* and initial time $t_0$ to yield:

$$\frac{1}{k_f([S_1]_0 - [D_1])} - \frac{1}{k_f([S_1]_0 - [D_1]_0)} = (t - t_0) \tag{6}$$

Where equation 3 can be substituted to yield:

$$\frac{1}{k_f([S_1]_0 - [D_1])} - \frac{1}{k_f([S_1]_0)} = (t - t_0) \tag{7}$$

Which allows one to calculate elapsed time based on known initial concentration $[S_1]_0$, desired final concentration $[D_1]$, and rate-constant $k_f$.

**Table S14**. Elapsed times calculated for select values of initial concentration $[S_1]_0$, final concentration $[D_1]$, and rate-constant $k_f$.

| Data source | $[S_1]_0$ (nM) | $[D_1]$ (nM) | $k_f$ ($M^{-1}s^{-1}$) | $t-t_0$ (s) |
|---|---|---|---|---|
| **RND-1 (10C)** | 10 nM | 5 nM | $9.2 \times 10^3$ | 10870 |
| **RND-2 (10C)** | 10 nM | 5 nM | $6.4 \times 10^5$ | 156 |
| **W-Fit-1 (50C)** | 10 nM | 5 nM | $2.4 \times 10^7$ | 4.2 |
| **W-Fit-3 (50C)** | 10 nM | 5 nM | $1.8 \times 10^7$ | 5.6 |
| **W-Fit-2 (10C)** | 10 nM | 5 nM | $2.6 \times 10^5$ | 385 |
| **W-Fit-3 (10C)** | 10 nM | 5 nM | $2.1 \times 10^5$ | 476 |
