# Supplementary material for: Generation of DNA oligomers with similar chemical kinetics via in-silico optimization
Source: Commun Chem. 2023 Oct 18;6:226. doi: 10.1038/s42004-023-01026-w (PMC10584830; doi:10.1038/s42004-023-01026-w)
Supplement: Supplementary file 3 — Description of Additional Supplementary Files [file 42004_2023_1026_MOESM3_ESM.pdf]

# Description of Additional Supplementary Files

**File name:** Supplementary Data 1

**Description:** Numerical data underlying figures.

**File name:** Supplementary Software

**Description:** Versions 2.0 of the SeqEvo and DevPro computer programs.
