# Supplementary material for: Generation of DNA oligomers with similar chemical kinetics via in-silico optimization
Source: Commun Chem. 2023 Oct 18;6:226. doi: 10.1038/s42004-023-01026-w (PMC10584830; doi:10.1038/s42004-023-01026-w)
Supplement: Supplementary file 5 — Supplementary Software 1 [file 42004_2023_1026_MOESM5_ESM.zip › doc/DevPro_Manual.pdf]

USER’S MANUAL FOR DEVPRO

SYSTEM REQUIREMENTS

DevPro requires a Java Development Kit (such as the one at https://openjdk.org/) to be installed. The current code has been verified to run with openJDK 21 (https://jdk.java.net/21/). Instructions for installing openJDK can be found here (https://stackoverflow.com/questions/52511778/how-to-install-openjdk-11-on-windows).

INPUT FILES

Running DevPro requires the following 4 input files: (1) A “fixed domains” file, (2) a “variable domains” file, (3) an “oligomers” file, and (4) a “parameters” file.

Fixed domains file. A file specifying the base-sequence of domains which are not free to be modified during optimization. Any duplex involving only fixed sequences is part of a networks design and is considered necessary (i.e., they do not contribute to certain fitness scores). By default, this file is named “dp_in_domains_fixed.txt” and is present in the directory where the program is run. A different file can be specified by providing a new value for IN_FIXED_DOMAINS in the parameters file. This file must contain one line per domain. Each line should contain (in order) a name for the domain, a tab or space character, and the base-sequence for the domain. Domain names may contain: (1) upper or lower case letters, (2) numbers, (3) dash (-), and (4) underscore(_) characters. Base-sequences may contain upper or lower case a/t/c/g and should be specified starting from the 5’ end of the molecule.

Variable domains file. A file specifying the base-sequence of domains which are free to be modified during optimization. By default, this file is named “dp_in_domains_variable.txt” and is present in the directory where the program is run. A different file can be specified by providing a new value for IN_VARIABLE_DOMAINS in the parameters file. This file must contain one line per domain. Each line should contain (in order) a name for the domain, a tab or space character, and the base-sequence for the domain. Domain names may contain: (1) upper or lower case letters, (2) numbers, (3) dash (-), and (4) underscore(_) characters. Base-sequences may contain upper or lower case a/t/c/g and should be specified starting from the 5’ end of the molecule.

Oligomers file. A file specifying the binding domains on each oligomer. By default, this file is named “dp_in_oligomers.txt” and is present in the directory where the program is run. A different file can be specified by providing a new value for IN_OLIGOMERS in the parameters file. This file should contain one line per oligomer. Each line should contain (in order) a name for the oligomer, a tab or space character, and a space-separated sequence of domains or domain/complements. Oligomer names may contain: (1) upper or lower case letters, (2) numbers, (3) dash (-), and (4) underscore(_) characters. The sequence of domains on the oligomer should be specified starting at the 5’ end of the molecule. The sequence of domains may contain any domain specified in the domain files, or the binding complement (i.e., reverse complement) of a domain specified in the domain files. The binding complement of a named binding domain can be indicated using “c.” and the domain name.

Parameters File. A file specifying the runtime parameters for the program to use. By default, this file is named “dp_parameters.txt” and is present in the directory where the program is run. A different parameters file can be specified when running the program (*i.e.*, “Java -jar DevPro.jar other_parameters.txt”). The format of the parameters file should contain one line per parameter. Each line should contain (in order) a parameter name, a space or tab character, and a value for the parameter. Example parameters files can be found in the examples

folder. An example parameters file containing all available parameters can be created from DevPro by using the -ep option (*i.e.*, “java -jar DevPro.jar -ep”).

RUNNING DEVPRO

If the DevPro jar file is not already present, it must be compiled before the program can be run. This can be done by following the instructions in the jar/README.txt file.

To run DevPro: (1) open a command line, (2) navigate to the directory containing the input files, and (3) execute the command “java -jar DevPro.jar”. If the jar file is not located in the current directory, you will need to specify the correct location for it. For example, when running the example in the 2007_Zhang-et-al_Autocatalytic directory, the command would be “java -jar ../../jar/DevPro.jar”.

AVAILABLE PARAMETERS

The following parameters can be provided to DevPro in the parameters file.

Output files and properties are assigned 3-character labels based on the below listed rules. The term necessary refers to duplexes which are implied by either the domain-level-design or by fixed base-sequences. The term unnecessary describes all other duplexes. The term prominent refers to duplexes which are not part of a larger duplex. The term largest refers to the duplexes with the most base-pairs. For example, the counts of unnecessary intra-oligomer duplexes are reported in the PROP_CUA property and OUT_CUA file.

Character 1:
- (C) Counts of each length
- (D) Details of duplex location and base-sequence
- (L) Details of duplex location and base-sequence for the largest prominent duplexes
- (P) Counts of each length for prominent duplexes
- (S) Size of the largest duplex in base-pairs

Character 2:
- (N) Necessary duplexes only
- (U) Unnecessary duplexes only
- (X) Both necessary and unnecessary

Character 3:
- (A) Intra-Oligomer duplexes only
- (E) Inter-Oligomer duplexes only

INPUT FILE PARAMETERS

IN_FIXED_DOMAINS - Text file listing the fixed domains for the network. The default value is “dp_in_domains_fixed.txt”.

IN_VARIABLE_DOMAINS - Text file listing the variable domains for the network. The default value is “dp_in_domains_variable.txt”.

IN_OLIGOMERS - Text file listing the oligomers for the network. The default value is “dp_in_oligomers.txt”.

ANALYSIS PARAMETERS

interSB – Abbreviation of inter-oligomer scoring base. The fitness points contributed by each inter-oligomer duplex are equal to this value raised to the length of the duplex. Must be an integer greater than or equal to 0 and less than 2147483647. The default value is 10.

interSLC - Abbreviation of inter-oligomer scoring length criterion. Inter-oligomer duplexes with base-pairs less than this value do not contribute to counts or scores. Must be an integer greater than or equal to 1 and less than 2147483647. The default value is 1.

intraSB – Abbreviation of intra-oligomer scoring base. The fitness points contributed by each intra-oligomer duplex are equal to this value raised to the length of the duplex. Must be an integer greater than or equal to 0 and less than 2147483647. The default value is 10.

intraSLC - Abbreviation of intra-oligomer scoring length criterion. Intra-oligomer duplexes with base-pairs less than this value do not contribute to counts or scores. Must be an integer greater than or equal to 1 and less than 2147483647. The default value is 1.

numberLargestDuplexes - Maximum number of duplexes to include when listing the largest duplexes. Must be an integer greater than or equal to 1 and less than 2147483647. The default value is 1000.

scoringWeightX - Wx will be calculated as O times this value plus N. Must be an integer greater than or equal to 1 and less than 2147483647. The default value is 10,000.

REQUESTABLE PROPERTY PARAMETERS

PROP_baselineN - Network Fitness Score resulting from necessary duplexes. Must be true or false. A value of true will request this property. The default value of this parameter is false.

PROP_baselineO - Oligomer Fitness Score resulting from necessary duplexes. Must be true or false. A value of true will request this property. The default value of this parameter is false.

PROP_baselineW - Weighted Fitness Score resulting from necessary duplexes. Must be true or false. A value of true will request this property. The default value of this parameter is false.

PROP_deltaN - Network Fitness Score resulting from unnecessary duplexes. Must be true or false. A value of true will request this property. The default value of this parameter is true.

PROP_deltaO - Oligomer Fitness Score resulting from unnecessary duplexes. Must be true or false. A value of true will request this property. The default value of this parameter is true.

PROP_deltaW - Weighted Fitness Score resulting from unnecessary duplexes. Must be true or false. A value of true will request this property. The default value of this parameter is true.

PROP_N - Network Fitness Score. Must be true or false. A value of true will request this property. The default value of this parameter is false.

PROP_O - Oligomer Fitness Score. Must be true or false. A value of true will request this property. The default value of this parameter is false.

PROP_W - Weighted Fitness Score. Must be true or false. A value of true will request this property. The default value of this parameter is false.

PROP_CXA - Profile of the length-counts for all intra-oligomer duplexes. Must be true or false. A value of true will request this property. The default value of this parameter is false.

PROP_LXA - List of the largest intra-oligomer duplexes. Must be true or false. A value of true will request this property. The default value of this parameter is false.

PROP_PXA - Profile of the length-counts for the most prominent intra-oligomer duplexes. Must be true or false. A value of true will request this property. The default value of this parameter is false.

PROP_DXA - List of all intra-oligomer duplexes. Must be true or false. A value of true will request this property. The default value of this parameter is false.

PROP_CXE - Profile of the length-counts for all inter-oligomer duplexes. Must be true or false. A value of true will request this property. The default value of this parameter is false.

PROP_LXE - List of the largest inter-oligomer duplexes. Must be true or false. A value of true will request this property. The default value of this parameter is false.

PROP_PXE - Profile of the length-counts for the most prominent inter-oligomer duplexes. Must be true or false. A value of true will request this property. The default value of this parameter is false.

PROP_DXE - List of all inter-oligomer duplexes. Must be true or false. A value of true will request this property. The default value of this parameter is false.

PROP_CNA - Profile of the length-counts for baseline intra-oligomer duplexes. Must be true or false. A value of true will request this property. The default value of this parameter is false.

PROP_LNA - List of the largest baseline intra-oligomer duplexes. Must be true or false. A value of true will request this property. The default value of this parameter is false.

PROP_PNA - Profile of the length-counts for the most prominent baseline intra-oligomer duplexes. Must be true or false. A value of true will request this property. The default value of this parameter is false.

PROP_DNA - List of baseline intra-oligomer duplexes. Must be true or false. A value of true will request this property. The default value of this parameter is false.

PROP_CNE - Profile of the length-counts for baseline inter-oligomer duplexes. Must be true or false. A value of true will request this property. The default value of this parameter is false.

PROP_LNE - List of the largest baseline inter-oligomer duplexes. Must be true or false. A value of true will request this property. The default value of this parameter is false.

PROP_PNE - Profile of the length-counts for the most prominent baseline inter-oligomer duplexes. Must be true or false. A value of true will request this property. The default value of this parameter is false.

PROP_DNE - List of baseline inter-oligomer duplexes. Must be true or false. A value of true will request this property. The default value of this parameter is false.

PROP_CUA - Profile of the length-counts for unnecessary intra-oligomer duplexes. Must be true or false. A value of true will request this property. The default value of this parameter is false.

PROP_LUA - List of the largest unnecessary intra-oligomer duplexes. Must be true or false. A value of true will request this property. The default value of this parameter is false.

PROP_PUA - Profile of the length-counts for the most prominent unnecessary intra-oligomer duplexes. Must be true or false. A value of true will request this property. The default value of this parameter is false.

PROP_DUA - List of unnecessary intra-oligomer duplexes. Must be true or false. A value of true will request this property. The default value of this parameter is false.

PROP_CUE - Profile of the length-counts for unnecessary inter-oligomer duplexes. Must be true or false. A value of true will request this property. The default value of this parameter is false.

PROP_LUE - List of the largest unnecessary inter-oligomer duplexes. Must be true or false. A value of true will request this property. The default value of this parameter is false.

PROP_PUE - Profile of the length-counts for the most prominent unnecessary inter-oligomer duplexes. Must be true or false. A value of true will request this property. The default value of this parameter is false.

PROP_DUE - List of unnecessary inter-oligomer duplexes. Must be true or false. A value of true will request this property. The default value of this parameter is false.

PROP_SUA - Size of the largest unnecessary intra-oligomer duplex. Must be true or false. A value of true will request this property. The default value of this parameter is true.

PROP_SUE - Size of the largest unnecessary inter-oligomer duplex. Must be true or false. A value of true will request this property. The default value of this parameter is true.

OUTPUT PARAMETERS

outputDirectory – Directory where output files will be placed. Default value is ‘output\’.

OUT_FILE_REPORT - Text file detailing key results and parameters used. Value must be either “false” or end with “.txt”. The default value is “se_out_report.txt”.

OUT_CXA - Profile of the length-counts for all intra-oligomer duplexes. Must be either a file name ending with .csv or false. The default value of this parameter is false.

OUT_LXA - List of the largest unique intra-oligomer duplexes. Must be either a file name ending with .csv or false. The default value of this parameter is false.

OUT_PXA - Profile of the length-counts for the most prominent intra-oligomer duplexes. The default value of this parameter is false.

OUT_DXA - List of all prominent intra-oligomer duplexes. Must be either a file name ending with .csv or false. The default value of this parameter is false.

OUT_CXE - Profile of the length-counts for all inter-oligomer duplexes. Must be either a file name ending with .csv or false. The default value of this parameter is false.

OUT_LXE - List of all unique inter-oligomer duplexes. Must be either a file name ending with .csv or false. The default value of this parameter is false.

OUT_PXE - Profile of the length-counts for the most prominent inter-oligomer duplexes. Must be either a file name ending with .csv or false. The default value of this parameter is false.

OUT_DXE - List of all prominent inter-oligomer duplexes. Must be either a file name ending with .csv or false. The default value of this parameter is false.

OUT_CNA - Profile of the length-counts for necessary intra-oligomer duplexes. Must be either a file name ending with .csv or false. The default value of this parameter is “dp_out_Counts_Necessary_Intra.csv”.

OUT_LNA - List of baseline unique intra-oligomer duplexes. Must be either a file name ending with .csv or false. The default value of this parameter is “dp_out_Details_Necessary_Intra_Largest.csv”.

OUT_PNA - Profile of the length-counts for the most prominent necessary intra-oligomer duplexes. Must be either a file name ending with .csv or false. The default value of this parameter is false.

OUT_DNA - List of necessary prominent intra-oligomer duplexes. Must be either a file name ending with .csv or false. The default value of this parameter is false.

OUT_CNE - Profile of the length-counts for necessary inter-oligomer duplexes. Must be either a file name ending with .csv or false. The default value of this parameter is “dp_out_Counts_Necessary_Inter.csv”.

OUT_LNE - List of baseline unique inter-oligomer duplexes. Must be either a file name ending with .csv or false. The default value of this parameter is “dp_out_Details_Necessary_Inter_Largest.csv”.

OUT_PNE - Profile of the length-counts for the most prominent necessary inter-oligomer duplexes. Must be either a file name ending with .csv or false. The default value of this parameter is false.

OUT_DNE - List of necessary prominent inter-oligomer duplexes. Must be either a file name ending with .csv or false. The default value of this parameter is false.

OUT_CUA - Profile of the length-counts for unnecessary intra-oligomer duplexes. Must be either a file name ending with .csv or false. The default value of this parameter is “dp_out_Counts_Unnecessary_Intra.csv”.

OUT_LUA - List of delta unique intra-oligomer duplexes. Must be either a file name ending with .csv or false. The default value of this parameter is “dp_out_Details_Unnecessary_Intra_Largest.csv”.

OUT_PUA - Profile of the length-counts for the most prominent unnecessary intra-oligomer duplexes. Must be either a file name ending with .csv or false. The default value of this parameter is false.

OUT_DUA - List of unnecessary prominent intra-oligomer duplexes. Must be either a file name ending with .csv or false. The default value of this parameter is false.

OUT_CUE - Profile of the length-counts for unnecessary inter-oligomer duplexes. Must be either a file name ending with .csv or false. The default value of this parameter is “dp_out_Counts_Unnecessary_Inter.csv”.

OUT_LUE - List of delta unique inter-oligomer duplexes. Must be either a file name ending with .csv or false. The default value of this parameter is “dp_out_Details_Unnecessary_Inter_Largest.csv”.

OUT_PUE - Profile of the length-counts for the most prominent unnecessary inter-oligomer duplexes. Must be either a file name ending with .csv or false. The default value of this parameter is false.

OUT_DUE - List of unnecessary prominent inter-oligomer duplexes. Must be either a file name ending with .csv or false. The default value of this parameter is false.

OUTPUT FILES

By default, running DevPro produces the following output files: (1) the counts of the necessary inter-oligomer duplexes, (2) the counts of the necessary intra-oligomer duplexes, (3) the counts of the unnecessary inter-oligomer duplexes, (4) the counts of the unnecessary intra-oligomer duplexes, (5) the details of the largest necessary inter-oligomer duplexes, (6) the details of the largest necessary intra-oligomer duplexes, (7) the details of the largest unnecessary inter-oligomer duplexes, (8) the details of the largest unnecessary intra-oligomer duplexes, and (9) a report file. Additional output files can be activated using the parameters listed above.

dp_out_counts_necessary_inter.csv – A comma separated values (csv) file listing the number of necessary inter-oligomer duplexes of each size. The location and name of this output file can be changed by modifying the OUT_CNE parameter. This file can be disabled by providing the value 'false' for this parameter. The counts in this file include duplexes which are part of a larger duplex (i.e., each 8 base-pair duplex implies 2 x 7 base-pair duplexes, 3 x 6 base-pair duplexes, etc). A similar file which reports counts calculated without sub-duplexes can be activated using the OUT_PNE parameter.

dp_out_counts_necessary_intra.csv – A comma separated values (csv) file listing the number of necessary intra-oligomer duplexes of each size. The location and name of this output file can be changed by modifying the OUT_CNA parameter. This file can be disabled by providing the value 'false' for this parameter. The counts in this file include duplexes which are part of a larger duplex (i.e., each 8 base-pair duplex implies 2 x 7 base-pair duplexes, 3 x 6 base-pair duplexes, etc). A similar file which reports counts calculated without sub-duplexes can be activated using the OUT_PNA parameter.

dp_out_counts_unnecessary_inter.csv – A comma separated values (csv) file listing the number of unnecessary inter-oligomer duplexes of each size. The location and name of this output file can be changed by modifying the OUT_CUE parameter. This file can be disabled by providing the value 'false' for this parameter. The counts in this file include duplexes which are part of a larger duplex (i.e., each 8 base-pair duplex implies 2 x 7 base-pair duplexes, 3 x 6 base-pair duplexes, etc). A similar file which reports counts calculated without sub-duplexes can be activated using the OUT_PUE parameter.

dp_out_counts_unnecessary_intra.csv – A comma separated values (csv) file listing the number of unnecessary intra-oligomer duplexes of each size. The location and name of this output file can be changed by modifying the OUT_CUA parameter. This file can be disabled by providing the value 'false' for this parameter. The counts in this file include duplexes which are part of a larger duplex (i.e., each 8 base-pair duplex implies 2 x 7 base-pair duplexes, 3 x 6 base-pair duplexes, etc). A similar file which reports counts calculated without sub-duplexes can be activated using the OUT_PUA parameter.

dp_out_details_necessary_inter_largest.csv – A comma separated values (csv) file detailing the largest necessary inter-oligomer duplexes. The details include (1) the size of the duplex, (2) the first oligomer involved, (3) the base-sequence involved from the first oligomer, (4) the location of the base-sequence involved from the first oligomer, (5) the second oligomer involved, (6) the base sequences involved from the second oligomer, and (7) the location of the base-sequences involved from the second oligomer. The index of the 5' most base of the duplex relative to

the 5’ most base of the oligomer is used to report location. Alternatively stated, an index of 0 indicates that the 5’ most base of the duplex is the 5’ most base of the oligomer. The location and name of this output file can be changed by modifying the OUT_LNE parameter. This file can be disabled by providing the value ‘false’ for this parameter. This file does not include duplexes which are part of a larger duplex (i.e., each 8 base-pair duplex implies 2 x 7 base-pair duplexes, 3 x 6 base-pair duplexes, etc). A similar file which includes duplexes which are part of a larger duplex can be activated using the OUT_DNE parameter.

dp_out_details_necessary_intra_largest.csv – A comma separated values (csv) file detailing the largest necessary intra-oligomer duplexes. The details include (1) the size of the duplex, (2) the first oligomer involved, (3) the base-sequence involved from the first oligomer, (4) the location of the base-sequence involved from the first oligomer, (5) the second oligomer involved, (6) the base sequences involved from the second oligomer, and (7) the location of the base-sequences involved from the second oligomer. The two oligomers should always be the same for this file. The index of the 5’ most base of the duplex relative to the 5’ most base of the oligomer is used to report location. Alternatively stated, an index of 0 indicates that the 5’ most base of the duplex is the 5’ most base of the oligomer. The location and name of this output file can be changed by modifying the OUT_LNA parameter. This file can be disabled by providing the value ‘false’ for this parameter. This file does not include duplexes which are part of a larger duplex (i.e., each 8 base-pair duplex implies 2 x 7 base-pair duplexes, 3 x 6 base-pair duplexes, etc). A similar file which includes duplexes which are part of a larger duplex can be activated using the OUT_DNA parameter.

dp_out_details_unnecessary_inter_largest.csv – A comma separated values (csv) file detailing the largest unnecessary inter-oligomer duplexes. The details include (1) the size of the duplex, (2) the first oligomer involved, (3) the base-sequence involved from the first oligomer, (4) the location of the base-sequence involved from the first oligomer, (5) the second oligomer involved, (6) the base sequences involved from the second oligomer, and (7) the location of the base-sequences involved from the second oligomer. The index of the 5’ most base of the duplex relative to the 5’ most base of the oligomer is used to report location. Alternatively stated, an index of 0 indicates that the 5’ most base of the duplex is the 5’ most base of the oligomer. The location and name of this output file can be changed by modifying the OUT_LUE parameter. This file can be disabled by providing the value ‘false’ for this parameter. This file does not include duplexes which are part of a larger duplex (i.e., each 8 base-pair duplex implies 2 x 7 base-pair duplexes, 3 x 6 base-pair duplexes, etc). A similar file which includes duplexes which are part of a larger duplex can be activated using the OUT_DUE parameter.

dp_out_details_unnecessary_intra_largest.csv – A comma separated values (csv) file detailing the largest unnecessary intra-oligomer duplexes. The details include (1) the size of the duplex, (2) the first oligomer involved, (3) the base-sequence involved from the first oligomer, (4) the location of the base-sequence involved from the first oligomer, (5) the second oligomer involved, (6) the base sequences involved from the second oligomer, and (7) the location of the base-sequences involved from the second oligomer. The two oligomers should always be the same for this file. The index of the 5’ most base of the duplex relative to the 5’ most base of the oligomer is used to report location. Alternatively stated, an index of 0 indicates that the 5’ most base of the duplex is the 5’ most base of the oligomer. The location and name of this output file can be changed by modifying the OUT_LUA parameter. This file can be disabled by providing the value ‘false’ for this parameter. This file does not include duplexes which are part of a larger duplex (i.e., each 8 base-pair duplex implies 2 x 7 base-pair duplexes, 3 x 6 base-pair duplexes, etc). A similar file which includes duplexes which are part of a larger duplex can be activated using the OUT_DUA parameter.

dp_out_report.txt – A text file listing numerous details from the run. This includes: (1) any requested properties, (2) any properties which were necessary to calculate the requested properties, (3) the base-sequence for each

fixed domain, (4) the base-sequence of each variable domain, (5) the domains on each oligomer, (6) the base-sequence of each oligomer, (7) the value of all parameters used. The location and name of this output file can be changed by modifying the OUT_FILE_REPORT parameter. This file can be disabled by providing the value ‘false’ for this parameter.
