# Supplementary material for: Generation of DNA oligomers with similar chemical kinetics via in-silico optimization
Source: Commun Chem. 2023 Oct 18;6:226. doi: 10.1038/s42004-023-01026-w (PMC10584830; doi:10.1038/s42004-023-01026-w)
Supplement: Supplementary file 5 — Supplementary Software 1 [file 42004_2023_1026_MOESM5_ESM.zip › doc/SeqEvo_Manual.pdf]

USER'S MANUAL FOR SEQEVO

SYSTEM REQUIREMENTS

SeqEvo requires a Java Development Kit (such as the one at https://openjdk.org/) to be installed. The current code has been verified to run with openJDK 21 (https://jdk.java.net/21/). Instructions for installing openJDK can be found here (https://stackoverflow.com/questions/52511778/how-to-install-openjdk-11-on-windows).

INPUT FILES

Running SeqEvo requires 4 input files: (1) A "fixed domains" file, (2) a "variable domains" file, (3) an "oligomers" file, and (4) a "parameters" file.

Fixed domains file. A file specifying the base-sequence of domains which will not be modified during optimization. Any duplex containing only fixed sequences is part of a network's design and is considered necessary (i.e., they do not contribute to certain fitness scores). By default, this file is named "se_in_domains_fixed.txt" and is present in the directory where the program is run. A different file can be specified by providing a new value for IN_FIXED_DOMAINS in the parameters file. This file must contain one line per domain. Each line should contain (in order) a name for the domain, a tab or space character, and the base-sequence for the domain. Domain names may contain: (1) upper or lower case letters, (2) numbers, (3) dash (-), and (4) underscore(_) characters. Base-sequences may contain upper or lower case a/t/c/g and should be specified starting from the 5' end of the molecule.

Variable domains file. A file specifying the base-sequence of domains which will be modified during optimization. By default, this file is named "se_in_domains_variable.txt" and is present in the directory where the program is run. A different file can be specified by providing a new value for IN_VARIABLE_DOMAINS in the parameters file. This file must contain one line per domain. Each line should contain (in order) a name for the domain, a tab or space character, and the base-sequence for the domain. Domain names may contain: (1) upper or lower case letters, (2) numbers, (3) dash (-), and (4) underscore(_) characters. Base-sequences may contain upper or lower case a/t/c/g and should be specified starting from the 5' end of the molecule.

Oligomers file. A file specifying the binding domains on each oligomer. By default, this file is named "se_in_oligomers.txt" and is present in the directory where the program is run. A different file can be specified by providing a new value for IN_OLIGOMERS in the parameters file. This file should contain one line per oligomer. Each line should contain (in order) a name for the oligomer, a tab or space character, and a space-separated sequence of domains or domain/complements. Oligomer names may contain: (1) upper or lower case letters, (2) numbers, (3) dash (-), and (4) underscore(_) characters. The sequence of domains on the oligomer should be specified starting at the 5' end of the molecule. The sequence of domains may contain any domain specified in the domain files, or the binding complement (i.e., reverse complement) of a domain specified in the domain files. The binding complement of a named binding domain can be indicated using "c." and the domain name.

Parameters File. A file specifying the runtime parameters for the program to use. By default, this file is named "se_parameters.txt" and is present in the directory where the program is run. A different parameters file can be specified when running the SeqEvo command (i.e., "Java -jar SeqEvo.jar other_parameters.txt"). The format of the parameters file should contain one line per parameter. Each line should contain (in order) a parameter name, a space or tab character, and a value for the parameter. Example parameters files can be found in the examples

folder. An example parameters file containing all available parameters for the program can be created from SeqEvo by using the -ep option.

RUNNING SEQEVO

If the SeqEvo jar file is not already present, it must be compiled before SeqEvo can be run. This can be done by following the instructions in the jar/README.txt file.

To run SeqEvo: (1) open a command line, (2) navigate to the directory containing the input files, and (3) execute the command "java -jar SeqEvo.jar". If the jar file is not located in the current directory, you will need to specify the correct location for it. For example, when running the example in the 2007_Zhang-et-al_Autocatalytic directory, the command would be "java -jar ../../jar/SeqEvo.jar".

AVAILABLE PARAMETERS

The following parameters can be provided to SeqEvo in the parameters file.

INPUT FILE PARAMETERS

IN_FIXED_DOMAINS - Text file listing the fixed domains for the network. The default value is "se_in_domains_fixed.txt".

IN_VARIABLE_DOMAINS - Text file listing the variable domains for the network. The default value is "se_in_domains_variable.txt".

IN_OLIGOMERS - Text file listing the oligomers for the network. The default value is "se_in_oligomers.txt".

HEURISTIC PARAMETERS

CPL - Cycles-Per-Lineage. Must be an integer greater than or equal to 1 and less than 2147483647. The default value is 100,000.

GPC - Generations-Per-Cycle. Must be an integer greater than or equal to 1 and less than 2147483647. The default value is 1.

NDPG - New-Daughters-Per-Generation. Must be an integer greater than or equal to 1 and less than 2147483647. The default value is 1.

NL - Number-of-Lineages. Must be an integer greater than or equal to 1 and less than 2147483647. The default value is 8.

NMPC - New-Mothers-Per-Cycle. Must be an integer greater than or equal to 1 and less than 2147483647. The default value is 2.

SCORING PARAMETERS

FITNESS_SCORE - Fitness score to optimize. The default and only accepted value is Wx.

interSB – The fitness points contributed by each inter-oligomer duplex are equal to this value raised to the length of the duplex. Must be an integer greater than or equal to 0 and less than 2147483647. The default value is 10.

interSLC - Inter-oligomer duplexes with base-pairs less than this value do not contribute to counts or scores. Must be an integer greater than or equal to 1 and less than 2147483647. The default value is 1.

intraSB – The fitness points contributed by each intra-oligomer duplex are equal to this value raised to the length of the duplex. Must be an integer greater than or equal to 0 and less than 2147483647. The default value is 10.

intraSLC - Intra-oligomer duplexes with base-pairs less than this value do not contribute to counts or scores. Must be an integer greater than or equal to 1 and less than 2147483647. The default value is 1.

maxAA - Maximum number of consecutive adenosine bases. Any stretch of bases greater than this number will make a network invalid. Must be an integer greater than or equal to 1 and less than 2147483647. The default value is 6.

maxCC - Maximum number of consecutive cytosine bases. Any stretch of bases greater than this number will make a network invalid. Must be an integer greater than or equal to 1 and less than 2147483647. The default value is 3.

maxGG - Maximum number of consecutive guanine bases. Any stretch of bases greater than this number will make a network invalid. Must be an integer greater than or equal to 1 and less than 2147483647. The default value is 3.

maxTT - Maximum number of consecutive thymine bases. Any stretch of bases greater than this number will make a network invalid. Must be an integer greater than or equal to 1 and less than 2147483647. The default value is 6.

scoringWeightX - Wx will be calculated as O times this value plus N. Must be an integer greater than or equal to 1 and less than 2147483647. The default value is 10,000.

OUTPUT PARAMETERS

OUT_FILE_REPORT - Text file detailing key results and parameters used. Value must be either “false” or end with “.txt”. The default value is “se_out_report.txt”.

OUT_FILE_DOMAINS_VARIABLE - Text file listing the base-sequence of the variable domains following optimization. Value must be either “false” or end with “.txt”. The default value is “se_out_domains_variable.txt”.

OUT_FILE_OLIGOMERS - Text file listing the base-sequence of the oligomers following optimization. Value must be either “false” or end with “.txt”. The default value is “se_out_oligomers.txt”.

OUT_FILE_SCORES - Text file listing the scores of the networks in each generation. Value must be either “false” or end with “.csv”. The default value is “se_out_score_trajectories.csv”.

OUT_FILE_LOG_SCORES - Text file listing the scores of the networks in logarithmically distributed generations. Value must be either “false” or end with “.csv”. The default value is “se_out_score_trajectories_log.csv”.

OUTPUT FILES

By default, running SeqEvo produces the following output files: (1) a variable domains file, (2) an oligomers file, (3) a report file, (4) a score trajectories file, and (5) a log score trajectories file.

se_out_domains_variable.txt – A text file listing the base-sequence of variable designs following optimization. Alternatively stated, this file contains the variable domains of the most-fit network encountered during the search.

The location and name of this output file can be changed by modifying the OUT_FILE_DOMAINS_VARIABLE parameter. This file can be disabled by providing the value 'false' for this parameter.

se_out_oligomers.txt – A text file listing the base-sequence of each oligomer following optimization. Alternatively stated, this file contains the oligomer base-sequences of the most-fit network encountered during the search. The location and name of this output file can be changed by modifying the OUT_FILE_OLIGOMERS parameter. This file can be disabled by providing the value 'false' for this parameter.

se_out_report.txt – A text file listing numerous details from the run. This includes: (1) the fitness scores of the initial and final networks, (2) values for all runtime parameters used, (3) the sequences for each fixed domain (4) the initial sequence of each variable domain, (5) the domains on each oligomer, (6) the initial sequence of each oligomer, (7) the final sequence of each variable domain, (8) the final sequence of each oligomer. The location and name of this output file can be changed by modifying the OUT_FILE_REPORT parameter. This file can be disabled by providing the value 'false' for this parameter.

se_out_score_trajectories.csv – A comma separated values (csv) file listing the score of each lineage at the end of each evolutionary cycle. Generation 1 in this file represents initial lineage mother created for the lineage. This file is useful for determining the effectiveness of the heuristic search, however this file may become inconveniently large for some parameter settings. The location and name of this output file can be changed by modifying the OUT_FILE_SCORES parameter. This file can be disabled by providing the value 'false' for this parameter.

se_out_score_trajectories_log.csv – A comma separated values (csv) file listing the score of each lineage at the of select evolutionary cycles. Cycle scores are recorded every time the total number of generations double. Generation 1 in this file represents initial lineage mother created for the lineage. The size of this file remains reasonably even for parameters where the se_out_score_trajectories.csv file is inconveniently large. The location and name of this output file can be changed by modifying the OUT_FILE_LOG_SCORES parameter. This file can be disabled by providing the value 'false' for this parameter.
